# Di-*meta*-Substituted Fluorinated Benzenesulfonamides as Potent and Selective Anticancer Inhibitors of Carbonic Anhydrase IX and XII

**DOI:** 10.1021/acs.jmedchem.5c01142

**Published:** 2025-08-20

**Authors:** Aivaras Vaškevičius, Mantas Žvirblis, Maija Kurtenoka, Janis Leitans, Elena Manakova, Vaida Paketurytė-Latvė, Agnė Kvietkauskaitė, Andris Kazaks, Vladislava Eimonta, Kamilė Čerepenkaitė, Justina Kazokaitė-Adomaitienė, Aurelija Mickevičiu̅tė, Vaida Juozapaitienė, Kaspars Tars, Saulius Gražulis, Jurgita Matulienė, Virginija Dudutienė, Kirill Shubin, Daumantas Matulis, Asta Zubrienė

**Affiliations:** 1 Department of Biothermodynamics and Drug Design, Institute of Biotechnology, Life Sciences Center, 54694 Vilnius University, Saulėtekio 7, Vilnius LT-10257, Lithuania; 2 Department of Organic Chemistry, 187008 Latvian Institute of Organic Synthesis, Aizkraukles 21, Riga LV-1006, Latvia; 3 382968 Latvian Biomedical Research and Study Centre, Ratsupites 1 k-1, Riga 1067, Latvia; 4 Department of Protein−DNA Interactions, Institute of Biotechnology, Life Sciences Center, 54694 Vilnius University, Saulėtekio 7, Vilnius LT-10257, Lithuania; 5 Sector of Crystallography and Chemical Informatics, Institute of Biotechnology, Life Sciences Center, 54694 Vilnius University, Saulėtekio 7, Vilnius 10257, Lithuania; 6 Division of Biochemistry, The Netherlands Cancer Institute, Amsterdam 1066 CX, The Netherlands

## Abstract

The development of selective drug candidate molecules for cancer-related carbonic anhydrase isozymes IX and XII is challenging due to high homology binding sites among 12 catalytically active isozymes. Starting from the trifluorinated benzenesulfonamide with cyclooctylamino substituent at the *meta* position, we designed and synthesized di-*meta*-substituted fluorinated benzenesulfonamides with up to 10-fold affinity improvement for CAIX, resulting in low picomolar binders. The resulting CAIX-targeting compounds showed up to 1000-fold selectivity over off-target CA isozymes. The crystal structures of CAIX and CAXII complexes with synthesized compounds revealed detailed insights into protein–ligand interactions and adopted complex conformation. The potential of compounds with reduced off-target effects as possible anticancer drugs is supported by this study.

## Graphical abstract

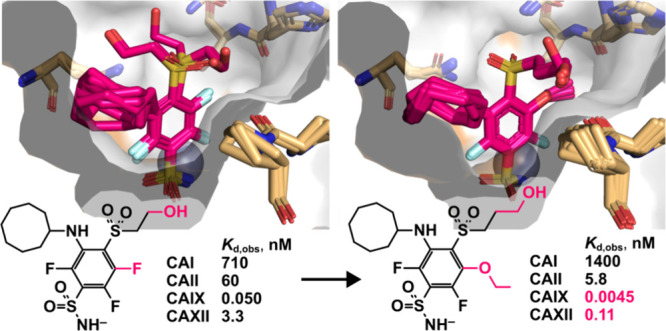

## Introduction

Optimization of the affinity and selectivity of small molecules for a target protein is a key goal of drug development. Typically, affinity optimizations are performed by introducing apolar or polar substituents to the lead drug candidate molecule. Hydrophobic groups that fill the protein cavity with good shape complementarity increase the binding affinity to the target protein. Polar groups are essential for the solubility properties of a lead compound. In addition, polar substituents that make strong hydrogen bond interactions with the target protein contribute favorably to the affinity.^1,2^

The role of carbonic anhydrase isozyme IX (CAIX) in tumor cell survival, proliferation, migration, pH regulation, and cell-signaling pathways made this enzyme a promising therapeutic target in oncology.^3,4^ Tumor cells primarily express two membrane-associated carbonic anhydrases, CAIX and CAXII.^5^ These isozymes belong to a family of zinc metalloenzymes that catalyze the reversible hydration of CO_2_ to form HCO_3_^–^ and H^+^. There are an additional ten CA isozymes in the human body with conserved catalytic activity and various distribution in tissues, which are involved in many physiological processes.^6–8^ When designing anticancer drugs that inhibit CAIX and CAXII activity, the aim is to prevent their binding to the other ten CA isozymes. Therefore, there is interest in the design of selective drugs targeting only tumor-associated CA isozymes with high affinity.

CAIX expression in normal cells is limited. The protein is found only in the gastrointestinal tract and gall bladder. However, it is strongly upregulated in different types of tumor tissues, including the brain, breast, bladder, colon, kidney, lung, ovaries, etc.^9,10^ Careful data analysis of clinical studies, which assessed the predictive value of CAIX expression in solid tumors, showed a strong correlation between high CAIX expression and poor prognosis for many different tumor types, indicating an important role of CAIX in cancer progression and treatment resistance.^11^ CAXII protein is abundant in normal tissues, but its upregulated expression is observed in several cancers: renal cell carcinomas, colorectal, breast, bladder, head, neck cancers and glioblastomas.^8^ The combined role of these two isozymes appears to be linked to tumorigenesis in various cancers.

CAIX is comprised of an N-terminal proteoglycan-like (PG) domain, an extracellularly located catalytic domain, a single transmembrane domain, and a short intracellular C-terminal domain.^12^ The intrinsically disordered PG domain is enriched in negatively charged groups and functions as a proton buffer, facilitating CAIX catalytic activity.^13^ Moreover, this domain also serves as a ‘proton antenna’ for monocarboxylate transporters, facilitating lactate flux, which both contributes to cancer cell survival under hypoxic conditions.^13^ The intracellular domain supports cancer cell migration^14^ and is essential for the proper functioning of CAIX.^15^ The catalytic domain plays a role in the enzymatic catalysis of CO_2_ hydration and is required for the CAIX-mediated pH regulation in hypoxia.^16^

Similarly to all catalytically active CA isozymes, a zinc ion is situated at the bottom of the active site of CAIX, coordinated by three histidine residues (His94, 96, and 119) and a water molecule/hydroxide ion. The relatively spacious active center of CAIX is composed of hydrophilic amino acid residues (Thr199, Thr200, His64, Pro201, and Pro202) on one side and hydrophobic amino acids (Val121, Val143, Val131, and Leu198) on the other. Although the active site is characterized by conserved amino acids between 12 isozymes, the amino acids further away from the zinc ion provide some variability.^17^ Therefore, by changing the length, size, and polarity of functional groups on the main scaffold of benzenesulfonamides, one of the most investigated CA inhibitors, the affinity for a particular CA isozyme can be drastically varied. Efforts are made to obtain CAIX and CAXII-selective compounds of high affinity and selectivity, which could be effective anticancer drugs. Both CA isozymes-inhibiting selective drugs could be easier on patients (causing limited undesirable effects).^18^

The series of our previously designed fluorinated benzenesulfonamides, such as 3-(cyclooctylamino)-2,5,6-trifluoro-4-[(2-hydroxyethyl)­sulfonyl]­benzenesulfonamide (VD11–4–2), exhibited high affinity for CAIX isozyme (*K*_d_ = 50 pM).^19,20^ The benzenesulfonamide compounds with fluorines possess lower p*K*_*a*_ of the sulfonamide group compared to analogous nonfluorinated benzenesulfonamides; therefore, fluorination of benzenesulfonamide ring substantially strengthens the interaction with CA isozymes.^21,22^ While optimizing the fluorinated benzenesulfonamide inhibitors, we noticed that their binding affinity and selectivity for cancer-associated CAIX were highly sensitive to substituents located at the *meta* or *ortho* position of fluorinated benzenesulfonamide. The bulky hydrophobic groups like cyclooctyl or cyclododecyl substituents at *ortho* or *meta* positions are necessary for favorable hydrophobic contact with the CAIX binding site. In contrast, the steric effects prevented the binding to off-target CAI and CAII isozymes.^20,23^ Previously, we found that the addition of another substituent at the *meta* position highly influences the affinity for CA isozymes.^24^ Herein, we report the synthesis of an expanded set of di-*meta*-substituted fluorinated benzenesulfonamides and the evaluation of binding affinities for all 12 catalytically active CA isozymes. The most potential compounds **13** and **14** showed excellent picomolar affinities for cancer-associated CAIX. Crystal structures of CAIX and CAXII with five compounds by using soaking or cocrystallization were determined to confirm the binding mode of the ligands and reveal insights that govern enhanced affinity.

## Results and Discussion

### Chemistry

A common synthesis of di-*meta*-substituted benzenesulfonamides started with pentafluorobenzenesulfonamide (**2**), which was synthesized from commercially available pentafluorobenzenesulfonyl chloride (Scheme 1). In the next step, using aromatic nucleophilic substitution reaction with appropriate nucleophile, such as (*N*-(2-mercaptoethyl)­acetamide (**a**) (synthesized from 2-mercaptoethylamine hydrochloride with acetic anhydride in basic conditions); 2-mercaptoethanol (**b**) or 3-mercaptopropanol (**c**) compounds **3a**–**c** were obtained. Afterward, compounds **3a**–**c** were oxidized in acetic acid by 30% H_2_O_2_ (aq) to produce appropriate sulfones **4a**–**c**. Sulfonamides **5a**–**c** were synthesized using aromatic nucleophilic substitution reaction by substituting fluorine atom at *meta* position to cyclooctylamino fragment. Compounds **6–21** were synthesized by aromatic nucleophilic substitution of compounds **5a**–**c** with appropriate nucleophiles in MeOH or DMSO. Sulfonamides **6–11** were obtained from compound **5c** by nucleophilic substitution in MeOH. Compound **12** was synthesized by removing the phenyl group from compound **11** with Pd/C (10%) in an H_2_ atmosphere using THF as solvent. Compounds **13** and **14** were synthesized using appropriate oxide in an appropriate solvent (MeOH in **13** case and EtOH in **14** case) from compound **5c**. Meanwhile, compounds **15–20** were synthesized using appropriate amine (cyclooctylamine; cyclopentylamine; cyclopropylamine; ethylamine or propylamine) in DMSO. Cyclic compound **21** synthesis was achieved by stirring compound **5c** in 1,8-diazabicyclo[5.4.0]­undec-7-ene in a pressure vial at 70 °C. In the preparation of compound **24**, compound **4a** was hydrolyzed with HCl (conc.) in MeOH, followed by protection with (Boc)_2_O and Et_3_N in THF. The obtained compound **22** was modified by introducing cyclooctylamine fragment via aromatic nucleophilic substitution in DMSO and sulfonamide **23** was obtained. Afterward, another aromatic nucleophilic substitution followed in DMSO at 75 °C to produce compound **24**.

**Scheme 1:**
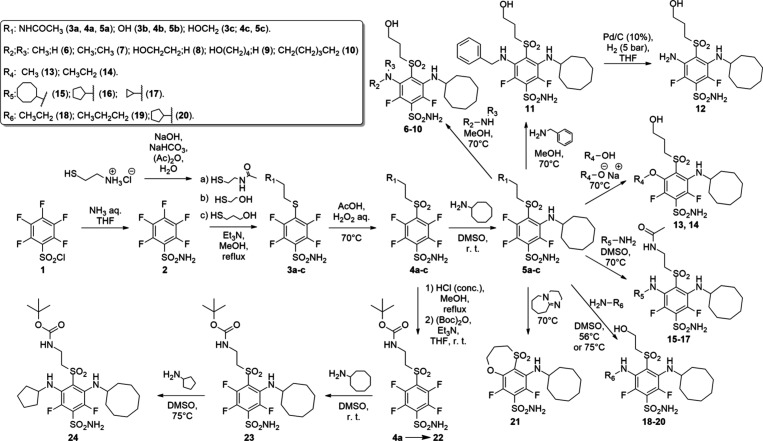
Synthesis of the Final Compounds–Primary Sulfonamide Group Containing Benzenesulfonamides

Sulfonamide **25** was synthesized from pentafluorobenzenesulfonamide **(2)** and sodium methanesulfinate via an aromatic nucleophilic substitution reaction (Scheme 2). This product was used in further *meta*-position fluorine substitutions, resulting in compounds **26–29** when using appropriate amine nucleophiles. However, instead of separate reactions, compounds **26**-**29** were successfully synthesized via one-pot synthesis.

**Scheme 2:**
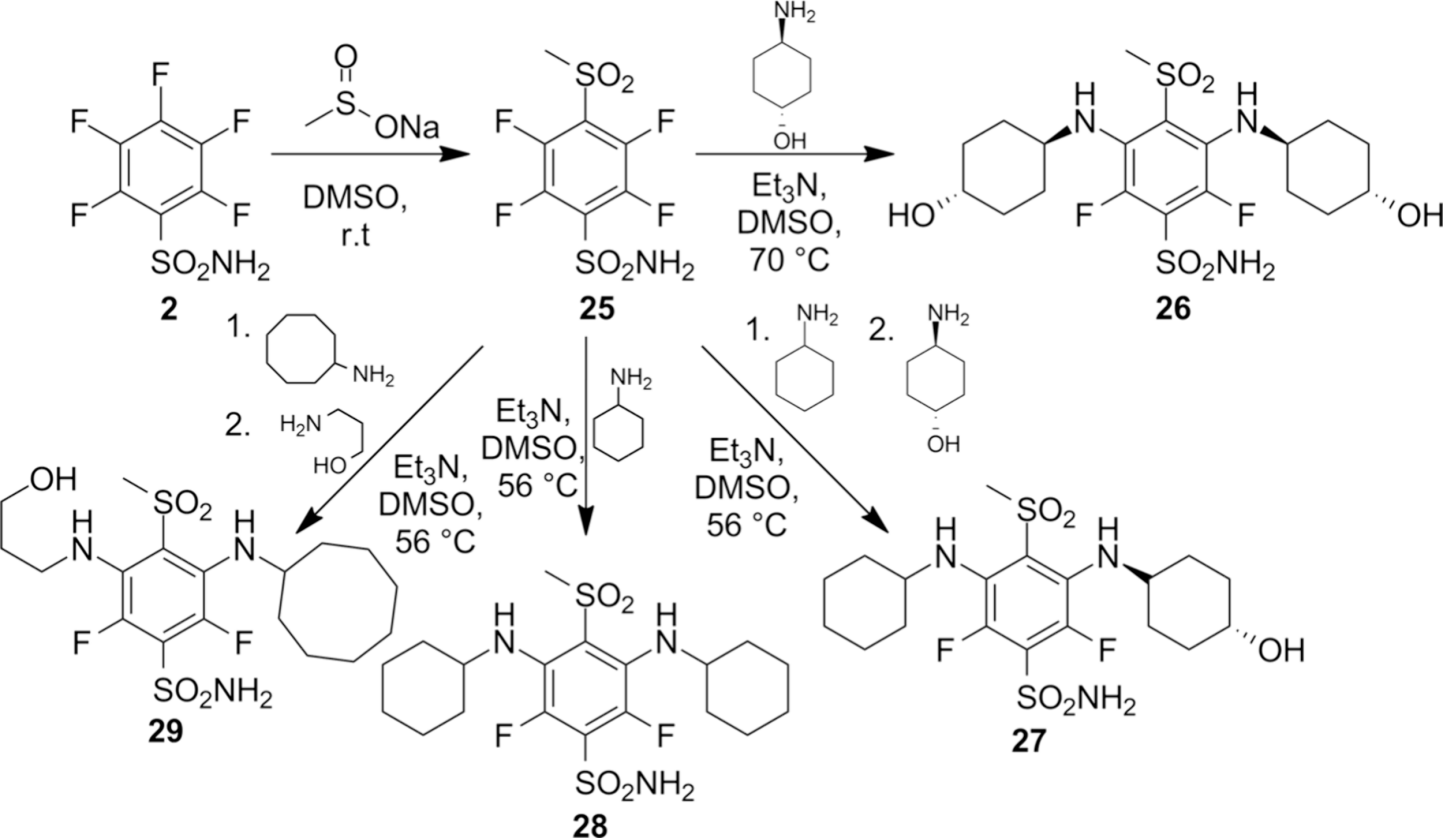
Synthesis of the Final Compounds **26**–**29**

### Compound Binding to CA Isozymes

We have previously designed compounds that inhibit cancer-associated CAIX with high affinity. Fluorinated *meta*-substituted benzenesulfonamide VD11–4–2 (compound **5b**) exhibited double-digit picomolar affinity for CAIX (*K*_d_ = 50 pM) and more than 1000 and 14000-fold selectivity over ubiquitous off-target isozymes, CA I and CA II, respectively^20^ (Table 1). Its parent tetrafluorinated compound **4b**, without cyclooctylamino group at 3-position bound most CA isozymes with nanomolar affinity, with highest affinity for CAI isozyme (*K*_d_ = 0.2 nM). *Para* substituent marginally influences the affinity, with the more hydrophobic (3-hydroxypropyl)­sulfonyl tail-bearing compound **4c** binding up to 5-fold tighter than the compounds **4a** and **4b** with 3-acetamidoethylsulfonyl and 3-hydroxyethylsulfonyl substituents, respectively. High-affinity compound VD11–4–2 exhibited insufficient selectivity against other CA isozymes and had moderate aqueous solubility. Based on the CAIX-VD11–4–2 complex structure analysis (PDB ID 6FE1),^23^ the cyclooctylamine group at the 3-position of benzenesulfonamide fits into the hydrophobic pocket of the CA IX, but the active site of the enzyme at the hydrophilic pocket is not fully occupied. We hypothesized that the selectivity and solubility of the lead compound might be improved by the introduction of a second *meta* substituent at 5-position. Our design strategy to maximize the size and bulkiness of 5-substituent to enhance binding affinity for cancer-associated protein CAIX started from compound **5c**, similar to VD11–4–2, but having extended *para* tail by one CH_2_ group. We systematically explored the impact of fluorine modification at the 5-position to different functional groups of various lengths and bulkiness on the binding affinity for 12 CA isozymes (compounds **6**-**14**, Table 1). We also varied substituents at *para* and *meta* positions of the benzenesulfonamide ring to further explore their impact on the binding affinity (compounds **15**-**20, 24** and **26–29**, Table 1).

**Table 1:**
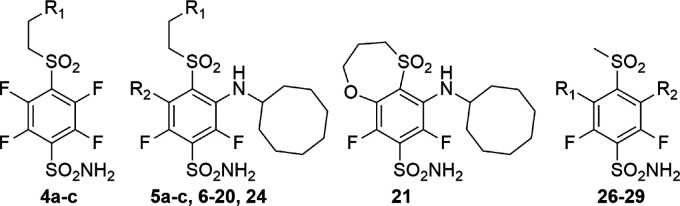

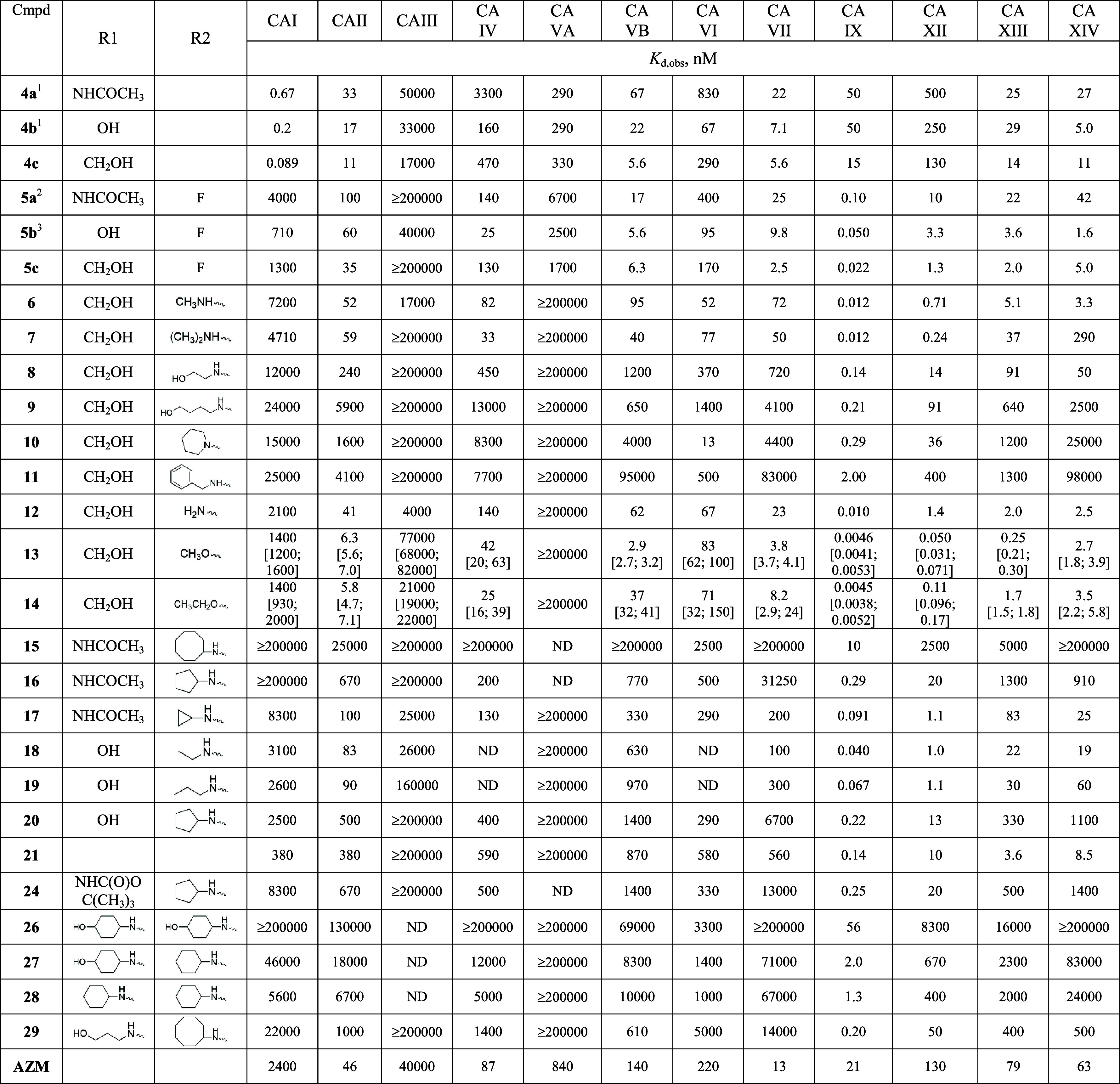
Observed Dissociation Constants *K*_d,obs_ (nM) for Compound Interaction with Human Recombinant CA Isozymes as Determined by FTSA at 37 °C and pH 7.0

1 Described by our group in ref 25.

2 Described in ref 26.

3 Potent CAIX inhibitor, VD11–4–2, described by our group in ref 20. Uncertainty of FTSA measurement is approximately 2-fold of the *K*_d_ as determined from at least two measurements. 95% confidence intervals for *K*_d_ measurements are given in brackets for the most effective compounds **13** and **14**.

The binding affinities to all 12 catalytically active CA isozymes were determined by fluorescence-based thermal shift assay (FTSA). The observed dissociation constant (*K*_d_,_obs_) values at physiological pH (pH 7.0) are presented in Table 1, while raw FTSA data and dose–response data are presented in Figure S1–S23.

We first examined the influence of linear or cyclic 5-substitutions of different lengths and hydrophobicity (compounds **6**-**14)** on the binding affinity for CA isozymes. These compounds have analogous hydroxypropyl substituent at 4-position and cyclooctylamine group at 3-position. All substituents at the 5-position of the benzene ring are bound through the N or O atom. The methylation of the amino group (mono- (**6**); di- (**7**) and unmethylated – **12**) does not affect binding affinity to CAIX, however, the affinity for several other CA isozymes decreases up to 5-fold. As a result, dimethylamino group-bearing compound **7** is more selective for CAIX compared to compounds **12** and **6** (Table S1). The elongation of substituent length and simultaneous addition of hydroxyl group (compounds **8** and **9**) decrease the binding affinity for all CAs, including CAIX with about 10-fold decline (*K*_d,obs_ 0.14 and 0.21 nM, respectively) as compared with compound **6** (*K*_d,obs_ 0.012 nM). Compound **10** with cyclic piperidinyl group retains a high affinity for CAIX (*K*_d,obs_ 0.29 nM). Interestingly, the affinity of this compound for CAVI is the highest from the whole series of di-*meta* substituted compounds and exceeds 13 nM in *K*_d,obs_. The introduction of a more flexible benzylamino substituent (compound **11**) decreases the binding with most CAs, but the selectivity for CAIX remains high (more than 200-fold selectivity).

The introduction of methoxy (compound **13**) and ethoxy (compound **14**) groups at the 5-position of fluorinated benzenesulfonamide **5c** resulted in the most strongly binding compounds for CAIX, with the *K*_d,obs_ reaching 4.5 pM at pH 7.0 (Figure 1A, C). Figure 1C shows that in the case of tight ligand binding (compounds **5c**, **13**, **14**) CAIX melting temperature (*T*_m_) reaches its highest value upon saturation of the protein (at 1:1 stochiometric protein: ligand ratio) and does not increase with increasing ligand concentration according to the model. This could be due to the long residence time and very slow off-rates of tight binders, making it impossible to achieve complete equilibrium. However, the dosing curve model^27^ fits the experimental data point at a 1:1 stochiometric protein: ligand ratio, allowing us to determine *K*_d,obs_ with sufficient accuracy.

**Figure 1:**
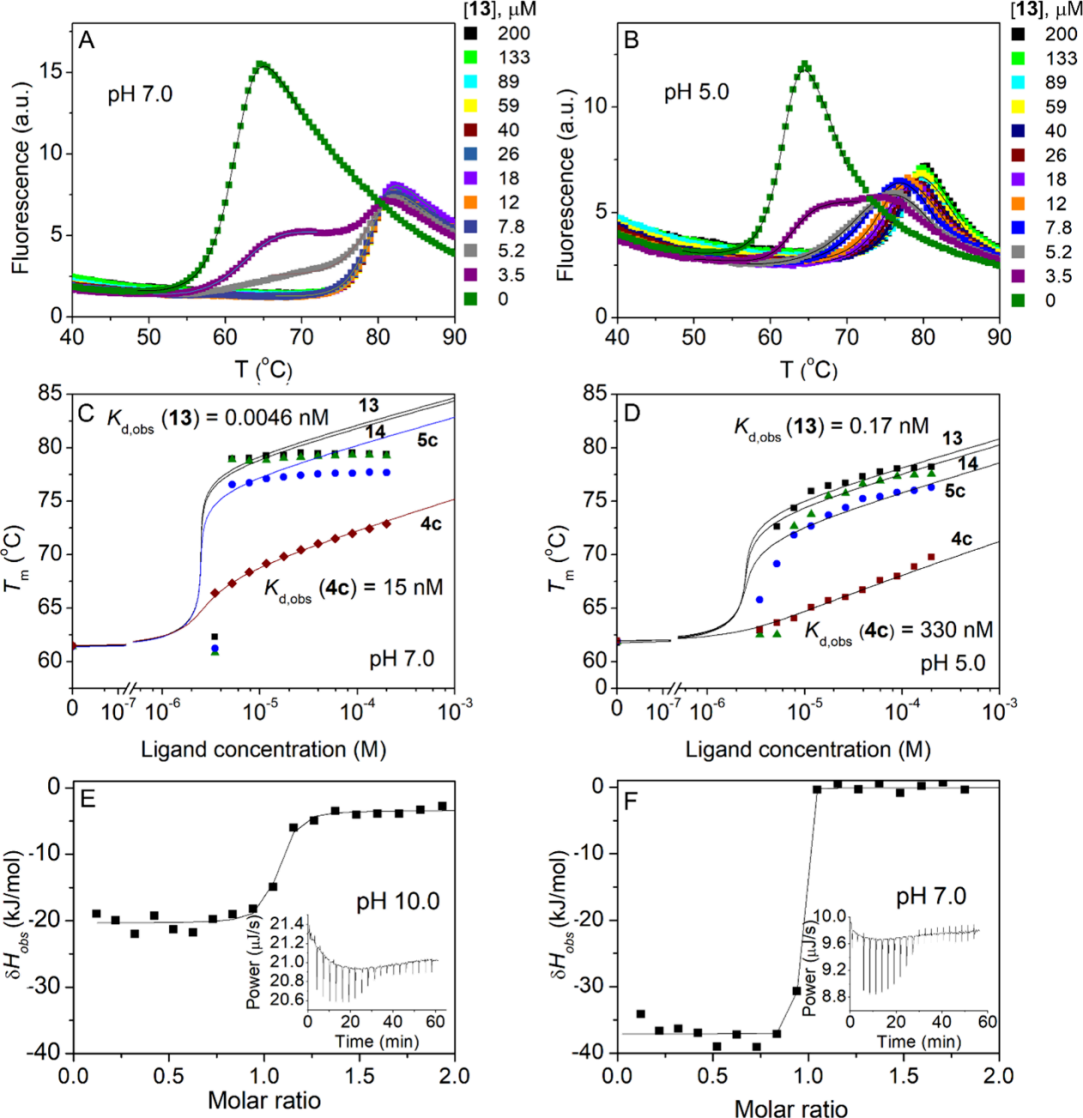
Compound binding to CAIX at several pH values (pH 5.0, pH 7.0 and pH 10.0). (A) FTSA data showing CAIX thermal melting curve shift with increasing compound **13** concentration at pH 7.0. (B) FTSA data showing CAIX thermal melting curve shift with increasing compound **13** concentration at pH 5.0. (C) Dependence of the CA IX *T*_*m*_ values on the concentrations of added compounds **4c**, **5c**, **13,** and **14** at pH 7.0 (fitted according to ref 27). (D) Dependence of the CA IX *T*_*m*_ values on the concentrations of added compounds **4c**, **5c**, **13,** and **14** at pH 5.0 (fitted according to ref 27). (E) ITC data of compound **13** binding to CAIX at pH 10.0. (F) ITC data of compound **13** binding to CAIX at pH 7.0.

It is known that sulfonamide binding affinity to CA isozymes follows a U-shape pH dependence,^21^ leading to a decrease in binding constants at low and high pHs. To confirm that the *K*_d,obs_ values are accurately determined at pH 7.0 we determined *K*_d,obs_ values for five sulfonamides **4c**, **5c**, **6**, **13** and **14** at lower pH, namely, pH 5.0, and calculated the intrinsic *K*_d,int_ values from the *K*_d,obs_ determined at pH 7.0 and pH 5.0 (Figure 1A, B, C, D and Figure S24). As seen in Table S2 the calculated *K*_d,int_ values differ by no more than 1.8-fold which is within our *K*_d_ determination error margin. This confirms that the value of *K*_d,obs_ is determined reliably by fitting the dosing curve of the high-affinity ligand (when no increase in *T*_m_ by increasing ligand concentration is observable). The tight binding of **13** to CAIX was confirmed by the ITC experiment, the slope of the integrated binding curve at pH 7.0 was extremely steep not allowing accurate determination of *K*_d_, however at pH 10 we obtained quite reasonable ITC binding isotherm with *K*_d,obs_ around 26 nM (Figure 1E, F and Figure S25).

The elongation of the 5-substituent from methoxy to ethoxy decreased binding to CAVB by 12.8-fold and to CAXIII by 6.8-fold, whereas other anhydrases showed up to 2-fold change in affinity. Interestingly, cancer-related CAXII also interacts strongly with **13** and **14**, exhibiting 0.05 nM and 0.11 nM binding affinities, respectively. Both compounds do not interact with CAVA and bind weakly to CAI and CAIII (*K*_d,obs_ 1.4 μM and about 70 μM), whereas the affinity for other CA isozymes is in the nanomolar range (*K*_d,obs_ from 0.25 to 71 nM).

The nature of the *para* substituent does not significantly affect the strength of interactions with CA isozymes. Comparing compounds **16**, **20** and **24**, which differ only in the functional group of the *para* tail, with acetamide, hydroxy, or *tert*-butyl carbamate groups, respectively, all compounds bind to CA with the same affinity (*K*_d,obs_ do not differ by more than 2-fold). The exception is compound **16**, which does not bind CAI and CAVII (*K*_d,obs_ ≥ 200 μM), whereas compounds **20** and **24** affinities for CAI are low micromolar (*K*_d,obs_ 2.5 and 8.3 μM, respectively). The gradual increase of the size of the cyclic substituent from cyclopropyl **17**, cyclopentyl **16**, to cyclooctyl **15** groups resulted in the decrease of binding strength for all CAs. The *K*_d_s for CAIX increases in the direction **17** → **16** → **15** from 0.09 nM → 0.29 nM → 10 nM. Compound **15** is very selective for CAIX, binding only CAVI and CAXII with 2.5 μM, and CAXIII with 5 μM *K*_d_.

We have also synthesized compounds **26–29** that have a short methylsulfonyl group at the *para*-position. The compounds differ in the substituents at the 3- and 5-positions. The weakest binding to CAIX (*K*_d,obs_ = 56 nM) was observed with compound **26** bearing hydroxycyclohexyl moieties at both *meta* positions. The hydroxycyclohexyl group in the hydrophobic pocket of the active site is probably unfavorable for interaction. Compound **29** was the most selective for CAIX (*K*_d,obs_ = 0.2 nM), showed 250-fold lower affinity to CAXII and more than 2000-fold lower affinity to all other CA isozymes.

In summary, all di-*meta* substituted compounds **6**-**29** display the highest affinity for CAIX, with *K*_d,obs_s ranging from nanomolar to picomolar (from 56 nM for **26** to 4.5 pM for **13** and **14**). Compounds **7**, **9**, **24** and **29** (*K*_d,obs_s for CAIX in the range of 0.012 – 0.25 nM) showed more than 1000-fold selectivity toward CAIX at the same time binding to CAXII with high affinity (*K*_d_s in the range of 0.24 – 90 nM).

Two compounds with the highest affinity for CAIX, namely **13** and **14**, were competitively dosed in a mixture with fluorescein-labeled compound GZ19–32 (10 nM) to determine their affinities for the HeLa cell-expressed CAIX as described in ref 28 (Figure 2). The dose–response competition curves showed that compound **13** bound to cell-expressed CAIX with a 2.5-fold increased affinity than compound **14**.

**Figure 2:**
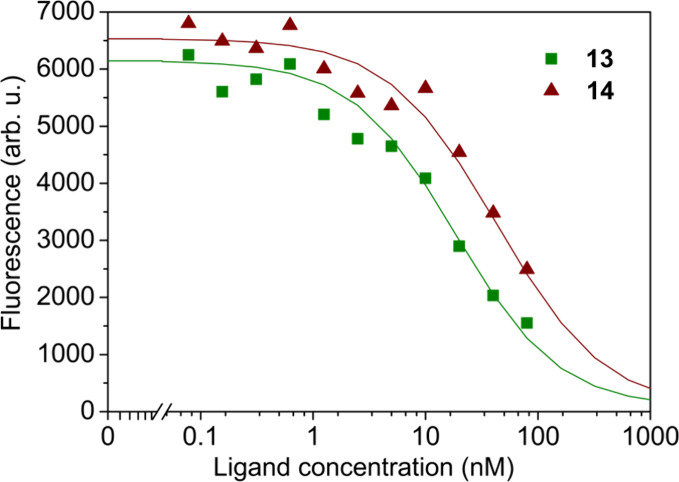
Determination of compound **13** (green squares) and **14** (red triangles) affinities for HeLa cell-expressed CAIX by competition assay with fluorescein-labeled GZ19–32 compound. The cells were grown under hypoxia. The application of the competition model yielded the *K*_d,obs_ value for compound **13** equal to 0.4 nM, for **14** - 1 nM. Both curves were fit using the total CAIX concentration of 5 nM and the *K*_d,obs_ of GZ19–32 of 200 pM.

### The Crystal Structures of CAIX and CAXII in Complexes with Compounds

In this study, we report eight X-ray crystal structures of protein–ligand complexes. Data collection and refinement statistics are summarized in Tables S3 and S4, while the images of the electron densities of the ligands in their soaked and/or cocrystallized structures are shown in Figure S26–S30. These structures include CAIX in complex with compound **26** (PDB ID: 9R30) and CAXII in complex with compound **9** (PDB ID: 9F3G). Additionally, we produced crystals of three ligands **10**, **13,** and **14** in complexes with CAXII by applying two protocols, cocrystallization, and soaking, to study if the binding of larger ligands induces protein conformation changes. Four ligands in complexes with CAXII are of similar structures, contain a cyclooctylamine group at the 3-position and (3-hydroxypropyl)­sulfonyl tail at the 4-position, but vary in substituents at the 5-position: compound **9** has a (4-hydroxybutyl)­amino moiety, compound **10** - piperidinyl, compound **13** - methoxy, and compound **14** - ethoxy (Figure 3). In contrast, compound **26** (in complex with CAIX, Figure 4) is structurally symmetrical, featuring 4-hydroxycyclohexylamino groups at both *meta* positions and methanesulfonyl group at the *para* position.

**Figure 3:**
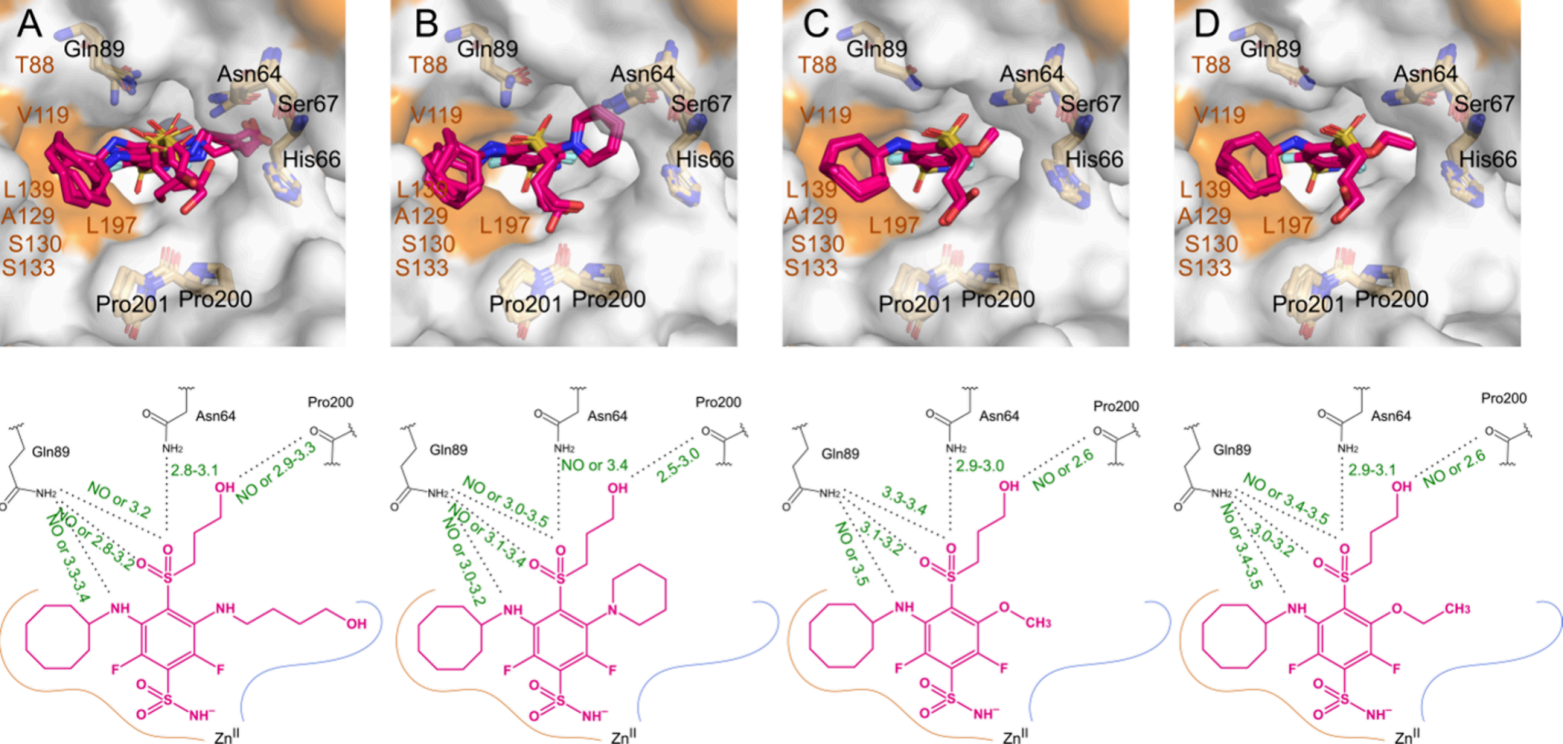
Binding modes of investigated compounds in the active site of CAXII. (A) Compound **9** (PDB ID: 9F3G). in all four subunits of CAXII within the asymmetric unit are displayed; (B) Six binding modes of compound **10** (PDB IDs: 9F2N and 9R0L) identified in two subunits of CAXII in 9F2N and four subunits in 9R0L. (C) Eight binding modes of the ligand **13** (PDB IDs: 9F2O and 9R31) in complex structures each containing four subunits. (D) Structures of CAXII-**14** (PDB IDs: 9F30 and 9R0U) each containing four subunits, presenting eight binding modes of **14**. The surface of hydrophobic amino acids is highlighted in orange. Below the crystal structures, the chemical structures of the compounds are shown, along with summarized distances of possible hydrogen bonds. Distances greater than 3.5 Å are labeled as “NO”, indicating that hydrogen bonding is unlikely. For simplicity, coordination and hydrogen bonds between the sulfonamide group, zinc, and threonine, which are characteristic of this pharmacophoric group, are not depicted.

**Figure 4:**
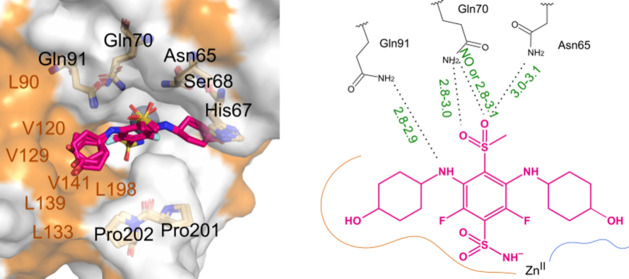
Binding modes of **26** in the active site of CAIX (PDB ID: 9R30). Ligands from all four subunits within the asymmetric unit are displayed. The surface of hydrophobic amino acids is highlighted in orange. Next to the crystal structure, the chemical structure of the compound is displayed, summarizing the distances of possible hydrogen bonds. Distances greater than 3.5 Å are labeled as “NO”, indicating that hydrogen bonding is unlikely. For simplicity, coordination and hydrogen bonds between the sulfonamide group, zinc, and threonine, characteristic of this pharmacophoric group, are not shown.

Each crystal structure contains four protein chains in the asymmetric unit, except for PDB ID: 9F2N, which contains two chains. This allows for comparative analysis of ligand binding modes across multiple subunits and independently determined structures. The variability in binding modes can be attributed to (i) the flexibility of certain ligand substituents; (ii) lower resolution and weaker electron density, making accurate modeling challenging. However, analyzing multiple subunits and independent crystal structures provides a more detailed understanding of protein–ligand interactions and structural variations.

A complex of CAXII-**10** is present in structures containing two and four subunits. In all subunits, the ligand adopts a consistent binding mode, with variability in the cyclooctylamino and piperidinyl rings. Additionally, the *para*-tail hydroxyl group shows positional variability, as it is exposed to solvent. Interestingly, greater conformational variability is observed in the four-subunit structure (PDB ID: 9R0L). The largest differences occur between subunits A and D (RMSD ∼ 1 Å, the overlay was based on H91, H93, H117 and Zn), primarily due to cyclooctylamino ring flexibility.

Complexes of CAXII-**13** and CAXII-**14** each have four subunits in both duplicate structures, and the ligands adopt nearly identical conformations across all eight subunits. Even the cyclooctylamino rings show high consistency, with minor *para*-tail flexibility in the C subunits (PDB IDs: 9F2O and 9F30).

For CAXII-**9**, a single conformation was modeled, but its exact positioning could not be determined with high confidence. In chain A, the benzenesulfonamide rotates ∼ 17° at the sulfonamide sulfur, causing positional variations across chains. However, ligand conformations in chains B, C, and D are more consistent, as reflected by RMSD values.

Meanwhile, compound **26** in complex with CAIX occupies similar positions in all four subunits. Larger variability is observed in the *meta* substituent, which interacts with the hydrophobic active site. Differences also exist in the *para*-substituent orientation.

Due to the similarity of chemical structures, the complexes of all ligands with CAXII can be generalized in some aspects (Figure 3). First, the *meta* cyclooctylamino group is positioned in a hydrophobic region of the active site, making the main contacts with A129-S130 (main chain), V119, S133, and side chains of L197 and L139.

The sulfoxide linker of the *para*-tail is stabilized by a hydrogen bond with N64 and Q89, except in CAXII-**10**, where N64 is too distant for interaction, but in one subunit 3.4 Å hydrogen bond is still possible. Q89 can form three potential hydrogen bonds with the sulfoxide oxygen atoms and nitrogen linker of the cyclooctylamine. Assuming a hydrogen bond distance of less than or equal to 3.5 Å, all possible contacts are represented in 2D schemes (Figure 3). The hydroxyl tail is oriented against the backbone of P200–P201, forming a hydrogen bond with the main chain oxygen of P200 in most subunits. It should be noted that not all subunits have a well-defined tail density for all atoms, especially the terminal oxygen atom, making it difficult to model this substitution objectively. However, it is clear that despite the tail’s lability, a hydroxy group of this length is most likely to form a hydrogen bond with P200.

Variations in interactions arise from the opposite and different *meta-*substituents. However, all these substituents are positioned in a hydrophilic region of the cavity formed by residues W4, Y6, N64, H66–S67, H91, and T200. In the structure containing compound **13** with a *meta*-methoxy group, a water molecule is consistently found between the ligand and protein, forming hydrogen bonds with Y6 and H67, but without a water-mediated hydrogen bond. The *meta*-ethoxy group in compound **14** is oriented similarly within the hydrophilic region of the active site, but unlike compound **13**, there is no space for a water molecule between the ligand and protein side chains. The *meta*-piperidinyl group in compound **10** is well-stabilized and tightly packed against the hydrophilic cavity wall, with all conformations being similar. Like compound **13**, a water molecule is also found in a similar position and forms hydrogen bonds with Y6 and H67. However, there is no direct interaction between this *meta-*substituent of the ligand and the protein.

In the CAXII-**9** complex, hydrogen bonds are possible between the hydroxyl group and the main chains oxygens of H66 and L92. However, a hydrogen bond with Y6 is unlikely due to unfavorable geometry. The ligand occupies most of the binding cavity, leaving minimal space for solvent molecules.

The structure of CAIX has important differences from CAXII, but certain aspects of the interaction with this type of compound remain the same (Figure 4). The hydrophobic side of the active site of CAIX is slightly larger due to a shifted α helix compared to CAXII and is more hydrophobic, consisting of L90, V120, V129, L133, L139, V141, and L198 amino acids. Q91 forms a hydrogen bond with the nitrogen linker from the cyclooctylamino substituent, while Q70 can form two possible hydrogen bonds with the sulfonyl oxygen atoms. Similar to CAXII, Asn65 also forms a hydrogen bond with the sulfonyl oxygen atom. The most surprising feature of the CAIX-**26** structure is that the substituents on the hydrophilic side push a fragment of the protein molecule. The hydrophilic side of the active site should be closed and consist of N-terminal amino acids (WRYGGDPPWPRV). The electron density for this fragment is absent in this crystal structure. The protein–ligand interaction is likely stronger than the bonds holding this 12-amino acid fragment in place. Due to the lost bonds, the fragment becomes flexible, leading to a lack of clearly identifiable electron density. This shift of the protein fragment occurs because, compared to other crystal structures found in the PDB, the active site of the protein is too small to accommodate a ligand of this size and an obvious steric clash is observed.

There are examples in the literature showing that the crystal structures obtained from soaked and cocrystallized protein–ligand complexes differ quite significantly.^29,30^ Larger and flexible ligands can cause conformational changes in the protein structure, therefore the binding poses of ligands in cocrystallized and soaked structures can deviate. The selection of crystal structures can be important in rational drug design and optimization, as the outcome may be different depending on which structure is used for optimization.

Our previous studies showed that carbonic anhydrases are rather rigid proteins and do not undergo significant conformational changes upon ligand binding.^31^ Interestingly, despite the bulkiness of synthesized di-*meta*-substituted compounds **10**, **13,** and **14**, especially the first one compound **10**, which possesses large piperidinyl and cyclooctylamino groups, ligands acquire quite similar binding poses in the soaked and cocrystallized complexes with CAXII. Alternatively, the bulky compound **26** with 4-hydroxycyclohexylamino substituents at both *met*a positions seems to push aside the N-terminal amino acid fragment when it binds to CAIX, allowing it to fit into the active site pocket. As we did not obtain the crystal structure of this compound with CAIX by soaking, it is not clear whether such amino acid perturbations would be observed using this crystallization method. Presumably, for bulky compounds, cocrystallization will more effectively capture the conformational rearrangements induced in the protein during ligand binding.

## Conclusions

In this study, we performed a structure-affinity relationship analysis and optimization of the previously discovered hit compound VD11–4–2 (compound **5b**) by systematic structural modification at the 5-position of benzenesulfonamide with functional groups of various lengths and bulkiness. We synthesized a library of di-*meta*-substituted compounds with higher selectivity for cancer-related CAIX and CAXII isozymes than the parent *meta*-substituted compound VD11–4–2 (compound **5b**). Two compounds **13** and **14** bearing methoxy and ethoxy substitutions bind to CAIX with exceptionally low picomolar affinity (*K*_d,obs_ = 4.5 pM) and also exhibit strong binding to the CAXII isozyme. The crystal structure studies of CAIX and CAXII complexes with compounds containing 4-hydroxycyclohexylamino (compound **26**), 4-hydroxybutylamino (compound **9**), piperidinyl (compound **10**), methoxy (compound **13**), and ethoxy (compound **14**) moieties at the 5-position revealed that these substituents fit in the hydrophilic region of the active sites. In contrast, the bulky substituents at the 3-position fill a hydrophobic pocket in both proteins. Our findings demonstrated the therapeutic potential of di-*meta*-substituted compounds, efficient binders of cancer-related CAIX and CAXII isozymes, for cancer treatment.

## Experimental Section

### General Procedures

All solvents and chemicals were commercially available or prepared according to known procedures. Melting points of the compounds were determined in open capillaries on a Thermo Scientific 9100 Series and are uncorrected. ^1^H and ^13^C NMR spectra were recorded on a Bruker spectrometer (400 and 100 MHz, respectively) in DMSO-*d*_6_ or CDCl_3_ using residual DMSO, CDCl_3_ signals (2.50, 7.26, and 39.52 ppm, 77.16 ppm for ^1^H and ^13^C NMR spectra, respectively) as the internal standard. ^19^F NMR spectra were recorded on a Bruker spectrometer (376 MHz) with CFCl_3_ as an internal standard. TLC was performed with silica gel 60 F254 aluminum plates (Merck) and visualized with UV light. Column chromatography was performed using silica gel 60 (0.040–0.063 mm, Merck). The purity of final compounds was verified by UPLC-MS assay (column: Waters Acquity UPLC BEH-C_18_, 2.1 mm × 50 mm, 1.7 μm, column temperature 30.0 ± 5.0 °C; gradient: CH_3_CN/0.01% aq. TFA from 10 to 95%; flow rate: 0.5 mL/min; run time: 8 min; detector: photodiode array in 220–320 nm range, MS detector: Waters SQ detector with an electrospray ion source) and is ≥ 95%. High-resolution mass spectra (HRMS) were recorded on a Dual-ESI Q-TOF 6520 mass spectrometer (Agilent Technologies). Elemental analyses were conducted on Carlo Erba EA-1108 apparatus. Melting points are uncorrected. Compound IUPAC names were generated with ChemDraw ultra 12.0.

#### Pentafluorobenzenesulfonamide (2)

Pentafluorobenzenesulfonyl chloride (2 mL, 13.5 mmol, 1 equiv) was dissolved in THF (70 mL) at −10 °C temperature and intensely stirred while adding 100 μL of NH_3_ (25%) aq every several minutes as well as maintaining −10 °C. After reaction completion mixture was stirred for an additional 30 min and the solvent was evaporated under reduced pressure. The crude product was purified by crystallization from H_2_O. Yield: 2.3 g (69%), as white solid, Mp: 154 – 155 °C (close to the determined temperature in literature – Mp: 155 – 156 °C^32^).

#### *N*-(2-Mercaptoethyl)­acetamide

*N*-(2-Mercaptoethyl)­acetamide was prepared according to known procedure in literature.^33^ Cysteine hydrochloride (6.0 g, 52.8 mmol, 1.02 equiv) was dissolved in H_2_O (25 mL) and NaOH (2.1 g, 52.8 mmol, 1.02 equiv) was added in portions. After complete NaOH dissolution, NaHCO_3_ (5.3 g, 63.2 mmol, 1.22 equiv) was added and by intensely mixing solution over 30 min period (Ac)_2_O (4.9 mL, 51.8 mmol, 1 equiv) was added dropwise. Afterward, the mixture is stirred for an additional 20 min, washed with brine (10 mL) and extracted with EtOAc (5 × 10 mL). The organic phase was dried over anhydrous Na_2_SO_4_, filtered and the organic solvent was removed under reduced pressure. Yield: 4.97 g (79%), as slightly purple oil. ^**1**^ **H NMR** (400 MHz, CDCl_3_) δ: 1.75 (1H, s, HSCH_2_), 2.02 (3H, s, CH_3_CO), 2.87 (2H, t, *J* = 6.4 Hz, HSCH_2_), 3.58 (2H, q, *J* = 6.3 Hz, NHCH_2_), 6.43 (1H, s, NHCH_2_).

#### 2,3,5,6-Tetrafluoro-4-(2-(acetamido)­ethylthio)­benzenesulfonamide (3a)

2,3,5,6-Tetrafluoro-4-(2-(acetamido)­ethylthio)­benzenesulfonamide was prepared according to a procedure known in the literature.^33^ A mixture of compound **2** (2.3 g, 9.4 mmol, 1 equiv), *N*-(2-mercaptoethyl)­acetamide (1.3 mL, 13.1 mmol, 1.4 equiv) and Et_3_N (2.0 mL, 14.1 mmol, 1.5 equiv) in MeOH (25 mL) was stirred at room temperature. After 2 h additional *N*-(2-mercaptoethyl)­acetamide (150 μL, 1.5 mmol, 0.16 equiv) and Et_3_N (150 μL, 1.1 mmol, 0.1 equiv) portions were added. An hour later the solvent was removed under reduced pressure. The crude product was purified by crystallization from H_2_O/MeOH (1:6) mixture. Yield: 2.6 g (78%), as white solid, Mp: 169 – 170 °C (close to the determined temperature in literature – Mp: 169 – 171 °C^33^). ^**1**^ **H NMR** (400 MHz, DMSO-*d*_6_) δ: 1.72 (3H, s, CH_3_CO), 3.13 (2H, t, *J* = 6.2 Hz, SCH_2_), 3.24 (2H, q, *J* = 6.0 Hz, CH_2_NH), 8.00 (1H, t, *J* = 4.9 Hz, NHCH_2_), 8.42 (s, 2H, SO_2_NH_2_). ^**13**^ **C NMR** (100 MHz, DMSO-*d*_6_) δ: 22.74 (CH_3_CO), 33.42 (NHCH_2_, t, *J* = 3.0 Hz), 39.43 (SCH_2_), 118.67 (C1, t, *J*(^19^F – ^13^C) = 20.1 Hz), 122.85 (C4, t, *J*(^19^F – ^13^C) = 15.5 Hz), 142.93 (C2 and C6, dd ^1^ *J*(^19^F – ^13^C) = 253.3 Hz, ^2^ *J*(^19^F – ^13^C) = 16.8 Hz), 146.92 (C3 and C5, dd, ^1^ *J*(^19^F – ^13^C) = 240.3 Hz, ^2^ *J*(^19^F – ^13^C) = 18.9 Hz), 169.81 (CO). ^**19**^ **F NMR** (376 MHz, DMSO-*d*_6_) δ: −139.37 (2F, ddd, ^1^ *J* = 24.0 Hz, ^2^ *J =* 13.9 Hz, ^3^ *J* = 4.5 Hz), −132.84 (2F, ddd, ^1^ *J* = 24.0 Hz, ^2^ *J =* 13.9 Hz, ^3^ *J* = 4.5 Hz). HRMS for C_10_H_10_F_4_N_2_O_3_S_2_ [(M+H)^+^]: calc. 347.0142, found 347.0135.

#### 2,3,5,6-Tetrafluoro-4-((2-hydroxyethyl)­thio)­benzenesulfonamide (3b)

2,3,5,6-Tetrafluoro-4-((2-hydroxyethyl)­thio)­benzensulfonamide was prepared according to the known procedure in literature.^32^ A mixture of compound **2** (1.0 g, 4.1 mmol, 1 equiv), 2-mercaptoethanol (340 μL, 4.9 mmol, 1.2 equiv) and Et_3_N (680 μL, 4.9 mmol, 1.2 equiv) in MeOH (20 mL) was stirred at room temperature overnight. Next morning additional 2-mercaptoethanol (85 μL, 1.21 mmol, 0.3 equiv) and Et_3_N (170 μL, 1.21 mmol, 0.3 equiv) portions were added and the reaction mixture was stirred for an additional 2 h. After reaction completion, the solvent was evaporated under reduced pressure and the resultant precipitate was washed with H_2_O. The crude product was purified by crystallization from H_2_O. Yield: 1.03 g (83%), as white solid, Mp: 111 – 112 °C (close to the determined temperature in literature – Mp: 111 – 112 °C).

#### 2,3,5,6-Tetrafluoro-4-((3-hydroxypropyl)­thio)­benzenesulfonamide (3c)

A mixture of compound **2** (1.0 g, 4.05 mmol, 1 equiv), 3-mercaptopropan-1-ol (0.41 mL, 4.5 mmol, 1.1 equiv) and Et_3_N (0.57 mL, 4.09 mmol, 1.01 equiv) in MeOH (30 mL), was refluxed for 2 h. After reaction completion, the solvent was evaporated under reduced pressure and the resultant precipitate was washed with H_2_O. The crude product was purified by crystallization from EtOH. Yield: 1.1 g (85%), mp 136 – 138 °C. ^**1**^ **H NMR** (400 MHz, DMSO-*d*_6_): 1.55–1.74 (2H, m, SCH_2_CH_2_CH_2_), 3.02–3.16 (2H, m, SCH_2_CH_2_CH_2_), 3.40–3.53 (2H, m, SCH_2_CH_2_CH_2_), 4.28–4.80 (1H, br s, OH), 8.03–8.65 (2H, br s, SO_2_NH_2_). ^**13**^ **C NMR** (400 MHz, DMSO-*d*_6_): 30.78 (SCH_2_ CH_2_CH_2_), 32.69 (SCH_2_), 58.70 (SCH_2_CH_2_ CH_2_), 118.57 (C4, t, *J*(^19^F – ^13^C) = 20.5 Hz), 122.46 (C1, t, *J*(^19^F – ^13^C) = 15.9 Hz), 141.05 – 144.12 (C3 and C5, m), 145.03 – 147.97 (C2 and C6, m). ^**19**^ **F NMR** (400 MHz, DMSO-*d*_6_): −133.25 to −133.46 (2F, m), −139.06 to −139.27 (2F, m). Anal. Calcd for C_9_H_9_F_4_NO_3_S_2_: C, 33.86; H, 2.84; N, 4.39. Found: C, 34.15; H, 2.93; N, 4.32. HRMS (*m*/*z*): [M-H]^−^ calc. for C_9_H_9_F_4_NO_3_S_2_, 317.9882, found 317.9893.

#### 2,3,5,6-Tetrafluoro-4-(2-(acetamido)­ethylsulfonyl)­benzenesulfonamide (4a)

2,3,5,6-Tetrafluoro-4-(2-(acetamido)­ethylsulfonyl)­benzenesulfonamide was prepared according to known procedure in literature.^34^ Compound **3a** (2.6 g, 7.5 mmol) was dissolved in AcOH (70 mL) and heated at 75 °C temperature for 10 h. H_2_O_2_ (40%) was added in portions (100 μL) every 15 min (overall 4.2 mL) until complete starting material conversion. Afterward, the solvent was evaporated under reduced pressure and the resultant precipitate was washed with H_2_O. The crude product was purified by crystallization from H_2_O/MeOH (1:6) mixture. Yield: 1.58 g (56%), as white solid, Mp: 224 – 225 °C (close to the determined temperature in literature – Mp: 224 – 225 °C^34^). ^**1**^ **H NMR** (400 MHz, DMSO-*d*_6_) δ: 1.64 (3H, s, CH_3_CO), 3.48 (2H, q, *J* = 5.8 Hz, CH_2_NH), 3.78 (2H, t, *J* = 6.1 Hz, SO_2_CH_2_), 8.07 (1H, t, *J* = 4.8 Hz, CH_2_NH), 8.68 (s, 2H, SO_2_NH_2_). ^**13**^ **C NMR** (100 MHz, DMSO-*d*_6_) δ: 22.54 (CH_3_CO), 33.34 (NHCH_2_), 56.14 (SO_2_CH_2_), 121.42 (C1, t, *J*(^19^F – ^13^C) = 14.8 Hz), 128.17 (C4, t, *J*(^19^F – ^13^C) = 15.4 Hz), 142.10 (C2 and C6, dd, ^1^ *J*(^19^F – ^13^C) = 254.1 Hz, ^2^ *J*(^19^F – ^13^C) = 16.1 Hz), 143.5 (C3 and C5, dd, ^1^ *J*(^19^F – ^13^C) = 256.1 Hz, ^2^ *J*(^19^F – ^13^C) = 16.2 Hz), 170.15 (CO). ^**19**^ **F NMR** (376 MHz, DMSO-*d*_6_) δ: −135.9 (2F, ddd, ^1^ *J* = 26.3 Hz, ^2^ *J* = 15.0 Hz, ^3^ *J* = 7.5 Hz), −136.6 (2F, ddd, ^1^ *J* = 26.3 Hz, ^2^ *J* = 11.3 Hz, ^3^ *J* = 7.5 Hz). HRMS for C_10_H_10_F_4_N_2_O_5_S_2_ [(M+H)^+^]: calc. 379.0040, found 379.0038.

#### 2,3,5,6-Tetrafluoro-4-((2-hydroxyethyl)­sulfonyl)­benzenesulfonamide (4b)

2,3,5,6-Tetrafluoro-4-((2-hydroxyethyl)­sulfonyl)­benzenesulfonamide was prepared according to known procedure in literature.^32^ Compound **3b** (1.54 g, 5.06 mmol, 1 equiv) was dissolved in acetic acid (30 mL) and heated at 75 °C temperature for 18 h. H_2_O_2_ (30%) was added in portions (100 μL) every 30 min (overall 3.6 mL) until complete starting material conversion. Afterward, the solvent was evaporated under reduced pressure and the product was purified by column chromatography (silica, EtOAc/CHCl_3_ (1:1), *R*_F_ = 0.13). Yield: 768 mg (45%), as white solid, Mp: 138 – 139 °C (close to the determined temperature in literature – Mp: 139 – 140 °C^32^).

#### 2,3,5,6-Tetrafluoro-4-((3-hydroxypropyl)­sulfonyl)­benzenesulfonamide (4c)

Compound **3c** (3.0 g, 9.7 mmol, 1 equiv) was placed in a 200 mL pressure tube with a magnet, followed by AcOH (30 mL), H_2_O_2_ (15 mL, 35%) and water (15 mL). The tube was sealed and stirred at 70 °C for 18 h. Upon cooling to room temperature reaction mixture was concentrated *in vacuo*, and the crude product was purified by crystallization from EtOH/water (2:1). Yield: 3.1 g (94%), as white solid, Mp: 180 – 182 °C. ^**1**^ **H NMR** (400 MHz, DMSO-*d*_6_) δ: 1.80 – 1.90 (2H, m, SO_2_CH_2_CH_2_CH_2_), 3.47 (2H, t, *J* = 6.1 Hz, SO_2_CH_2_CH_2_CH_2_), 3.56 – 3.63 (2H, m, SO_2_CH_2_CH_2_CH_2_), 4.02 (1H, br s, OH overlapped with water), 8.62 (2H, s, SO_2_NH_2_). ^**13**^ **C NMR** (101 MHz, DMSO-*d*_6_) δ: 25.43 (SO_2_CH_2_ CH_2_CH_2_), 54.51 (SO_2_ CH_2_CH_2_CH_2_), 58.81 (SO_2_CH_2_CH_2_ CH_2_), 120.96 (C4, t, *J*(^19^F – ^13^C) = 15.1 Hz), 128.02 (C1, t, *J*(^19^F – ^13^C) = 15.3 Hz), 143.43 (C2 and C6, dd, ^1^ *J*(^19^F – ^13^C) = 257.7, ^2^ *J*(^19^F – ^13^C) = 14.2 Hz), 144.80 (C3 and C5, dd, ^1^ *J*(^19^F – ^13^C) = 257.3, ^2^ *J*(^19^F – ^13^C) = 12.4 Hz). ^**19**^ **F NMR** (376 MHz, DMSO-*d*_6_) δ: −135.93 – −136.15 (2F, m), −136.45 – −136.66 (2F, m). Anal. Calcd for C_9_H_9_F_4_NO_5_S_2_: C, 30.77; H, 2.58; N, 3.99. Found: C, 31.01; H, 2.75; N, 3.91. HRMS for C_9_H_9_F_4_NO_5_S_2_ [(M-H)^−^]: calc. 349.9780, found 349.9789.

#### 3-(Cyclooctylamino)-2,5,6-trifluoro-4-((2-acetamido)­ethylsulfonyl)­benzenesulfonamide (5a)

3-(Cyclooctylamino)-2,5,6-trifluoro-4-((2-acetamido)­ethylsulfonyl)­benzenesulfonamide was prepared according to known procedure in literature.^34^ A mixture of compound **4a** (1.39 g, 3.67 mmol, 1 equiv) and cyclooctylamine (446 μL, 3.26 mmol, 2 equiv) in DMSO (2 mL) was stirred at room temperature overnight. After full starting material conversion reaction mixture was washed with brine (10 mL) and extracted with EtOAc (3 × 15 mL). The organic phase was dried over anhydrous Na_2_SO_4_, filtered and the organic solvent was removed under reduced pressure. The product was purified by column chromatography (silica, EtOAc/CHCl_3_ (1:1), *R*_F_ = 0.53). Yield: 0.90 g (50%), as white solid, Mp: 161 – 162 °C (close to the determined temperature in literature – Mp: 162 – 163 °C^34^).

#### 3-(Cyclooctylamino)-2,5,6-trifluoro-4-((2-hydroxyethyl)­sulfonyl)­benzenesulfonamide (5b)

3-(Cyclooctylamino)-2,5,6-trifluoro-4-((2-hydroxyethyl)­sulfonyl)­benzenesulfonamide was prepared according to known procedure in literature.^35^ A mixture of compound **4b** (550 mg, 1.63 mmol, 1 equiv) and cyclooctylamine (446 μL, 3.26 mmol, 2 equiv) in DMSO (2 mL) was stirred at room temperature overnight. After full starting material conversion reaction mixture was washed with brine (10 mL) and extracted with EtOAc (3 × 15 mL). The organic phase was dried over anhydrous Na_2_SO_4,_ filtered and the organic solvent was removed under reduced pressure. The product was purified by column chromatography (silica, EtOAc/CHCl_3_ (1:1), *R*_F_ = 0.52). Yield: 380 mg (52%), as white solid, Mp: 90 – 91 °C (close to the determined temperature in literature – Mp: 89 – 90 °C^35^).

#### 3-(Cyclooctylamino)-2,5,6-trifluoro-4-((3-hydroxypropyl)­sulfonyl)­benzenesulfonamide (5c)

A mixture of compound **4c** (3.1 g, 8.82 mmol, 1 equiv) and cyclooctylamine (2.2 g, 17.7 mmol, 2 equiv) in anhydrous DMSO (50 mL) was stirred at room temperature. After 1 h water (50 mL) was added and the mixture was extracted with EtOAc (2 × 50 mL). Combined organic extracts were washed with brine (3 × 50 mL), dried over MgSO_4_, and filtered and the organic solvent was removed under reduced pressure. The crude product was dried *in vacuo* for 16 h at room temperature and purified by flash chromatography (silica, EtOAc/Hexane (1:1)). Yield: 2.5 g (63%), as white solid, mp 150 – 152 °C. ^**1**^ **H NMR** (400 MHz, DMSO-*d*_6_) δ: 1.40 – 1.70 (12H, m, cyclooctyl), 1.74 – 1.90 (4H, m, cyclooctyl and SO_2_CH_2_CH_2_CH_2_), 3.41 – 3.50 (2H, m, SO_2_CH_2_CH_2_CH_2_), 3.50 – 3.59 (2H, m, SO_2_CH_2_CH_2_CH_2_), 3.73 – 3.85 (1H, m, cyclooctyl NHCH), 4.70 (1H, t, *J* = 5.3 Hz, OH), 6.63 – 6.74 (1H, m, cyclooctyl NHCH), 8.34 (2H, s, SO_2_NH_2_). ^**13**^ **C NMR** (101 MHz, DMSO-*d*_6_) δ: 22.78 (cyclooctyl), 24.98 (SO_2_CH_2_ CH_2_CH_2_), 25.43 (cyclooctyl), 26.76 (cyclooctyl), 32.15 (cyclooctyl), 53.88 (SO_2_ CH_2_CH_2_CH_2_), 55.35 (cyclooctyl NHCH), 58.34 (SO_2_CH_2_CH_2_ CH_2_), 114.51 (C4, dd,^1^ *J*(^19^F – ^13^C) = 12.7 Hz,^2^ *J*(^19^F – ^13^C) = 5.3 Hz), 127.49 (C1, dd,^1^ *J*(^19^F – ^13^C) 18.5 Hz, ^2^ *J*(^19^F – ^13^C) = 14.2 Hz), 134.85 (C3, dd,^1^ *J*(^19^F – ^13^C) = 13.8 Hz, ^2^ *J*(^19^F – ^13^C) = 1.8 Hz), 138.01 (C6, dd,^1^ *J*(^19^F – ^13^C) = 12.2 Hz,^2^ *J*(^19^F – ^13^C) = 4.7 Hz), 144.06 (C2, d, *J* = 253.9 Hz), 145.56 (C5, dd,^1^ *J*(^19^F – ^13^C) = 251.1 Hz, ^2^ *J*(^19^F – ^13^C) = 15.1 Hz). ^**19**^ **F NMR** (376 MHz, DMSO-*d*_6_) δ: −124.77 (C2–F, s), −134.36 (C6–F, dd,^1^ *J* = 26.8 Hz,^2^ *J* = 12.6 Hz), −150.36 (C5–F, dd,^1^ *J* = 27.1 Hz,^2^ *J* = 6.7 Hz). Anal. Calcd for C_17_H_25_F_3_N_2_O_5_S_2_: C, 44.53; H, 5.50; N, 6.11. Found: C, 44.73; H, 5.63; N, 5.87. HRMS for C_17_H_25_F_3_N_2_O_5_S_2_ [(M+H)^+^]: calc. 459.1235, found 459.1234

#### 3-(Cyclooctylamino)-2,6-difluoro-4-((3-hydroxypropyl)­sulfonyl)-5-(methylamino)­benzenesulfonamide (6)

A mixture of compound **5c** (200 mg, 0.44 mmol, 1 equiv) and 2 M solution of MeNH_2_ in MeOH (0.65 mL, 1.32 mmol, 3 equiv) in anhydrous MeOH (2 mL) was stirred in a pressure vial at 70 °C. After 6 h the mixture was cooled to room temperature, poured into water (20 mL), and extracted with EtOAc (3 × 20 mL). Organic extracts were washed with brine (2 × 20 mL), dried over Na_2_SO_4_, filtered and concentrated under reduced pressure. The crude product was purified by flash chromatography (silica, EtOAc/Hexane/DCM (1:1:1)). Yield: 52 mg (25%), as yellow oil. ^**1**^ **H NMR** (400 MHz, CDCl_3_) δ: 1.44 – 1.77 (13H, m, cyclooctyl and OH), 1.81 – 1.93 (2H, m, cyclooctyl), 1.94 – 2.03 (2H, m, SO_2_CH_2_ CH_2_CH_2_), 3.03 (3H, d, *J* = 7.2 Hz, CH_3_), 3.33 – 3.41 (2H, m, SO_2_CH_2_CH_2_CH_2_), 3.71 – 3.78 (2H, t, *J* = 6.0 Hz, SO_2_CH_2_CH_2_CH_2_), 3.79 – 3.91 (1H, m, cyclooctyl CHNH), 5.46 (2H, SO_2_NH_2_), 6.06 – 6.56 (2H, br s, MeNH and cyclooctyl CHNH). ^**13**^ **C NMR** (101 MHz, CDCl_3_) δ: 23.64 (cyclooctyl), 25.44 (SO_2_CH_2_ CH_2_CH_2_), 25.69 (cyclooctyl), 27.28 (cyclooctyl), 33.28 (cyclooctyl), 34.19 (CH_3_, d, *J* = 13.4 Hz), 51.88 (SO_2_ CH_2_CH_2_CH_2_), 56.11 (cyclooctyl CHNH, d, *J* = 12.1 Hz), 60.38 (SO_2_CH_2_CH_2_ CH_2_), 111.03 (C4, t, *J*(^19^F – ^13^C) = 4.2 Hz), 126.29 (C1, t, *J*(^19^F – ^13^C) = 15.8 Hz), 135.29 (C3 or C5, dd,^1^ *J*(^19^F – ^13^C) = 12.7 Hz, ^2^ *J*(^19^F – ^13^C) = 2.7 Hz), 137.08 (C5 or C3, dd,^1^ *J*(^19^F – ^13^C) = 11.9 Hz, ^2^ *J*(^19^F – ^13^C) = 2.9 Hz), 139.57 (C2 and C6, dd,^1^ *J*(^19^F – ^13^C) = 244.6 Hz,^2^ *J*(^19^F – ^13^C) = 3.8 Hz). ^**19**^ **F NMR** (376 MHz, CDCl_3_) δ: −137.87 (1F, d, *J* = 10.0 Hz), −140.13 (1F, dt,^1^ *J* = 18.0 Hz,^2^ *J* = 7.2 Hz). HRMS for C_18_H_29_F_2_N_3_O_5_S_2_ [(M+H)^+^]: calc. 470.1595, found 470.1592.

#### 3-(Cyclooctylamino)-5-(dimethylamino)-2,6-difluoro-4-((3-hydroxypropyl)­sulfonyl)­benzenesulfonamide (7)

A mixture of compound **5c** (200 mg, 0.44 mmol, 1 equiv) and 2 M solution of Me_2_NH in MeOH (0.65 mL, 1.32 mmol, 3 equiv) in anhydrous MeOH (2 mL) was stirred in a pressure vial at 70 °C. After 6 h the mixture was cooled to room temperature, poured into water (20 mL), and extracted with EtOAc (3 × 20 mL). Organic extracts were washed with brine (2 × 20 mL), dried over Na_2_SO_4_, filtered and concentrated under reduced pressure. The crude product was purified by flash chromatography (silica, EtOAc/Hexane/DCM (1:1:1)). Yield: 47 mg (22%), as a yellow oil. ^**1**^ **H NMR** (400 MHz, CDCl_3_) δ: 1.43 – 1.75 (13H, m, cyclooctyl and OH), 1.81 – 1.91 (2H, m, cyclooctyl), 1.94 – 2.04 (2H, m, SO_2_CH_2_CH_2_CH_2_), 2.78 (6H, d, *J* = 1.6 Hz, N­(CH_3_)_2_), 3.63 – 3.72 (2H, m, SO_2_CH_2_CH_2_CH_2_), 3.73 – 3.84 (3H, m, cyclooctyl CHNH and SO_2_CH_2_CH_2_CH_2_), 5.44 (2H, s, SO_2_NH_2_), 7.21 (1H, d, *J* = 8.4 Hz, cyclooctyl CHNH). ^**13**^ **C NMR** (101 MHz, CDCl_3_) δ: 23.55 (cyclooctyl), 25.58 (SO_2_CH_2_ CH_2_CH_2_), 26.26 (cyclooctyl), 27.42 (cyclooctyl), 32.93 (cyclooctyl), 44.20 (N­(CH_3_)_2_, d, *J* = 4.7 Hz), 55.07 (SO_2_ CH_2_CH_2_CH_2_), 56.47 (cyclooctyl CHNH, d, *J* = 11.5 Hz), 60.63 (SO_2_CH_2_CH_2_ CH_2_), 125.38 (C1, t, *J*(^19^F – ^13^C) = 15.9 Hz), 125.91 (C4, dd,^1^ *J*(^19^F – ^13^C) = 6.4 Hz,^2^ *J*(^19^F – ^13^C) = 3.9 Hz), 137.23 (C3 or C5, dd,^1^ *J*(^19^F – ^13^C) = 13.0 Hz,^2^ *J*(^19^F – ^13^C) = 2.6 Hz), 137.88 (C5 or C3, dd,^1^ *J*(^19^F – ^13^C) = 15.0 Hz,^2^ *J*(^19^F – ^13^C) = 4.2 Hz), 147.52 (C2 or C6, dd,^1^ *J*(^19^F – ^13^C) = 257.6 Hz,^2^ *J*(^19^F – ^13^C) = 3.7 Hz), 148.93 (C6 or C2, dd,^1^ *J*(^19^F – ^13^C) = 252.5 Hz,^2^ *J*(^19^F – ^13^C) = 4.2 Hz). ^**19**^ **F NMR** (376 MHz, CDCl_3_) δ: −120.24 (1F, s), −132.07 (1F, s). HRMS for C_19_H_31_F_2_N_3_O_5_S_2_ [(M+H)^+^]: calc. 484.1751, found 484.1749.

#### 3-(Cyclooctylamino)-2,6-difluoro-5-((2-hydroxyethyl)­amino)-4-((3-hydroxypropyl)­sulfonyl)­benzenesulfonamide (8)

A mixture of compound **5c** (50 mg, 0.109 mmol, 1 equiv) in ethanolamine (0.2 mL, 3.31 mmol, 30 equiv) was stirred in a pressure vial at 70 °C. After 12 h the mixture was cooled to room temperature, and diluted with water (2 mL). The solution was filtered and purified by flash chromatography on RP-C_18_ column, using MeCN in water as an eluent. Yield: 35 mg (65%), as yellow oil. ^**1**^ **H NMR** (400 MHz, MeOH-*d*_4_) δ: 1.44 – 1.80 (12H, m, cyclooctyl), 1.80 – 2.02 (4H, m, cyclooctyl and SO_2_CH_2_CH_2_CH_2_), 2.68 – 2.78 (1H, m, OH), 3.40 – 3.48 (2H, m, NCH_2_CH_2_O), 3.48 – 3.55 (2H, m, SO_2_CH_2_CH_2_CH_2_), 3.57 (1H, t, *J* = 5.5 Hz, OH), 3.62 (2H, t, *J* = 6.1 Hz, SO_2_CH_2_CH_2_CH_2_), 3.69 – 3.74 (2H, m, NHCH_2_CH_2_O), 3.79 – 3.89 (1H, m, cyclooctyl CHNH), 4.86 (water overlapped with NHCH_2_CH_2_O, NHCH and SO_2_NH_2_). ^**13**^ **C NMR** (101 MHz, MeOH-*d*_4_) δ: 24.67 (cyclooctyl), 26.59 (cyclooctyl), 26.70 (SO_2_CH_2_ CH_2_CH_2_), 28.36 (cyclooctyl), 33.91 (cyclooctyl), 50.43 (NCH_2_CH_2_O, d, *J* = 11.5 Hz), 53.08 (SO_2_ CH_2_CH_2_CH_2_), 57.21 (cyclooctyl CHNH, d, *J* = 12.0 Hz), 60.60 (SO_2_CH_2_CH_2_ CH_2_), 62.22 (NCH_2_ CH_2_O, *J* = 3.2 Hz), 113.78 (C4, t, *J*(^19^F – ^13^C) = 3.8 Hz), 129.82 (C1, t, *J*(^19^F – ^13^C) = 17.5 Hz), 136.32 (C3 or C5, dd,^1^ *J*(^19^F – ^13^C) = 13.3 Hz,^2^ *J*(^19^F – ^13^C) = 3.3 Hz), 136.63 (C5 or C3, dd,^1^ *J*(^19^F – ^13^C) = 13.1 Hz,^2^ *J*(^19^F – ^13^C) = 3.1 Hz), 142.14 (C2 or C6, d, *J*(^19^F – ^13^C) = 243.0 Hz), 142.17 (C6 or C2, d, *J*(^19^F – ^13^C) = 242.6 Hz). ^**19**^ **F NMR** (376 MHz, MeOH-*d*_4_) δ: −136.00 (1F, d, *J* = 7.3 Hz), −137.69 (1F, dt,^1^ *J* = 8.2,^2^ *J* = 4.4 Hz). HRMS for C_19_H_31_F_2_N_3_O_6_S_2_ [(M+H)^+^]: calc. 500.1701, found 500.1714.

#### 3-(Cyclooctylamino)-2,6-difluoro-5-((4-hydroxybutyl)­amino)-4-((3-hydroxypropyl)­sulfonyl)­benzenesulfonamide (9)

A mixture of compound **5c** (50 mg, 0.11 mmol, 1 equiv) in 4-amino-1-butanol (0.2 mL, 2.13 mmol, 20 equiv) was stirred in a pressure vial at 70 °C. After 12 h the mixture was cooled to room temperature, and diluted with water (2 mL). The solution was filtered and purified by flash chromatography on RP-C_18_ column, using MeCN in water as an eluent. Yield: 25 mg (43%), as yellow oil. ^**1**^ **H NMR** (400 MHz, MeOH-*d*_4_) δ: 1.49 – 1.77 (16H, m, cyclooctyl and NCH_2_CH_2_CH_2_CH_2_O), 1.86 – 1.95 (4H, m, cyclooctyl and SO_2_CH_2_CH_2_CH_2_), 3.36 (2H, td, *J* = 7.1, 3.7 Hz, NCH_2_CH_2_CH_2_CH_2_O), 3.41 – 3.46 (2H, m, SO_2_CH_2_CH_2_CH_2_), 3.59 (2H, t, *J* = 6.3 Hz, NCH_2_CH_2_CH_2_CH_2_O), 3.62 (2H, t, *J* = 6.0 Hz, SO_2_CH_2_CH_2_CH_2_), 3.82 – 3.90 (1H, m, cyclooctyl CHNH), 4.86 (water overlapped with CH_2_CH_2_CH_2_CH_2_OH, SO_2_CH_2_CH_2_CH_2_OH, NHCH_2_, NHCH and SO_2_NH_2_). ^**13**^ **C NMR** (101 MHz, MeOH-*d*_4_) δ: 24.69 (cyclooctyl), 26.68 (cyclooctyl), 26.73 (SO_2_CH_2_ CH_2_CH_2_), 28.27 (NCH_2_ CH_2_CH_2_CH_2_O, d, *J* = 2.5 Hz), 28.32 (cyclooctyl), 30.96 (NCH_2_CH_2_ CH_2_CH_2_O), 34.04 (cyclooctyl), 48.35 (NCH_2_CH_2_CH_2_CH_2_O, d, *J* = 12.0 Hz), 53.20 (SO_2_ CH_2_CH_2_CH_2_), 57.18 (cyclooctyl CHNH, d, *J* = 11.9 Hz), 60.49 (SO_2_CH_2_CH_2_ CH_2_), 62.55 (NCH_2_CH_2_CH_2_ CH_2_O), 113.47 (C4, t, *J*(^19^F – ^13^C) = 4.1 Hz), 128.68 (C1, t, *J*(^19^F – ^13^C) = 16.5 Hz), 136.12 (C3 or C5, dd,^1^ *J*(^19^F – ^13^C) = 12.8 Hz,^2^ *J*(^19^F – ^13^C) = 3.0 Hz), 137.25 (C5 or C3, dd,^1^ *J*(^19^F – ^13^C) = 12.8 Hz,^2^ *J*(^19^F – ^13^C) = 2.8 Hz), 141.71 (C2 or C6, dd,^1^ *J*(^19^F – ^13^C) = 245.8 Hz,^2^ *J*(^19^F – ^13^C) = 4.0 Hz), 141.71 (C2 or C6, dd,^1^ *J*(^19^F – ^13^C) = 245.8 Hz,^2^ *J*(^19^F – ^13^C) = 4.0 Hz). ^**19**^ **F NMR** (376 MHz, MeOH-*d*_4_) δ: −136.87 (1F, d, *J* = 8.3 Hz), −138.25 (1F, dt,^1^ *J* = 8.0 Hz,^2^ *J* = 4.0 Hz). HRMS for C_21_H_35_F_2_N_3_O_6_S_2_ [(M+H)^+^]: calc. 528.2014, found 528.2017.

#### 3-(Cyclooctylamino)-2,6-difluoro-4-((3-hydroxypropyl)­sulfonyl)-5-(piperidin-1-yl)­benzenesulfonamide (10)

A mixture of compound **5c** (150 mg, 0.32 mmol, 1 equiv) and piperidine (64 μL, 0.65 mmol, 2 equiv) in anhydrous MeOH (2 mL) was stirred in a pressure vial at 70 °C. After 6 h the mixture was cooled to room temperature, poured to water (20 mL), and extracted with EtOAc (3 × 20 mL). Organic extracts were washed with brine (2 × 20 mL), dried over Na_2_SO_4_, filtered and concentrated under reduced pressure. The crude product was purified by flash chromatography (silica, /EtOAc/Hexane/DCM (1:1:1)). Yield: 50 mg (30%), as yellow oil. ^**1**^ **H NMR** (400 MHz, CDCl_3_) δ: 1.41 – 1.90 (18H, m, cyclooctyl and piperidine), 1.94 – 2.05 (2H, m, SO_2_CH_2_CH_2_CH_2_), 2.21 – 2.31 (1H, m, piperidine), 2.46 – 2.52 (1H, m, piperidine), 2.94 – 3.02 (2H, m, piperidine), 3.03 – 3.15 (2H, m, piperidine), 3.69 – 3.82 (5H, m, cyclooctyl CHNH, SO_2_CH_2_CH_2_CH_2_ and SO_2_CH_2_CH_2_CH_2_), 4.35 (1H, t, *J* = 7.1 Hz, OH), 5.57 (2H, s, SO_2_NH_2_), 7.22 (1H, d, *J* = 7.1 Hz, cyclooctyl CHNH). ^**13**^ **C NMR** (101 MHz, CDCl_3_) δ: 23.54 (cyclooctyl), 23.80 (piperidine), 25.56 (SO_2_CH_2_ CH_2_CH_2_), 26.00 (piperidine), 26.08 (cyclooctyl), 27.40 (cyclooctyl), 32.89 (cyclooctyl), 52.31 (CH_2_NCH_2_, d, *J* = 4.9 Hz), 54.52 (SO_2_ CH_2_CH_2_CH_2_), 56.51 (cyclooctyl CHNH, d, *J* = 11.5 Hz), 60.61 (SO_2_CH_2_CH_2_ CH_2_), 125.36 (C1, t, *J*(^19^F – ^13^C) = 16.2 Hz), 125.57 (C4, td,^1^ *J*(^19^F – ^13^C) = 6.0 Hz,^2^ *J*(^19^F – ^13^C) = 5.0 Hz,^3^ *J*(^19^F – ^13^C) = 1.8 Hz), 137.35 (C3 or C5, dd,^1^ *J*(^19^F – ^13^C) = 12.9 Hz,^2^ *J*(^19^F – ^13^C) = 2.4 Hz), 137.48 (C5 or C3, dd,^1^ *J*(^19^F – ^13^C) = 14.9 Hz,^2^ *J*(^19^F – ^13^C) = 4.4 Hz), 149.03 (C2 or C6, dd,^1^ *J*(^19^F – ^13^C) = 253.5 Hz,^2^ *J*(^19^F – ^13^C) = 3.9 Hz), 147.53 (C6 or C2, dd,^1^ *J*(^19^F – ^13^C) = 257.7 Hz,^2^ *J*(^19^F – ^13^C) = 3.7 Hz). ^**19**^ **F NMR** (376 MHz, CDCl_3_) δ: −120.15 (1F, s), −130.98 (1F, s). HRMS for C_22_H_35_F_2_N_3_O_5_S_2_ [(M+H)^+^]: calc. 524.2064, found 524.2073.

#### 3-(Benzylamino)-5-(cyclooctylamino)-2,6-difluoro-4-((3-hydroxypropyl)­sulfonyl)­benzenesulfonamide (11)

A mixture of compound **5c** (150 mg, 0.32 mmol, 1 equiv) and benzylamine (71 μL, 0.65 mmol, 2 equiv) in anhydrous MeOH (2 mL) was stirred in a pressure vial at 70 °C. After 6 h the mixture was cooled to room temperature, poured to water (20 mL), and extracted with EtOAc (3 × 20 mL). Organic extracts were washed with brine (2 × 20 mL), dried over Na_2_SO_4_, filtered and concentrated under reduced pressure. The crude product was purified by flash chromatography (silica, EtOAc/Hexane/DCM (1:1:1)). Yield: 68 mg (41%), as yellow oil. ^**1**^ **H NMR** (400 MHz, CDCl_3_) δ: 1.41 – 1.77 (13H, m, cyclooctyl and OH), 1.81 – 1.92 (4H, m, cyclooctyl and SO_2_CH_2_CH_2_CH_2_), 3.11 – 3.27 (2H, m, SO_2_CH_2_CH_2_CH_2_), 3.64 (2H, t, *J* = 5.9 Hz, SO_2_CH_2_CH_2_CH_2_), 3.77 – 3.94 (1H, m, cyclooctyl CHNH), 4.48 (2H, s, PhCH_2_), 5.38 (2H, s, SO_2_NH_2_), 6.35 – 6.68 (2H, m, NHCH_2_ and NHCH), 7.25 – 7.37 (5H, m, Ph). ^**13**^ **C NMR** (101 MHz, CDCl_3_) δ: 23.59 (cyclooctyl), 25.20 (SO_2_CH_2_CH_2_CH_2_), 25.65 (cyclooctyl), 27.24 (cyclooctyl), 33.27 (cyclooctyl), 51.33 (PhCH_2_, d, *J* = 13.1 Hz), 51.90 (SO_2_CH_2_CH_2_CH_2_), 56.09 (cyclooctyl CHNH, d, *J* = 11.9 Hz), 60.27 (SO_2_CH_2_CH_2_CH_2_), 112.20 (C4, t, *J*(^19^F – ^13^C) = 4.4 Hz), 126.20 (C1, t, *J*(^19^F – ^13^C) = 15.9 Hz), 127.79 (Ph), 128.03 (Ph), 128.93 (Ph), 135.31 (C3 or C5, dd,^1^ *J*(^19^F – ^13^C) = 12.5 Hz,^2^ *J*(^19^F – ^13^C) = 2.5 Hz), 135.59 (C5 or C3, dd,^1^ *J*(^19^F – ^13^C) = 12.4 Hz,^2^ *J*(^19^F – ^13^C) = 2.6 Hz), 139.10 (Ph), 139.90 (C2 or C6, dd,^1^ *J*(^19^F – ^13^C) = 244.6 Hz,^2^ *J*(^19^F – ^13^C) = 3.4 Hz), 140.19 (C6 or C2, dd,^1^ *J*(^19^F – ^13^C) = 247.0 Hz,^2^ *J*(^19^F – ^13^C) = 3.4 Hz). ^**19**^ **F NMR** (376 MHz, CDCl_3_) δ: −136.69 (1F, d, *J* = 9.8 Hz), −137.48 (1F, dd,^1^ *J* = 9.7 Hz,^2^ *J* = 3.4 Hz). HRMS for C_24_H_33_F_2_N_3_O_5_S_2_ [(M+H)^+^]: calc. 546.1908, found 546.1917

#### 3-Amino-5-(cyclooctylamino)-2,6-difluoro-4-((3-hydroxypropyl)­sulfonyl)­benzenesulfonamide (12)

To a solution of compound **11** (60 mg, 0.110 mmol, 1 equiv) in anhydrous THF (2 mL) 10% Pd/C (10 mg) was added. The mixture was stirred under a hydrogen atmosphere (5 bar) for 6 h at room temperature. The catalyst was removed by filtration through a Celite pad, and then washed with THF. Filtrates were evaporated to dryness *in vacuo*, redissolved in MeOH/DCM (1:9) mixture and filtered through a silica pad. Yield: 39 mg (78%), as a yellow solid. ^**1**^ **H NMR** (400 MHz, DMSO-*d*_6_) δ: 1.39 – 1.59 (10H, m, cyclooctyl), 1.59 – 1.69 (2H, m, cyclooctyl), 1.70 – 1.89 (4H, m, cyclooctyl and SO_2_CH_2_ CH_2_CH_2_), 3.38 – 3.49 (4H, m, SO_2_CH_2_CH_2_CH_2_ and SO_2_CH_2_CH_2_CH_2_), 3.67 – 3.79 (1H, m, cyclooctyl CHNH), 4.69 (1H, t, *J* = 5.3 Hz, OH), 6.08 (2H, s, NH_2_), 6.24 (1H, dd, *J* = 8.5, 2.0 Hz, cyclooctyl CHNH), 8.05 (2H, s, SO_2_NH_2_). ^**13**^ **C NMR** (101 MHz, DMSO-*d*_6_) δ: 23.00 (2C, cyclooctyl), 25.09 (SO_2_CH_2_ CH_2_CH_2_), 25.39 (cyclooctyl), 26.73 (cyclooctyl), 32.29 (cyclooctyl), 51.17 (SO_2_ CH_2_CH_2_CH_2_), 55.12 (cyclooctyl CHNH, d, *J* = 11.2 Hz), 58.47 (SO_2_CH_2_CH_2_ CH_2_), 107.90 (C4, m), 126.42 (C1, dd,^1^ *J*(^19^F – ^13^C) = 17.6 Hz,^2^ *J*(^19^F – ^13^C) = 14.6 Hz), 133.36 (C3 or C5, dd,^1^ *J*(^19^F – ^13^C) = 12.5 Hz,^2^ *J*(^19^F – ^13^C) = 2.9 Hz), 135.08 (C5 or C3, dd,^1^ *J*(^19^F – ^13^C) = 16.0 Hz,^2^ *J*(^19^F – ^13^C) = 2.0 Hz), 137.40 (C2 or C6, dd,^1^ *J*(^19^F – ^13^C) = 243.0 Hz,^2^ *J*(^19^F – ^13^C) = 5.3 Hz), 137.62 (C6 or C2, dd,^1^ *J*(^19^F – ^13^C) = 240.4 Hz,^2^ *J*(^19^F – ^13^C) = 3.4 Hz). ^**19**^ **F NMR** (376 MHz, DMSO-*d*_6_) δ: −140.86 (1F, d, *J* = 9.2 Hz), −142.29 (1F, d, *J* = 9.9 Hz). HRMS for C_17_H_27_F_2_N_3_O_5_S_2_ [(M+H)^+^]: calc. 456.1438, found 456.1440.

#### 3-(Cyclooctylamino)-2,6-difluoro-4-((3-hydroxypropyl)­sulfonyl)-5-methoxybenzenesulfonamide (13)

A mixture of compound **5c** (100 mg, 0.22 mmol, 1 equiv) and 5.4 M solution of MeONa in MeOH (230 μL, 1.24 mmol, 5.6 equiv) in anhydrous MeOH (2 mL) was stirred in a pressure vial at 70 °C. After 5 days the mixture was cooled to room temperature, poured into water (20 mL), and extracted with EtOAc (3 × 20 mL). Organic extracts were washed with brine (2 × 20 mL), dried over Na_2_SO_4_, filtered and concentrated under reduced pressure. Crude material was dissolved in MeOH/DCM (1:9) mixture and filtered through a silica pad. Filtrates were concentrated *in vacuo* and additionally purified on preparative TLC, using MeOH/DCM (1:20). Yield: 22 mg (21%), as yellow oil. ^**1**^ **H NMR** (400 MHz, CDCl_3_) δ: 1.40 – 1.79 (13H, m, cyclooctyl and OH), 1.79 – 1.92 (2H, m, cyclooctyl), 1.92 – 2.09 (2H, m, SO_2_CH_2_CH_2_CH_2_), 3.49 – 3.59 (2H, m, SO_2_CH_2_CH_2_CH_2_), 3.75 (2H, t, *J* = 6.0 Hz, SO_2_CH_2_CH_2_CH_2_), 3.77 – 3.86 (1H, m, cyclooctyl CHNH), 3.98 (3H, d, *J* = 1.3 Hz, OCH_3_), 5.55 (2H, s, SO_2_NH_2_), 7.01 (1H, d, *J* = 8.6 Hz, cyclooctyl CHNH). ^**13**^ **C NMR** (101 MHz, CDCl_3_) δ: 23.47 (cyclooctyl), 25.56 (SO_2_CH_2_ CH_2_CH_2_), 25.75 (cyclooctyl), 27.38 (cyclooctyl), 32.91 (cyclooctyl), 54.76 (SO_2_ CH_2_CH_2_CH_2_), 56.20 (cyclooctyl CHNH, d, *J* = 11.4 Hz), 60.52 (SO_2_CH_2_CH_2_ CH_2_), 63.30 (CH_3_O, d, *J* = 6.1 Hz), 120.22 (C4, d, *J*(^19^F – ^13^C) = 6.2 Hz), 125.93 (C1, dd,^1^ *J*(^19^F – ^13^C) = 16.7 Hz,^2^ *J*(^19^F – ^13^C) = 14.7 Hz), 136.34 (C3, dd,^1^ *J*(^19^F – ^13^C) = 13.4 Hz,^2^ *J*(^19^F – ^13^C) = 2.8 Hz), 142.17 (C2 or C6, dd,^1^ *J*(^19^F – ^13^C) = 248.9 Hz,^2^ *J*(^19^F – ^13^C) = 4.3 Hz), 144.45 (C5, dd,^1^ *J*(^19^F – ^13^C) = 14.6 Hz,^2^ *J*(^19^F – ^13^C) = 4.0 Hz), 144.63 (C6 or C2, dd,^1^ *J*(^19^F – ^13^C) = 254.3 Hz,^2^ *J*(^19^F – ^13^C) = 3.0 Hz). ^**19**^ **F NMR** (376 MHz, CDCl_3_) δ: −125.70 (1F, d, *J* = 7.8 Hz), −142.63 (1F, d, *J* = 7.8 Hz). HRMS for C_18_H_28_F_2_N_2_O_6_S_2_ [(M+H)^+^]: calc. 471.1435, found 471.1434.

#### 3-(Cyclooctylamino)-5-ethoxy-2,6-difluoro-4-((3-hydroxypropyl)­sulfonyl)­benzenesulfonamide (14)

A mixture of compound **5c** (300 mg, 0.65 mmol, 1 equiv) and EtONa (950 mg, 14.0 mmol, 21 equiv) in anhydrous EtOH (5 mL) was stirred in a pressure vial at 70 °C. After 20 h the mixture was cooled to room temperature and concentrated *in vacuo*. The residue was mixed with water (20 mL) and extracted with EtOAc (3 × 20 mL). Organic extracts were washed with brine (2 × 20 mL), dried over Na_2_SO_4_, filtered and concentrated in a vacuum. The crude product was purified by flash chromatography (silica, EtOAc/Hexane/DCM (1:1:1)). Obtained product was additionally purified on a preparative glass TLC plate using MeOH/CHCl_3_ (1:20) as an eluent. Yield: 10 mg (3%), as yellow oil. ^**1**^ **H NMR** (400 MHz, CDCl_3_) δ: 1.44 (3H, t, *J* = 7.0 Hz, CH_3_CH_2_O), 1.48 – 1.75 (13H, m, cyclooctyl and OH), 1.79 – 1.93 (2H, m, cyclooctyl), 1.99 (2H, dq,^1^ *J* = 12.0,^2^ *J* = 6.1 Hz, SO_2_CH_2_CH_2_CH_2_), 3.51 – 3.65 (2H, m, SO_2_CH_2_CH_2_CH_2_), 3.76 (2H, t, *J* = 6.0 Hz, SO_2_CH_2_CH_2_CH_2_), 3.75 – 3.88 (1H, m, cyclooctyl CHNH), 4.21 (2H, q, *J* = 7.0 Hz, CH_3_CH_2_O), 5.47 (2H, s, SO_2_NH_2_), 6.93 – 7.13 (1H, m, cyclooctyl CHNH). ^**13**^ **C NMR** (101 MHz, CDCl_3_) δ: 15.44 (CH_3_CH_2_O), 23.51 (cyclooctyl), 25.59 (SO_2_CH_2_ CH_2_CH_2_), 25.81 (cyclooctyl), 27.41 (cyclooctyl), 32.96 (cyclooctyl), 54.70 (SO_2_ CH_2_CH_2_CH_2_), 56.23 (cyclooctyl CHNH, d, *J* = 11.4 Hz), 60.62 (SO_2_CH_2_CH_2_ CH_2_), 72.57 (CH_3_ CH_2_O, d, *J* = 5.8 Hz), 120.44 (C4, d, *J*(^19^F – ^13^C) = 6.4 Hz), 125.81 (C1, dd,^1^ *J*(^19^F – ^13^C) = 16.5 Hz,^2^ *J*(^19^F – ^13^C) = 14.9 Hz), 136.39 (C3, dd,^1^ *J*(^19^F – ^13^C) = 13.4 Hz,^2^ *J*(^19^F – ^13^C) = 2.9 Hz), 142.28 (C2 or C6, dd,^1^ *J*(^19^F – ^13^C) = 248.4 Hz,^2^ *J*(^19^F – ^13^C) = 3.8 Hz), 143.75 (C5, dd,^1^ *J*(^19^F – ^13^C) = 14.9 Hz,^2^ *J*(^19^F – ^13^C) = 4.0 Hz), 144.49 (C6 or C2, dd,^1^ *J*(^19^F – ^13^C) = 254.0 Hz,^1^ *J*(^19^F – ^13^C) = 2.7 Hz). ^**19**^ **F NMR** (376 MHz, CDCl_3_) δ: −126.05 (1F, d, *J* = 10.0 Hz), −142.30 (1F, d, *J* = 7.8 Hz). HRMS for C_19_H_30_F_2_N_2_O_6_S_2_ [(M+H)^+^]: calc. 485.1592, found 485.1588.

#### 3,5-Bis­(cyclooctylamino)-2,6-trifluoro-4-((2-acetamido)­ethylsulfonyl)­benzenesulfonamide (15)

A mixture of compound **5a** (350 mg, 0.72 mmol, 1 equiv), cyclooctylamine (202 μL, 1.47 mmol, 2.04 equiv) and Et_3_N (205 μL, 1.47 mmol, 2.04 equiv) in DMSO (2 mL) was stirred for 17 h at 75 °C. Afterward, the reaction mixture was washed with brine (10 mL) and extracted with EtOAc (3 × 15 mL). The organic phase was dried using anhydrous Na_2_SO_4_ and evaporated under reduced pressure. The product was purified by column chromatography (silica, EtOAc, *R*_F_ = 0.65). Yield: 201 mg (47%), as yellow solid, Mp: 156 – 157 °C. ^**1**^ **H NMR** (400 MHz, CDCl_3_) δ: 1.48 – 1.61 (20H, m, cyclooctyl), 1.62 – 1.71 (4H, m, cyclooctyl), 1.82 – 1.91 (4H, m, cyclooctyl), 1.94 (3H, s, C­(O)­CH_3_) 3.44 (2H, t, *J* = 5.6 Hz, SO_2_CH_2_CH_2_), 3.71 (2H, q, *J* = 5.9 Hz, SO_2_CH_2_CH_2_), 3.85 (2H, br. s, cyclooctyl CHNH), 5.60 (2H, s, SO_2_NH_2_), 6.14 (1H, t, *J* = 6.0 Hz, NHC­(O)). ^**13**^ **C NMR** (100 MHz, CDCl_3_) δ: 23.10 (C­(O)CH_3_), 23.65 (cyclooctyl), 25.70 (cyclooctyl), 27.24 (cyclooctyl), 33.44 (cyclooctyl), 33.58 (SO_2_CH_2_ CH_2_), 53.96 (SO_2_ CH_2_CH_2_), 56.19 (cyclooctyl CHNH, t, *J* = 5.8 Hz) 111.29 (C4, t, *J*(^19^F – ^13^C) = 4.7 Hz), 126.60 (C1, t, *J*(^19^F – ^13^C) = 16.0 Hz), 135.08 (C3 and C5, dd,^1^ *J*(^19^F – ^13^C) = 10.0 Hz,^2^ *J*(^19^F – ^13^C) = 5.3 Hz), 139.34 (C2 and C6, dd,^1^ *J*(^19^F – ^13^C) = 241.9 Hz,^2^ *J*(^19^F – ^13^C) = 4.3 Hz), 170.45 (CO). ^**19**^ **F NMR** (376 MHz, CDCl_3_) δ: −138.42 (2F, s). HRMS for C_26_H_42_F_2_N_4_O_5_S_2_ [(M+H)^+^]: calc. 593.2637, found 593.2642.

#### 3-(Cyclooctylamino)-5-(cyclopentylamino)-2,6-trifluoro-4-((2-acetamido)­ethylsulfonyl)­benzenesulfonamide (16)

A mixture of compound **5a** (227 mg, 0.47 mmol, 1 equiv), cyclopentylamine (94 μL, 0.94 mmol, 2.04 equiv) and Et_3_N (133 μL, 0.94 mmol, 2.04 equiv) in DMSO (1 mL) was stirred for 12 h at 75 °C temperature. Afterward, the reaction mixture was washed with brine (10 mL) and extracted with EtOAc (3 × 15 mL). The organic phase was dried using anhydrous Na_2_SO_4_ and evaporated under reduced pressure. The product was purified by column chromatography (silica, EtOAc, *R*_F_ = 0.48). Yield: 136 mg (53%), as yellow solid, Mp: 168 – 169 °C. ^**1**^ **H NMR** (400 MHz, CDCl_3_) δ: 1.44 – 1.72 (20H, m, cyclooctyl and cyclopentyl), 1.82 – 1.90 (2H, m, cyclooctyl and cyclopentyl), 1.93 (3H, s, C­(O)­CH_3_), 3.44 (2H, dd,^1^ *J* = 6.6 Hz,^2^ *J* = 4.7 Hz, SO_2_CH_2_CH_2_), 3.71 (2H, t, *J* = 5.8 Hz, SO_2_CH_2_CH_2_), 3.85 (1H, br. s, cyclooctyl CHNH), 4.10 (1H, p, *J* = 5,5 Hz, cyclopentyl CHNH), 5.57 (2H, s, SO_2_NH_2_), 6.11 (1H, t, *J* = 6,0 Hz, NHCO), 6.25 (1H, d, *J* = 6 Hz, cyclooctyl CHNH), 6.35 (1H, d, *J* = 6,4 Hz, cyclopentyl CHNH). ^**13**^ **C NMR** (100 MHz, CDCl_3_) δ: 23.09 (C­(O)­CH_3_), 23.63 (cyclooctyl), 23.70 (cyclopentyl), 25.67 (cyclooctyl), 27.26 (cyclooctyl), 33.37 (cyclooctyl), 33.61 (SO_2_CH_2_ CH_2_), 34.69 (cyclopentyl CH_2_, d, *J* = 2.2 Hz), 53.89 (SO_2_ CH_2_CH_2_), 56.20 (cyclooctyl CHNH, d, *J* = 11.9 Hz), 58.16 (cyclopentyl CHNH, d, *J* = 11.3 Hz), 110.97 (C4, t, *J*(^19^F – ^13^C) = 4.7 Hz), 126.68 (C1, t, *J*(^19^F – ^13^C) = 16.2 Hz), 135.13 (C3, dd,^1^ *J*(^19^F – ^13^C) = 12.5 Hz,^2^ *J*(^19^F – ^13^C) = 2.9 Hz), 135.49 (C5, dd,^1^ *J*(^19^F – ^13^C) = 12.7 Hz,^2^ *J*(^19^F – ^13^C) = 2.9 Hz), 139.14 (C2 and C6, dd,^1^ *J*(^19^F – ^13^C) = 243.0 Hz,^2^ *J*(^19^F – ^13^C) = 39.5 Hz, 170.34 (CO). ^**19**^ **F NMR** (376 MHz, CDCl_3_) δ: −138.36 (1F, d, *J* = 10.1 Hz), −138.75 (1F, d, *J* = 10,1 Hz). HRMS for C_23_H_36_F_2_N_4_O_5_S_2_ [(M+H)^+^]: calc. 551.2168, found 551.2180.

#### 3-(Cyclooctylamino)-5-(cyclopropylamino)-2,6-difluoro-4-((2-hydroxyethyl)­sulfonyl)­benzenesulfonamide (17)

A mixture of compound **5a** (300 mg, 0.62 mmol, 1 equiv), cyclopropylamine (130 μL, 1.26 mmol, 2.04 equiv) and Et_3_N (176 μL, 1.26 mmol, 2.04 equiv) in DMSO (1.5 mL) was stirred for 8 h at 75 °C. Afterward, reaction mixture was washed with brine (10 mL) and extracted with EtOAc (3 × 15 mL). The organic phase was dried using anhydrous Na_2_SO_4_ and evaporated under reduced pressure. The product was purified by column chromatography (silica, EtOAc, *R*_F_ = 0.48). Yield: 253 mg (78%), as yellow solid, Mp: 108 – 109 °C. ^**1**^ **H NMR** (400 MHz, CDCl_3_) δ: 1.46–1.70 (15H, m, cyclooctyl and cyclopropyl), 1.82 – 1.90 (3H, m, cyclooctyl and cyclopropyl) 1.93 (3H, s, C­(O)­CH_3_), 2.88 – 2.96 (1H, m, NHCH cyclopropyl), 3.38 (2H, dd,^1^ *J* = 5.6 Hz, ^2^ *J* = 4.9 Hz, SO_2_CH_2_CH_2_), 3.67 (2H, q, *J* = 5.8 Hz, SO_2_CH_2_CH_2_), 3.84 (1H, br. s, NHCH cyclooctyl), 5.61 (2H, s, SO_2_NH_2_), 6.08 (1H, t, *J* = 6.0 Hz, NHC­(O)), 6.37 (2H, br. s, cyclooctyl CHNH and cyclopropyl CHNH). ^**13**^ **C NMR** (100 MHz, CDCl_3_) δ: 8.66 (cyclopropyl CH_2_, d, *J* = 5.1 Hz), 23.04 (C­(O)CH_3_), 23.58 (cyclooctyl), 25.62 (cyclooctyl), 27.26 (cyclooctyl), 28.73 (cyclopropyl CHNH, d, *J* = 12.2 Hz), 33.21 (cyclooctyl), 33.73 (SO_2_CH_2_ CH_2_), 53.60 (SO_2_ CH_2_CH_2_), 56.38 (cyclooctyl CHNH, d, *J* = 11.8 Hz), 111.31 (C4, t, *J*(^19^F – ^13^C) = 4.3 Hz), 126.76 (C1, t, *J*(^19^F – ^13^C) = 15.8 Hz), 134.79 (C3, dd,^1^ *J*(^19^F – ^13^C) = 13.6 Hz,^2^ *J*(^19^F – ^13^C) = 3.0 Hz), 135.72 (C5, dd,^1^ *J*(^19^F – ^13^C) = 12.1 Hz,^2^ *J*(^19^F – ^13^C) = 2.9 Hz), 139.77 (C2 and C6, ddd,^1^ *J*(^19^F – ^13^C) = 244.9 Hz,^2^ *J*(^19^F – ^13^C) = 48.2 Hz, ^3^ *J*(^19^F – ^13^C) = 3.5 Hz), 170.69 (CO). ^**19**^ **F NMR** (376 MHz, CDCl_3_) δ: −136.87 (1F, d, *J* = 9.9 Hz), −137.32 (1F, br. s). HRMS for C_21_H_32_F_2_N_4_O_5_S_2_ [(M+H)^+^]: calc. 523.1855, found 523.1857.

#### 3-(Cyclooctylamino)-5-(ethylamino)-2,6-difluoro-4-((2-hydroxyethyl)­sulfonyl)­benzenesulfonamide (18)

A mixture of **5b** (100 mg, 0.23 mmol, 1 equiv) and ethylamine (70%) (37 μL, 0.46 mmol, 2 equiv) in DMSO (1 mL) was stirred for 36 h at 56 °C. Afterward, the reaction mixture was washed with brine (10 mL) and extracted with EtOAc (2 × 10 mL). The organic phase was dried using anhydrous Na_2_SO_4_ and evaporated under reduced pressure. The product was purified by column chromatography (silica, EtOAc/CHCl_3_ (1:1), *R*_F_ = 0.54). Yield: 39 mg (37%), as yellow oil. ^**1**^ **H NMR** (400 MHz, DMSO-*d*_6_) δ: 1.15 (3H, t, *J* = 7.2 Hz, CH_3_), 1.41 – 1.69 (12H, m, cyclooctyl), 1.78 – 1.87 (2H, m, cyclooctyl), 3.22 – 3.31 (2H, m, NHCH_2_CH_3_), 3.59 (2H, t, *J* = 5.8 Hz, SO_2_CH_2_CH_2_), 3.72 (1H, m, cyclooctyl CHNH), 3.76 (2H, q, *J* = 5.6 Hz, SO_2_CH_2_CH_2_), 5.02 (1H, t, *J* = 5.4 Hz, OH), 5.85 (1H, t, *J* = 3.9 Hz, NHCH_2_CH_3_), 6.33 (1H, d, *J* = 8.5 Hz, NHCH), 8.06 (2H, s, SO_2_NH_2_). ^**13**^ **C NMR** (100 MHz, DMSO-*d*_6_) δ: 15.79 (NHCH_2_ CH_3_, d, *J* = 2.6 Hz), 23.07 (cyclooctyl), 25.12 (cyclooctyl), 26.73 (cyclooctyl), 32.40 (cyclooctyl), 41.81 (NHCH_2_CH_3_, d, *J* = 11.7 Hz), 54.82 (SO_2_CH_2_ CH_2_), 55.46 (cyclooctyl CHNH, d, *J* = 11.7 Hz), 57.48 (SO_2_ CH_2_CH_2_), 113.93 (C4, t, *J*(^19^F – ^13^C) = 3.8 Hz), 127.18 (C1, t, *J*(^19^F – ^13^C) = 16.4 Hz), 134.76 (C3 and C5, ddd,^1^ *J*(^19^F – ^13^C) = 112.4 Hz), ^2^ *J*(^19^F – ^13^C) = 12.4 Hz,^3^ *J*(^19^F – ^13^C) = 2.1 Hz), 140.11 (C2 and C6, ddd,^1^ *J*(^19^F – ^13^C) = 243.5 Hz), ^2^ *J*(^19^F – ^13^C) = 10.8 Hz,^3^ *J*(^19^F – ^13^C) = 5.0 Hz). ^**19**^ **F NMR** (376 MHz, DMSO-*d*_6_) δ: −135.14 (1F, d, *J* = 8.2 Hz), −136.68 (1F, s). HRMS for C_18_H_29_F_2_N_3_O_5_S_2_ [(M+H)^+^]: calc. 470.1589, found 470.1595.

#### 3-(Cyclooctylamino)-2,6-difluoro-4-((2-hydroxyethyl)­sulfonyl)-5-(propylamino)­benzenesulfonamide (19)

A mixture of compound **5b** (100 mg, 0.23 mmol, 1 equiv), propylamino hydrochloride (32 mg, 0.34 mmol, 1.5 equiv) and Et_3_N (78 μL, 0.56 mmol, 2.5 equiv) in DMSO (1 mL) was stirred for 36 h at 56 °C. Afterward, the reaction mixture was washed with brine (10 mL) and extracted with EtOAc (2 × 10 mL). The organic phase was dried using anhydrous Na_2_SO_4_ and evaporated under reduced pressure. The product was purified by column chromatography (silica, EtOAc/CHCl_3_ (1:1), *R*_F_ = 0.65). Yield: 37 mg (36%), as yellow oil. ^**1**^ **H NMR** (400 MHz, DMSO-*d*_6_) δ: 0.91 (3H, t, *J* = 7.3 Hz, CH_3_), 1.40 – 1.69 (14H, m, cyclooctyl and NHCH_2_CH_2_), 1.77 – 1.86 (2H, m, cyclooctyl), 3.14 – 3.24 (2H, m, NHCH_2_CH_2_), 3.58 (2H, t, *J* = 6.0 Hz, SO_2_CH_2_CH_2_), 3.72 (1H, m, CH cyclooctyl), 3.76 (2H, q, *J* = 5.6 Hz, SO_2_CH_2_CH_2_), 5.03 (1H, t, *J* = 5.4 Hz, OH), 5.96 (1H, t, *J* = 5.5 Hz, NHCH_2_CH_3_), 6.30 (1H, d, *J* = 7.9 Hz, cyclooctyl CHNH), 8.07 (2H, s, SO_2_NH_2_). ^**13**^ **C NMR** (100 MHz, DMSO-*d*_6_) δ: 11.32 (NHCH_2_CH_2_ CH_3_), 23.08 (cyclooctyl), 23.40 (NHCH_2_ CH_2_CH_3_, d, *J* = 2.6 Hz), 25.12 (cyclooctyl), 26.73 (cyclooctyl), 32.41 (cyclooctyl), 48.87 (NHCH_2_, d, *J* = 11.3 Hz), 54.81 (SO_2_CH_2_ CH_2_), 55.46 (cyclooctyl CHNH, d, *J* = 11.3 Hz), 57.45 (SO_2_ CH_2_CH_2_), 113.53 (C4), 127.23 (C1, t, *J*(^19^F – ^13^C) = 15.9 Hz), 134.83 (C3 and C5, ddd, ^1^ *J*(^19^F – ^13^C) = 133.1 Hz), ^2^ *J*(^19^F – ^13^C) = 13.2 Hz,^3^ *J*(^19^F – ^13^C) = 3.3 Hz), 140.45 (C2 and C6, ddd,^1^ *J*(^19^F – ^13^C) = 244.6 Hz, ^2^ *J*(^19^F – ^13^C) = 11.8 Hz,^3^ *J*(^19^F – ^13^C) = 3.4 Hz). ^**19**^ **F NMR** (376 MHz, DMSO-*d*_6_) δ: −135.55 (1F, d, *J* = 7.1 Hz), −136.70 (1F, s). HRMS for C_19_H_31_F_2_N_3_O_5_S_2_ [(M+H)^+^]: calc. 484.1746, found 484.1760.

#### 3-(Cyclooctylamino)-5-(cyclopentylamino)-2,6-difluoro-4-((2-hydroxyethyl)­sulfonyl)­benzenesulfonamide (20)

A mixture of compound **5b** (200 mg, 0.50 mmol, 1 equiv), cyclopentylamine (100 μL, 1.01 mmol, 2 equiv) and Et_3_N (142 μL, 1.01 mmol, 2 equiv) in DMSO (1 mL) was stirred for 10 h at 75 °C. Afterward, the reaction mixture was washed with brine (10 mL) and extracted with EtOAc (3 × 15 mL). The organic phase was dried using anhydrous Na_2_SO_4_ and evaporated under reduced pressure. The product was purified by column chromatography (silica, EtOAc/CHCl_3_ (1:1), *R*_F_ = 0.65). Yield: 59 mg (23%), as yellow oil. ^**1**^ **H NMR** (400 MHz, DMSO-*d*_6_) δ: 1.40 – 1.70 (22H, m, cyclooctyl and cyclopentyl), 3.56 (2H, t, *J* = 5.9 Hz, SO_2_CH_2_CH_2_), 3.74 (1H, br. s, cyclopentyl CHNH), 3.76 (2H, q, *J* = 5.6 Hz, SO_2_CH_2_CH_2_), 3.99 (1H, m, cyclooctyl CHNH), 5.05 (1H, t, *J* = 5.2 Hz, OH), 6.15 (1H, d, *J* = 7.0 Hz, cyclopentyl CHNH), 6.22 (1H, t, *J* = 8.3 Hz, cyclooctyl CHNH), 8.05 (2H, s, SO_2_NH_2_). ^**13**^ **C NMR** (100 MHz, DMSO-*d*_6_) δ: 23.10 (cyclooctyl), 25.16 (cyclooctyl), 26.88 (cyclooctyl), 32.52 (cyclopentyl), 33.73 (cyclopentyl), 33.75 (cyclooctyl) 54.66 (SO_2_CH_2_ CH_2_), 55.47 (cyclopentyl CHNH, d, *J* = 10.8 Hz), 57.20 (SO_2_ CH_2_CH_2_), 57.76 (cyclooctyl CHNH, d, *J* = 10.2 Hz), 112.82 (C4, t, *J*(^19^F – ^13^C) = 4.2 Hz), 127.31 (C1, t, *J*(^19^F – ^13^C) = 16.6 Hz), 134.41 (C3 and C5, ddd,^1^ *J*(^19^F – ^13^C) = 59.2 Hz, ^2^ *J*(^19^F – ^13^C) = 12.7 Hz,^3^ *J*(^19^F – ^13^C) = 4.7 Hz), 139.39 (C2 and C6, ddd,^1^ *J*(^19^F – ^13^C) = 243.8 Hz,^2^ *J*(^19^F – ^13^C) = 26.2 Hz,^3^ *J*(^19^F – ^13^C) = 4.3 Hz). ^**19**^ **F NMR** (376 MHz, DMSO-*d*_6_) δ: −136.38 (2F, q, *J* = 8.3 Hz). HRMS for C_21_H_33_F_2_N_3_O_5_S_2_ [(M+H)^+^]: calc. 510.1902, found 510.1892.

#### 6-(Cyclooctylamino)-7,9-difluoro-3,4-dihydro-2*H*-benzo­[*b*]­[1,4]­oxathiepine-8-sulfonamide-5,5-dioxide (21)

A mixture of compound **5c** (50 mg, 0.109 mmol, 1 equiv) in 1,8-diazabicyclo[5.4.0]­undec-7-ene (0.2 mL, 1.29 mmol, 12 equiv) was stirred in a pressure vial at 70 °C. After 12 h the mixture was cooled to room temperature, poured into water (5 mL) and extracted with DCM (2 × 5 mL). Organic extracts were washed with 5% aq. KHSO_4_ (4 × 2 mL), water (5 mL) and brine (5 mL), were dried over Na_2_SO_4_, filtered and evaporated. The crude product was purified by flash chromatography on RP-C_18_, using MeCN in water as an eluent. Yield: 11 mg (23%), as a colorless solid. ^**1**^ **H NMR** (400 MHz, CDCl_3_) δ: 1.43 – 1.64 (10H, m, cyclooctyl), 1.64 – 1.76 (2H, m, cyclooctyl), 1.82 – 1.93 (2H, m, cyclooctyl), 2.31 – 2.46 (2H, m, SO_2_CH_2_CH_2_CH_2_), 3.44 – 3.55 (2H, m, SO_2_CH_2_CH_2_CH_2_), 3.75 – 3.88 (1H, m, cyclooctyl CHNH), 4.16 – 4.30 (2H, m, SO_2_CH_2_CH_2_CH_2_), 5.50 (2H, s, SO_2_NH_2_), 6.55 – 6.70 (1H, br s, cyclooctyl CHNH). ^**13**^ **C NMR** (101 MHz, CDCl_3_) δ: 23.43 (cyclooctyl), 25.52 (SO_2_CH_2_ CH_2_CH_2_), 25.56 (cyclooctyl), 27.42 (cyclooctyl), 32.87 (cyclooctyl), 56.35 (cyclooctyl CHNH, d, *J* = 11.0 Hz), 57.79 (SO_2_ CH_2_CH_2_CH_2_), 73.43 (SO_2_CH_2_CH_2_ CH_2_), 124.46 (C4, d, *J*(^19^F – ^13^C) = 6.5 Hz), 125.58 (C1, dd,^1^ *J*(^19^F – ^13^C) = 16.4 Hz,^2^ *J*(^19^F – ^13^C) = 14.7 Hz), 135.30 (C3, dd,^1^ *J*(^19^F – ^13^C) = 13.6 Hz,^2^ *J*(^19^F – ^13^C) = 3.2 Hz), 141.92 (C2, dd,^1^ *J*(^19^F – ^13^C) = 248.4 Hz,^2^ *J*(^19^F – ^13^C) = 4.1 Hz), 142.25 (C5, dd,^1^ *J*(^19^F – ^13^C) = 16.4 Hz,^2^ *J*(^19^F – ^13^C) = 3.8 Hz), 145.42 (C6, dd,^1^ *J*(^19^F – ^13^C) = 256.2 Hz,^2^ *J*(^19^F – ^13^C) = 2.2 Hz). ^**19**^ **F NMR** (376 MHz, CDCl_3_) δ: −123.77 (1F, s), −143.06 (1F, d, *J* = 6.6 Hz). HRMS for C_17_H_24_F_2_N_2_O_5_S_2_ [(M+H)^+^]: calc. 439.1173, found 439.1153.

#### *tert*-Butyl­(2-((2,3,5,6-tetrafluoro-4-sulfamoylphenyl)­sulfonyl)­ethyl)­carbamate (22)

A mixture of compound **4a** (427 mg, 1.13 mmol, 1 equiv) and HCl conc. (2.5 mL) in MeOH (10 mL) was refluxed for 12 h. Afterward, the solvent was evaporated under reduced pressure, reaction mixture was washed with water. Obtained brown crystals (226 mg), (Boc)_2_O (119 mg, 0.545 mmol, 1 equiv) and Et_3_N (85 μL, 0.607 mmol, 1.1 equiv) were dissolved in THF (15 mL) and stirred for 4 h at room temperature. The solvent was evaporated under reduced pressure and the product was purified by column chromatography (silica gel, EtOAc/CHCl_3_ (1:1), *R*_F_ = 0.71). Yield: 160 mg (32%), as white solid, Mp: 153–154 °C. ^**1**^ **H NMR** (400 MHz, DMSO-*d*_6_) δ: 1.32 (9H, s, OC­(CH_3_)_3_), 3.41 (2H, q, *J* = 5.8 Hz, SO_2_CH_2_CH_2_), 3.74 (2H, t, *J* = 6.0 Hz, SO_2_CH_2_CH_2_), 6.93 (1H, t, *J* = 5,6 Hz, NH), 8.65 (2H, s, SO_2_NH_2_). ^**13**^ **C NMR** (100 MHz, DMSO-*d*_6_) δ: 27.94 (C­(CH_3_)_3_), 34,91 (SO_2_CH_2_CH_2_), 55.87 (SO_2_CH_2_CH_2_), 78.30 (C­(CH_3_)_3_), 121.30 (C1, t, *J*(^19^F – ^13^C) = 14.5 Hz), 127.63 (C4, t, *J*(^19^F – ^13^C) = 15.4 Hz), 142.90 (C2 and C6, dd^1^ *J*(^19^F – ^13^C) = 257.8 Hz,^2^ *J*(^19^F – ^13^C) = 10.2 Hz), 144.25 (C3 and C5, dd,^1^ *J*(^19^F – ^13^C) = 255.6 Hz,^2^ *J*(^19^F – ^13^C) = 12.0 Hz), 155.14 (NHC­(O)­O). ^**19**^ **F NMR** (376 MHz, DMSO-*d*_6_) δ: −135.77 – −136.11 (2F, m), −136.39 – −136.65 (2F, m). HRMS for C_13_H_16_F_4_N_2_O_6_S_2_ [(M+H)^+^]: calc. 437.0459, found 437.0462.

#### *tert*-Butyl­(2-((2-(cyclooctylamino)-3,5,6-trifluoro-4-sulfamoylphenyl)­sulfonyl)­ethyl)­carbamate (23)

A mixture of compound **22** (160 mg, 0.38 mmol, 1 equiv) and cyclooctylamine (1.04 mL, 0.76 mmol, 2 equiv) in DMSO (1 mL) was stirred for 4 h at room temperature. Afterward, the reaction mixture was washed with brine (10 mL) and extracted with EtOAc (3 × 15 mL). The organic phase was dried using anhydrous Na_2_SO_4_ and evaporated under reduced pressure. The product was purified by column chromatography (silica, EtOAc/CHCl_3_ (1:3), *R*_F_ = 0.60). Yield: 160 mg (76%), as yellow oil. ^**1**^ **H NMR** (400 MHz, DMSO-*d*_6_) δ: 1.33 (9H, s, C­(CH_3_)_3_), 1.46 – 1.69 (12H, m, cyclooctyl), 1.81 – 1.89 (2H, m, cyclooctyl), 3.35 (2H, m, SO_2_CH_2_CH_2_), 3.67 (2H, t, *J* = 6.0 Hz, SO_2_CH_2_CH_2_), 3.77 (1H, br. s, cyclooctyl CHNH), 6.58 (1H, d, *J* = 8.4 Hz, cyclooctyl CHNH), 6.94 (1H, t, *J* = 5.1 Hz, NHC­(O)­O), 8.34 (2H, s, SO_2_NH_2_). ^**13**^ **C NMR** (100 MHz, DMSO-*d*_6_) δ: 22.87 (cyclooctyl), 25.02 (cyclooctyl), 26.73 (cyclooctyl), 28.05 (C­(CH_3_)_3_), 32.27 (cyclooctyl), 34.32 (SO_2_CH_2_CH_2_), 55.43 (cyclooctyl CHNH, d, *J* (^19^F–^13^C) = 11,2 Hz), 55.67 (SO_2_CH_2_CH_2_), 78.29 (C­(CH_3_)_3_), 115.13 (C1, dd,^1^ *J*(^19^F – ^13^C) = 12.4 Hz,^2^ *J*(^19^F – ^13^C) = 4.9 Hz), 127.44 (C4, dd,^1^ *J*(^19^F – ^13^C) = 18.5 Hz,^2^ *J*(^19^F – ^13^C) = 14.2 Hz), 134.69 (C3, d, *J*(^19^F – ^13^C) = 12.8 Hz), 136.73 (C6, ddd,^1^ *J*(^19^F – ^13^C) = 247.1 Hz,^2^ *J*(^19^F – ^13^C) = 18.4 Hz,^3^ *J*(^19^F – ^13^C) = 3.8 Hz), 144.16 (C2, d, *J*(^19^F – ^13^C) = 254.8 Hz), 145.52 (C5, ddd,^1^ *J*(^19^F – ^13^C) = 249.5 Hz,^2^ *J*(^19^F – ^13^C) = 15.0 Hz,^3^ *J*(^19^F – ^13^C) = 4.6 Hz), 155.29 (NHC­(O)­O). ^**19**^ **F NMR** (376 MHz, DMSO-*d*_6_) δ: −124.85 (1F, dd,^1^ *J* = 12.3 Hz,^2^ *J* = 6.8 Hz), −134.57 (1F, dd,^1^ *J* = 26.9 Hz,^2^ *J* = 12.6 Hz), −150.67 (1F, dd,^1^ *J* = 27.2 Hz,^2^ *J* = 6.8 Hz). HRMS for C_21_H_32_F_3_N_3_O_6_S_2_ [(M+H)^+^]: calc. 544.1757, found 544.1772.

#### *tert*-Butyl­(2-((2-(cyclooctylamino)-6-(cyclopentylamino)-3,5-difluoro-4-sulfamoylphenyl) sulfonyl)­ethyl)­carbamate (24)

A mixture of compound **23** (81 mg, 0.15 mmol, 1 equiv), cyclopentylamine (31 μL, 0.31 mmol, 2 equiv) and Et_3_N (44 μL, 0.31 mmol, 2 equiv) in DMSO (1 mL) was stirred for 10 h at 75 °C. Afterward, the reaction mixture was washed with H_2_O (10 mL) and extracted with EtOAc (3 × 15 mL). The organic phase was dried using anhydrous Na_2_SO_4_ and evaporated under reduced pressure. The product was purified by column chromatography (silica, EtOAc/CHCl_3_ (1:3), *R*_F_ = 0.87). Yield: 59 mg (63%), as yellow oil. ^**1**^ **H NMR** (400 MHz, MeOD-*d*_4_) δ: 1.41 (9H, s, C­(CH_3_)_3_), 1.50 – 1.80 (18H, m, cyclooctyl and cyclopentyl), 1.87 – 2.03 (4H, m, cyclooctyl and cyclopentyl), 3.43 – 3.56 (4H, m, SO_2_CH_2_CH_2_ and SO_2_CH_2_CH_2_), 3.86 (1H, br. s), NHCH cyclooctyl), 4.92 (water overlapped with NHCH cyclopentyl, NHCH cyclooctyl, NHCH cyclopentyl, NHC­(O)O and SO_2_NH_2_). ^**13**^ **C NMR** (100 MHz, MeOD-*d*_4_) δ: 24.54 (cyclopentyl), 24.73 (cyclooctyl), 26.77 (cyclooctyl), 28.27 (cyclooctyl), 28.67 ((CH_3_)_3_CO), 34.15 (cyclooctyl), 35.22 (cyclopentyl), 35.44 (SO_2_CH_2_ CH_2_), 55.03 (SO_2_ CH_2_CH_2_), 57.26 (cyclooctyl CHNH, d, *J* = 11.7 Hz), 59.37 (cyclopentyl CHNH, d, *J* = 10.9 Hz), 113.48 (C4), 128.82 (C1, t, *J*(^19^F – ^13^C) = 20.6 Hz), 136.11 (C3 and C5, ddd,^1^ *J*(^19^F – ^13^C) = 51.4 Hz,^2^ *J*(^19^F – ^13^C) = 12.9 Hz, ^3^ *J*(^19^F – ^13^C) = 2.9 Hz), 141.31 (C2 and C6, ddd,^1^ *J*(^19^F – ^13^C) = 244.2 Hz,^2^ *J*(^19^F – ^13^C) = 28.4 Hz,^3^ *J*(^19^F – ^13^C) = 4.0 Hz), 157.84 (NHC­(O)­O). ^**19**^ **F NMR** (376 MHz, MeOD-*d*_4_) δ: −136.91 (1F, br. s), −137.05 (1F, br. s). HRMS for C_26_H_42_F_2_N_4_O_6_S [(M+H)^+^]: calc. 609.2587, found 609.2589.

#### 2,3,5,6-Tetrafluoro-4-(methylsulfonyl)­benzenesulfonamide (25)

A mixture of compound **2** (400 mg, 1.61 mmol, 1eq ), and sodium methanesulfinate (200 mg, 2 mmol, 1.3 equiv) in DMSO (2 mL) was stirred at room temperature for 24 h. The mixture was diluted with H_2_O (15 mL) and the precipitate was filtered. The crude product was purified by crystallization from H_2_O. Yield: 248 mg (50%), as white solid, Mp: 230 °C (decomposes). ^**1**^ **H NMR** (400 MHz, DMSO-*d*_6_) δ: 3.56 (3H, s, SO_2_CH_3_), 8.67 (2H, s, SO_2_NH_2_). ^**13**^ **C NMR** (100 MHz, DMSO-*d*_6_) δ: 45.41 (SO_2_CH_3_), 122.21 (C1, t, *J* (^19^F – ^13^C) = 15 Hz), 127.36 (C4, t, *J* (^19^F – ^13^C) = 15.4 Hz), 142.92 (C2 and C6, ddd,^1^ *J* (^19^F – ^13^C) = 251.4 Hz,^2^ *J* (^19^F – ^13^C) = 12.0 Hz,^3^ *J* (^19^F – ^13^C) = 6.2 Hz), 144.07 (C3 and C5, dd,^1^ *J* (^19^F – ^13^C) = 242.1 Hz,^2^ *J* (^19^F – ^13^C) = 6.2 Hz). ^**19**^ **F NMR** (376 MHz, DMSO-*d*_6_) δ: −136.40 – −136.57 (2F, m), −136.82 – −136.99 (2F, m). HRMS for C_7_H_5_F_4_NO_4_S_2_ [(M+H)^+^]: calc. 306.9596, did not ionize.

#### 2,6-Difluoro-3,5-bis­(((1*r*,4*R*)-4-hydroxycyclohexyl)­amino)-4-(methylsulfonyl)­benzenesulfonamide (26)

A mixture of compound **25** (110 mg, 0.36 mmol, 1 equiv), (1*r*,4*r*)-4-aminocyclohexan-1-ol (92 mg, 0.8 mmol, 2.2 equiv) and Et_3_N (102 μL, 0.7 mmol, 2 equiv) in DMSO (3 mL) was stirred for 12 h at 70 °C. Afterward, additional Et_3_N (102 μL, 0.7 mmol, 2 equiv) was added to the reaction mixture and the heating was continued for 6 h. The mixture was diluted with H_2_O (30 mL) and extracted with EtOAc (3 × 10 mL). The extract was dried over MgSO_4_ and concentrated under reduced pressure. The resulting yellow oil was subjected to gradient flash chromatography (silica, EtOAc/Hexane, gradient from 3:1 to 5:1). The purified product was then crystallized from a mixture of H_2_O/EtOH (2:1) to remove additional isomers that were difficult to purify with chromatography. Yield: 34 mg (18%), as greenish solid, Mp: 247 – 249 °C (decomposes). ^**1**^ **H NMR** (400 MHz, DMSO-*d*_6_) δ: 1.15 – 1.25 (8H, m, cyclohexanol), 1.82 (5H, br. s, cyclohexanol), 1.93 (5H, br. s, cyclohexanol), 3.37 (3H, s, SO_2_CH_3_), 3.42 – 3.47 (2H, m, NHCH), 4.60 (2H, d, *J* = 4.4 Hz, OH), 6.04 (2H, d, *J* = 7.92 Hz, NHCH), 8.1 (2H, s, SO_2_NH_2_). ^**13**^ **C NMR** (100 MHz, DMSO-*d*_6_) δ: 31.72 (cyclohexanol), 33.74 (cyclohexanol), 43.41 (SO_2_CH_3_), 54.45 – 54.69 (NHCH, m), 67.99 (CHOH), 114.86 (C4, t, *J*(^19^F – ^13^C) = 4.0 Hz), 127.05 (C1, t, *J*(^19^F – ^13^C) = 16.7 Hz), 134.13 (C3 and C5, dd,^1^ *J*(^19^F – ^13^C) = 11.2 Hz,^2^ *J*(^19^F – ^13^C) = 5.3 Hz), 140.13 (C2 and C6, dd,^1^ *J*(^19^F – ^13^C) = 245.4 Hz,^2^ *J*(^19^F – ^13^C) = 4.8 Hz). ^**19**^ **F NMR** (376 MHz, DMSO-*d*_6_) δ: −135.39 (2F, s). HRMS for C_19_H_29_F_2_N_3_O_6_S_2_ [(M+H)^+^]: calc. 498.1539, found 498.1555.

#### 3-(Cyclohexylamino)-2,6-difluoro-5-(((1*r*,4*r*)-4-hydroxycyclohexyl)­amino)-4-(methylsulfonyl)­benzenesulfonamide (27)

A mixture of compound **25** (90 mg, 0.29 mmol, 1 equiv), cyclohexanamine (34 μL, 0.29 mmol, 1 equiv) and Et_3_N (41 μL, 0.29 mmol, 1 equiv) in DMSO (3 mL) was stirred at room temperature for 2 h. Afterward, (1*r*,4*r*)-4-aminocyclohexan-1-ol (44 mg, 0.38 mmol, 1.4 equiv) and Et_3_N (53 μL, 0.38 mmol, 1.3 equiv) were added to the reaction mixture, which was then stirred for 50 h at 56 °C. The mixture was then diluted with H_2_O (30 mL) and extracted with EtOAc (3 × 10 mL). The extract was dried over MgSO_4_ and concentrated under reduced pressure. The resulting yellow oil was subjected to column chromatography (silica, EtOAc/Hexane, (2:1), *R*_F_ = 0.4). The purified product was then recrystallized from a mixture of H_2_O/EtOH (2:1) to remove additional isomers that were difficult to purify with chromatography. Yield: 26 mg (18%), as greenish solid, Mp: 193 – 194 °C. ^**1**^ **H NMR** (400 MHz, DMSO-*d*_6_) δ: 1.09–1.34 (8H, m, cyclohexyl), 1.50 – 1.59 (1H, m, cyclohexyl), 1.64 – 1.74 (2H, m, cyclohexyl), 1.77 – 1.98 (6H, m, cyclohexyl), 3.37 (3H, overlap with H_2_O signal, SO_2_CH_3_), 3.40 (2H, br. s (overlap with H_2_O), CHNH), 4.60 (1H, d, *J* = 4.4 Hz, CHOH), 6.04 (1H, d, *J* = 8.2 Hz, cyclohexyl CHNH), 6.13 (1H, d, *J* = 8.2 Hz, cyclohexan-4-ol CHNH), 8.1 (2H, s, SO_2_NH_2_). ^**13**^ **C NMR** (100 MHz, DMSO-*d*_6_) δ: 24.41 (cyclohexyl), 25.16 (cyclohexyl), 31.73 (cyclohexyl), 33.75 (cyclohexyl), 33.81 (cyclohexyl), 43.38 (SO_2_CH_3_), 54.58 (CHNH, d, *J* = 11 Hz), 54.71 (CHNH, d, *J* = 13.2 Hz), 67.99 (CHOH), 114.69 (C4, t,^1^ *J*(^19^F – ^13^C) = 4 Hz), 127.06 (C1, t,^1^ *J*(^19^F – ^13^C) = 16.7 Hz), 134.07 (C3 and C5, ddd,^1^ *J*(^19^F – ^13^C) = 13.2 Hz,^2^ *J*(^19^F – ^13^C) = 9.9 Hz,^3^ *J* (^19^F – ^13^C) = 3.1 Hz), 140.07 (C2 and C6, ddd,^1^ *J*(^19^F – ^13^C) = 243.5 Hz,^2^ *J*(^19^F – ^13^C) = 8 Hz, ^3^ *J*(^19^F – ^13^C) = 3.8 Hz). ^**19**^ **F NMR** (376 MHz, DMSO-*d*_6_) δ: −135.36 (1F, s), - 135.65 (1F, s). HRMS for C_19_H_29_F_2_N_3_O_5_S_2_ [(M+H)^+^]: calc. 482.1589, found 482.1602.

#### 3,5-Bis­(cyclohexylamino)-2,6-difluoro-4-(methylsulfonyl)­benzenesulfonamide (28)

A mixture of **25** (82 mg, 0.26 mmol, 1eq ), cyclohexanamine (65 μL, 0.56 mmol, 2 equiv) and Et_3_N (75 μL, 0.53 mmol, 2.1 equiv) in DMSO (3 mL) was stirred for 48 h at 56 °C. Afterward, cyclohexanamine (32 μL, 0.26 mmol, 1 equiv) and Et_3_N (37 μL, 0.26 mmol, 1 equiv) were added and heating was continued for 48h. The mixture was then diluted with H_2_O (30 mL) and extracted with EtOAc (3 × 10 mL). The extract was dried over MgSO_4_ and concentrated under reduced pressure. The resulting yellow oil was subjected to flash chromatography (silica, EtOAc/Hexane (3:2), *R*_F_ = 0.17). Yield: 49 mg (39%), as yellow solid, Mp: 91–92 °C. ^**1**^ **H NMR** (400 MHz, DMSO-*d*_6_) δ: 1.08 – 1.33 (8H, m, cyclohexyl), 1.48 – 1.60 (2H, m, cyclohexyl), 1.64 – 1.74 (4H, m, cyclohexyl), 1.84 – 1.97 (4H, m, cyclohexyl), 3.39 (3H, overlap with H_2_O signal, SO_2_CH_3_), 3.40 (2H, br. s (overlap with H_2_O), CHNH), 6.13 (2H, d, *J* = 7.9 Hz, cyclohexyl CHNH), 8.08 (2H, s, SO_2_NH_2_). ^**13**^ **C NMR** (100 MHz, DMSO-*d*_6_) δ: 24.42 (cyclohexyl), 25.17 (cyclohexyl), 33.83 (cyclohexyl), 43.37 (SO_2_CH_3_), 54.61 – 54.84 (cyclohexyl CHNH, m), 114.52 (C4, t, *J*(^19^F – ^13^C) = 4 Hz), 127.08 (C1, t, *J*(^19^F – ^13^C) = 16.7 Hz), 134.01 (C3 and C5, dd,^1^ *J*(^19^F – ^13^C) = 11 Hz,^2^ *J*(^19^F – ^13^C) = 5.5 Hz), 139.99 (C2 and C6, dd,^1^ *J*(^19^F – ^13^C) = 245.4 Hz,^2^ *J*(^19^F – ^13^C) = 4.8 Hz). ^**19**^ **F NMR** (376 MHz, DMSO-*d*_6_) δ: −135.60 (2F, s). HRMS for C_19_H_29_F_2_N_3_O_4_S_2_ [(M+H)^+^]: calc. 466.1640, found 466.1652.

#### 3-(Cyclooctylamino)-2,6-difluoro-5-((3-hydroxypropyl)­amino)-4-(methylsulfonyl)­benzenesulfonamide (29)

A mixture of compound **25** (90 mg, 0.29 mmol, 1eq ), cyclooctanamine (45 μL, 0.32 mmol, 1 equiv) and Et_3_N (45 μL, 0.32 mmol, 1 equiv) in DMSO (3 mL) was stirred for 1.5 h at room temperature. Afterward, 3-aminopropan-1-ol (32 μL, 0.42 mmol, 1.3 equiv) and Et_3_N (59 μL, 0.42 mmol, 1.3 equiv) were added to the reaction mixture, which was then heated for 24 h at 56 °C. Further 3-aminopropan-1-ol (32 μL, 0.42 mmol, 1.3 equiv) and Et_3_N (59 μL, 0.42 mmol, 1.3 equiv) were added to the mixture, and the reaction was heated for 27 h. The mixture was then diluted with H_2_O (40 mL) and extracted with EtOAc (3 × 10 mL). The extract was dried over MgSO_4_ and concentrated under reduced pressure. The resulting yellow oil was subjected to column chromatography (silica, EtOAc/Hexane (3:2), *R*_F_ = 0.2). Yield: 8 mg (5%), as a green oil. ^**1**^ **H NMR** (400 MHz, DMSO-*d*_6_) δ: 1.40 – 1.80 (16H, m, cyclooctyl and propanol), 3.37 (3H, overlap with H_2_O signal, SO_2_CH_3_), 3.40 (2H, br. s (overlap with H_2_O), CHNH), 3.50 (2H, t, *J* = 6.2 Hz, NHCH_2_CH_2_CH_2_OH), 3.71 (1H, br. s, cyclooctyl CHNH) 4.58 (1H, br. s, OH), 5.96 (1H, t, *J* = 4.8 Hz, NHCH_2_), 6.36 (1H, d, *J* = 8.2 Hz, NHCH), 8.08 (2H, s, SO_2_NH_2_). ^**13**^ **C NMR** (100 MHz, DMSO-*d*_6_) δ: 23.01 (cyclooctyl), 25.06 (cyclooctyl), 26.79 (cyclooctyl), 32.29 (cyclooctyl), 33.20 (OHCH_2_ CH_2_CH_2_NH), 43.40 (SO_2_CH_3_), 44.41 (NHCH_2_, d, *J* = 11.7 Hz), 55.62 (cyclooctyl CHNH, d, *J* = 11.7 Hz), 58.35 (CHOH), 114.55 (C4, t, *J*(^19^F – ^13^C) = 4 Hz), 127.22 (C1, t, *J*(^19^F – ^13^C) = 16.5 Hz), 134.00 (C3 or C5, dd,^1^ *J*(^19^F – ^13^C) = 13 Hz,^2^ *J*(^19^F – ^13^C) = 3.1 Hz), 135.13 (C3 or C5, dd,^1^ *J*(^19^F – ^13^C) = 12.8 Hz,^2^ *J*(^19^F – ^13^C) = 2.9 Hz), 140.15 (C2 and C6, dd,^1^ *J*(^19^F – ^13^C) = 245.4 Hz,^2^ *J*(^19^F – ^13^C) = 4.4 Hz). ^**19**^ **F NMR** (376 MHz, DMSO-*d*_6_) δ: −134.91 (1F, d, *J* = 8.2 Hz), - 136.25 (1F, s). HRMS for C_18_H_29_F_2_N_3_O_5_S_2_ [(M+H)^+^]: calc. 470.1589, found 470.1604.

### Protein Preparation

Production and purification of 12 recombinant human carbonic anhydrases (CAI, CAII, CAIII, CAIV, CAVB, CAVI, CAVII, CAIX, CAXII, CAXIII, and CAXIV) were prepared as previously described.^36^ CA VA was expressed in insect cells. The codon-optimized synthetic gene, encoding CAVA (40–305 amino acids), was cloned into the pFastBac-derived vector containing a C-terminal 10xHis-2xStrepII-tag using Ligation Independent Cloning (LIC), as described in ref 37. DNA construct was used for transposition into EMBACY Bacmid DNA according to Bac-to-Bac manufacturer procedures (Life Technologies). Bacmid DNA was isolated and transfected into Sf9 insect cells using cellfectin (Life Technologies) according to the manufacturer’s instructions. After 72 h of incubation at 28 °C, baculovirus was harvested (P0 stock). Virus was amplified by addition of 1.2 mL of P0 virus stock to 50 mL of 1 × 10^6^ Sf9 cells ml^–1^, grown in suspension. After 72 h at 28 °C, baculovirus was harvested (P1 stock). For CA VA expression in the large scale, 8 flasks of 500 mL Sf9 suspension culture at a density of 1 × 10^6^ cells ml^–1^ were infected with 1 mL of P1 virus per flask. Protein was expressed for 72 h at 28 °C and cells were harvested by centrifugation at 500 x g. Cell pellet was stored at −20 °C until further use. Cell pellet was resuspended in lysis buffer (25 mM Tris/HCl pH 8.0, 200 mM NaCl, 1 mM TCEP) supplemented with 5 mM imidazole. Cells were lysed by sonication and the debris was removed by centrifugation at 53,340 × g for 30 min at 4 °C. The clarified lysate was loaded onto 2.0 mL Chelating Sepharose Fast Flow (Cytiva) charged with nickel ions and beads were washed with 20 mL lysis buffer containing 25 mM imidazole. Proteins were eluted in lysis buffer complemented with 200 mM imidazole. Elution fractions were pooled and passed over 2.0 mL Strep-Tactin Superflow beads (IBA Lifesciences). Beads were washed with 20 mL lysis buffer and protein was eluted in the same buffer containing 2.5 mM d-Desthiobiotin (IBA Lifesciences). To remove the 10xhis-2xStrepII-tag, pooled elution fractions were incubated with GST-3C protease during overnight dialysis against lysis buffer at 4 °C. After reverse affinity purification using 1.0 mL Glutathione Sepharose 4 Fast Flow (Cytiva), the 10xhis-2xStrepII-tag was found to be efficiently cleaved off from CA VA. The flow-through fraction was concentrated to 1.0 mL using an Amicon ultrafiltration device and injected onto a Enrich SEC 650 10/300 column (Bio-Rad) connected to a NGC Chromatography system (Bio-Rad). The column was equilibrated with lysis buffer before running the protein sample. Peak fractions were concentrated to ∼4.9 mg/mL. Protein aliquots were snap-frozen in liquid nitrogen and stored in at -80 °C.

Concentrations of proteins were measured spectrophotometrically by UV absorption at 280 nm.

### Determination of Binding Affinity by the Fluorescence-Based Thermal Shift Assay (FTSA)

Experiments were performed in real-time PCR instrument QIAGEN Rotor-Gene with a blue channel used for 8-anilino-1-naphthalenesulfonate (ANS) excitation (365 nm) and detection (460 nm) or green channel for Glomelt dye excitation (468 nm) and detection (507 nm). Protein samples consisted of 5–10 μM CA isozyme containing 0–200 μM inhibitor (concentrations varying 1.5-fold or 2-fold), 50 μM ANS or 200 times diluted Glomelt dye. Samples were prepared in 50 mM sodium phosphate buffer (at pH 7.0) containing 100 mM sodium chloride. Protein–ligand solutions were heated from 25 to 99 °C by applying the heating rate of 1 °C/min. The binding of compounds **7**, **8a**, **8c**, **8h** and **8i** to CAIX at pH 5.0 in universal buffer, consisting of 50 mM sodium phosphate, 50 mM sodium acetate, 25 mM sodium borate, and 50 mM sodium chloride, was also measured to assess more accurate *K*_d,obs_ values for CAIX at pH 5.0 (weaker interactions at lower pH lead to a sigmoidal profile of the dose–response curve).

### Isothermal Titration Calorimetry (ITC)

ITC experiments were carried out at 37 °C on a MicroCal PEAQ ITC calorimeter. The cell was filled with 10 μM CAIX and the syringe with 100 μM compound **13**. Both protein and ligand solutions were made in 50 mM Tris buffer containing 100 mM NaCl with different pH (5.0, 7.0, 9.7, and 10.0). A typical experiment was made of 0.5 μL first injection followed by 20 × 2 μL injections of ligand, with a spacing of 180 s between injections, stirring of 800 r.p.m., and reference power of 5 μcals^–1^. The heat of ligand dilution was corrected from the baseline measured after protein saturation at the end of titration. Protein concentration was measured by UV absorption and calculated from the known molar absorption coefficient of CAIX (ε = 35075). ITC data were fit with the Origin software package.

### Compound Competition Assay on Live Cells

The assay was performed as previously described.^28^ Human cervical adenocarcinoma cells (HeLa) were seeded in 12-well plates and incubated for 3 days under hypoxia. After removing the media, 200 μL of 2-fold serially diluted compound **13** or **14** (12 concentrations, starting from 5120 nM) was mixed with 200 μL of 20 nM GZ19–32 solution in the FluoroBright medium (ThermoFisher). The obtained solutions were then applied to the cells grown in 12-well culture plates, starting from the lowest concentration. The plate was incubated at normoxia conditions for 20 min. The solution was removed and cells were washed 3 times for 1.5–3 min with 400 μL of PBS. Then 180 μL of TrypLe express enzyme (ThermoFisher) was added to each well. After 10 min incubation, 20 μL of Defined Trypsin Inhibitor solution (ThermoFisher) was added and cells were resuspended by pipetting. 150 μL of the suspension from each well were transferred to Thermo Scientific Nunc MicroWell 96-Well Optical-Bottom Plates for fluorescence and absorbance measurements. Fluorescence was measured at 485 nm excitation and 520 nm emission wavelengths on Synergy HTX, the BioTek plate reader. Absorbance was measured at 650 nm wavelength.

### Determination of Compound Sulfonamide Group p*K*_a_

Compound p*K*_a_ values were measured by obtaining UV–Vis spectra at 37 °C at different pH values (from 5.0 to 10.5) using a BMG Labtech CLARIOstarPlus plate reader spectrophotometer. Compounds were diluted to a constant concentration of 70–100 μM (depending on compound solubility and absorbance peak) in a universal buffer consisting of 50 mM sodium phosphate, 50 mM sodium acetate, 25 mM sodium borate, and 50 mM sodium chloride. The final DMSO concentration in the solution was 2% (v/v). The p*K*_a_ values were calculated by normalizing the absorbance and plotting it as a function of pH, then fitting it to the Henderson–Hasselbalch equation using the least-square method as described in ref 38.

### Intrinsic Binding Affinity

The observed CA-ligand dissociation constant (*K*_d,obs_) depends on the buffer pH. The intrinsic dissociation constant *K*_d,int_ is equal to the observed dissociation constant *K*_d,obs_ multiplied by the fractions of deprotonated inhibitor and protonated Zn bound water form of CA (eq 1).

$$K_{d,\mathrm{int}}=K_{d,obs}\times f_{RSO_{2}NH^{-}}f_{CAZnH_{2}O}$$ 1

The fractions of the deprotonated inhibitor and the Zn-bound water form of CA can be calculated if both p*K*_*a*_ values are known (eqs 2 and 3).

$$f_{RSO_{2}NH^{-}}=\frac{10^{pH-pK_{a\text{\_}sulf}}}{1+10^{pH-pK_{a\text{\_}sulf}}}$$ 2

$$f_{CAZnH_{2}O}=1-\frac{10^{pH-pK_{a\text{\_}CAZnH_{2}O}}}{1+10^{pH-pK_{a\text{\_}CAZnH_{2}O}}}$$ 3

### Crystallization and Structure Determination

Crystal structures of carbonic anhydrase and ligand complexes were obtained by the sitting drop technique and using soaking or cocrystallization methods.

For soaking technique (PDB ID: 9F2N, 9F2O, 9F3G, 9F30), the concentrated CAXII (25–35 mg/mL) was mixed with an equal volume of reservoir solution consisting of 0.1 M ammonium citrate (pH 7.0), 0.2 M ammonium sulfate, and 26% (w/v) PEG4000. Obtained crystals were soaked with reservoir solution supplemented by 1 mM ligand dissolved in DMSO. Cryo-protective solution (0.1 M ammonium citrate pH 7.23, 22% (w/v) PEG4000 and 20% (v/v) ethylene glycol) was applied to the crystals before data collection. All diffraction data were collected at EMBL beamline P13 at the PETRA III storage ring (DESY, Hamburg, Germany).

For cocrystallization (PDB ID: 9R30, 9R0L, 9R31, 9R0U), CAIX and CAXII (at 10 mg/mL) were mixed with the ligands (0.5 mM final concentration) and incubated overnight at 4 °C. While crystallization was performed at room temperature. Protein preparation and crystallization conditions of CAIX were previously described.^39^ CAXII isozyme was expressed and purified as described in ref 40. Solution of CAXII crystallization with compounds **10** and **13**: 0.15 M MgCl_2_, 0.1 M NaOAc (pH 5.5), 15% PEG 4000. Solution of CAXII crystallization with compound **14**: 0.25 M MgCl_2_, 0.1 M NaOAc (pH 5.5), 15% PEG 4000.

All data sets were processed using XDS.^41^ AIMLESS 0.7.4 was used for data scaling and other CCP4 tools v. 7.1.002 ^42^ were used for data processing. The structure was solved by molecular replacement with the help of MOLREP v.11.7.02.^43^ The model was refined by REFMAC v. 5.8.0258 ^44^ and inspected in COOT v.0.9.^45^ The inhibitor model was created and minimized using AVOGADRO v. 1.2.0.^46^

## Supplementary Material





## Notes

Raw FTSA data and dose–response curves (Figure S1–S23); CA IX selectivity table (Table S1), p*K*_a_ values of sulfonamide amino group (Figure S24); Observed and intrinsic affinity values of compound binding to CAIX (Table S2); ITC data of CAIX interaction with compound **13** (Figure S25); Crystal structures of CA XII complexes with ligands obtained by soaking (Table S3); Crystal structures of CAIX and CA XII complexes with ligands obtained by cocrystallization (Table S4); Images of the electron densities of five ligands in their soaked and/or cocrystallized structures (Figure S26–S30); ^1^H NMR, ^13^C NMR, ^19^F NMR and HRMS of all newly synthesized compounds (Figures S31–S128); UPLC spectra of compounds **13** and **14** (Figures S129–S130) (PDF)
SMILE strings of the synthesized compounds and dissociation constants (in nM units) of investigated sulfonamides to 12 catalytically active human CAs at 37 °C obtained by FTSA (CSV)

## Abbreviations Used

AcOH: acetic acid
ANS: 8-anilino-1-naphthalenesulfonate
CA: carbonic anhydrase
DCM: dichloromethane
DMSO: dimethyl sulfoxide
EtOAc: ethyl acetate
FTSA: fluorescent thermal shift assay
HeLa: human cervical adenocarcinoma cells
HRMS: high-resolution mass spectra
ITC: isothermal titration calorimetry
*K*_d,obs_: observed dissociation constant
MeCN: acetonitrile
Mp: melting point
PEG: polyethylene glycol
PG: proteoglycan-like
TFA: trifluoroacetic acid
THF: tetrahydrofuran
TLC: thin layer chromatography
*T*_m_: enzyme melting temperature
UPLC-MS: ultraperformance liquid chromatography–mass spectrometry
