# Supplementary material for: Di-meta-Substituted Fluorinated Benzenesulfonamides as Potent and Selective Anticancer Inhibitors of Carbonic Anhydrase IX and XII
Source: J Med Chem. 2025 Aug 20;68(17):18389–406. doi: 10.1021/acs.jmedchem.5c01142 (PMC12434658; doi:10.1021/acs.jmedchem.5c01142)

Supporting Information

Di-meta-substituted fluorinated benzenesulfonamides as potent and selective anticancer inhibitors of carbonic anhydrase IX and XII

Aivaras Vaškevičius[1], Mantas Žvirblis[1], Maija Kurtenoka[2], Janis Leitans[3], Elena Manakova[4], Vaida Paketurytė-Latvė[1], Agnė Kvietkauskaitė[1], Andris Kazaks[3], Vladislava Eimonta[3], Kamilė Čerepenkaitė[1], Justina Kazokaitė-Adomaitienė[1], Aurelija Mickevičiūtė[1], Vaida Juozapaitienė[1], Kaspars Tars[3], Saulius Gražulis[5], Jurgita Matulienė[1], Virginija Dudutienė[1], Kirill Shubin[2]*, Daumantas Matulis[1] and Asta Zubrienė[1]*

[1] Department of Biothermodynamics and Drug Design, Institute of Biotechnology, Life Sciences Center, Vilnius University, Saulėtekio 7, Vilnius LT-10257, Lithuania

[2] Department of Organic Chemistry, Latvian Institute of Organic Synthesis, Aizkraukles 21, Riga LV-1006, Latvia

[3] Latvian Biomedical Research and Study Centre, Ratsupites 1 k-1, 1067 Riga, Latvia

[4] Department of Protein-DNA Interactions, Institute of Biotechnology, Life Sciences Center, Vilnius University, Saulėtekio 7, Vilnius LT-10257, Lithuania

[5] Sector of Crystallography and Chemical Informatics, Institute of Biotechnology, Life Sciences Center, Vilnius University, Saulėtekio 7, 10257 Vilnius, Lithuania

Corresponding authors: asta.zubriene@bti.vu.lt (Asta Zubrienė) and kir101@osi.lv (Kirill Shubin)

Table of Contents

Figures S1-S23. FTSA data for synthesized compound binding to CA isozymes	S2
Table S1. Compound selectivity for CAIX is evaluated as the ratio of compound affinity towards off-target CA relative to CAIX isozyme	S72
Figure S24. pK_a values of sulfonamide amino group	S73
Table S2. Observed dissociation constant $K_{d,obs}$ measured at pH 7.0 and pH 5.0 for CAIX isozyme and intrinsic constant $K_{d,int}$ calculated from the $K_{d,obs}$ values determined at pH 7.0 and pH5.0	S75
Figure S25. ITC data of CAIX interaction with compound **13**	S77
Table S3. Crystal structures of CA XII complexes with ligands obtained by soaking	S77
Table S4. Crystal structures of CAIX and CA XII complexes with ligands obtained by cocrystalization	S78
Figures S26-S30. Electron density difference (Fo-Fc) maps for CAIX and CAXII in complexes with compounds	S79
Figures S31 – S128. Compound ^{1}H NMR, ^{13}C NMR, ^{19}F NMR and HRMS spectra	S85
Figures S129-S130. UPLC spectra of compounds **13** and **14**	S148

FTSA data for synthesized compound binding to CA isozymes

Figure S1. Compound 4c (MKV84) binding to CA isozymes

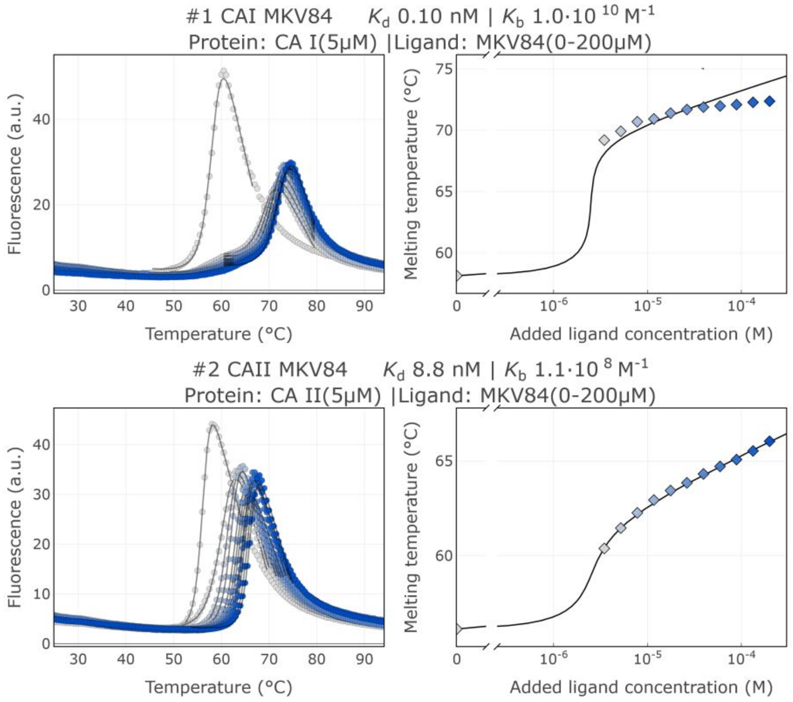

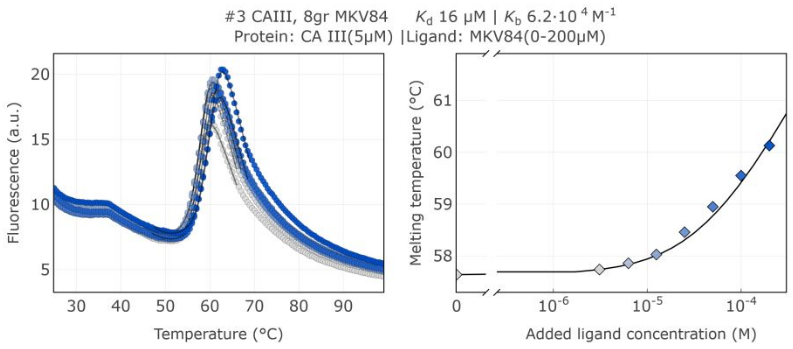
#3 CAIII, 8gr MKV84 K_d 16 µM | K_b 6.2·10 4 M-1
Protein: CA III(5µM) |Ligand: MKV84(0-200µM)
Fluorescence (a.u.)
Temperature (°C)
Melting temperature (°C)
Added ligand concentration (M)

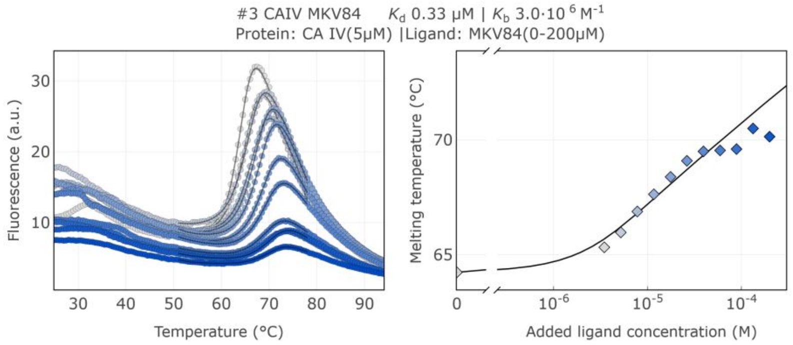
#3 CAIV MKV84 K_d 0.33 µM | K_b 3.0·10 6 M-1
Protein: CA IV(5µM) |Ligand: MKV84(0-200µM)
Fluorescence (a.u.)
Temperature (°C)
Melting temperature (°C)
Added ligand concentration (M)

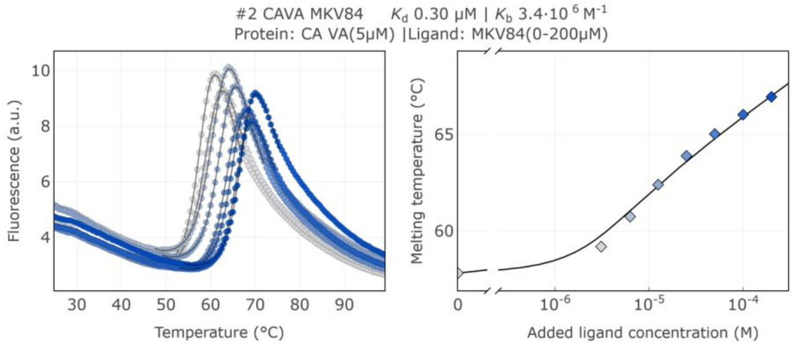
#2 CAVA MKV84 K_d 0.30 µM | K_b 3.4·10 6 M-1
Protein: CA VA(5µM) |Ligand: MKV84(0-200µM)
Fluorescence (a.u.)
Temperature (°C)
Melting temperature (°C)
Added ligand concentration (M)

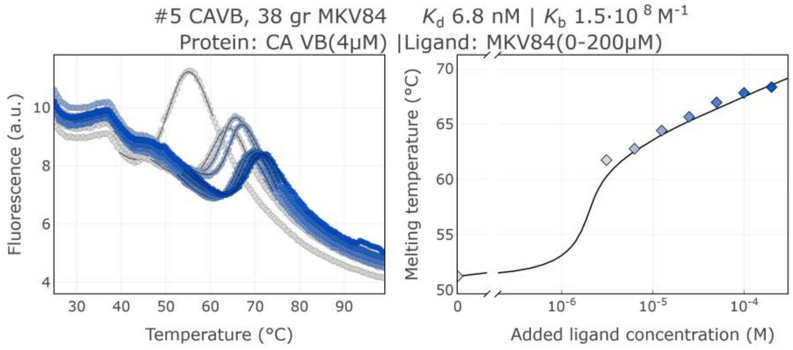
#5 CAVB, 38 gr MKV84 K_d 6.8 nM | K_b $1.5\cdot10^{8}$ M^{-1}
Protein: CA VB(4μM) |Ligand: MKV84(0-200μM)
Fluorescence (a.u.)
Temperature (°C)
Melting temperature (°C)
Added ligand concentration (M)

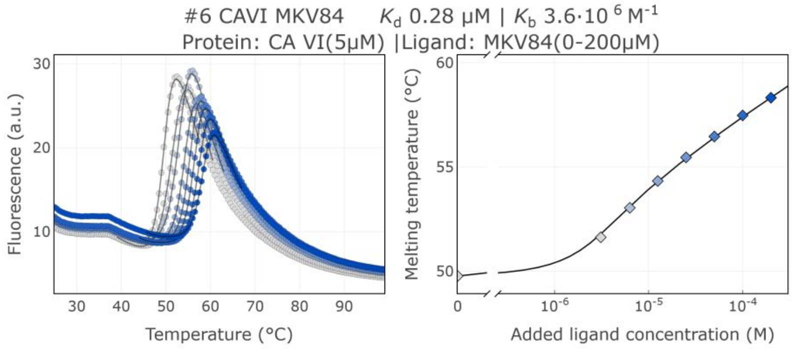
#6 CAVI MKV84 K_d 0.28 μM | K_b $3.6\cdot10^{6}$ M^{-1}
Protein: CA VI(5μM) |Ligand: MKV84(0-200μM)
Fluorescence (a.u.)
Temperature (°C)
Melting temperature (°C)
Added ligand concentration (M)

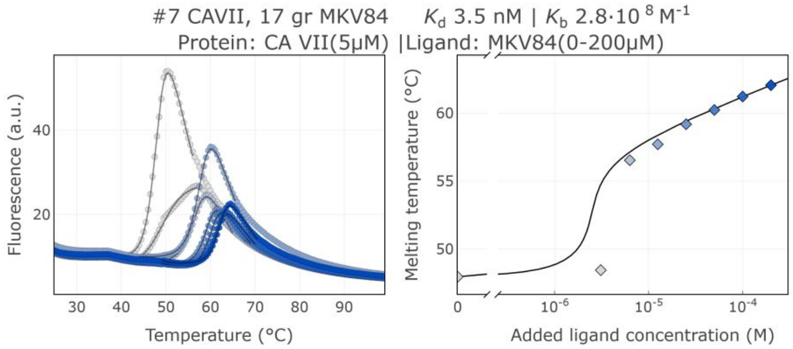
#7 CAVII, 17 gr MKV84 K_d 3.5 nM | K_b $2.8\cdot10^{8}$ M^{-1}
Protein: CA VII(5μM) |Ligand: MKV84(0-200μM)
Fluorescence (a.u.)
Temperature (°C)
Melting temperature (°C)
Added ligand concentration (M)

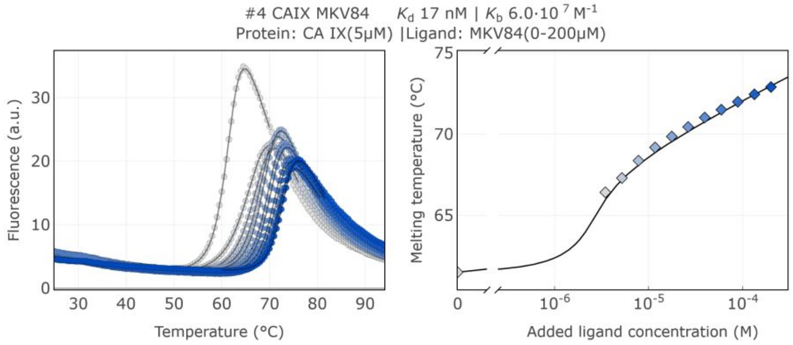
#4 CAIX MKV84 K_d 17 nM | K_b $6.0\cdot10^{7}$ M^{-1}
Protein: CA IX(5μM) |Ligand: MKV84(0-200μM)
Fluorescence (a.u.)
Temperature (°C)
Melting temperature (°C)
Added ligand concentration (M)

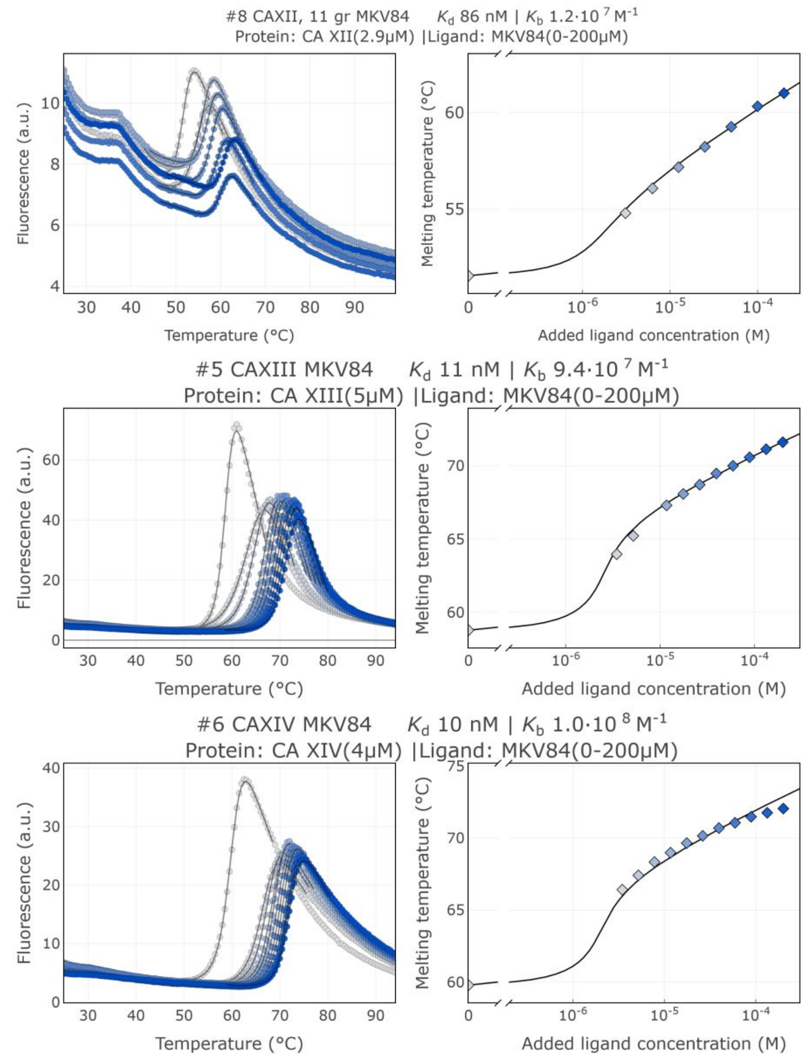
#8 CAXII, 11 gr MKV84 K_d 86 nM | K_b $1.2\cdot10^{7}$ M^{-1}
Protein: CA XII(2.9μM) |Ligand: MKV84(0-200μM)
Fluorescence (a.u.)
Temperature (°C)
Melting temperature (°C)
Added ligand concentration (M)
#5 CAXIII MKV84 K_d 11 nM | K_b $9.4\cdot10^{7}$ M^{-1}
Protein: CA XIII(5μM) |Ligand: MKV84(0-200μM)
Fluorescence (a.u.)
Temperature (°C)
Melting temperature (°C)
Added ligand concentration (M)
#6 CAXIV MKV84 K_d 10 nM | K_b $1.0\cdot10^{8}$ M^{-1}
Protein: CA XIV(4μM) |Ligand: MKV84(0-200μM)
Fluorescence (a.u.)
Temperature (°C)
Melting temperature (°C)
Added ligand concentration (M)

Figure S2. Compound 5c (MKV85) binding to CA isozymes

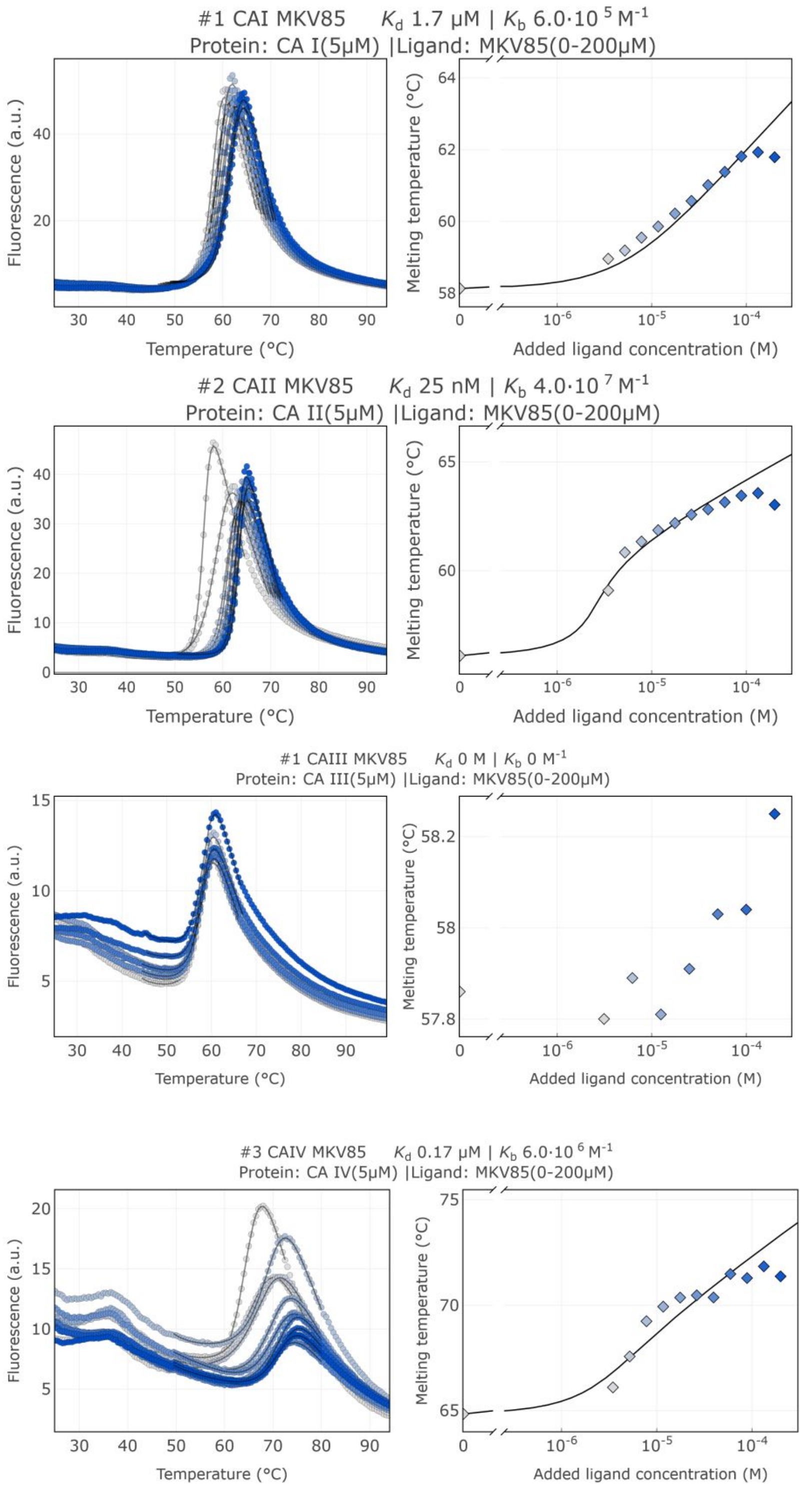

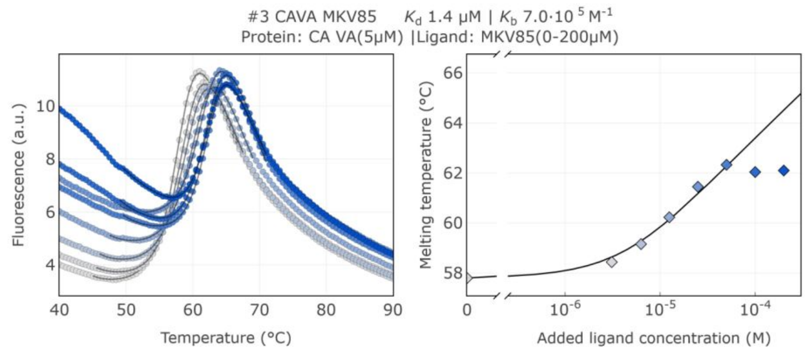
#3 CAVA MKV85 K_d 1.4 μM | K_b 7.0·10 5 M-1
Protein: CA VA(5μM) |Ligand: MKV85(0-200μM)
Fluorescence (a.u.)
Temperature (°C)
Melting temperature (°C)
Added ligand concentration (M)

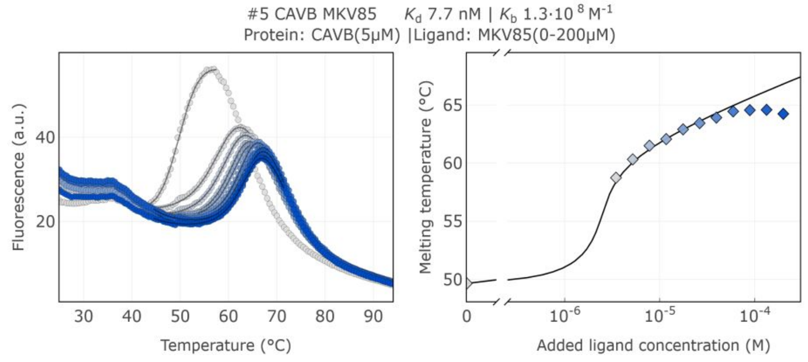
#5 CAVB MKV85 K_d 7.7 nM | K_b 1.3·10 8 M-1
Protein: CAVB(5μM) |Ligand: MKV85(0-200μM)
Fluorescence (a.u.)
Temperature (°C)
Melting temperature (°C)
Added ligand concentration (M)

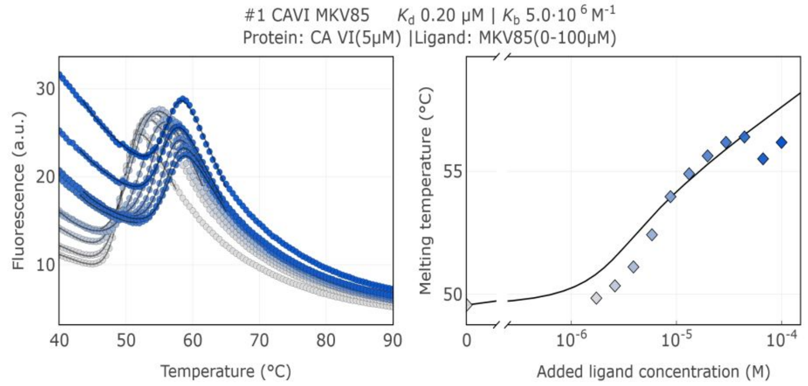
#1 CAVI MKV85 K_d 0.20 μM | K_b 5.0·10 6 M-1
Protein: CA VI(5μM) |Ligand: MKV85(0-100μM)
Fluorescence (a.u.)
Temperature (°C)
Melting temperature (°C)
Added ligand concentration (M)

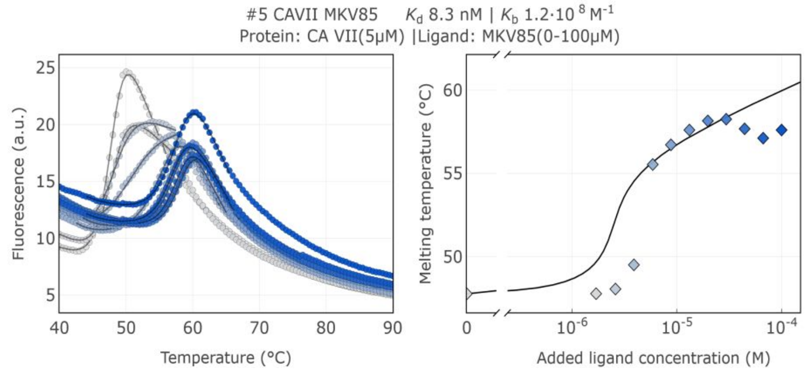
#5 CAVII MKV85 K_d 8.3 nM | K_b 1.2·10 8 M-1
Protein: CA VII(5μM) |Ligand: MKV85(0-100μM)
Fluorescence (a.u.)
Temperature (°C)
Melting temperature (°C)
Added ligand concentration (M)

#6 CAIX MKV85 K_d 0.020 nM | K_b $5.0\cdot10^{10}$ M^{-1}
Protein: CA IX(5μM) |Ligand: MKV85(0-200μM)

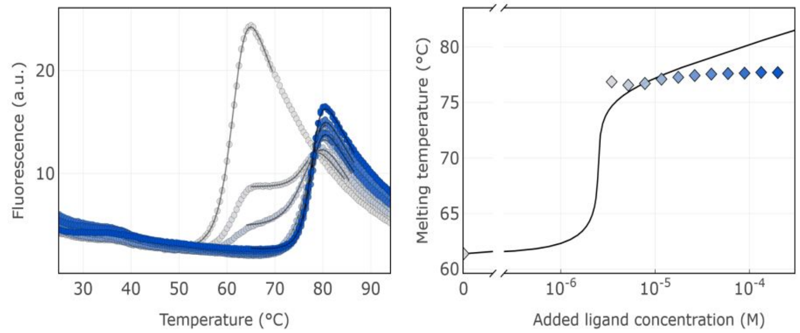

#3 CAXII MKV85 K_d 1.3 nM | K_b $8.0\cdot10^{8}$ M^{-1}
Protein: CA XII(4μM) |Ligand: MKV85(0-100μM)

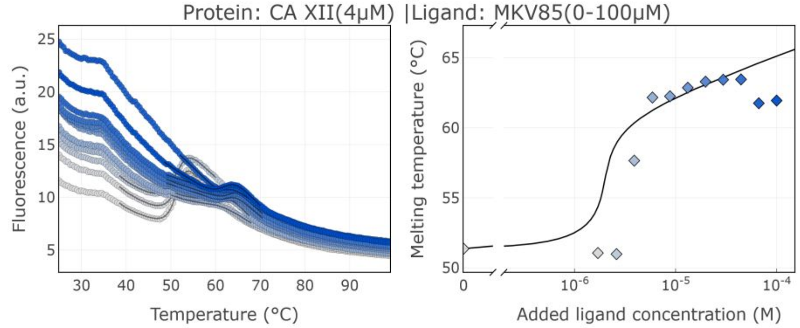

#4 CAXIII MKV85 K_d 5.0 nM | K_b $2.0\cdot10^{8}$ M^{-1}
Protein: CA XIII(4μM) |Ligand: MKV85(0-100μM)

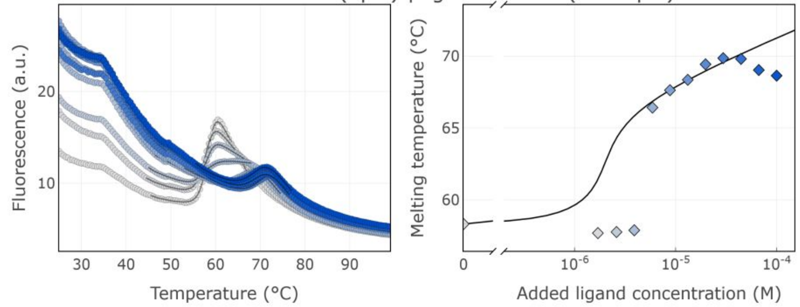

#5 CAXIV MKV85 K_d 5.0 nM | K_b $2.0\cdot10^{8}$ M^{-1}
Protein: CA XIV(4μM) |Ligand: MKV85(0-100μM)

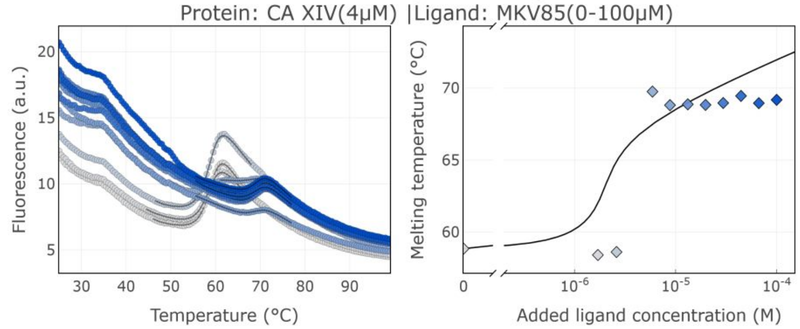

Figure S3. Compound 6 (MKV255) binding to CA isozymes

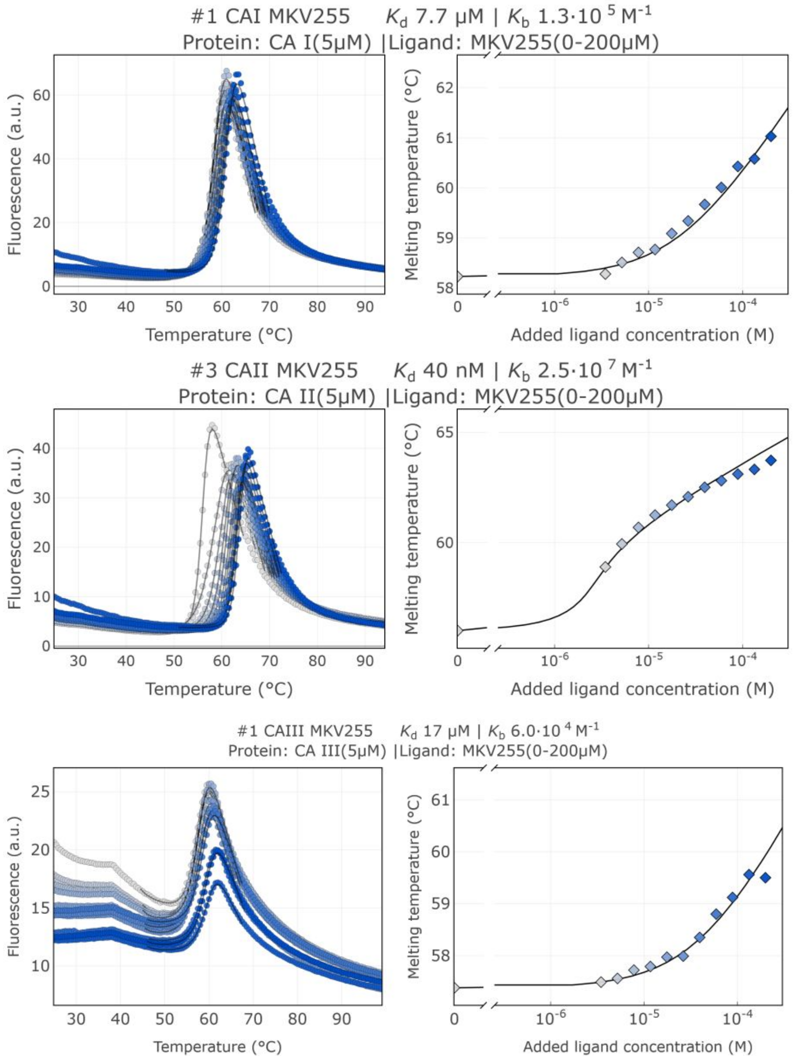

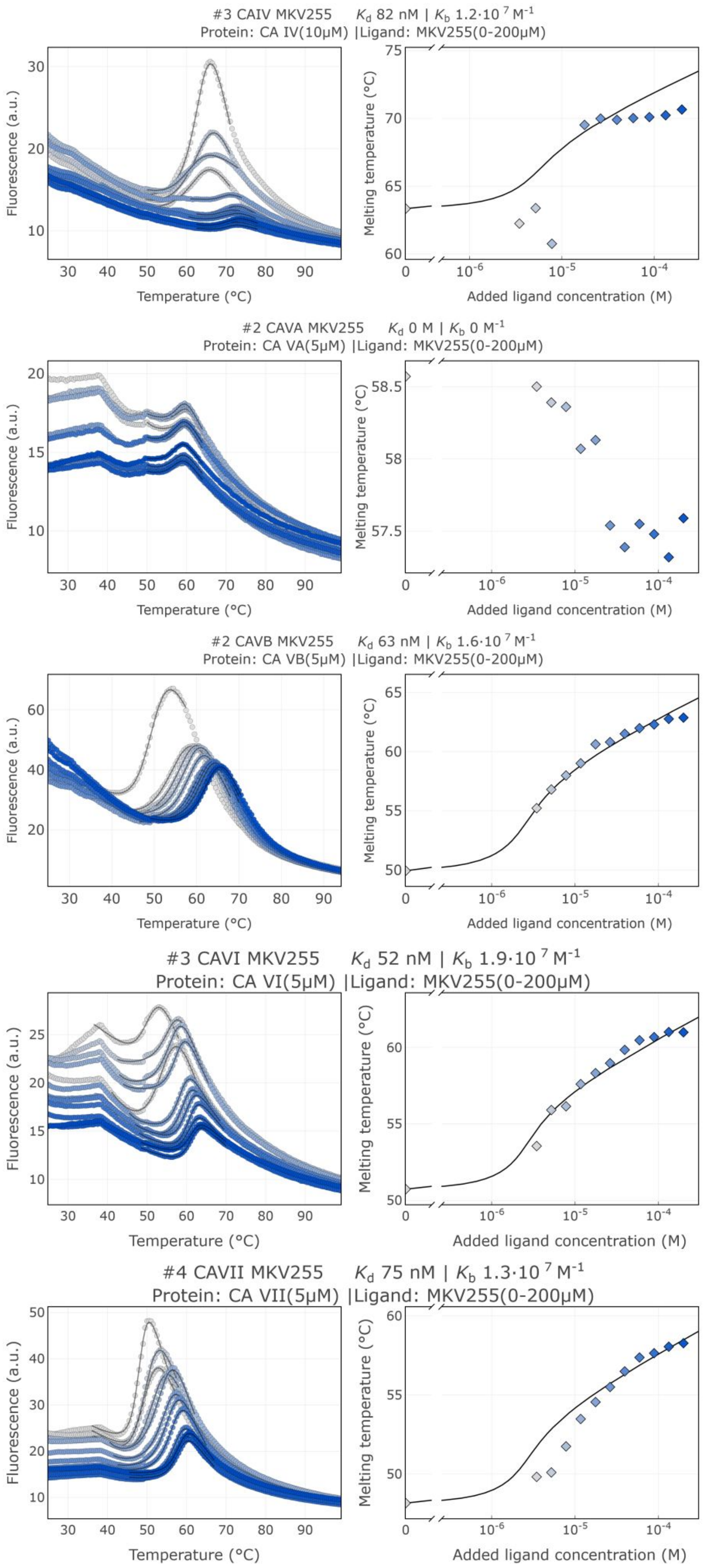
#3 CAIV MKV255 K_d 82 nM | K_b 1.2·10 ^{7} M^{-1}
Protein: CA IV(10μM) |Ligand: MKV255(0-200μM)
#2 CAVA MKV255 K_d 0 M | K_b 0 M^{-1}
Protein: CA VA(5μM) |Ligand: MKV255(0-200μM)
#2 CAVB MKV255 K_d 63 nM | K_b 1.6·10 ^{7} M^{-1}
Protein: CA VB(5μM) |Ligand: MKV255(0-200μM)
#3 CAVI MKV255 K_d 52 nM | K_b 1.9·10 ^{7} M^{-1}
Protein: CA VI(5μM) |Ligand: MKV255(0-200μM)
#4 CAVII MKV255 K_d 75 nM | K_b 1.3·10 ^{7} M^{-1}
Protein: CA VII(5μM) |Ligand: MKV255(0-200μM)
Fluorescence (a.u.)
Temperature (°C)
Melting temperature (°C)
Added ligand concentration (M)

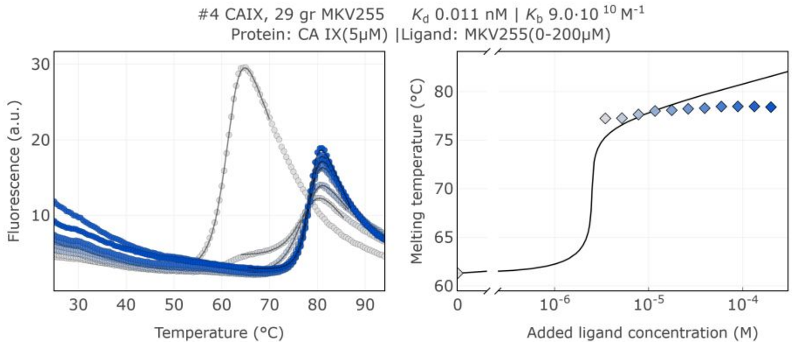
#4 CAIX, 29 gr MKV255 K_d 0.011 nM | K_b 9.0·10 ^{10} M^{-1}
Protein: CA IX(5μM) |Ligand: MKV255(0-200μM)
Fluorescence (a.u.)
Temperature (°C)
Melting temperature (°C)
Added ligand concentration (M)

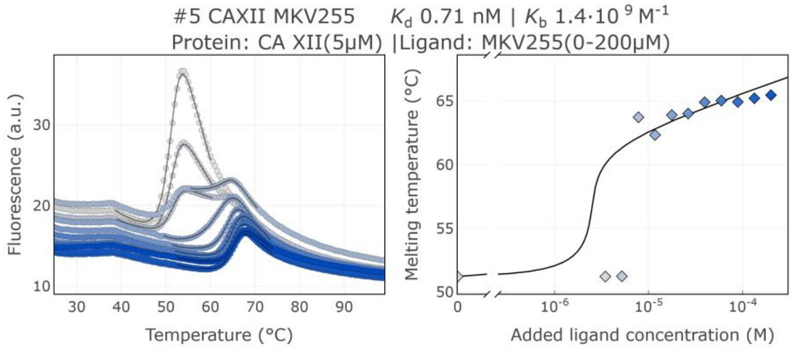
#5 CAXII MKV255 K_d 0.71 nM | K_b 1.4·10 ^{9} M^{-1}
Protein: CA XII(5μM) |Ligand: MKV255(0-200μM)
Fluorescence (a.u.)
Temperature (°C)
Melting temperature (°C)
Added ligand concentration (M)

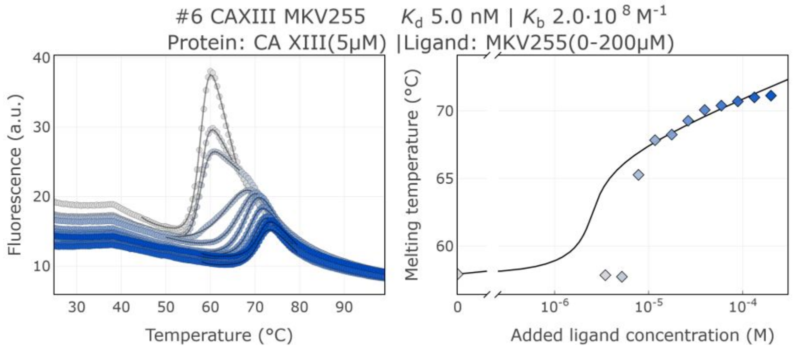
#6 CAXIII MKV255 K_d 5.0 nM | K_b 2.0·10 ^{8} M^{-1}
Protein: CA XIII(5μM) |Ligand: MKV255(0-200μM)
Fluorescence (a.u.)
Temperature (°C)
Melting temperature (°C)
Added ligand concentration (M)

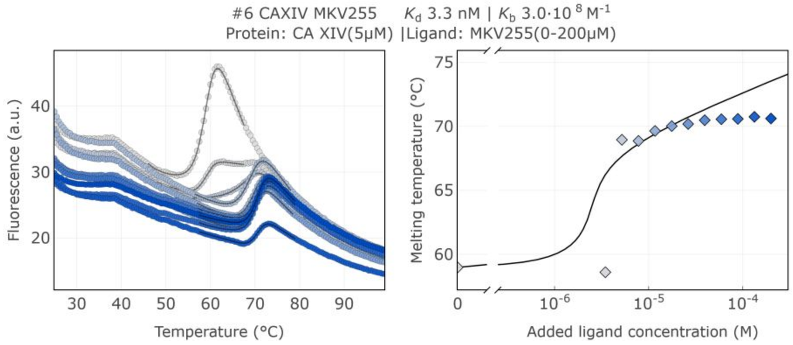
#6 CAXIV MKV255 K_d 3.3 nM | K_b 3.0·10 ^{8} M^{-1}
Protein: CA XIV(5μM) |Ligand: MKV255(0-200μM)
Fluorescence (a.u.)
Temperature (°C)
Melting temperature (°C)
Added ligand concentration (M)

Figure S4. Compound 7 (MKV256) binding to CA isozymes

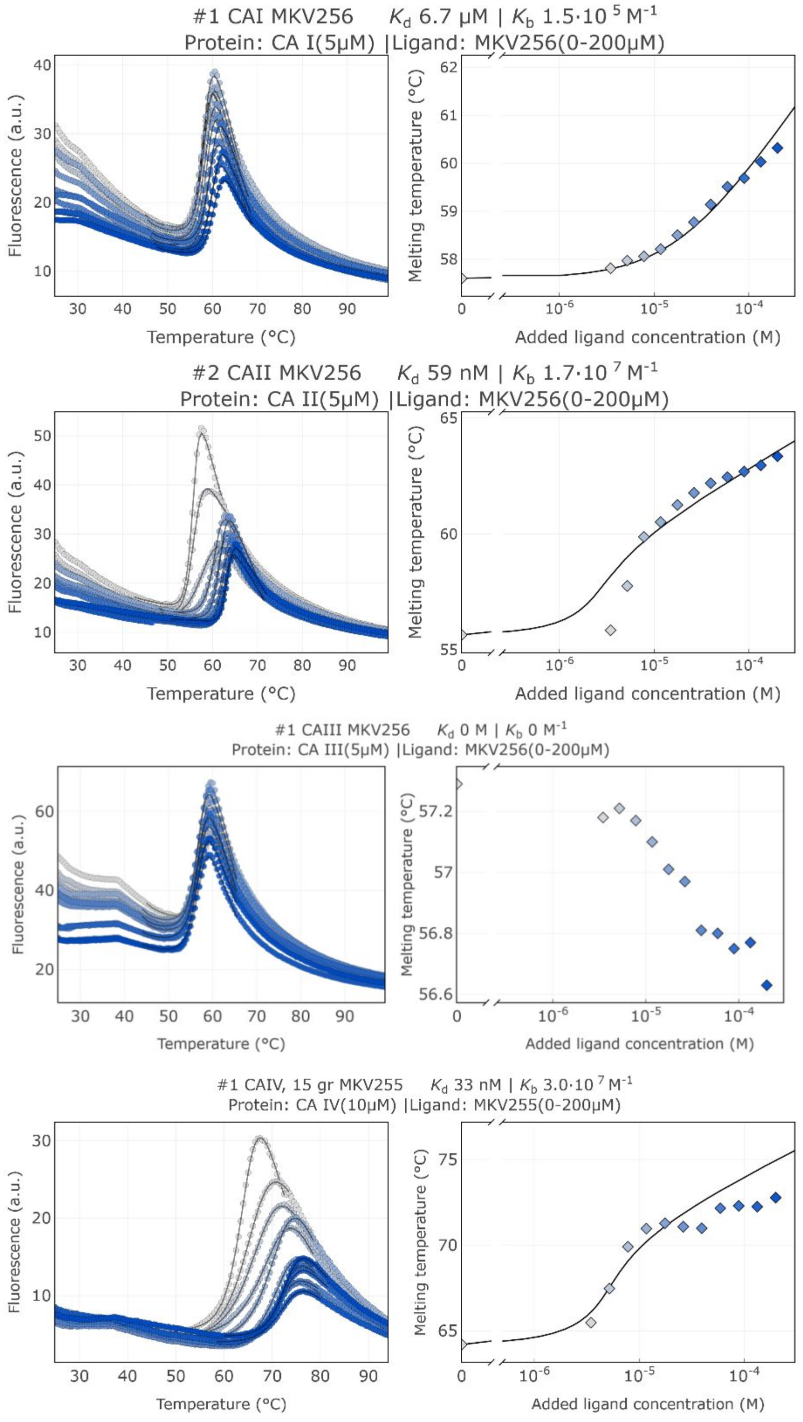

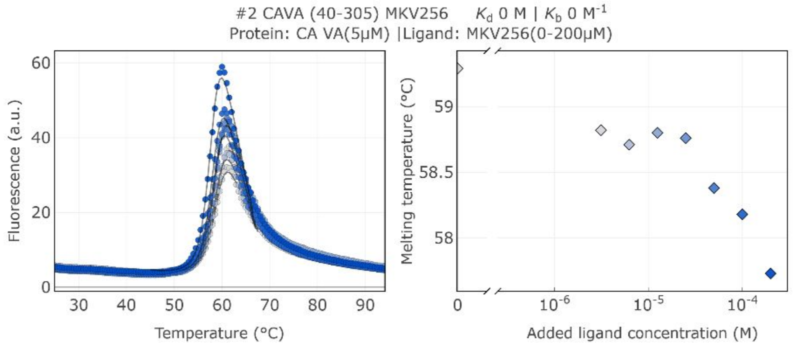
#2 CAVA (40-305) MKV256 K_d 0 M | K_b 0 M^{-1}
Protein: CA VA(5μM) |Ligand: MKV256(0-200μM)
Fluorescence (a.u.)
Temperature (°C)
Melting temperature (°C)
Added ligand concentration (M)
0
20
40
60
30
40
50
60
70
80
90
58
58.5
59
10^{-6}
10^{-5}
10^{-4}

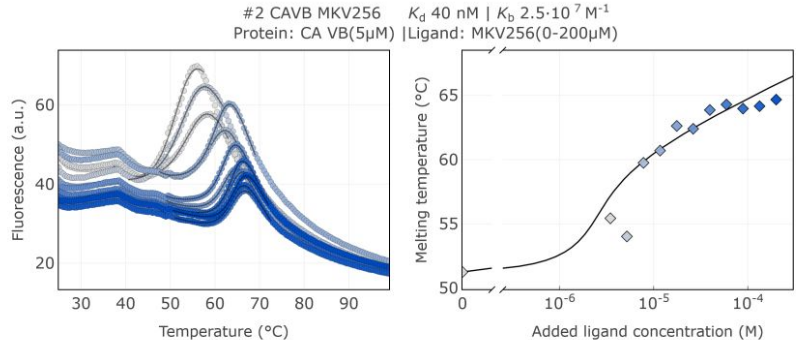
#2 CAVB MKV256 K_d 40 nM | K_b 2.5·10^{7} M^{-1}
Protein: CA VB(5μM) |Ligand: MKV256(0-200μM)
Fluorescence (a.u.)
Temperature (°C)
Melting temperature (°C)
Added ligand concentration (M)
20
40
60
30
40
50
60
70
80
90
50
55
60
65
0
10^{-6}
10^{-5}
10^{-4}

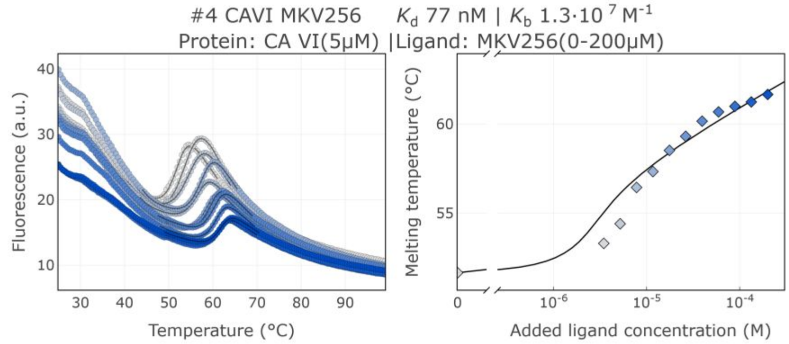
#4 CAVI MKV256 K_d 77 nM | K_b 1.3·10^{7} M^{-1}
Protein: CA VI(5μM) |Ligand: MKV256(0-200μM)
Fluorescence (a.u.)
Temperature (°C)
Melting temperature (°C)
Added ligand concentration (M)
10
20
30
40
30
40
50
60
70
80
90
55
60
0
10^{-6}
10^{-5}
10^{-4}

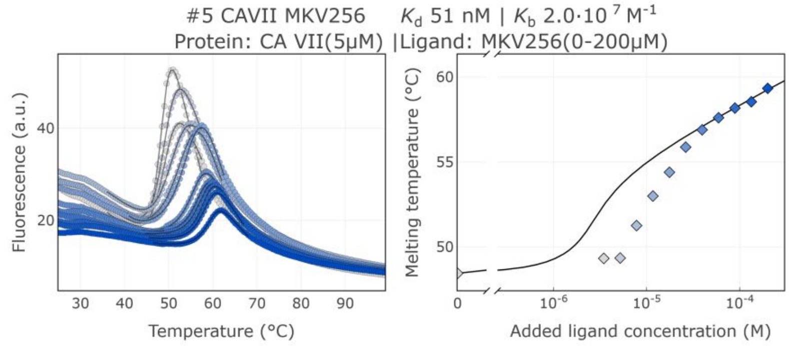
#5 CAVII MKV256 K_d 51 nM | K_b 2.0·10^{7} M^{-1}
Protein: CA VII(5μM) |Ligand: MKV256(0-200μM)
Fluorescence (a.u.)
Temperature (°C)
Melting temperature (°C)
Added ligand concentration (M)
20
40
30
40
50
60
70
80
90
50
55
60
0
10^{-6}
10^{-5}
10^{-4}

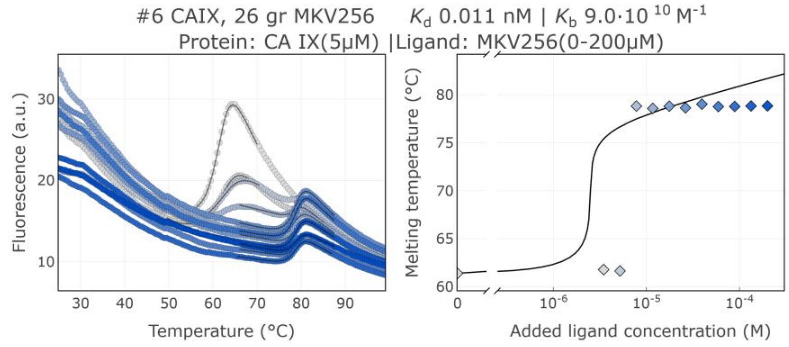
#6 CAIX, 26 gr MKV256 K_d 0.011 nM | K_b 9.0·10^{10} M^{-1}
Protein: CA IX(5μM) |Ligand: MKV256(0-200μM)
Fluorescence (a.u.)
Temperature (°C)
Melting temperature (°C)
Added ligand concentration (M)
10
20
30
30
40
50
60
70
80
90
60
65
70
75
80
0
10^{-6}
10^{-5}
10^{-4}

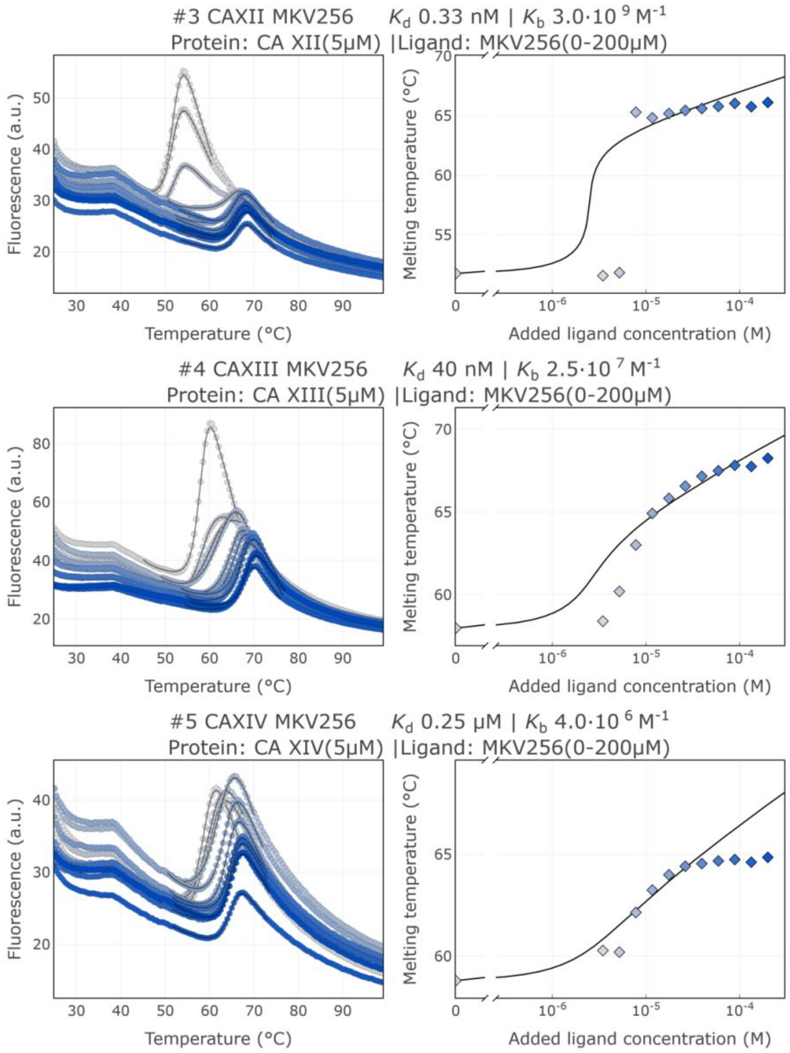
#3 CAXII MKV256 K_d 0.33 nM | K_b 3.0·10^{9} M^{-1}
Protein: CA XII(5μM) |Ligand: MKV256(0-200μM)
Fluorescence (a.u.)
Temperature (°C)
Melting temperature (°C)
Added ligand concentration (M)
#4 CAXIII MKV256 K_d 40 nM | K_b 2.5·10^{7} M^{-1}
Protein: CA XIII(5μM) |Ligand: MKV256(0-200μM)
Fluorescence (a.u.)
Temperature (°C)
Melting temperature (°C)
Added ligand concentration (M)
#5 CAXIV MKV256 K_d 0.25 μM | K_b 4.0·10^{6} M^{-1}
Protein: CA XIV(5μM) |Ligand: MKV256(0-200μM)
Fluorescence (a.u.)
Temperature (°C)
Melting temperature (°C)
Added ligand concentration (M)

Figure S5. Compound 8 (MKV450) binding to CA isozymes

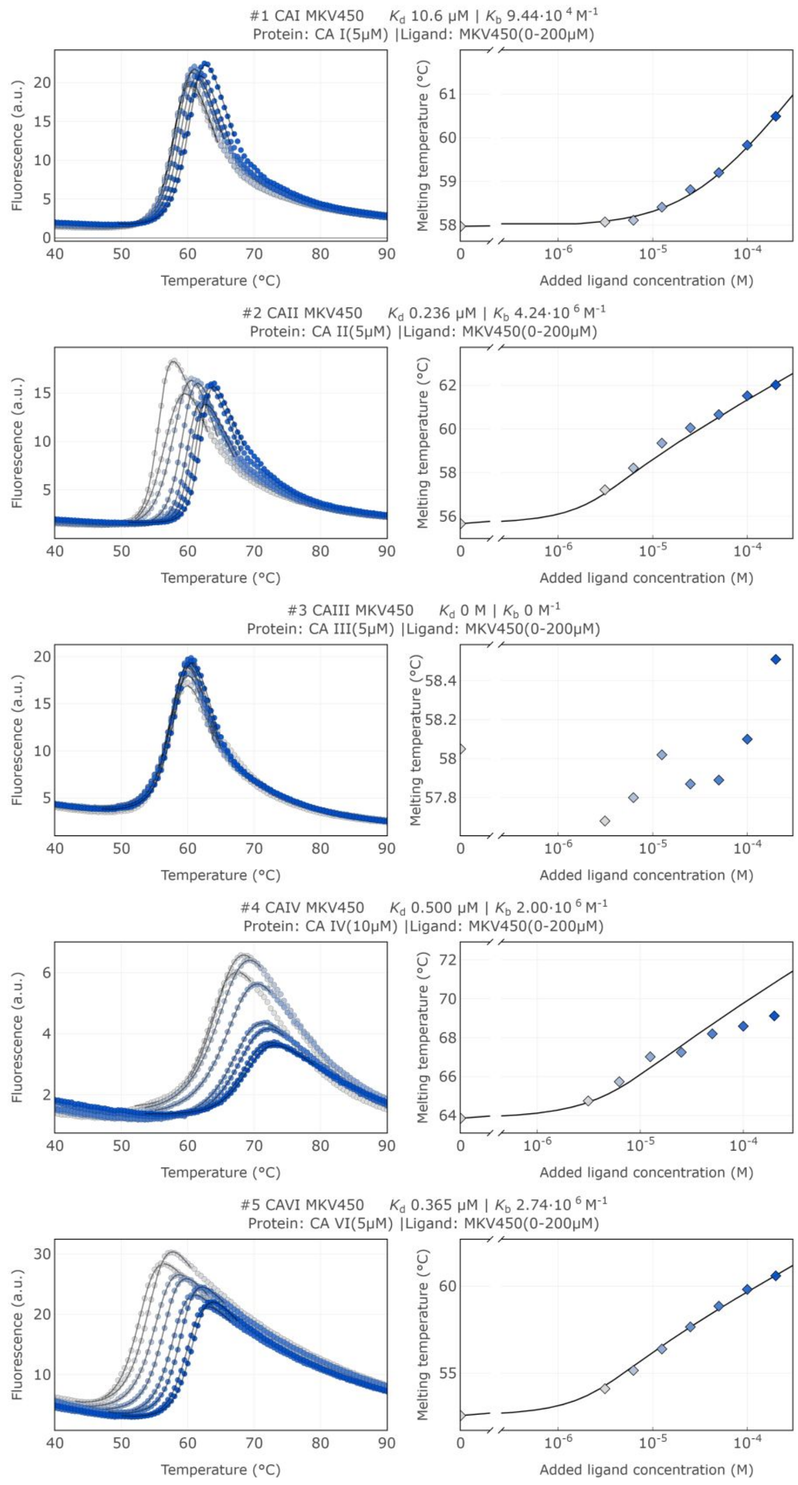

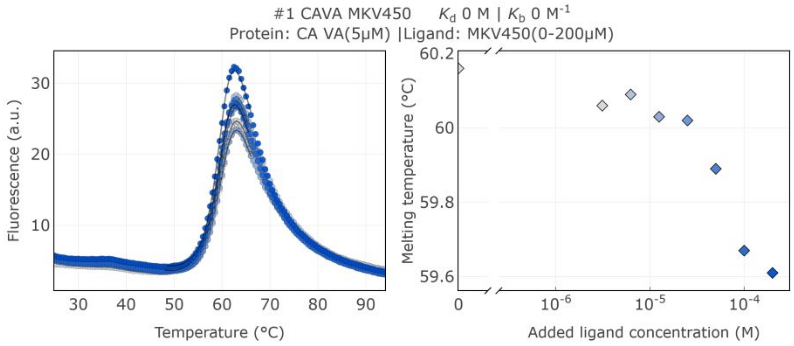
#1 CAVA MKV450 Kd 0 M | Kb 0 M-1
Protein: CA VA(5μM) |Ligand: MKV450(0-200μM)
Fluorescence (a.u.)
Temperature (°C)
Melting temperature (°C)
Added ligand concentration (M)

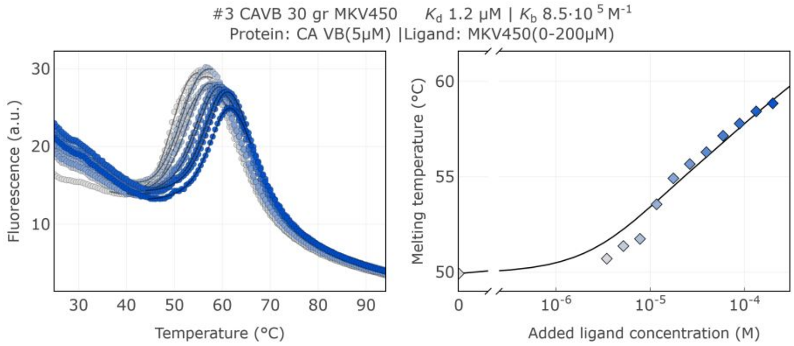
#3 CAVB 30 gr MKV450 Kd 1.2 μM | Kb 8.5·10 5 M-1
Protein: CA VB(5μM) |Ligand: MKV450(0-200μM)
Fluorescence (a.u.)
Temperature (°C)
Melting temperature (°C)
Added ligand concentration (M)

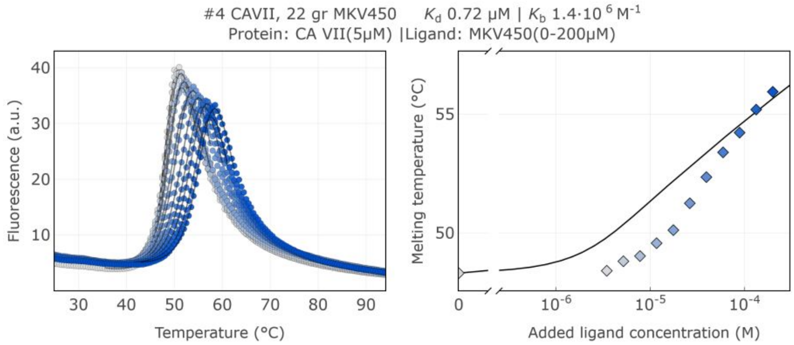
#4 CAVII, 22 gr MKV450 Kd 0.72 μM | Kb 1.4·10 6 M-1
Protein: CA VII(5μM) |Ligand: MKV450(0-200μM)
Fluorescence (a.u.)
Temperature (°C)
Melting temperature (°C)
Added ligand concentration (M)

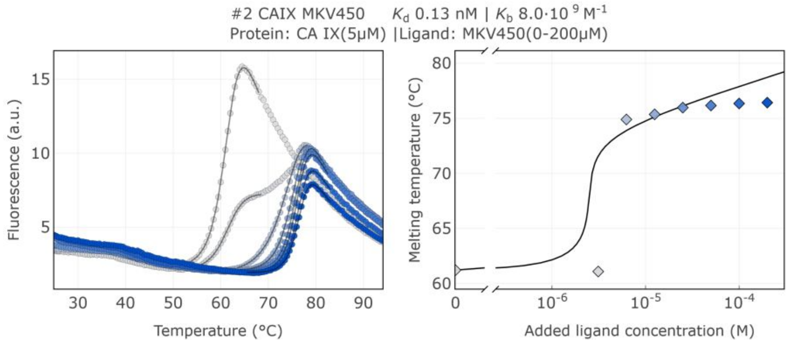
#2 CAIX MKV450 Kd 0.13 nM | Kb 8.0·10 9 M-1
Protein: CA IX(5μM) |Ligand: MKV450(0-200μM)
Fluorescence (a.u.)
Temperature (°C)
Melting temperature (°C)
Added ligand concentration (M)

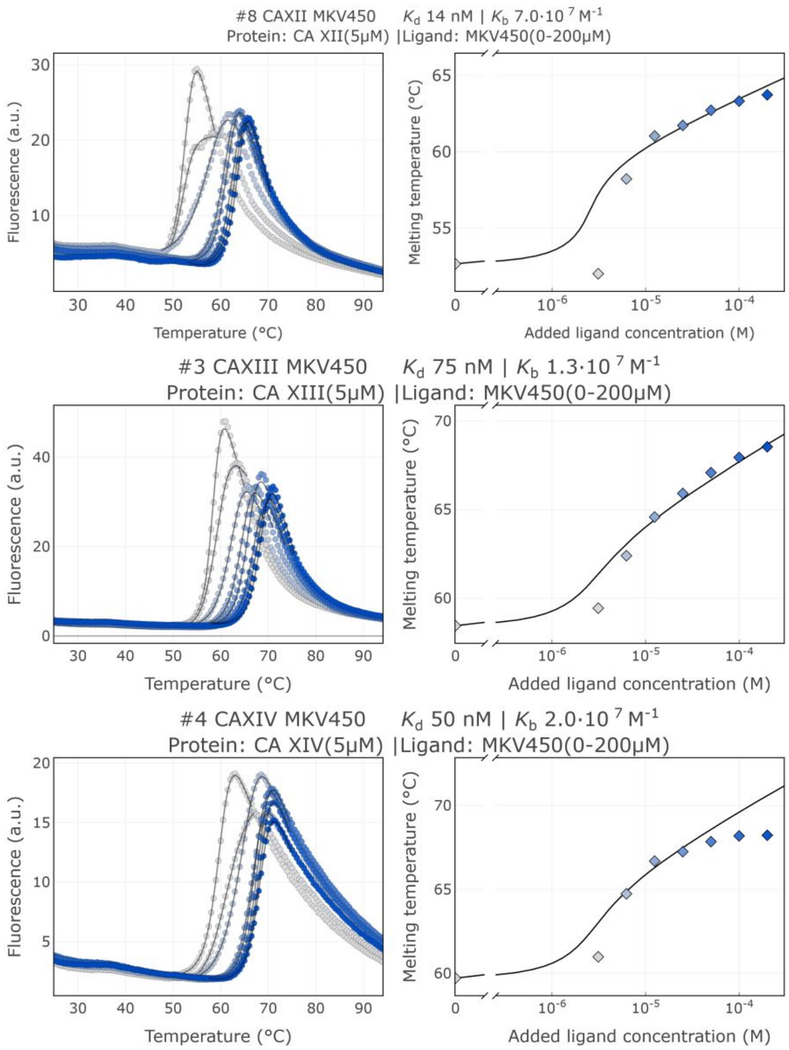
#8 CAXII MKV450 K_d 14 nM | K_b $7.0 \cdot 10^{7}$ M^{-1}
Protein: CA XII(5μM) |Ligand: MKV450(0-200μM)
Fluorescence (a.u.)
Temperature (°C)
Melting temperature (°C)
Added ligand concentration (M)
#3 CAXIII MKV450 K_d 75 nM | K_b $1.3 \cdot 10^{7}$ M^{-1}
Protein: CA XIII(5μM) |Ligand: MKV450(0-200μM)
Fluorescence (a.u.)
Temperature (°C)
Melting temperature (°C)
Added ligand concentration (M)
#4 CAXIV MKV450 K_d 50 nM | K_b $2.0 \cdot 10^{7}$ M^{-1}
Protein: CA XIV(5μM) |Ligand: MKV450(0-200μM)
Fluorescence (a.u.)
Temperature (°C)
Melting temperature (°C)
Added ligand concentration (M)

Figure S6. Compound 9 (MKV552) binding to CA isozymes

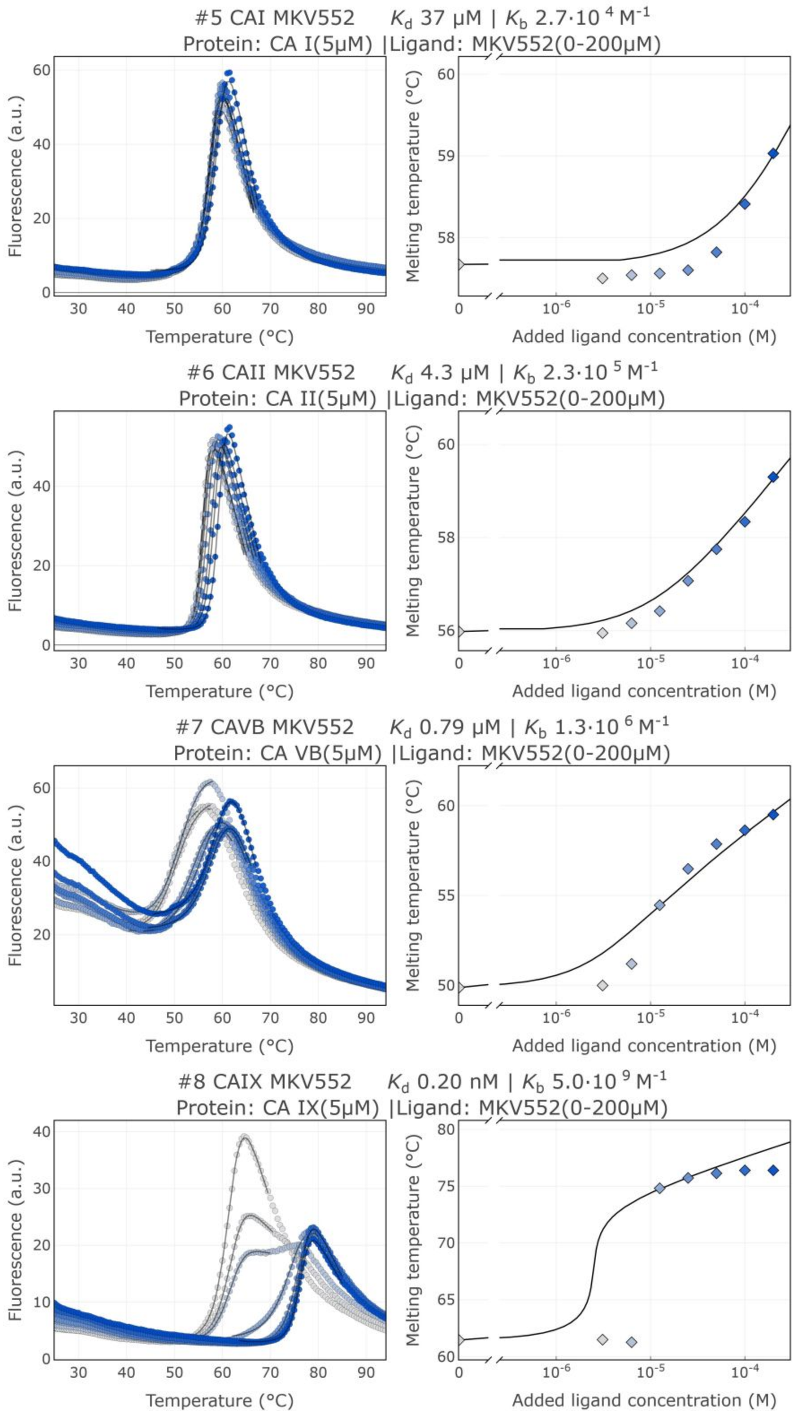

#4 CAVA (40-305) MKV552 K_d 0 M | K_b 0 M^{-1}
Protein: CA VA(5μM) |Ligand: MKV552(0-200μM)

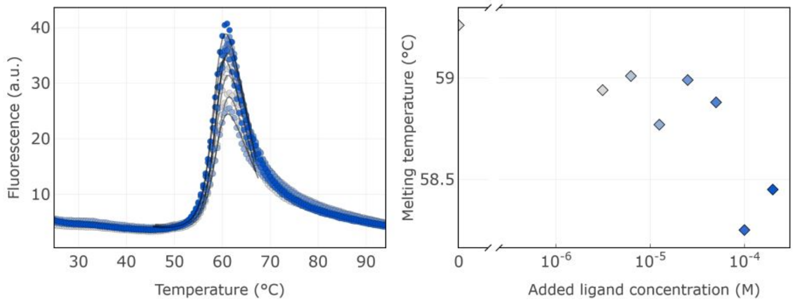

#5 CA VII MKV-552 K_d 4.1 μM | K_b 2.4·10 ^{5} M^{-1}
Protein: CA VII(5μM) |Ligand: MKV-552(0-200μM)

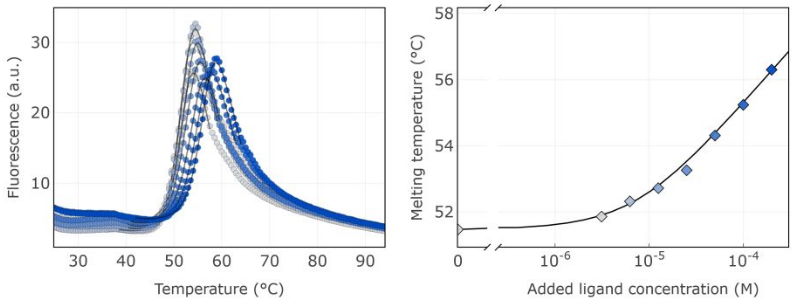

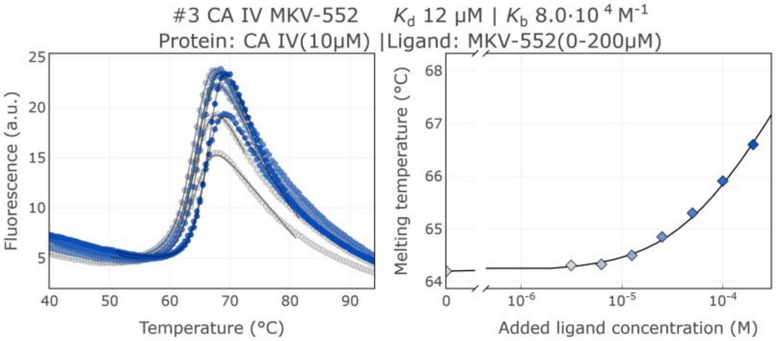
#3 CA IV MKV-552 K_d 12 μM | K_b $8.0\cdot10^4$ M^{-1}
Protein: CA IV(10μM) |Ligand: MKV-552(0-200μM)
Fluorescence (a.u.)
Temperature (°C)
Melting temperature (°C)
Added ligand concentration (M)

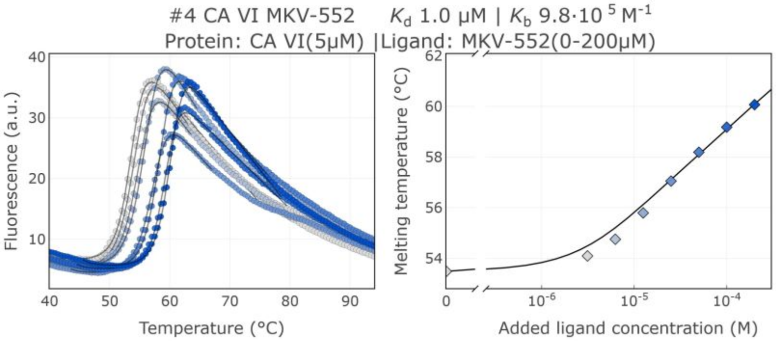
#4 CA VI MKV-552 K_d 1.0 μM | K_b $9.8\cdot10^5$ M^{-1}
Protein: CA VI(5μM) |Ligand: MKV-552(0-200μM)
Fluorescence (a.u.)
Temperature (°C)
Melting temperature (°C)
Added ligand concentration (M)

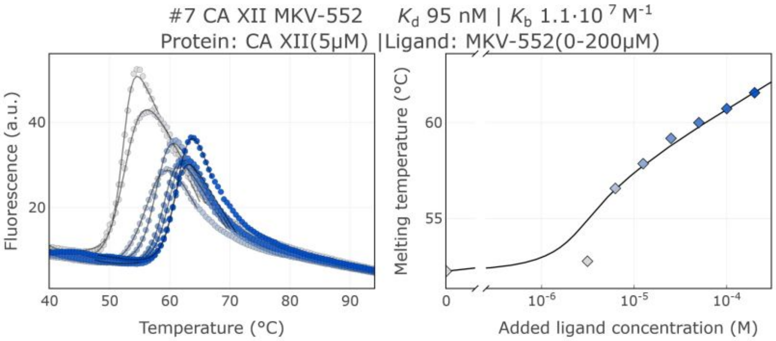
#7 CA XII MKV-552 K_d 95 nM | K_b $1.1\cdot10^7$ M^{-1}
Protein: CA XII(5μM) |Ligand: MKV-552(0-200μM)
Fluorescence (a.u.)
Temperature (°C)
Melting temperature (°C)
Added ligand concentration (M)

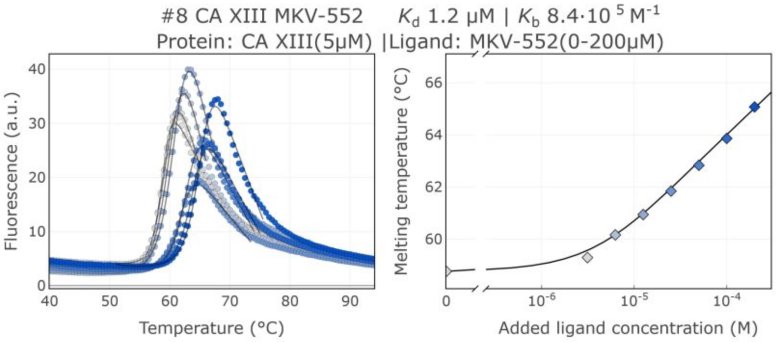
#8 CA XIII MKV-552 K_d 1.2 μM | K_b $8.4\cdot10^5$ M^{-1}
Protein: CA XIII(5μM) |Ligand: MKV-552(0-200μM)
Fluorescence (a.u.)
Temperature (°C)
Melting temperature (°C)
Added ligand concentration (M)

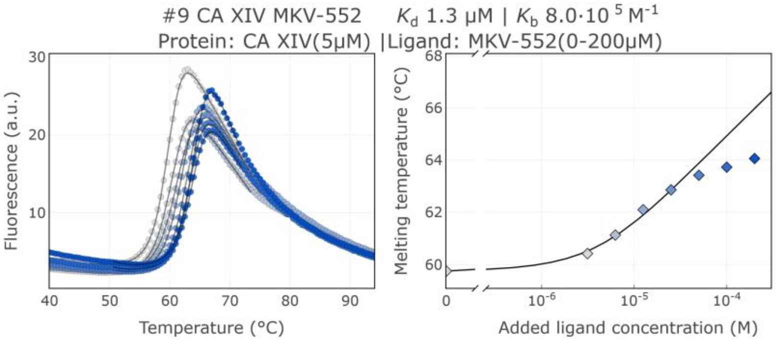
#9 CA XIV MKV-552 K_d 1.3 μM | K_b $8.0\cdot10^5$ M^{-1}
Protein: CA XIV(5μM) |Ligand: MKV-552(0-200μM)
Fluorescence (a.u.)
Temperature (°C)
Melting temperature (°C)
Added ligand concentration (M)

Figure S7. Compound 10 (MKV263) binding to CA isozymes

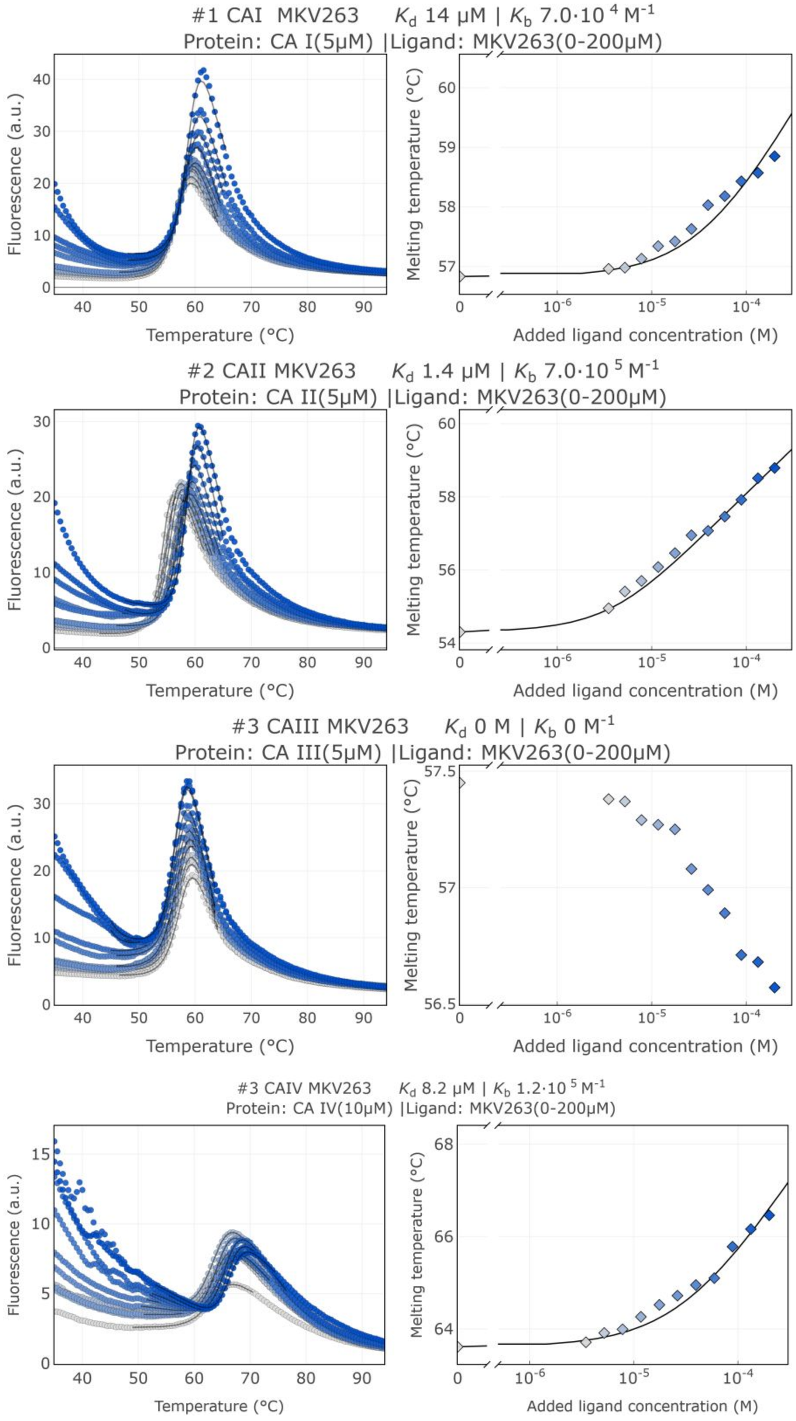

#1 CAVA MKV263 K_d 0.20 mM | K_b $5.0\cdot10^{3}$ M^{-1}
Protein: CA VA(5μM) |Ligand: MKV263(0-200μM)

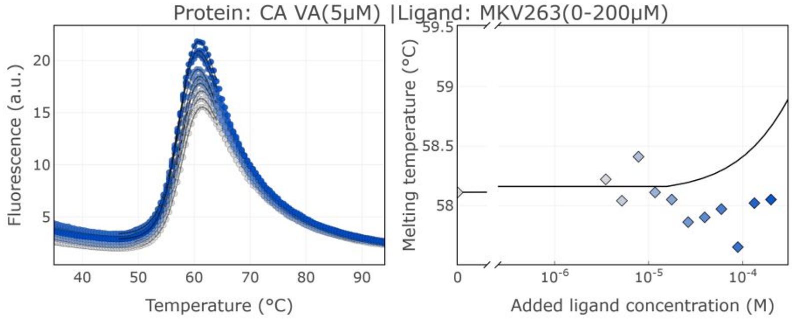

#2 CAVB MKV263 K_d 4.0 μM | K_b $2.5\cdot10^{5}$ M^{-1}
Protein: CA VB(5μM) |Ligand: MKV263(0-200μM)

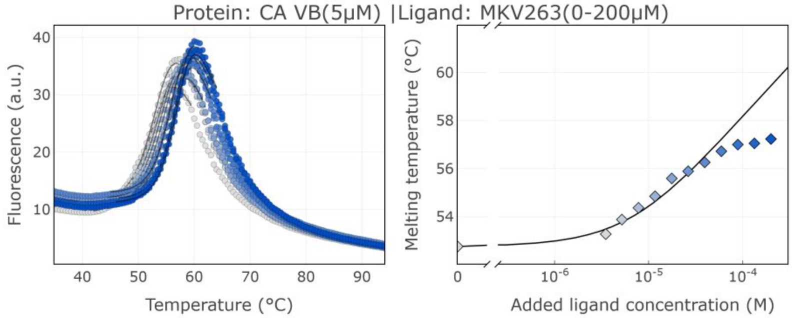

#3 CAVII MKV263 K_d 30 μM | K_b $3.4\cdot10^{4}$ M^{-1}
Protein: CA VII(5μM) |Ligand: MKV263(0-200μM)

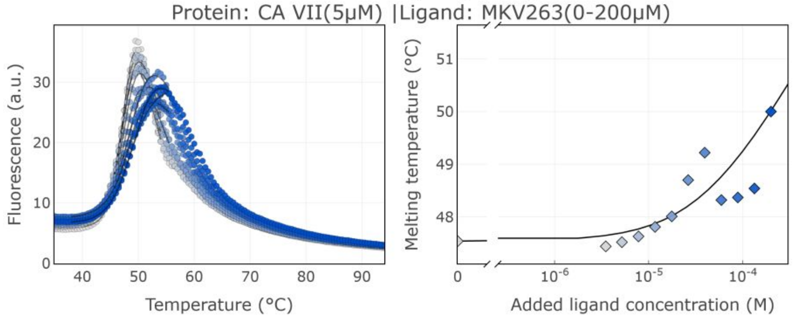

#4 CAVI MKV263 K_d 13 nM | K_b $7.9\cdot10^{7}$ M^{-1}
Protein: CA VI(5μM) |Ligand: MKV263(0-200μM)

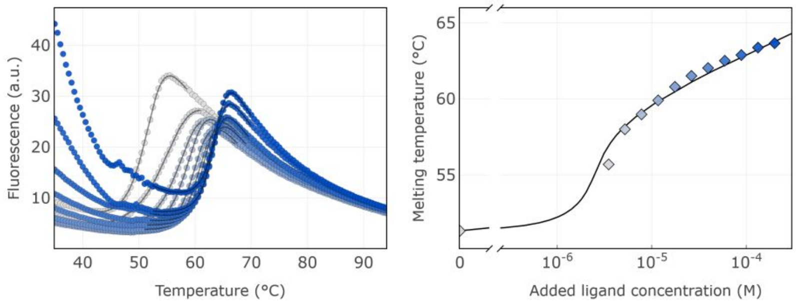

#5 CAIX MKV263 K_d 0.50 nM | K_b $2.0\cdot10^{9}$ M^{-1}
Protein: CA IX(5μM) |Ligand: MKV263(0-200μM)

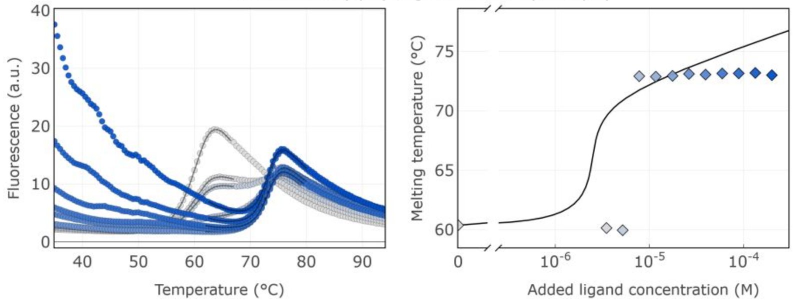

#6 CAXIII MKV263 K_d 1.2 μM | K_b $8.3 \cdot 10^{5}$ M^{-1}
Protein: CA XIII(5μM) |Ligand: MKV263(0-200μM)

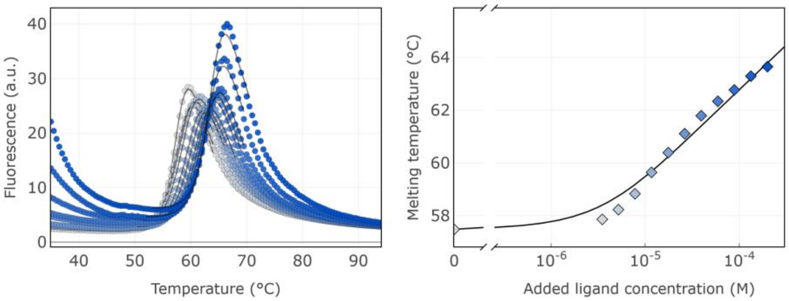

#4 CAXII MKV263 K_d 35 nM | K_b $2.8 \cdot 10^{7}$ M^{-1}
Protein: CA XII(5μM) |Ligand: MKV263(0-200μM)

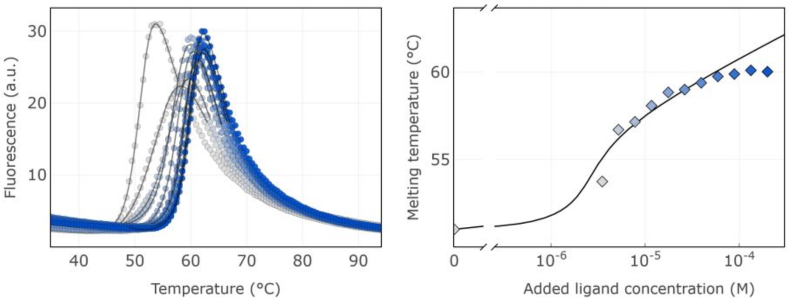

#5 CAXIV MKV263 K_d 25 μM | K_b $4.0 \cdot 10^{4}$ M^{-1}
Protein: CA XIV(5μM) |Ligand: MKV263(0-200μM)

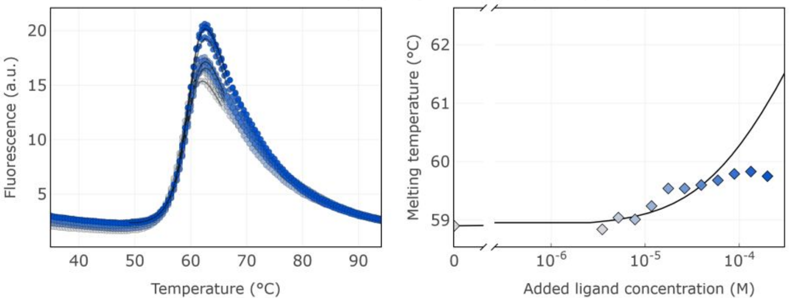

Figure S8. Compound 11 (MKV264) binding to CA isozymes

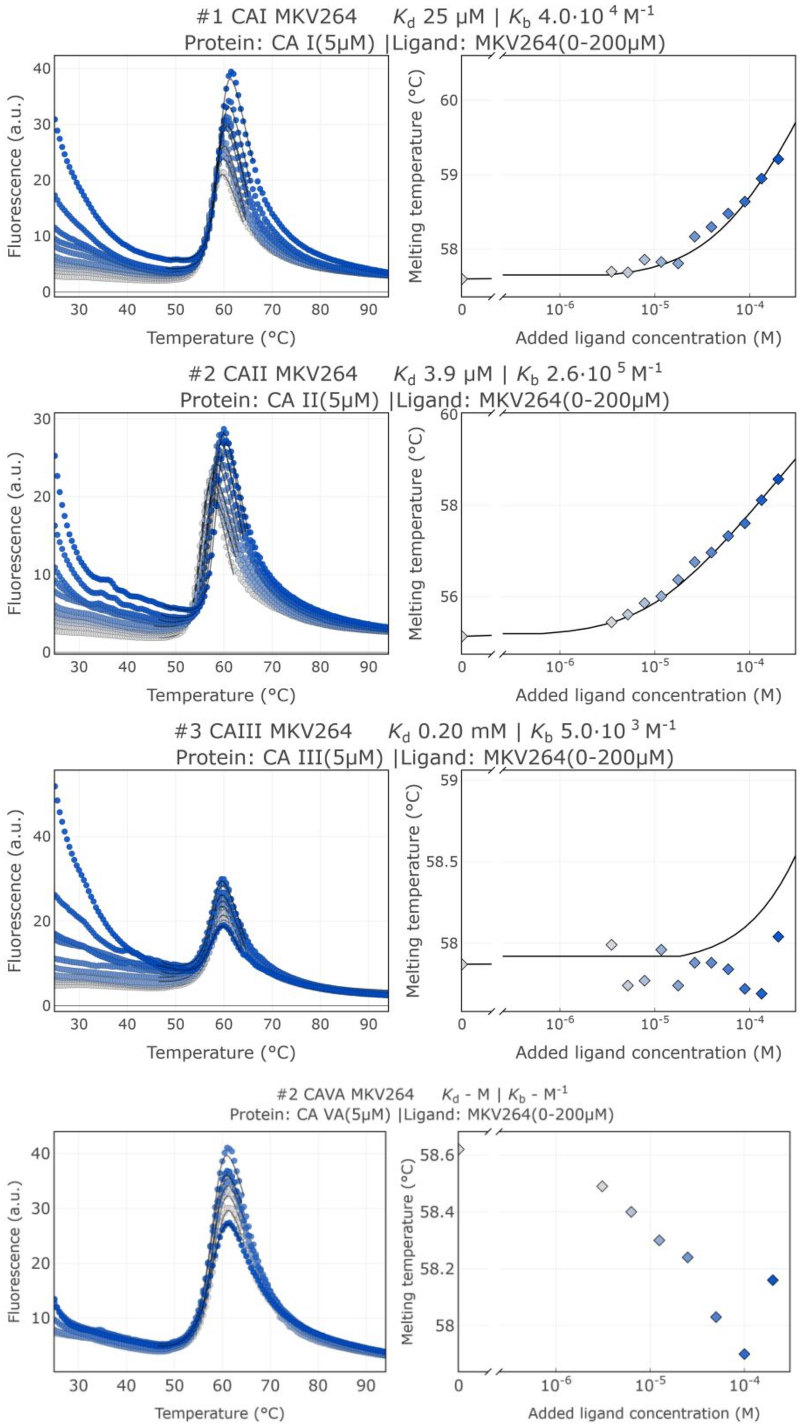

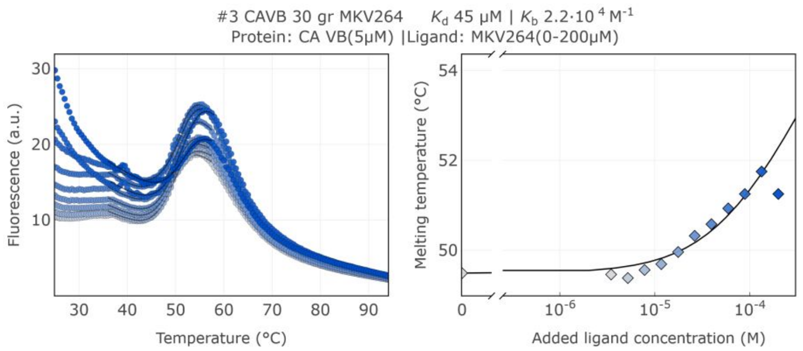
#3 CAVB 30 gr MKV264 K_d 45 µM | K_b $2.2\cdot10^4$ M^{-1}
Protein: CA VB(5µM) |Ligand: MKV264(0-200µM)
Fluorescence (a.u.)
Temperature (°C)
Melting temperature (°C)
Added ligand concentration (M)

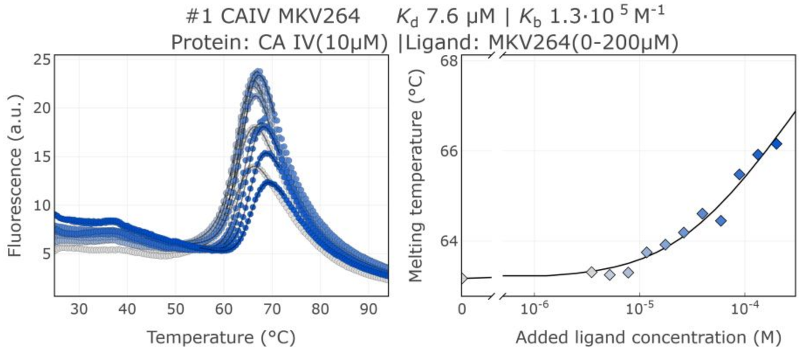
#1 CAIV MKV264 K_d 7.6 µM | K_b $1.3\cdot10^5$ M^{-1}
Protein: CA IV(10µM) |Ligand: MKV264(0-200µM)
Fluorescence (a.u.)
Temperature (°C)
Melting temperature (°C)
Added ligand concentration (M)

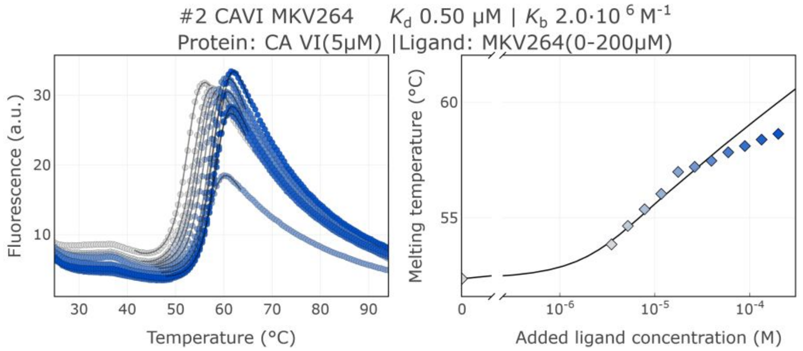
#2 CAVI MKV264 K_d 0.50 µM | K_b $2.0\cdot10^6$ M^{-1}
Protein: CA VI(5µM) |Ligand: MKV264(0-200µM)
Fluorescence (a.u.)
Temperature (°C)
Melting temperature (°C)
Added ligand concentration (M)

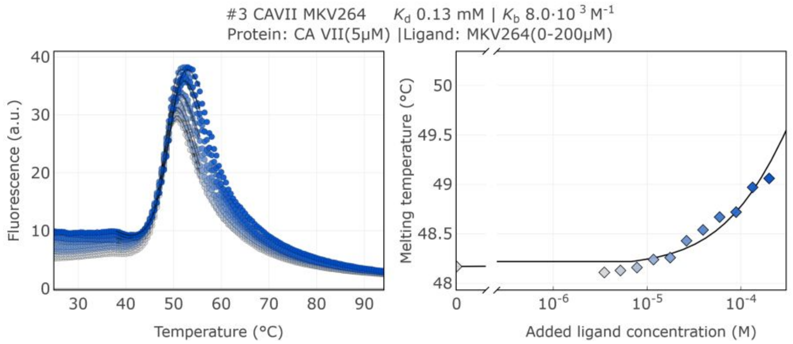
#3 CAVII MKV264 K_d 0.13 mM | K_b $8.0\cdot10^3$ M^{-1}
Protein: CA VII(5µM) |Ligand: MKV264(0-200µM)
Fluorescence (a.u.)
Temperature (°C)
Melting temperature (°C)
Added ligand concentration (M)

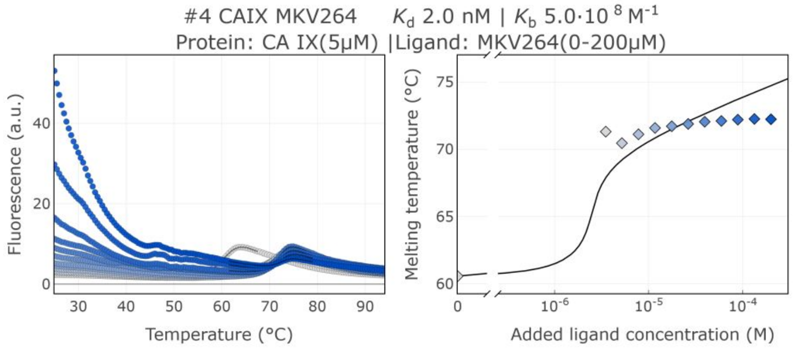
#4 CAIX MKV264 K_d 2.0 nM | K_b $5.0 \cdot 10^{8}$ M^{-1}
Protein: CA IX(5μM) |Ligand: MKV264(0-200μM)
Fluorescence (a.u.)
Temperature (°C)
Melting temperature (°C)
Added ligand concentration (M)

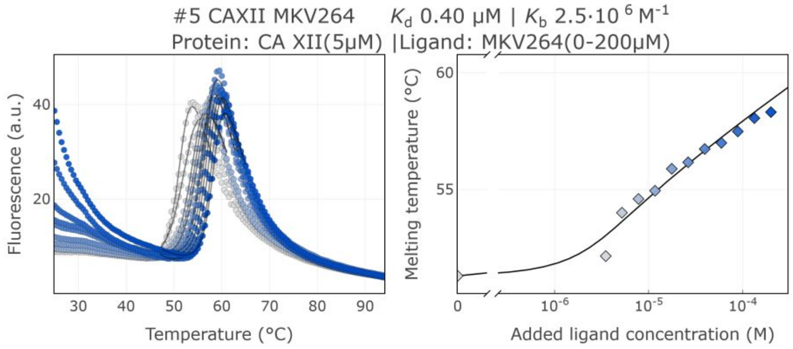
#5 CAXII MKV264 K_d 0.40 μM | K_b $2.5 \cdot 10^{6}$ M^{-1}
Protein: CA XII(5μM) |Ligand: MKV264(0-200μM)
Fluorescence (a.u.)
Temperature (°C)
Melting temperature (°C)
Added ligand concentration (M)

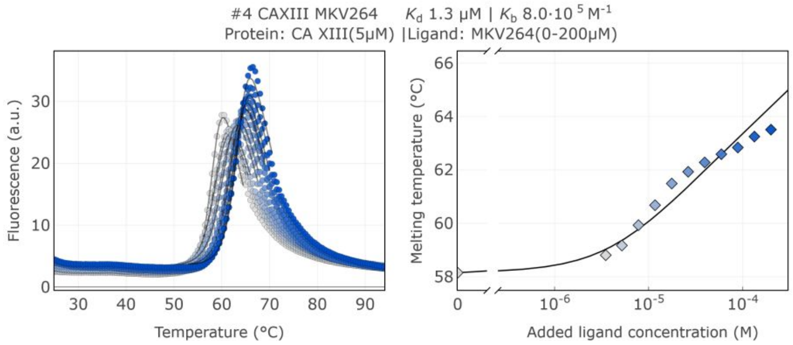
#4 CAXIII MKV264 K_d 1.3 μM | K_b $8.0 \cdot 10^{5}$ M^{-1}
Protein: CA XIII(5μM) |Ligand: MKV264(0-200μM)
Fluorescence (a.u.)
Temperature (°C)
Melting temperature (°C)
Added ligand concentration (M)

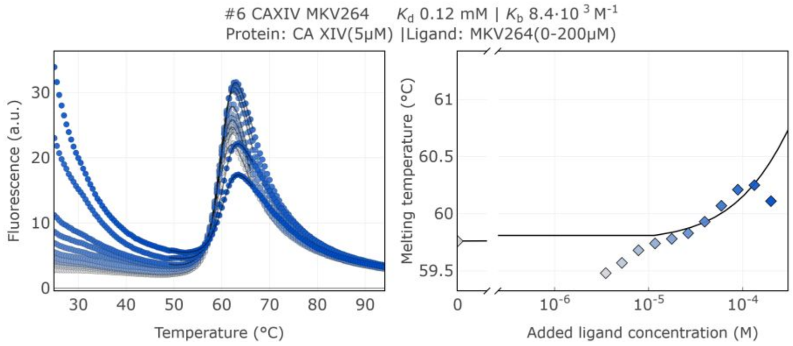
#6 CAXIV MKV264 K_d 0.12 mM | K_b $8.4 \cdot 10^{3}$ M^{-1}
Protein: CA XIV(5μM) |Ligand: MKV264(0-200μM)
Fluorescence (a.u.)
Temperature (°C)
Melting temperature (°C)
Added ligand concentration (M)

Figure S9. Compound 12 (MKV274) binding to CA isozymes

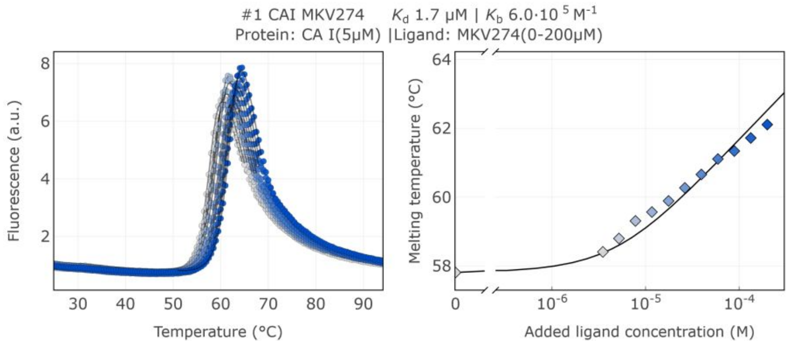

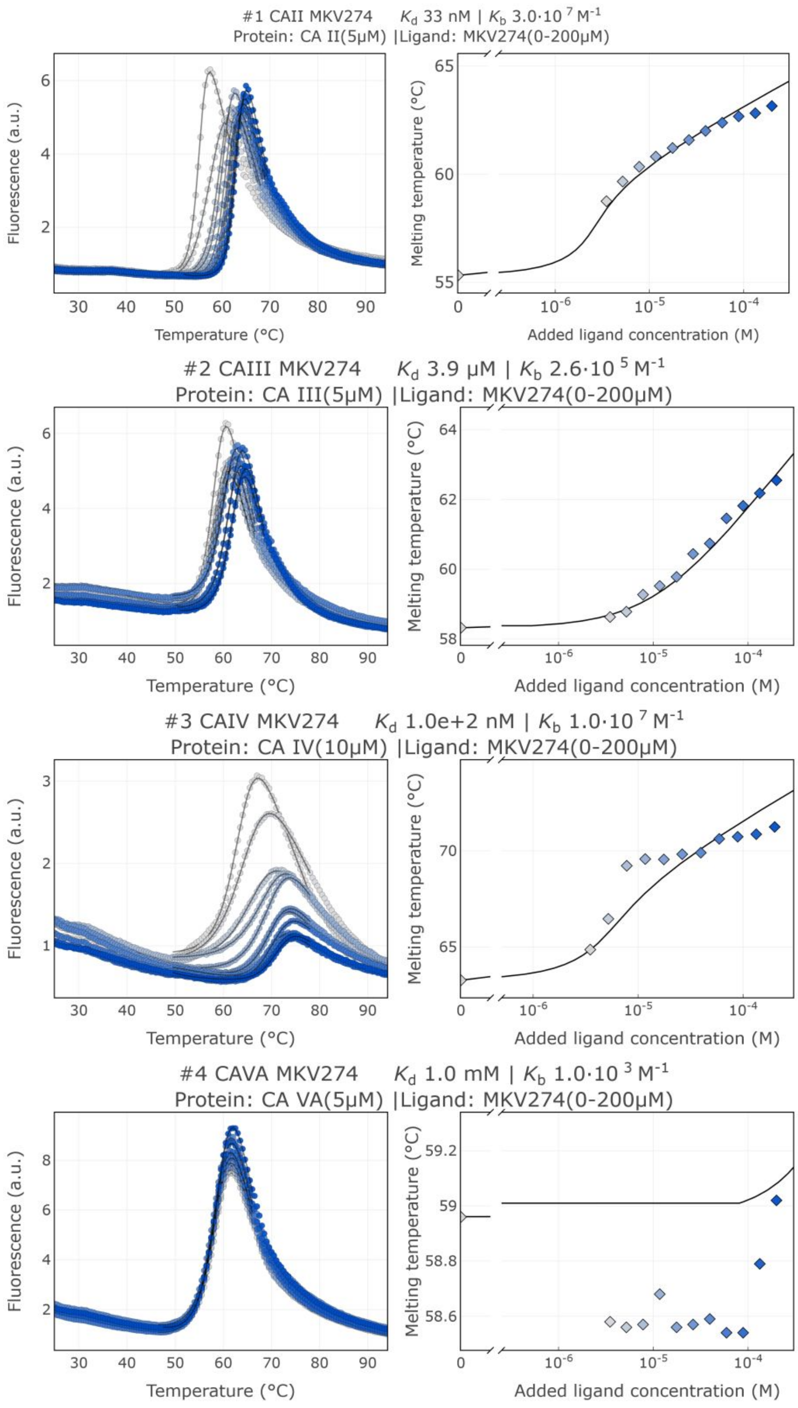
#1 CAII MKV274 K_d 33 nM | K_b $3.0\cdot10^{7}$ M^{-1}
Protein: CA II(5μM) |Ligand: MKV274(0-200μM)
#2 CAIII MKV274 K_d 3.9 μM | K_b $2.6\cdot10^{5}$ M^{-1}
Protein: CA III(5μM) |Ligand: MKV274(0-200μM)
#3 CAIV MKV274 K_d 1.0e+2 nM | K_b $1.0\cdot10^{7}$ M^{-1}
Protein: CA IV(10μM) |Ligand: MKV274(0-200μM)
#4 CAVA MKV274 K_d 1.0 mM | K_b $1.0\cdot10^{3}$ M^{-1}
Protein: CA VA(5μM) |Ligand: MKV274(0-200μM)
Fluorescence (a.u.)
Temperature (°C)
Melting temperature (°C)
Added ligand concentration (M)

#3 CAVB 30 gr MKV274 K_d 83 nM | K_b $1.2 \cdot 10^{7}$ M^{-1}
Protein: CA VB(5μM) |Ligand: MKV274(0-200μM)

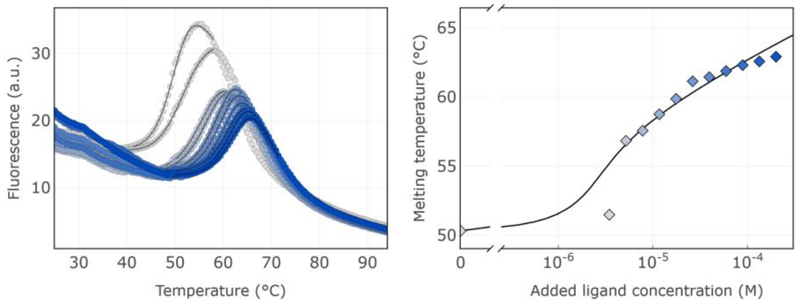

#2 CAVI MKV274 K_d 67 nM | K_b $1.5 \cdot 10^{7}$ M^{-1}
Protein: CA VI(5μM) |Ligand: MKV274(0-200μM)

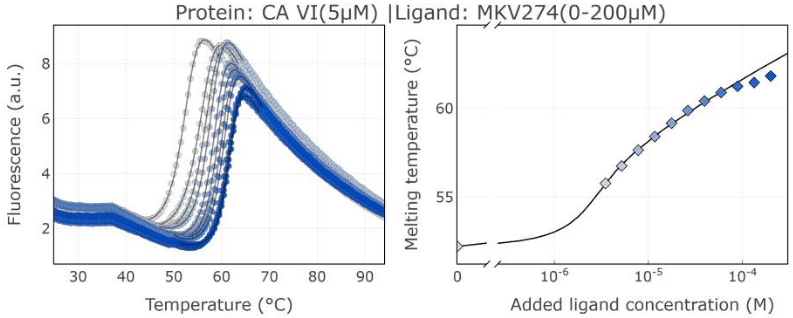

#3 CAVII MKV274 K_d 23 nM | K_b $4.4 \cdot 10^{7}$ M^{-1}
Protein: CA VII(5μM) |Ligand: MKV274(0-200μM)

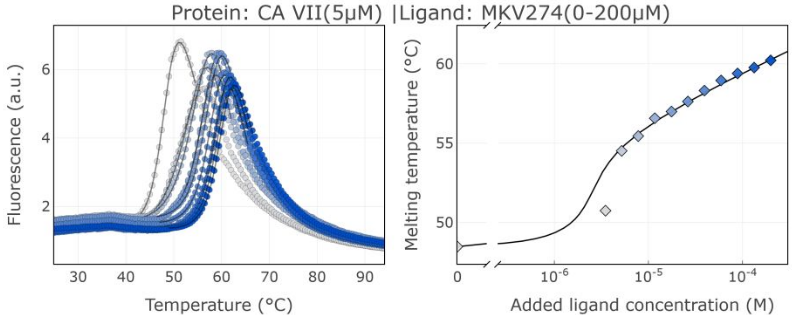

#6 CAIX MKV274 K_d 10 pM | K_b $1.0 \cdot 10^{11}$ M^{-1}
Protein: CA IX(5μM) |Ligand: MKV274(0-200μM)

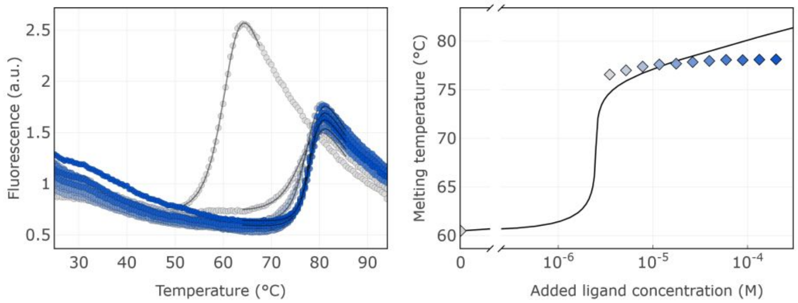

#4 CAXII MKV274 K_d 1.4 nM | K_b $7.0 \cdot 10^{8}$ M^{-1}
Protein: CA XII(5μM) |Ligand: MKV274(0-200μM)

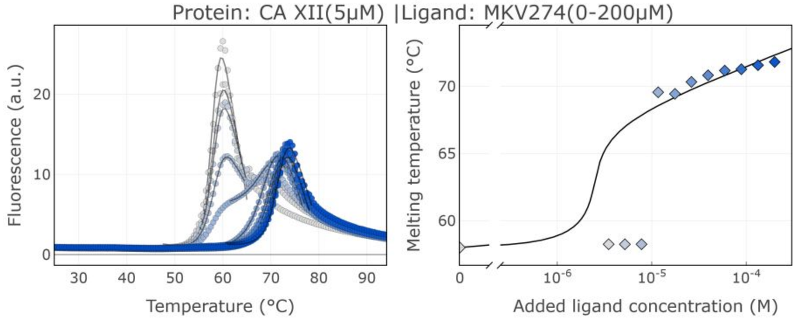

#5 CAXIII MKV274 K_d 1.7 nM | K_b $6.0 \cdot 10^{8}$ M^{-1}
Protein: CA XIII(5μM) |Ligand: MKV274(0-200μM)

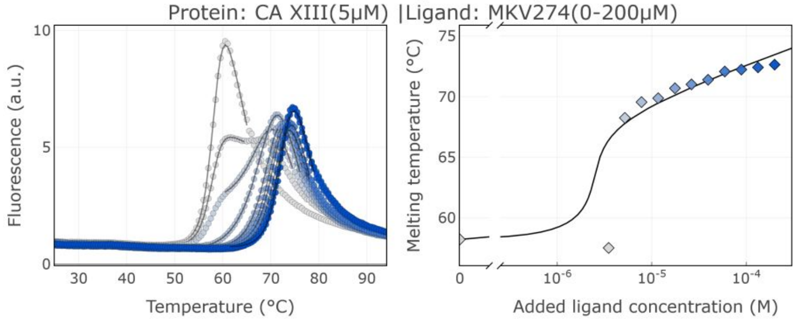

#6 CAXIV MKV274 K_d 2.5 nM | K_b $4.0 \cdot 10^{8}$ M^{-1}
Protein: CA XIV(5μM) |Ligand: MKV274(0-200μM)

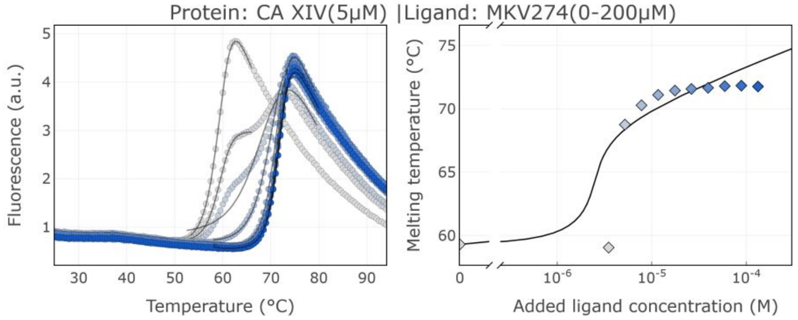

Figure S10. Compound 13 (MKV466) binding to CA isozymes

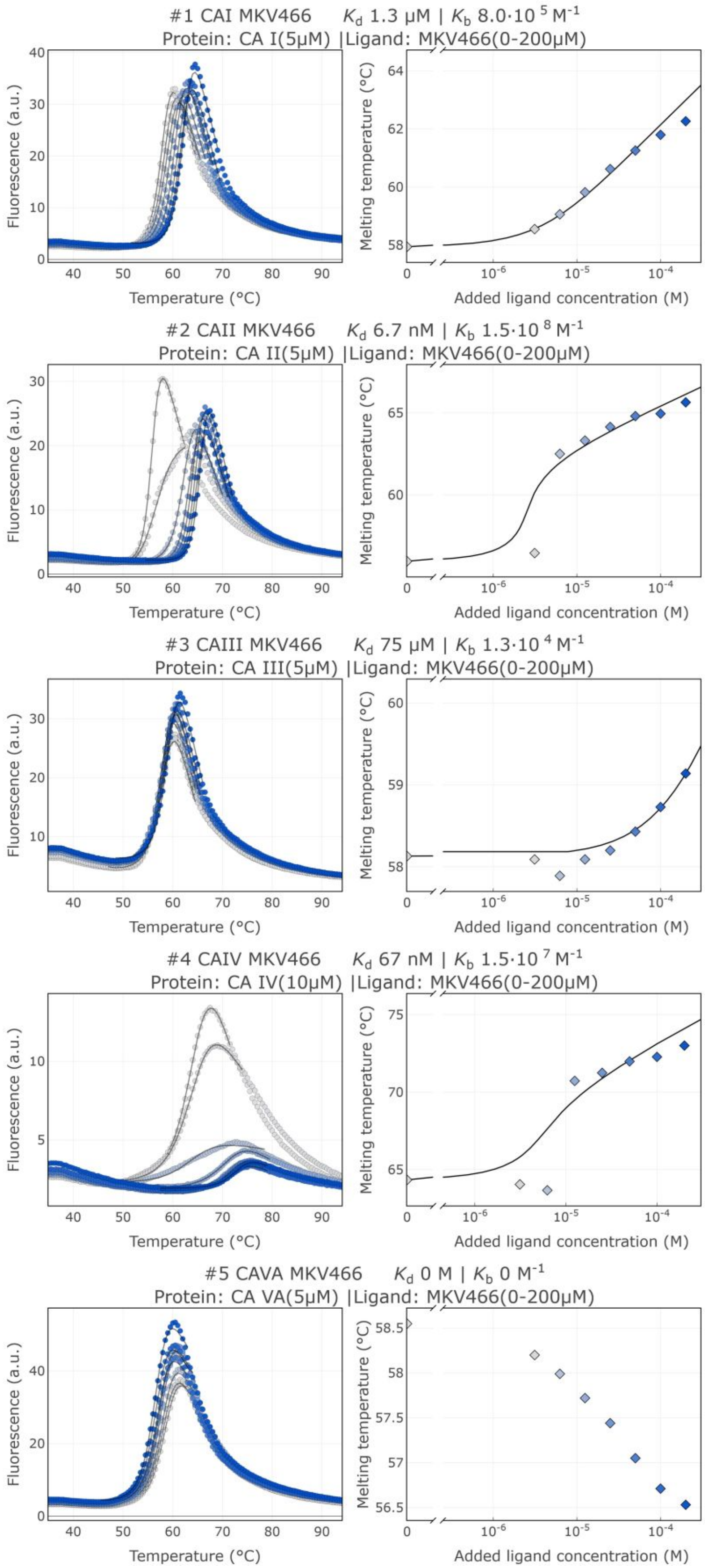

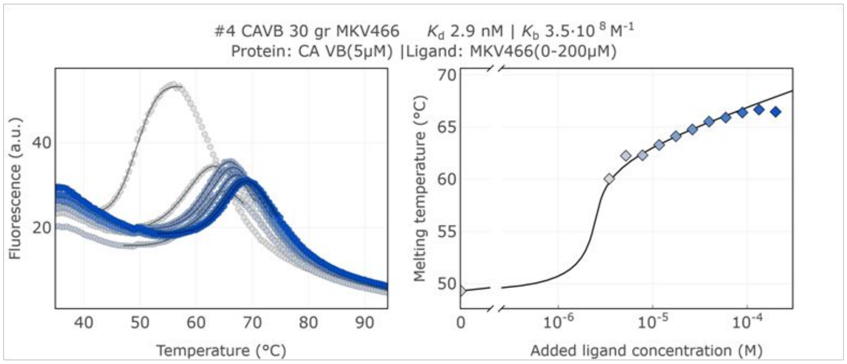
#4 CAVB 30 gr MKV466 K_d 2.9 nM | K_b 3.5·10^8 M^{-1}
Protein: CA VB(5μM) |Ligand: MKV466(0-200μM)
Fluorescence (a.u.)
Temperature (°C)
Melting temperature (°C)
Added ligand concentration (M)

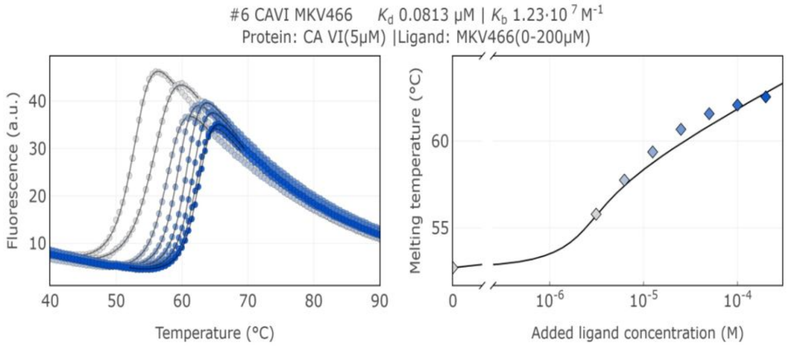
#6 CAVI MKV466 K_d 0.0813 μM | K_b 1.23·10^7 M^{-1}
Protein: CA VI(5μM) |Ligand: MKV466(0-200μM)
Fluorescence (a.u.)
Temperature (°C)
Melting temperature (°C)
Added ligand concentration (M)

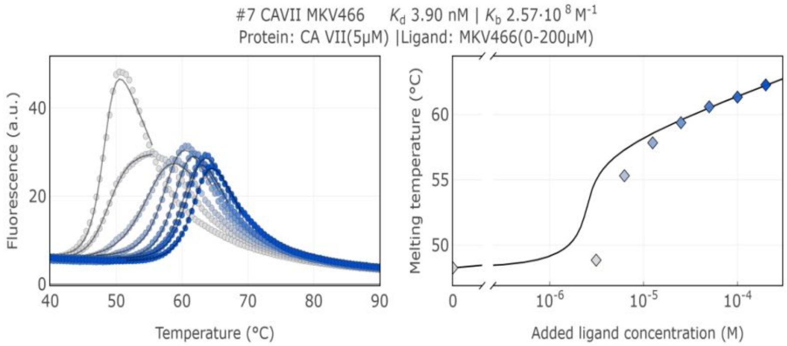
#7 CAVII MKV466 K_d 3.90 nM | K_b 2.57·10^8 M^{-1}
Protein: CA VII(5μM) |Ligand: MKV466(0-200μM)
Fluorescence (a.u.)
Temperature (°C)
Melting temperature (°C)
Added ligand concentration (M)

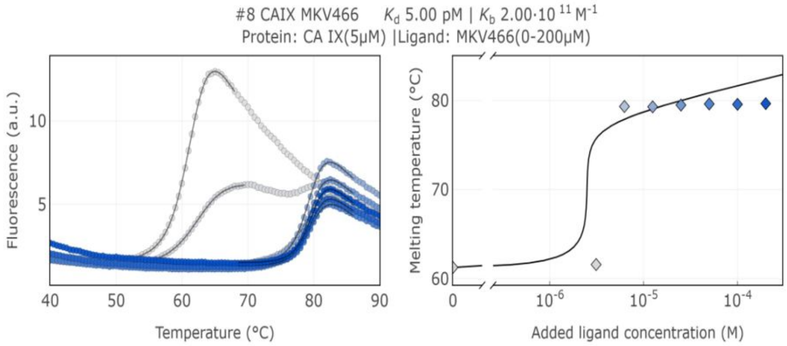
#8 CAIX MKV466 K_d 5.00 pM | K_b 2.00·10^{11} M^{-1}
Protein: CA IX(5μM) |Ligand: MKV466(0-200μM)
Fluorescence (a.u.)
Temperature (°C)
Melting temperature (°C)
Added ligand concentration (M)

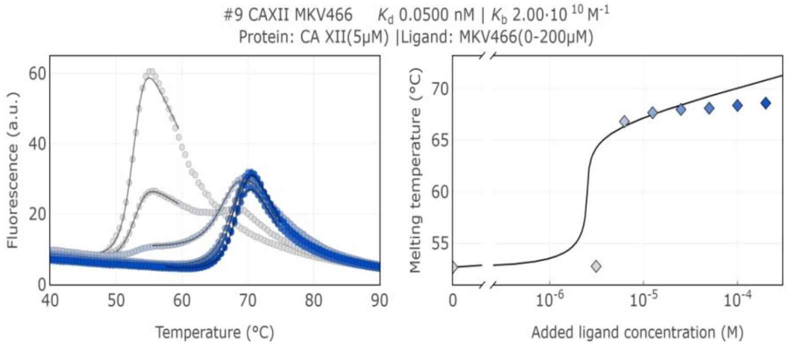
#9 CAXII MKV466 K_d 0.0500 nM | K_b 2.00·10^{10} M^{-1}
Protein: CA XII(5μM) |Ligand: MKV466(0-200μM)
Fluorescence (a.u.)
Temperature (°C)
Melting temperature (°C)
Added ligand concentration (M)

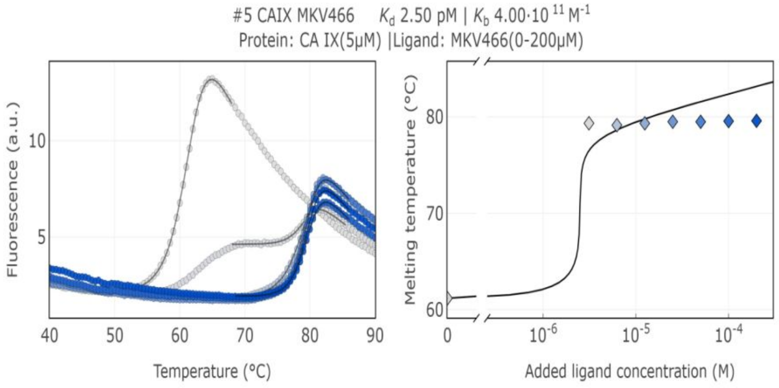
#5 CAIX MKV466 K_d 2.50 pM | K_b 4.00·10^{11} M^{-1}
Protein: CA IX(5μM) |Ligand: MKV466(0-200μM)
Fluorescence (a.u.)
Temperature (°C)
Melting temperature (°C)
Added ligand concentration (M)

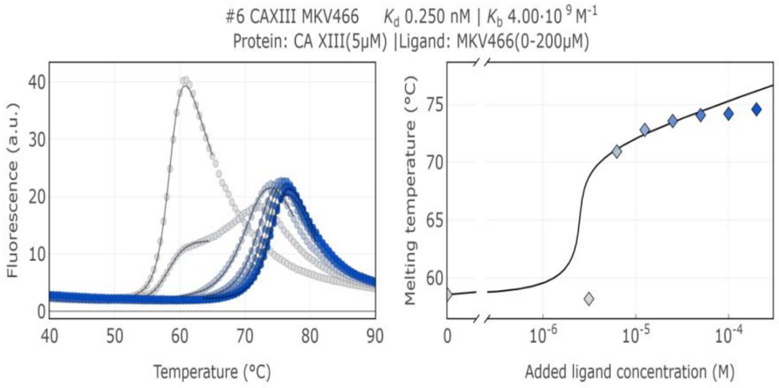
#6 CAXIII MKV466 K_d 0.250 nM | K_b 4.00·10^{9} M^{-1}
Protein: CA XIII(5μM) |Ligand: MKV466(0-200μM)
Fluorescence (a.u.)
Temperature (°C)
Melting temperature (°C)
Added ligand concentration (M)

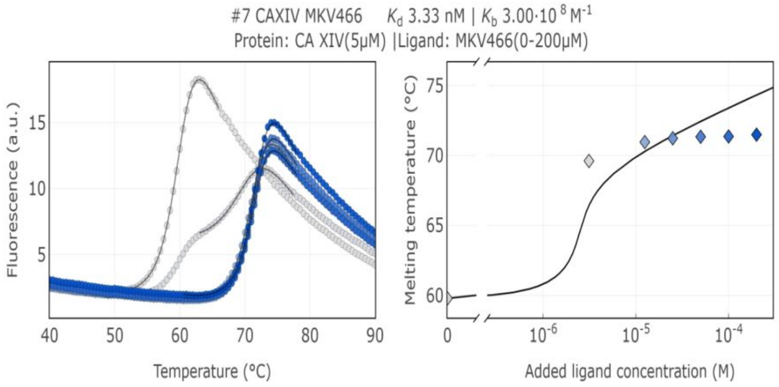
#7 CAXIV MKV466 K_d 3.33 nM | K_b 3.00·10^{8} M^{-1}
Protein: CA XIV(5μM) |Ligand: MKV466(0-200μM)
Fluorescence (a.u.)
Temperature (°C)
Melting temperature (°C)
Added ligand concentration (M)

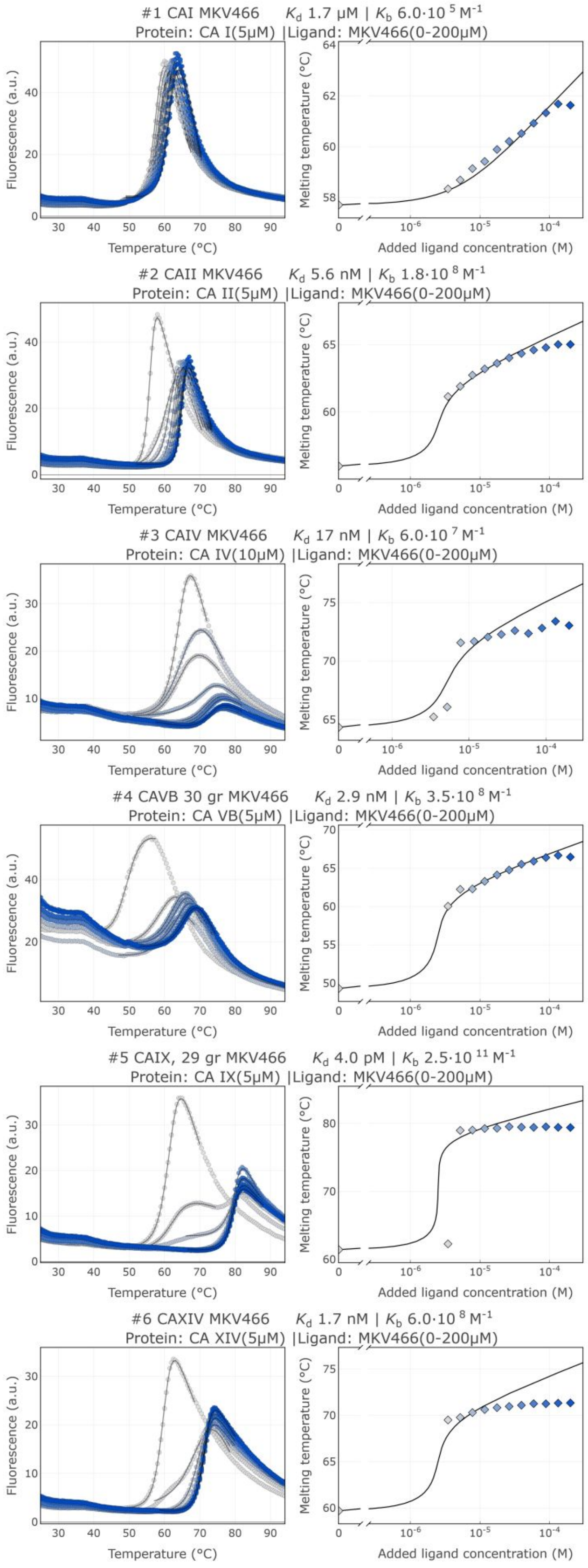
#1 CAI MKV466 K_d 1.7 µM | K_b 6.0·10 ^{5} M^{-1}
Protein: CA I(5µM) |Ligand: MKV466(0-200µM)
#2 CAII MKV466 K_d 5.6 nM | K_b 1.8·10 ^{8} M^{-1}
Protein: CA II(5µM) |Ligand: MKV466(0-200µM)
#3 CAIV MKV466 K_d 17 nM | K_b 6.0·10 ^{7} M^{-1}
Protein: CA IV(10µM) |Ligand: MKV466(0-200µM)
#4 CAVB 30 gr MKV466 K_d 2.9 nM | K_b 3.5·10 ^{8} M^{-1}
Protein: CA VB(5µM) |Ligand: MKV466(0-200µM)
#5 CAIX, 29 gr MKV466 K_d 4.0 pM | K_b 2.5·10 ^{11} M^{-1}
Protein: CA IX(5µM) |Ligand: MKV466(0-200µM)
#6 CAXIV MKV466 K_d 1.7 nM | K_b 6.0·10 ^{8} M^{-1}
Protein: CA XIV(5µM) |Ligand: MKV466(0-200µM)
Fluorescence (a.u.)
Temperature (°C)
Melting temperature (°C)
Added ligand concentration (M)

Figure S11. Compound 14 (KV558) binding to CA isozymes

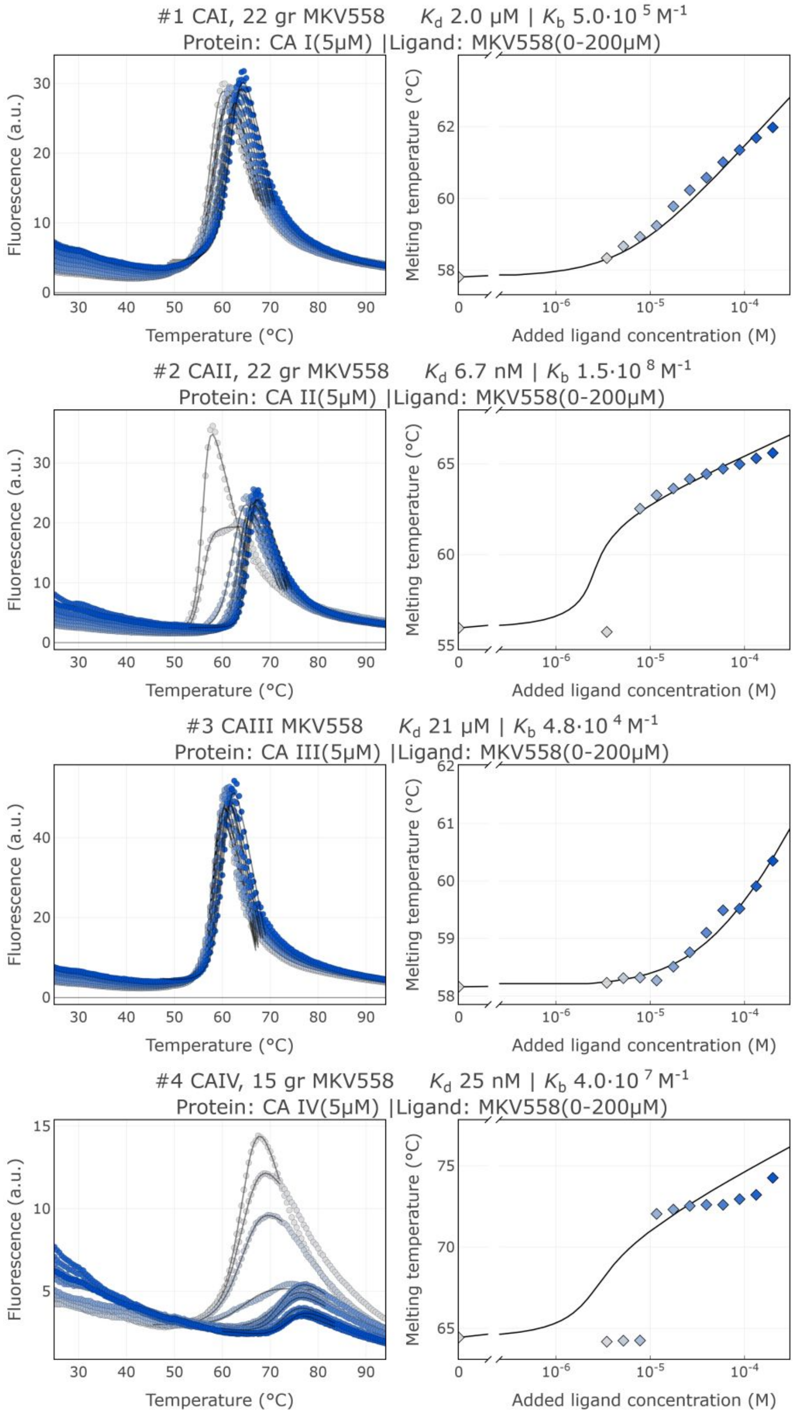

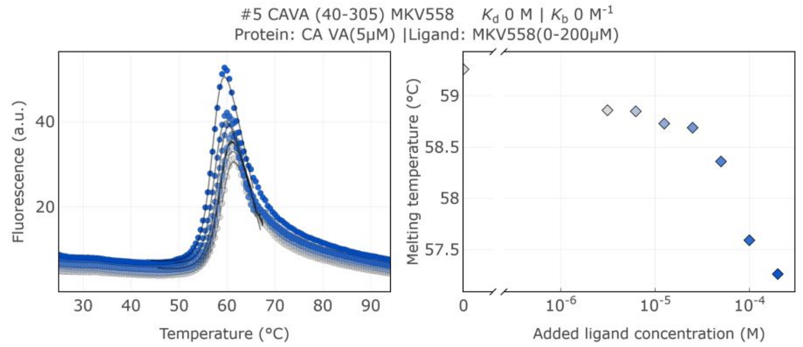
#5 CAVA (40-305) MKV558 K_d 0 M | K_b 0 M^{-1}
Protein: CA VA(5μM) |Ligand: MKV558(0-200μM)
Fluorescence (a.u.)
Temperature (°C)
Melting temperature (°C)
Added ligand concentration (M)

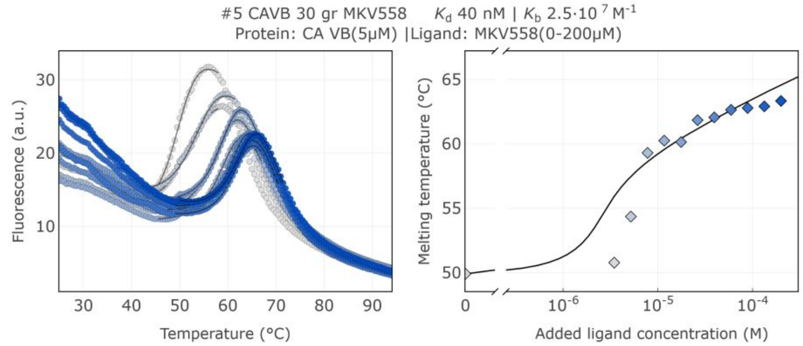
#5 CAVB 30 gr MKV558 K_d 40 nM | K_b $2.5 \cdot 10^{7}$ M^{-1}
Protein: CA VB(5μM) |Ligand: MKV558(0-200μM)
Fluorescence (a.u.)
Temperature (°C)
Melting temperature (°C)
Added ligand concentration (M)

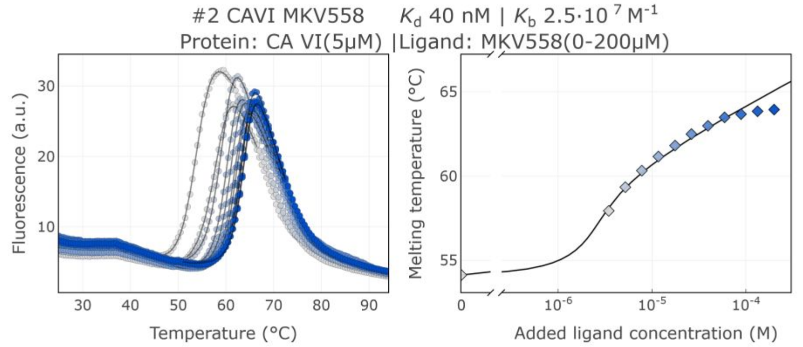
#2 CAVI MKV558 K_d 40 nM | K_b $2.5 \cdot 10^{7}$ M^{-1}
Protein: CA VI(5μM) |Ligand: MKV558(0-200μM)
Fluorescence (a.u.)
Temperature (°C)
Melting temperature (°C)
Added ligand concentration (M)

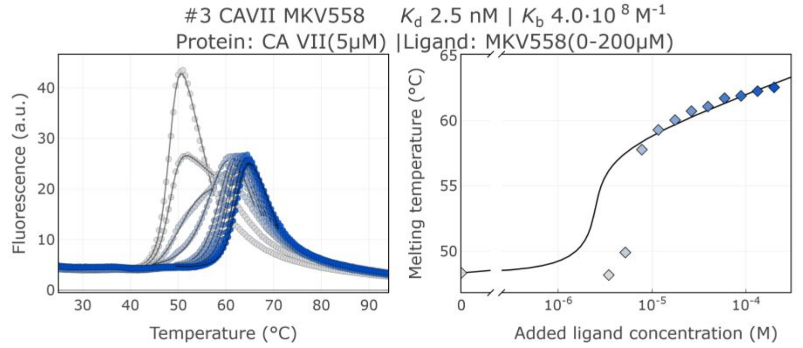
#3 CAVII MKV558 K_d 2.5 nM | K_b $4.0 \cdot 10^{8}$ M^{-1}
Protein: CA VII(5μM) |Ligand: MKV558(0-200μM)
Fluorescence (a.u.)
Temperature (°C)
Melting temperature (°C)
Added ligand concentration (M)

#6 CAIX, 29 gr MKV558 K_d 5.0 pM | K_b $2.0 \cdot 10^{11}$ M^{-1}
Protein: CA IX(5μM) |Ligand: MKV558(0-200μM)

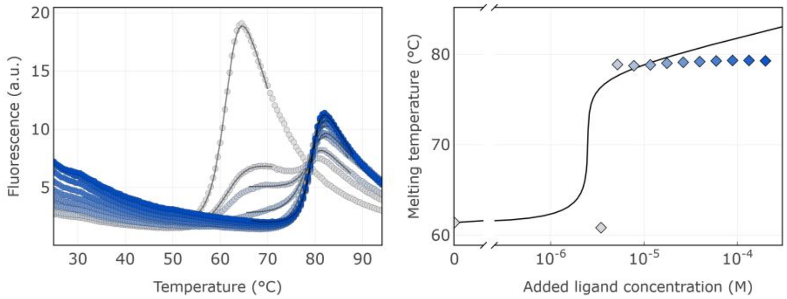

#4 CAXII MKV558 K_d 0.11 nM | K_b $9.0 \cdot 10^{9}$ M^{-1}
Protein: CA XII(5μM) |Ligand: MKV558(0-200μM)

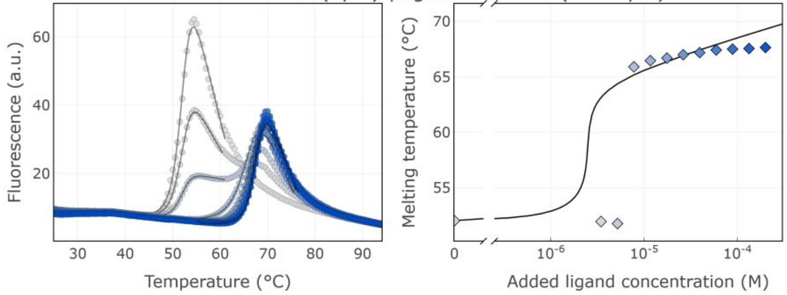

#5 CAXIII MKV558 K_d 1.7 nM | K_b $6.0 \cdot 10^{8}$ M^{-1}
Protein: CA XIII(5μM) |Ligand: MKV558(0-200μM)

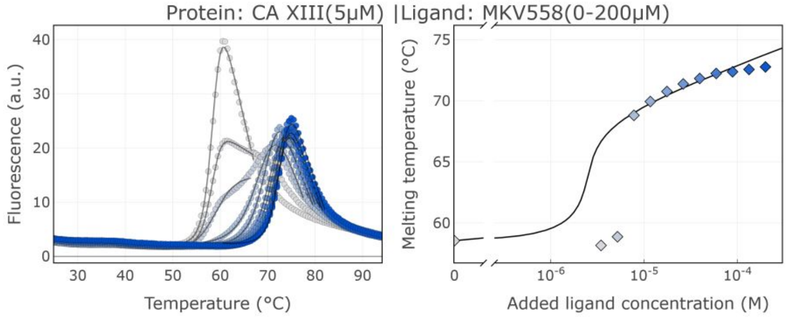

#6 CAXIV MKV558 K_d 2.5 nM | K_b $4.0 \cdot 10^{8}$ M^{-1}
Protein: CA XIV(4μM) |Ligand: MKV558(0-200μM)

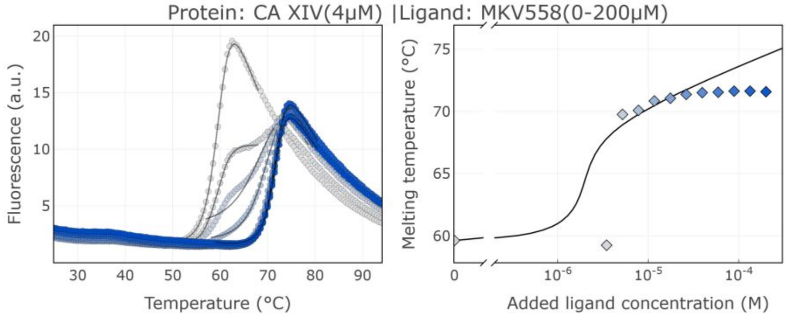

Figure S12. Compound 15 (AV17-33) binding to CA isozymes

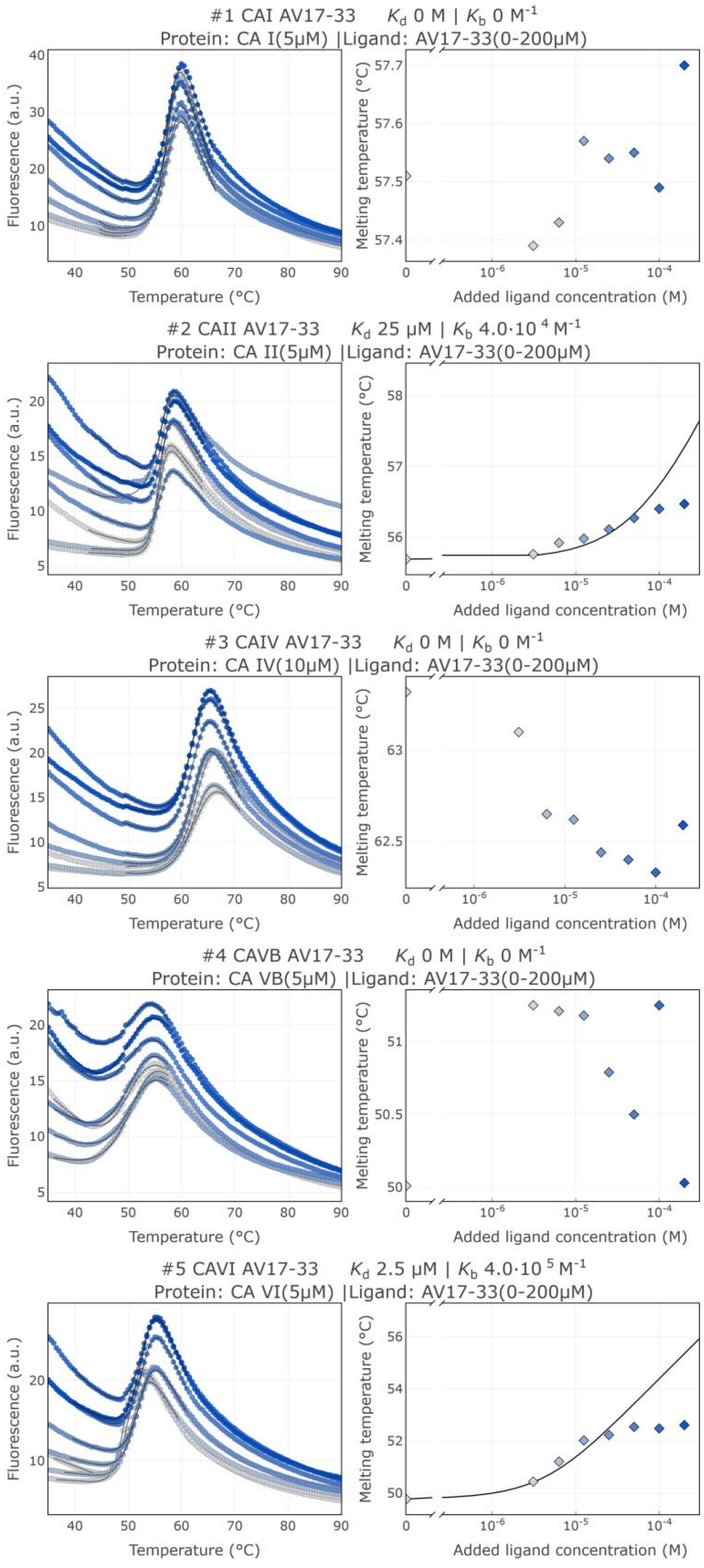

#1 CAIII AV17-33 K_d 0 M | K_b 0 M^{-1}
Protein: CA III(5μM) |Ligand: AV17-33(0-200μM)

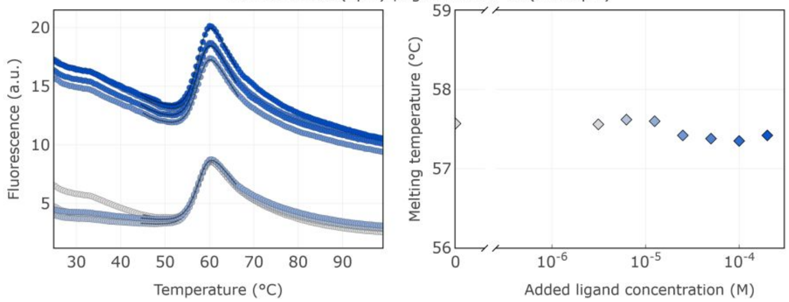

#6 CAIX AV17-33 K_d 10 nM | K_b 1.0·10 ^{8} M^{-1}
Protein: CA IX(5μM) |Ligand: AV17-33(0-200μM)

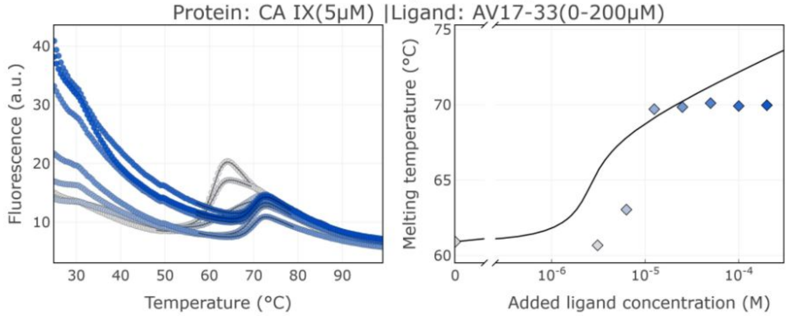

#7 CAXII AV17-33 K_d 2.5 μM | K_b 4.0·10 ^{5} M^{-1}
Protein: CA XII(5μM) |Ligand: AV17-33(0-200μM)

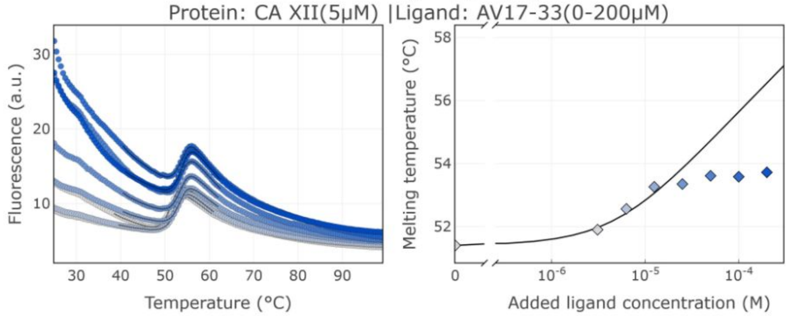

#8 CAXIII AV17-33 K_d 3.3 μM | K_b 3.0·10 ^{5} M^{-1}
Protein: CA XIII(5μM) |Ligand: AV17-33(0-200μM)

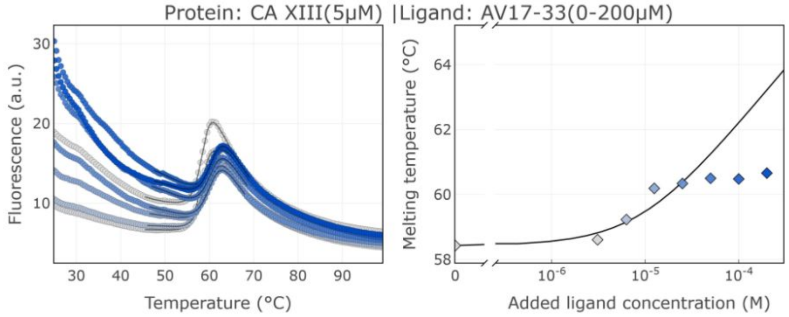

#9 CAXIV AV17-33 K_d 0 M | K_b 0 M^{-1}
Protein: CA XIV(5μM) |Ligand: AV17-33(0-200μM)

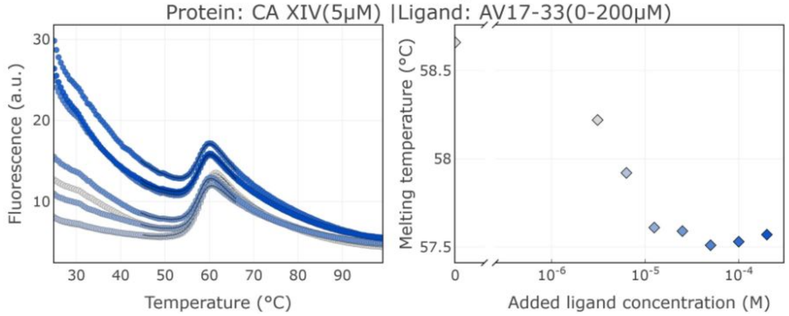

Figure S13. Compound 16 (AV17-42) binding to CA isozymes

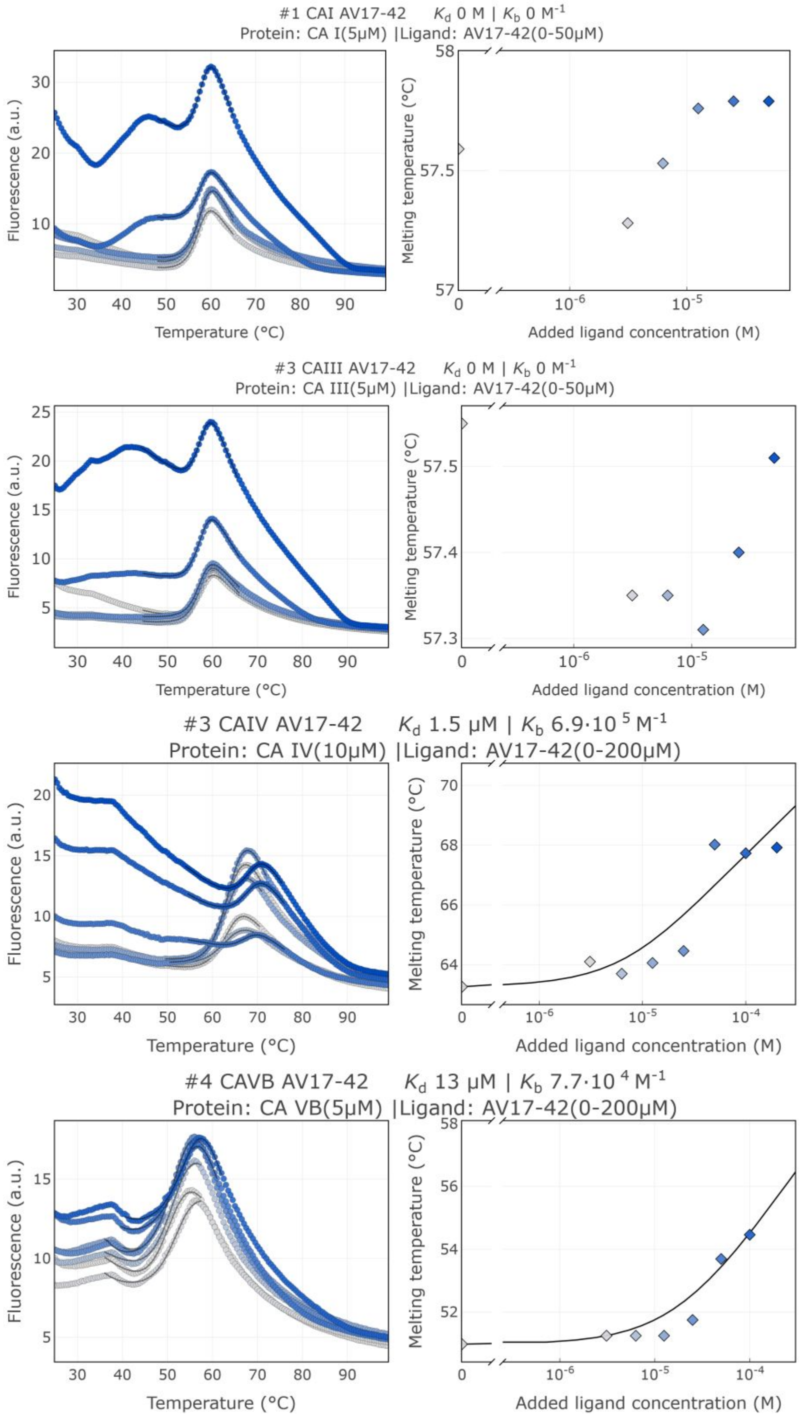

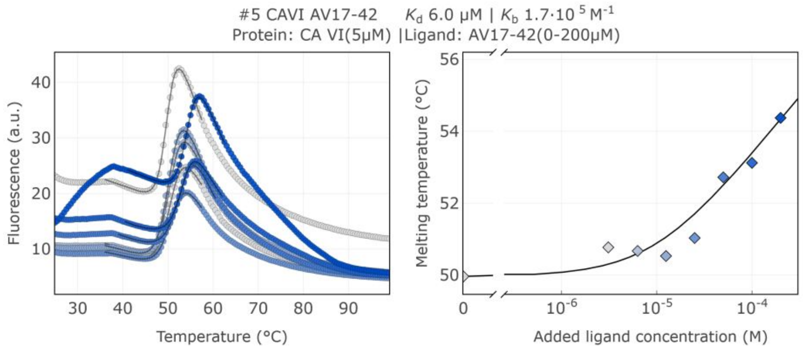
#5 CAVI AV17-42 K_d 6.0 µM | K_b 1.7·10 ^{5} M^{-1}
Protein: CA VI(5µM) |Ligand: AV17-42(0-200µM)
Fluorescence (a.u.)
Temperature (°C)
Melting temperature (°C)
Added ligand concentration (M)

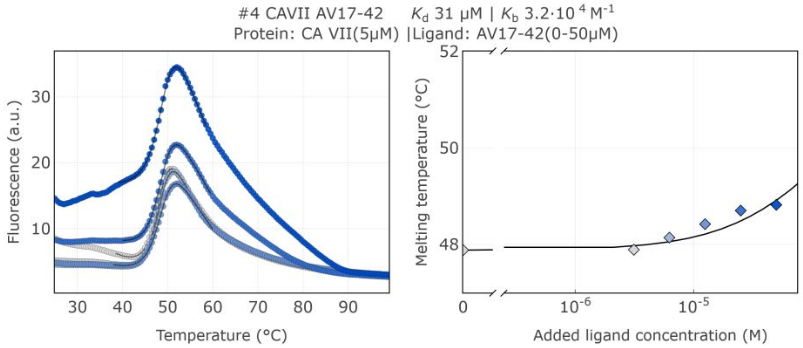
#4 CAVII AV17-42 K_d 31 µM | K_b 3.2·10 ^{4} M^{-1}
Protein: CA VII(5µM) |Ligand: AV17-42(0-50µM)
Fluorescence (a.u.)
Temperature (°C)
Melting temperature (°C)
Added ligand concentration (M)

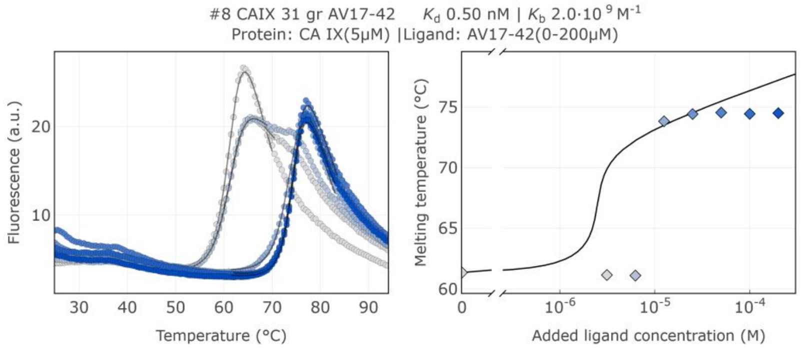
#8 CAIX 31 gr AV17-42 K_d 0.50 nM | K_b 2.0·10 ^{9} M^{-1}
Protein: CA IX(5µM) |Ligand: AV17-42(0-200µM)
Fluorescence (a.u.)
Temperature (°C)
Melting temperature (°C)
Added ligand concentration (M)

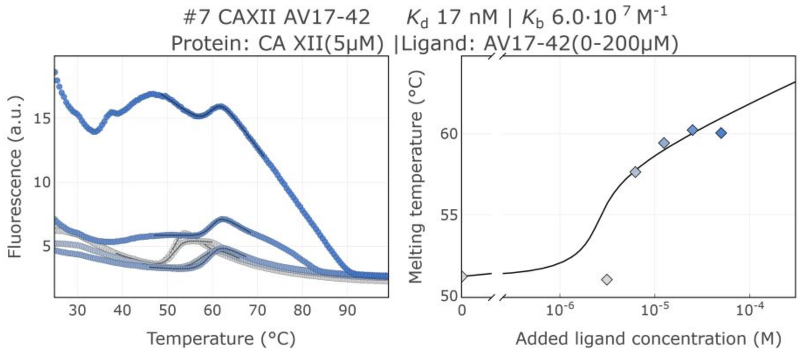
#7 CAXII AV17-42 K_d 17 nM | K_b 6.0·10 ^{7} M^{-1}
Protein: CA XII(5µM) |Ligand: AV17-42(0-200µM)
Fluorescence (a.u.)
Temperature (°C)
Melting temperature (°C)
Added ligand concentration (M)

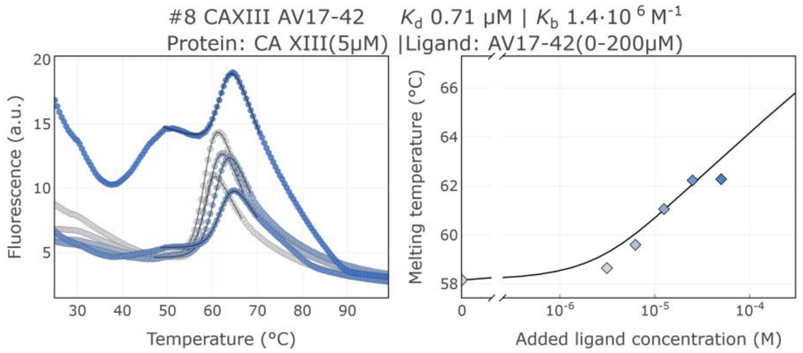
#8 CAXIII AV17-42 K_d 0.71 µM | K_b 1.4·10 ^{6} M^{-1}
Protein: CA XIII(5µM) |Ligand: AV17-42(0-200µM)
Fluorescence (a.u.)
Temperature (°C)
Melting temperature (°C)
Added ligand concentration (M)

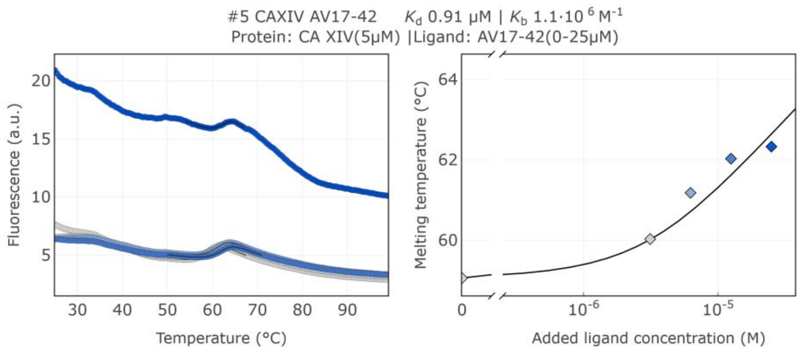
#5 CAXIV AV17-42 K_d 0.91 μM | K_b 1.1·10^{6} M^{-1}
Protein: CA XIV(5μM) |Ligand: AV17-42(0-25μM)
Fluorescence (a.u.)
Temperature (°C)
Melting temperature (°C)
Added ligand concentration (M)

Figure S14. Compound 17 (AV18-02) binding to CA isozymes

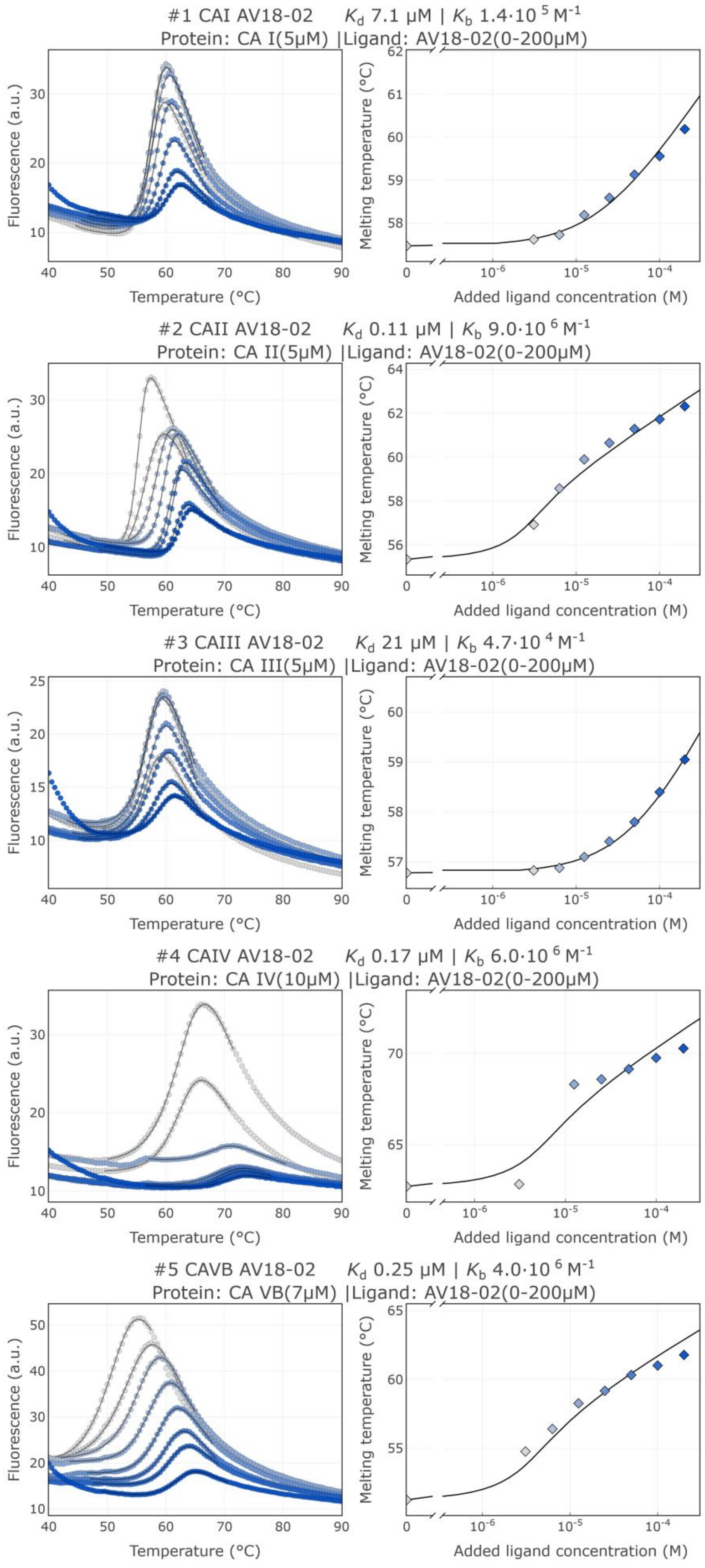

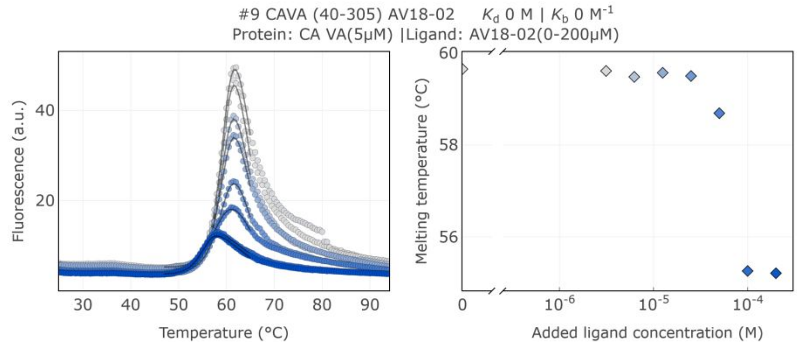
#9 CAVA (40-305) AV18-02 K_d 0 M | K_b 0 M^{-1}
Protein: CA VA(5μM) |Ligand: AV18-02(0-200μM)
Fluorescence (a.u.)
Temperature (°C)
Melting temperature (°C)
Added ligand concentration (M)

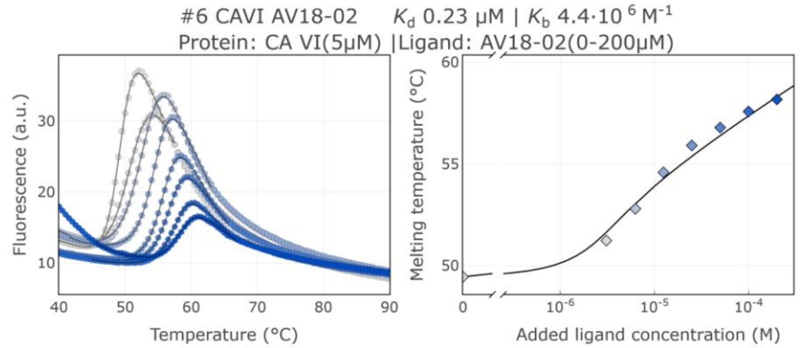
#6 CAVI AV18-02 K_d 0.23 μM | K_b 4.4·10 ^{6} M^{-1}
Protein: CA VI(5μM) |Ligand: AV18-02(0-200μM)
Fluorescence (a.u.)
Temperature (°C)
Melting temperature (°C)
Added ligand concentration (M)

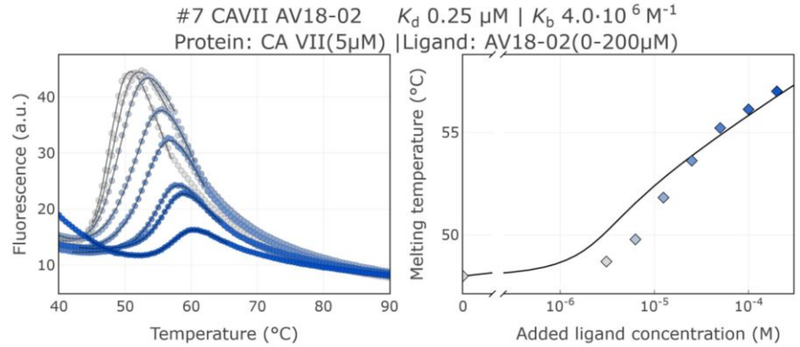
#7 CAVII AV18-02 K_d 0.25 μM | K_b 4.0·10 ^{6} M^{-1}
Protein: CA VII(5μM) |Ligand: AV18-02(0-200μM)
Fluorescence (a.u.)
Temperature (°C)
Melting temperature (°C)
Added ligand concentration (M)

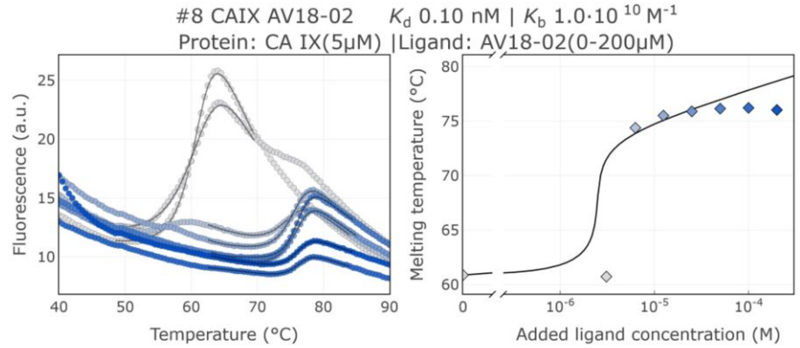
#8 CAIX AV18-02 K_d 0.10 nM | K_b 1.0·10 ^{10} M^{-1}
Protein: CA IX(5μM) |Ligand: AV18-02(0-200μM)
Fluorescence (a.u.)
Temperature (°C)
Melting temperature (°C)
Added ligand concentration (M)

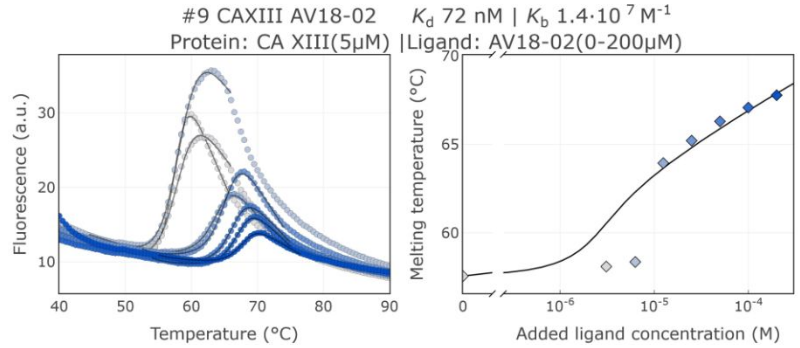
#9 CAXIII AV18-02 K_d 72 nM | K_b 1.4·10 ^{7} M^{-1}
Protein: CA XIII(5μM) |Ligand: AV18-02(0-200μM)
Fluorescence (a.u.)
Temperature (°C)
Melting temperature (°C)
Added ligand concentration (M)

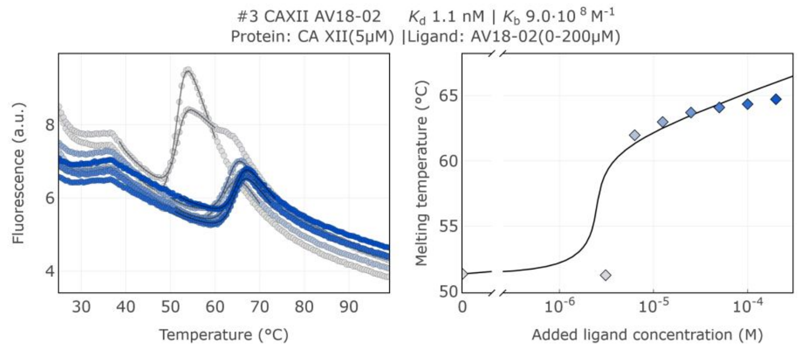
#3 CAXII AV18-02 K_d 1.1 nM | K_b 9.0·10 ^{8} M^{-1}
Protein: CA XII(5μM) |Ligand: AV18-02(0-200μM)
Fluorescence (a.u.)
Temperature (°C)
Melting temperature (°C)
Added ligand concentration (M)

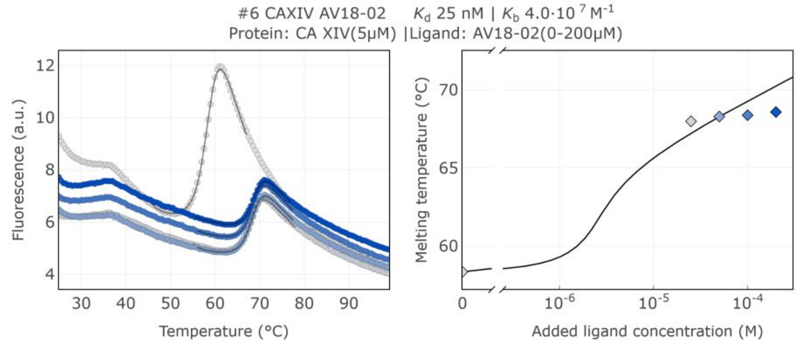
#6 CAXIV AV18-02 K_d 25 nM | K_b 4.0·10 ^{7} M^{-1}
Protein: CA XIV(5μM) |Ligand: AV18-02(0-200μM)
Fluorescence (a.u.)
Temperature (°C)
Melting temperature (°C)
Added ligand concentration (M)

Figure S15. Compound 18 (AV24-70) binding to CA isozymes

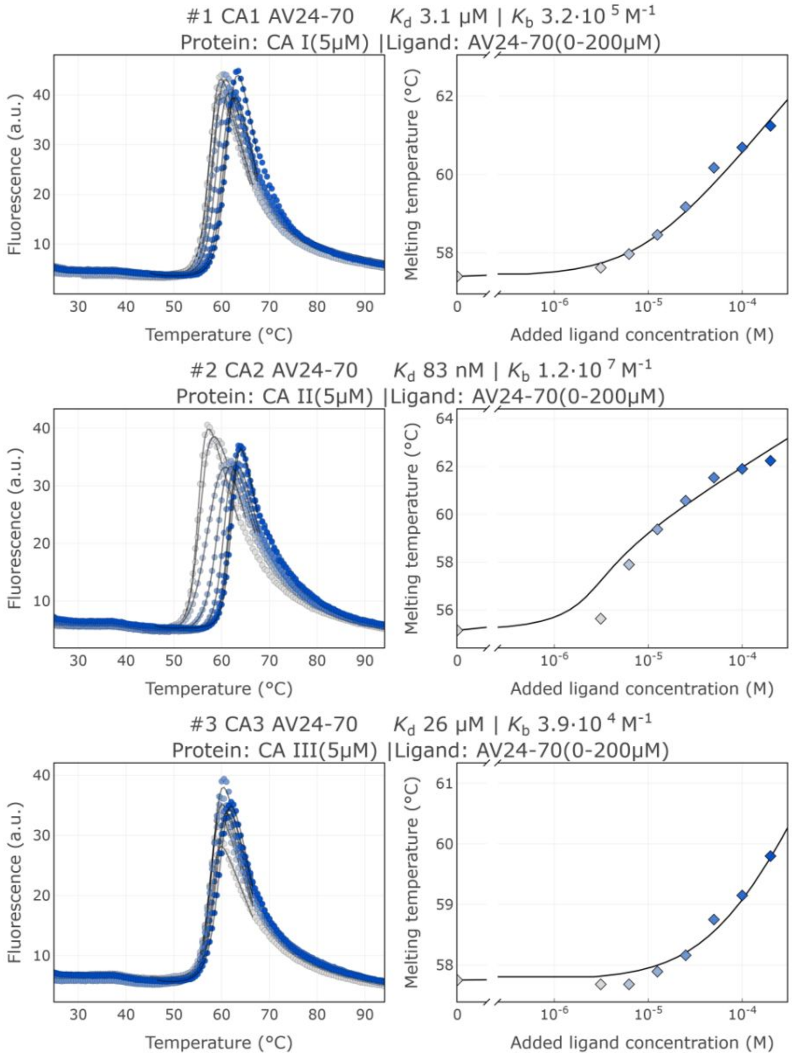

#1 CA5A AV24-70 K_d 0.20 mM | K_b $5.0 \cdot 10^{3}$ M^{-1}

Protein: CA VA(5μM) |Ligand: AV24-70(0-200μM)

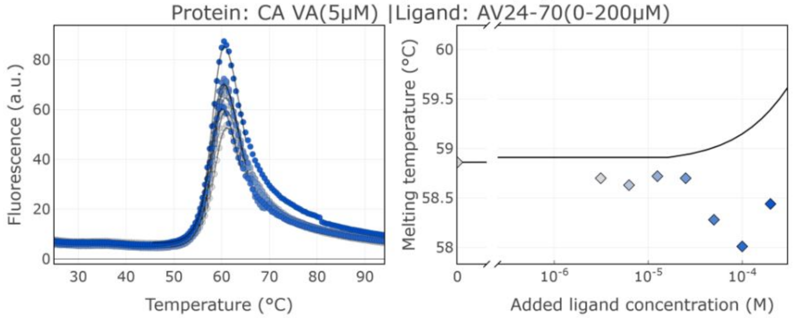

#2 CA5B AV24-70 K_d 0.63 μM | K_b $1.6 \cdot 10^{6}$ M^{-1}

Protein: CA VB(5μM) |Ligand: AV24-70(0-200μM)

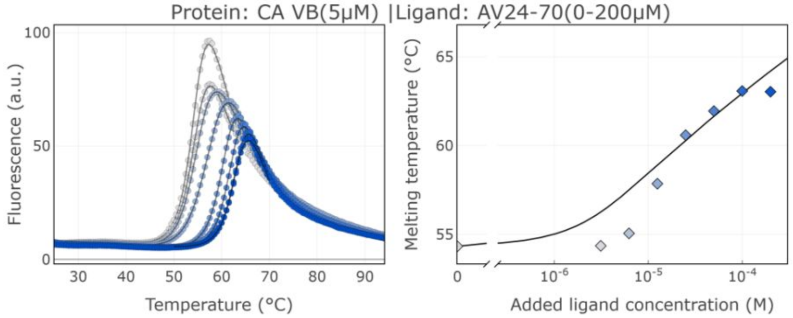

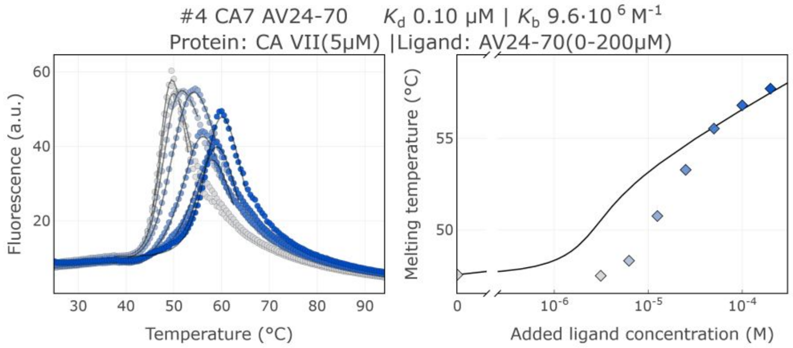
#4 CA7 AV24-70 K_d 0.10 µM | K_b 9.6·10^{6} M^{-1}
Protein: CA VII(5µM) |Ligand: AV24-70(0-200µM)
Fluorescence (a.u.)
Temperature (°C)
Melting temperature (°C)
Added ligand concentration (M)

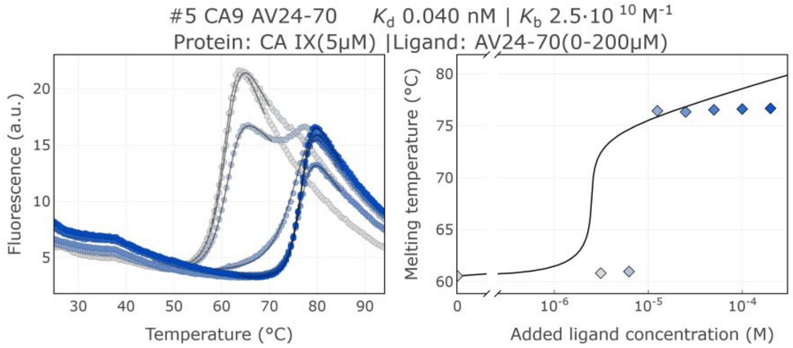
#5 CA9 AV24-70 K_d 0.040 nM | K_b 2.5·10^{10} M^{-1}
Protein: CA IX(5µM) |Ligand: AV24-70(0-200µM)
Fluorescence (a.u.)
Temperature (°C)
Melting temperature (°C)
Added ligand concentration (M)

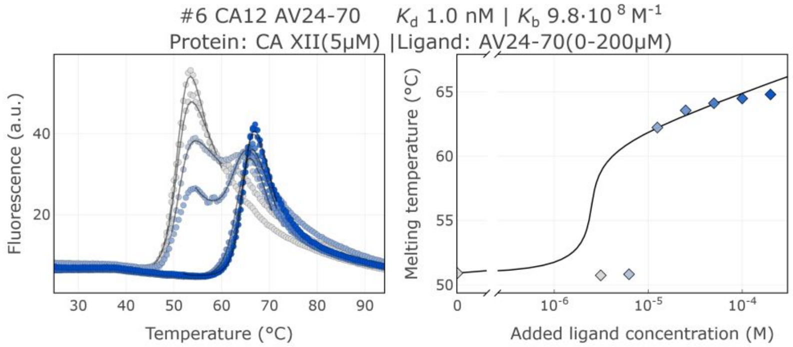
#6 CA12 AV24-70 K_d 1.0 nM | K_b 9.8·10^{8} M^{-1}
Protein: CA XII(5µM) |Ligand: AV24-70(0-200µM)
Fluorescence (a.u.)
Temperature (°C)
Melting temperature (°C)
Added ligand concentration (M)

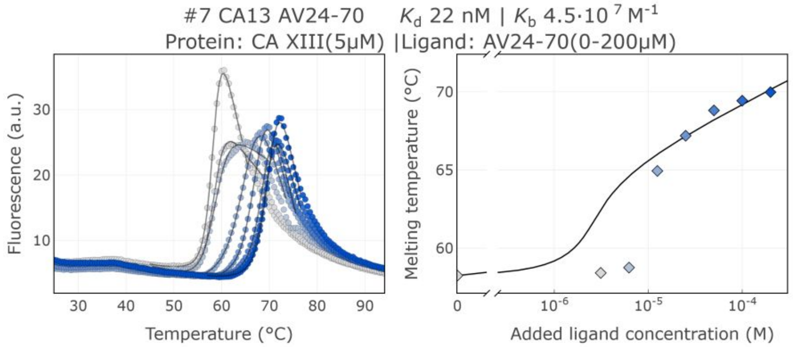
#7 CA13 AV24-70 K_d 22 nM | K_b 4.5·10^{7} M^{-1}
Protein: CA XIII(5µM) |Ligand: AV24-70(0-200µM)
Fluorescence (a.u.)
Temperature (°C)
Melting temperature (°C)
Added ligand concentration (M)

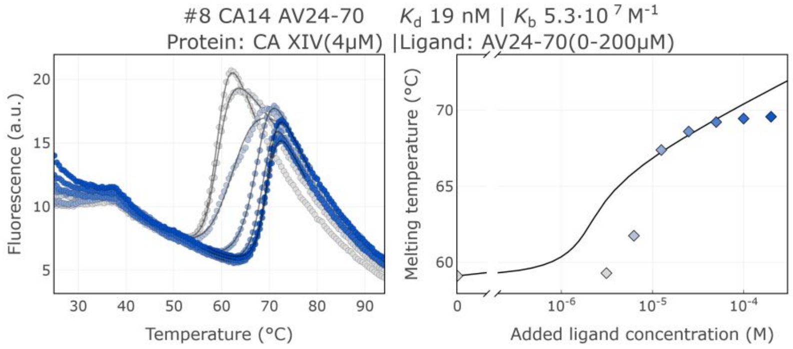
#8 CA14 AV24-70 K_d 19 nM | K_b 5.3·10^{7} M^{-1}
Protein: CA XIV(4µM) |Ligand: AV24-70(0-200µM)
Fluorescence (a.u.)
Temperature (°C)
Melting temperature (°C)
Added ligand concentration (M)

Figure S16. Compound 19 (AV24-72) binding to CA isozymes

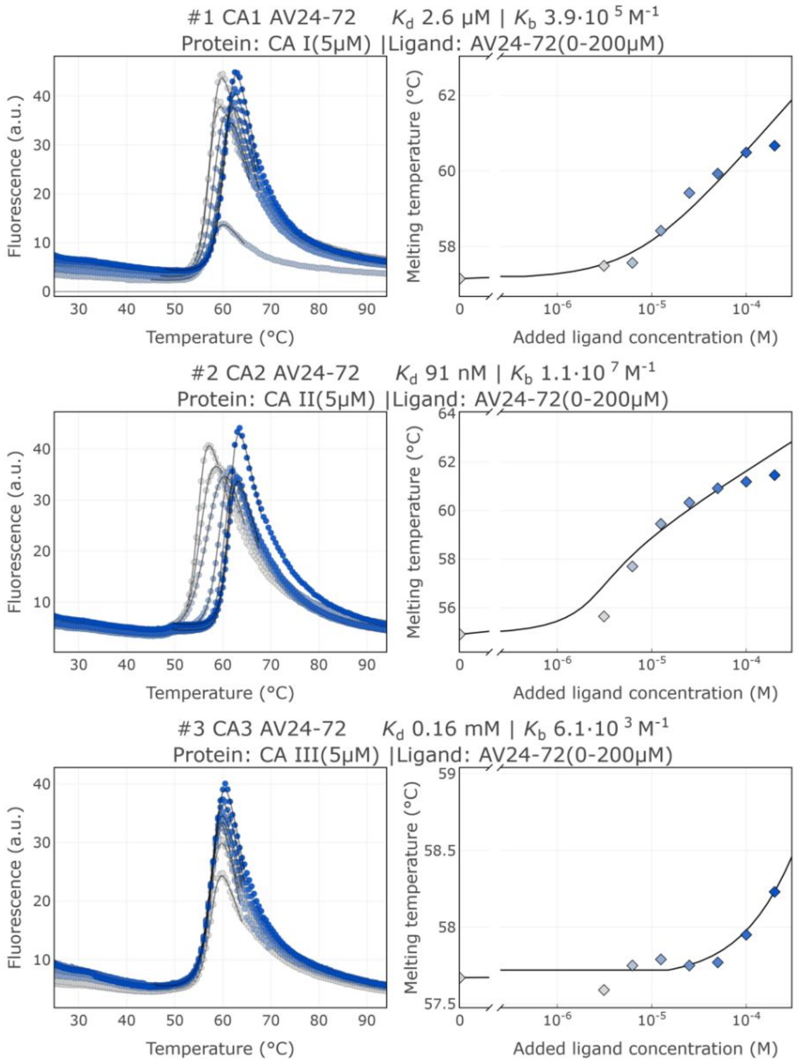

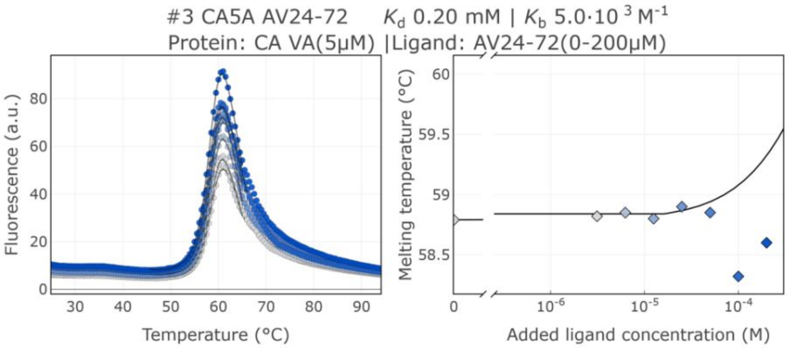
#3 CA5A AV24-72 K_d 0.20 mM | K_b 5.0·10^3 M^{-1}
Protein: CA VA(5μM) |Ligand: AV24-72(0-200μM)
Fluorescence (a.u.)
Temperature (°C)
Melting temperature (°C)
Added ligand concentration (M)

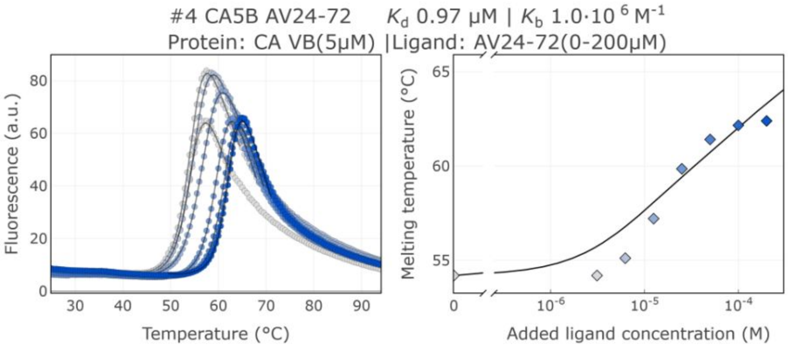
#4 CA5B AV24-72 K_d 0.97 μM | K_b 1.0·10^6 M^{-1}
Protein: CA VB(5μM) |Ligand: AV24-72(0-200μM)
Fluorescence (a.u.)
Temperature (°C)
Melting temperature (°C)
Added ligand concentration (M)

#4 CA7 AV24-72 K_d 0.30 µM | K_b $3.3 \cdot 10^{6}$ M^{-1}
Protein: CA VII(5µM) |Ligand: AV24-72(0-200µM)

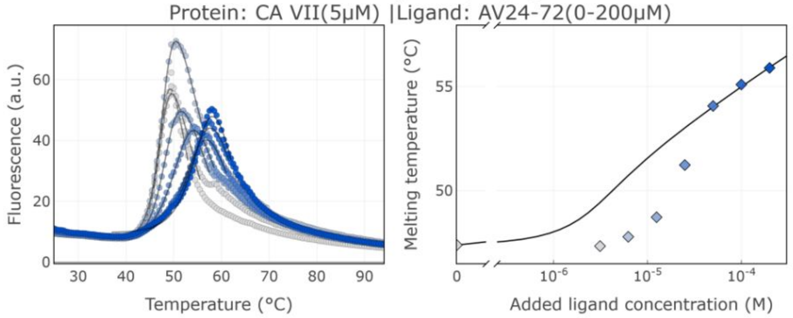

#5 CA9 AV24-72 K_d 0.067 nM | K_b $1.5 \cdot 10^{10}$ M^{-1}
Protein: CA IX(5µM) |Ligand: AV24-72(0-200µM)

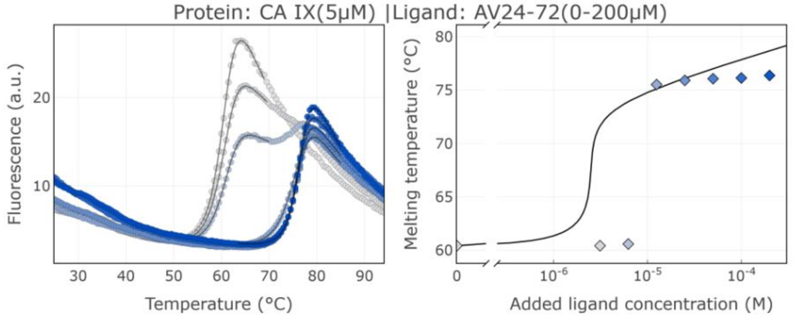

#6 CA12 AV24-72 K_d 1.1 nM | K_b $9.4 \cdot 10^{8}$ M^{-1}
Protein: CA XII(5µM) |Ligand: AV24-72(0-200µM)

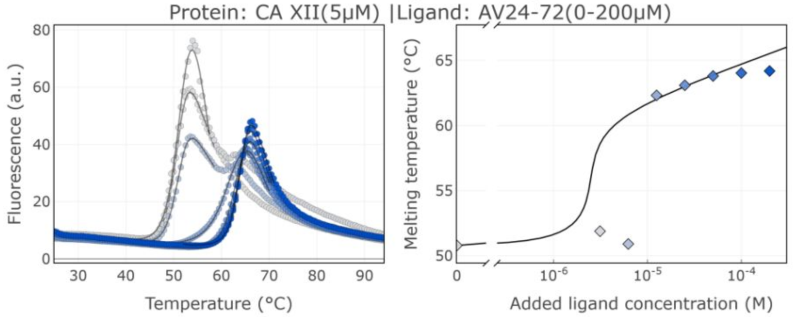

#7 CA13 AV24-72 K_d 30 nM | K_b $3.3 \cdot 10^{7}$ M^{-1}
Protein: CA XIII(5µM) |Ligand: AV24-72(0-200µM)

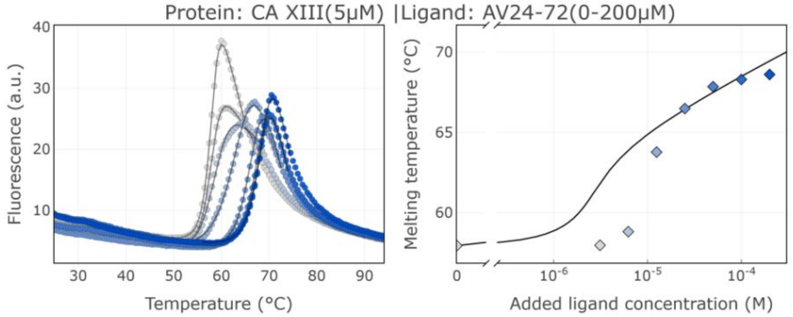

#5 CA14 AV24-72 K_d 59 nM | K_b $1.7 \cdot 10^{7}$ M^{-1}
Protein: CA XIV(5µM) |Ligand: AV24-72(0-200µM)

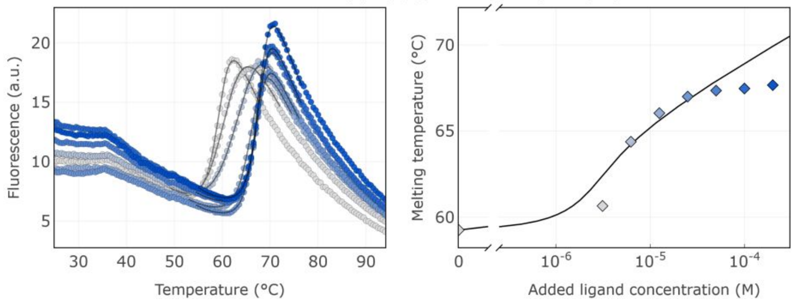

Figure S17. Compound 20 (AV18-13) binding to CA isozymes

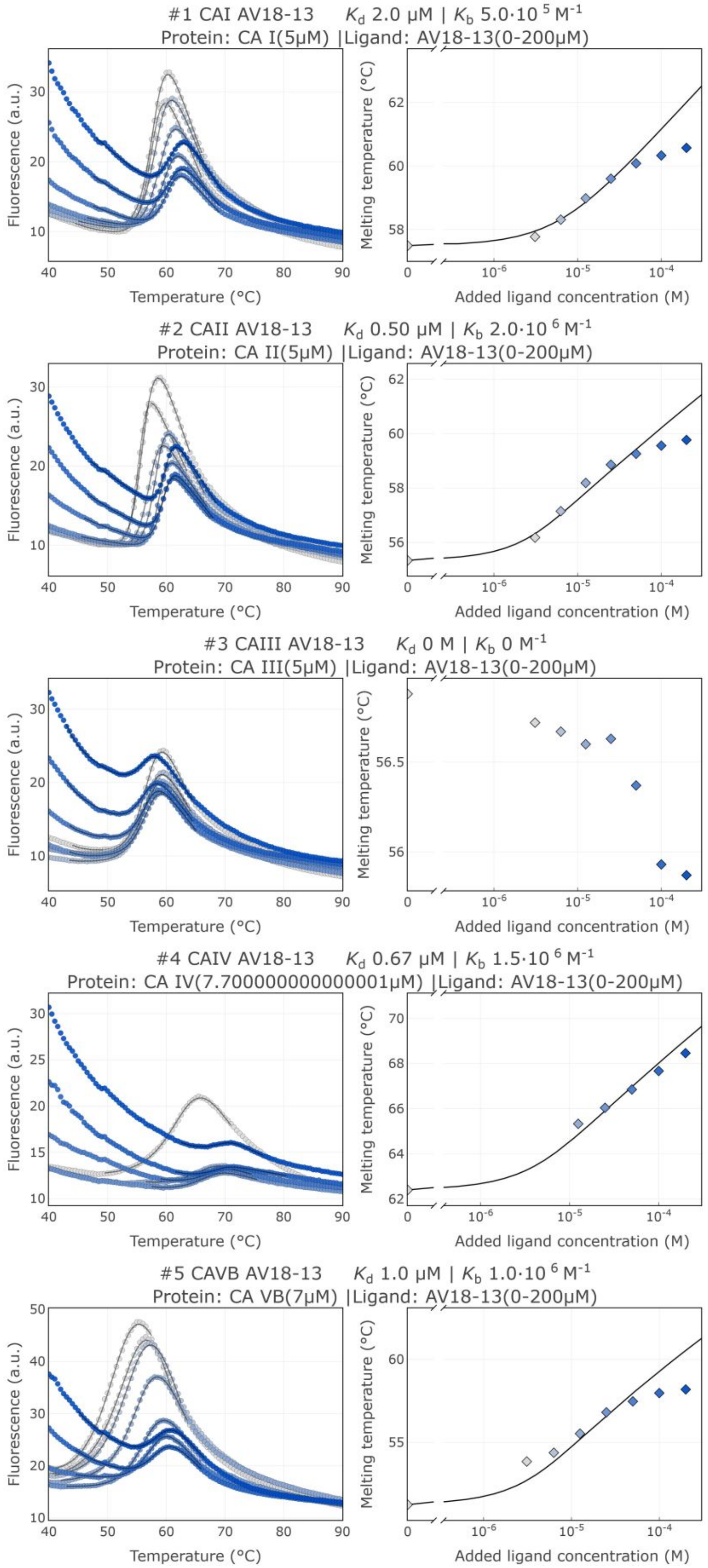

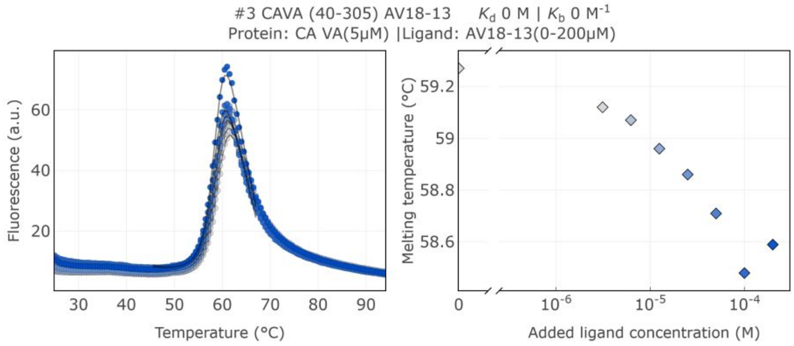
#3 CAVA (40-305) AV18-13 K_d 0 M | K_b 0 M^{-1}
Protein: CA VA(5μM) |Ligand: AV18-13(0-200μM)
Fluorescence (a.u.)
Temperature (°C)
Melting temperature (°C)
Added ligand concentration (M)

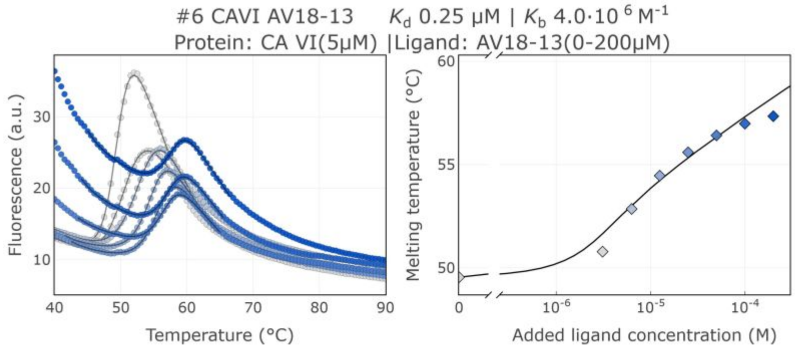
#6 CAVI AV18-13 K_d 0.25 μM | K_b 4.0·10 ^{6} M^{-1}
Protein: CA VI(5μM) |Ligand: AV18-13(0-200μM)
Fluorescence (a.u.)
Temperature (°C)
Melting temperature (°C)
Added ligand concentration (M)

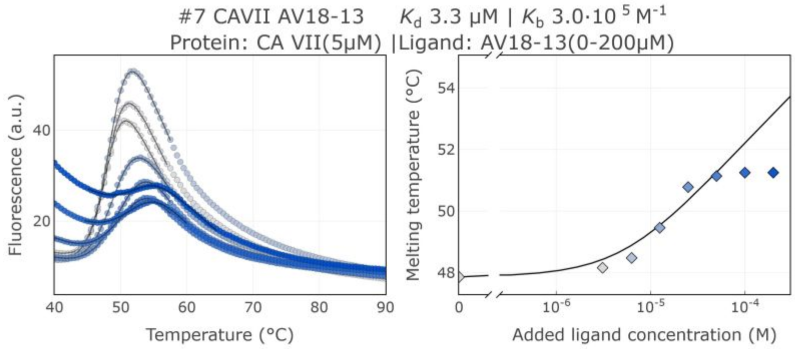
#7 CAVII AV18-13 K_d 3.3 μM | K_b 3.0·10 ^{5} M^{-1}
Protein: CA VII(5μM) |Ligand: AV18-13(0-200μM)
Fluorescence (a.u.)
Temperature (°C)
Melting temperature (°C)
Added ligand concentration (M)

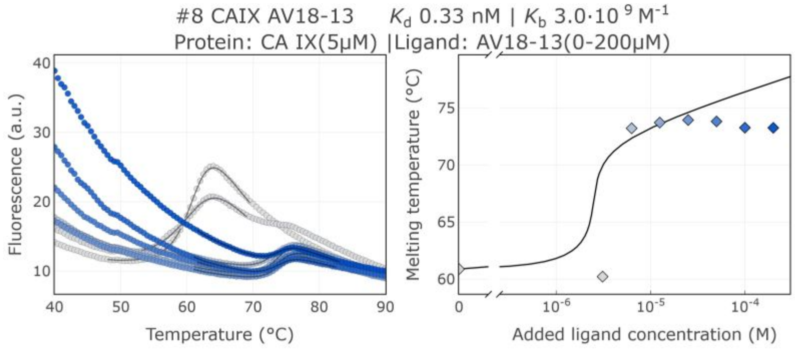
#8 CAIX AV18-13 K_d 0.33 nM | K_b 3.0·10 ^{9} M^{-1}
Protein: CA IX(5μM) |Ligand: AV18-13(0-200μM)
Fluorescence (a.u.)
Temperature (°C)
Melting temperature (°C)
Added ligand concentration (M)

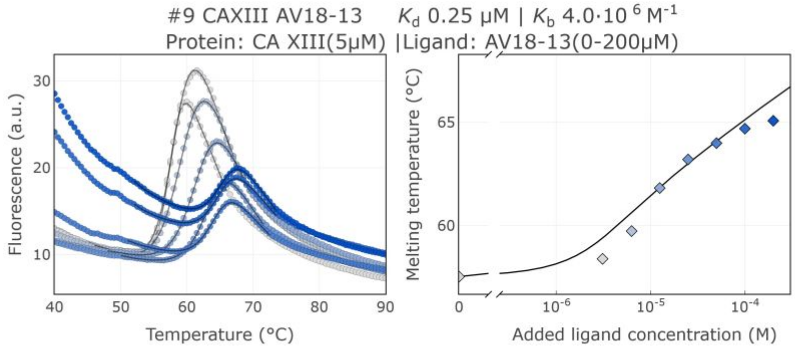
#9 CAXIII AV18-13 K_d 0.25 μM | K_b 4.0·10 ^{6} M^{-1}
Protein: CA XIII(5μM) |Ligand: AV18-13(0-200μM)
Fluorescence (a.u.)
Temperature (°C)
Melting temperature (°C)
Added ligand concentration (M)

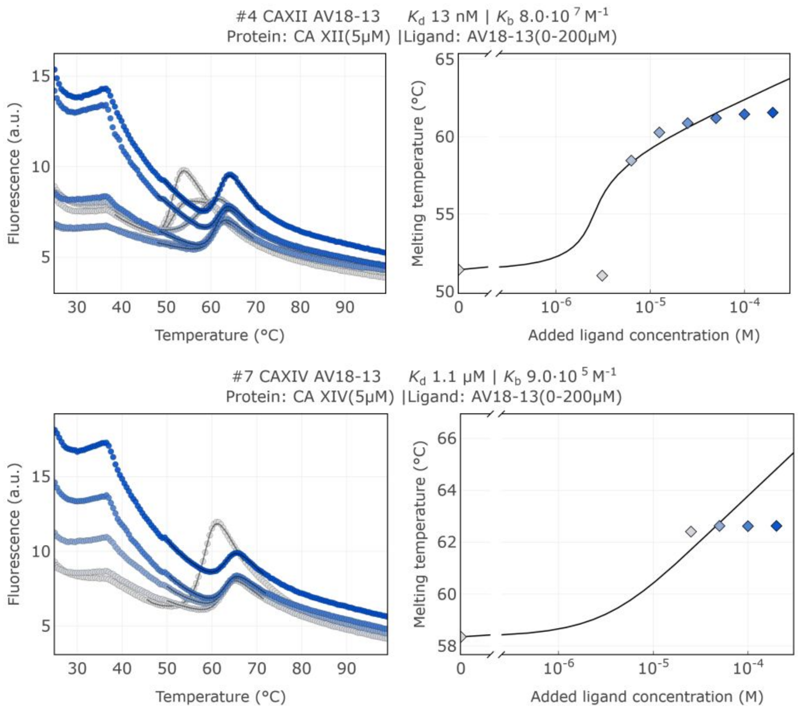

Figure S18. Compound 21 (MKV-549) binding to CA isozymes

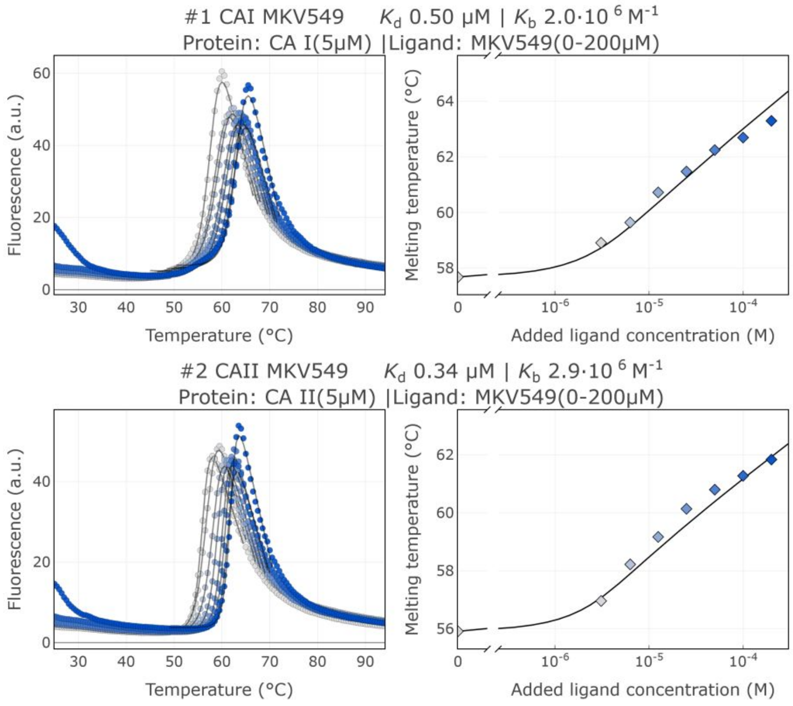

#1 CA III MKV-549 K_d 0.20 mM | K_b $5.0\cdot10^{3}$ M^{-1}
Protein: CA III(5µM) |Ligand: MKV-549(0-200µM)

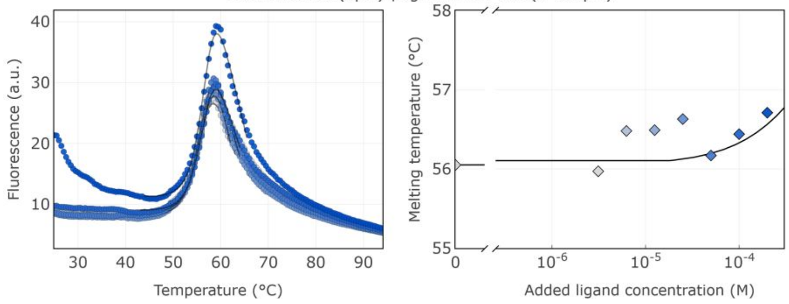

#3 CA IV MKV-549 K_d 0.60 µM | K_b $1.7\cdot10^{6}$ M^{-1}
Protein: CA IV(10µM) |Ligand: MKV-549(0-200µM)

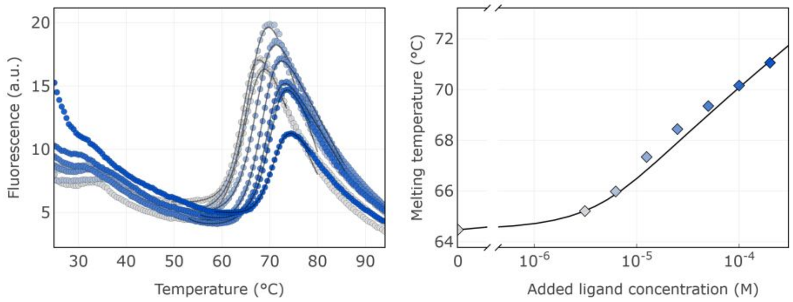

#3 CAVA (40-305) MKV549 K_d 0 M | K_b 0 M^{-1}
Protein: CA VA(5µM) |Ligand: MKV549(0-200µM)

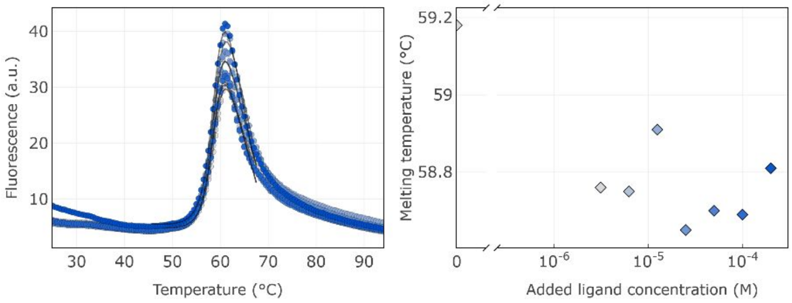

#3 CAVB MKV549 K_d 1.3 µM | K_b $8.0\cdot10^{5}$ M^{-1}
Protein: CA VB(5µM) |Ligand: MKV549(0-200µM)

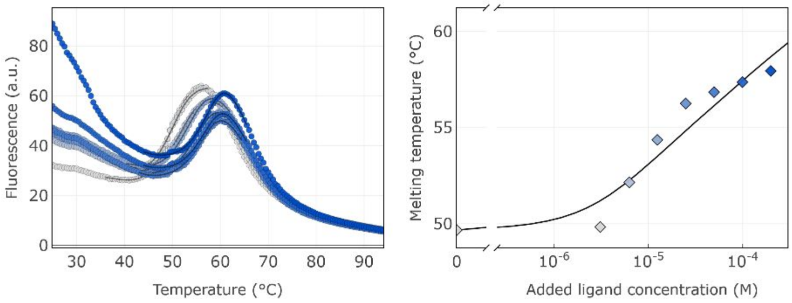

#4 CA VI MKV-549 K_d 0.33 μM | K_b $3.0 \cdot 10^6$ M^{-1}
Protein: CA VI(5μM) |Ligand: MKV-549(0-200μM)

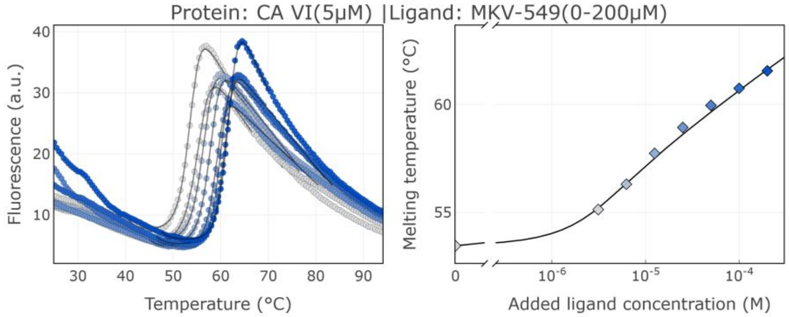

#5 CA VII MKV-549 K_d 0.54 μM | K_b $1.9 \cdot 10^6$ M^{-1}
Protein: CA VII(5μM) |Ligand: MKV-549(0-200μM)

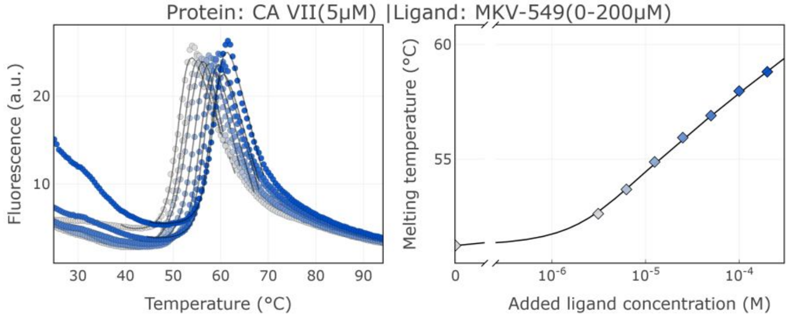

#4 CAIX MKV549 K_d 0.17 nM | K_b $6.0 \cdot 10^9$ M^{-1}
Protein: CA IX(5μM) |Ligand: MKV549(0-200μM)

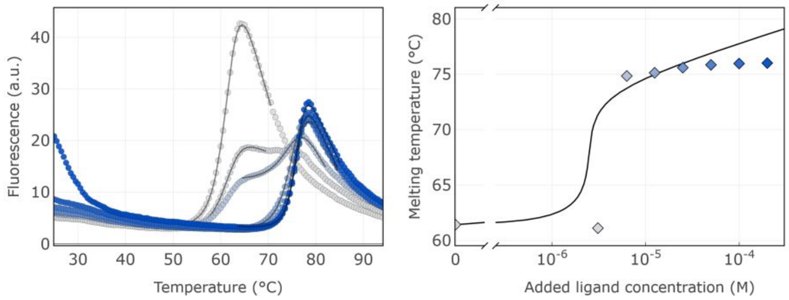

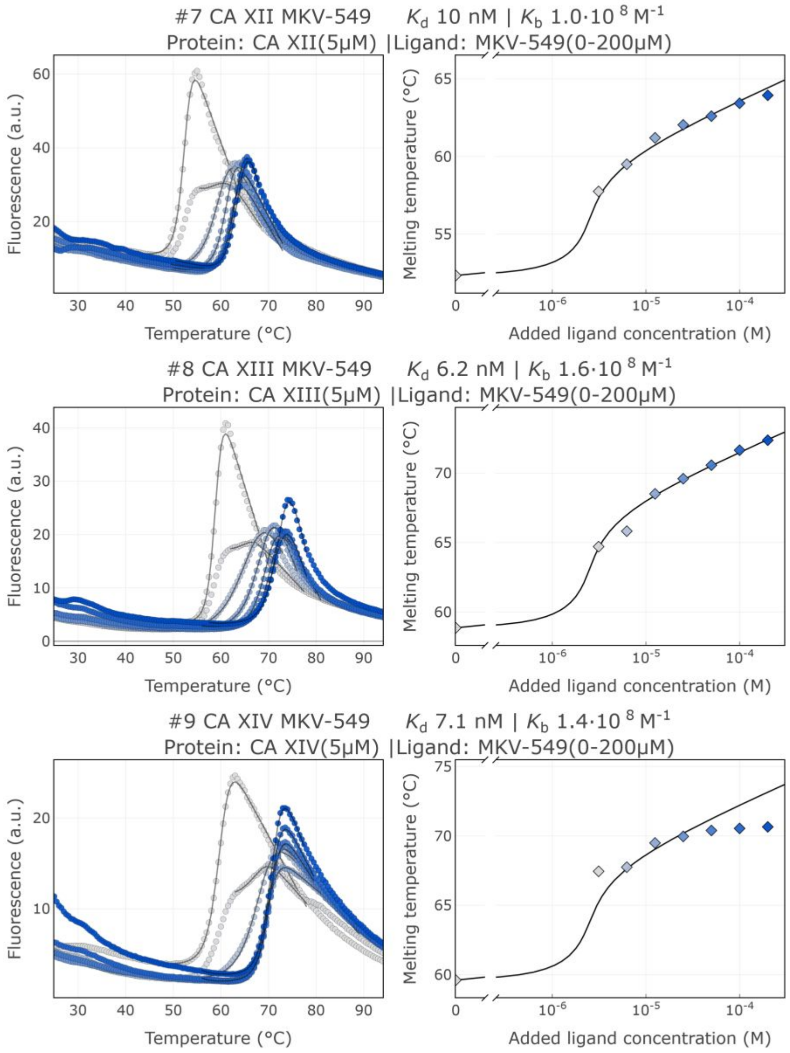
#7 CA XII MKV-549 K_d 10 nM | K_b 1.0·10 ^{8} M^{-1}
Protein: CA XII(5μM) |Ligand: MKV-549(0-200μM)
Fluorescence (a.u.)
Temperature (°C)
Melting temperature (°C)
Added ligand concentration (M)
#8 CA XIII MKV-549 K_d 6.2 nM | K_b 1.6·10 ^{8} M^{-1}
Protein: CA XIII(5μM) |Ligand: MKV-549(0-200μM)
Fluorescence (a.u.)
Temperature (°C)
Melting temperature (°C)
Added ligand concentration (M)
#9 CA XIV MKV-549 K_d 7.1 nM | K_b 1.4·10 ^{8} M^{-1}
Protein: CA XIV(5μM) |Ligand: MKV-549(0-200μM)
Fluorescence (a.u.)
Temperature (°C)
Melting temperature (°C)
Added ligand concentration (M)

Figure S19. Compound 24 (AV18-28) binding to CA isozymes

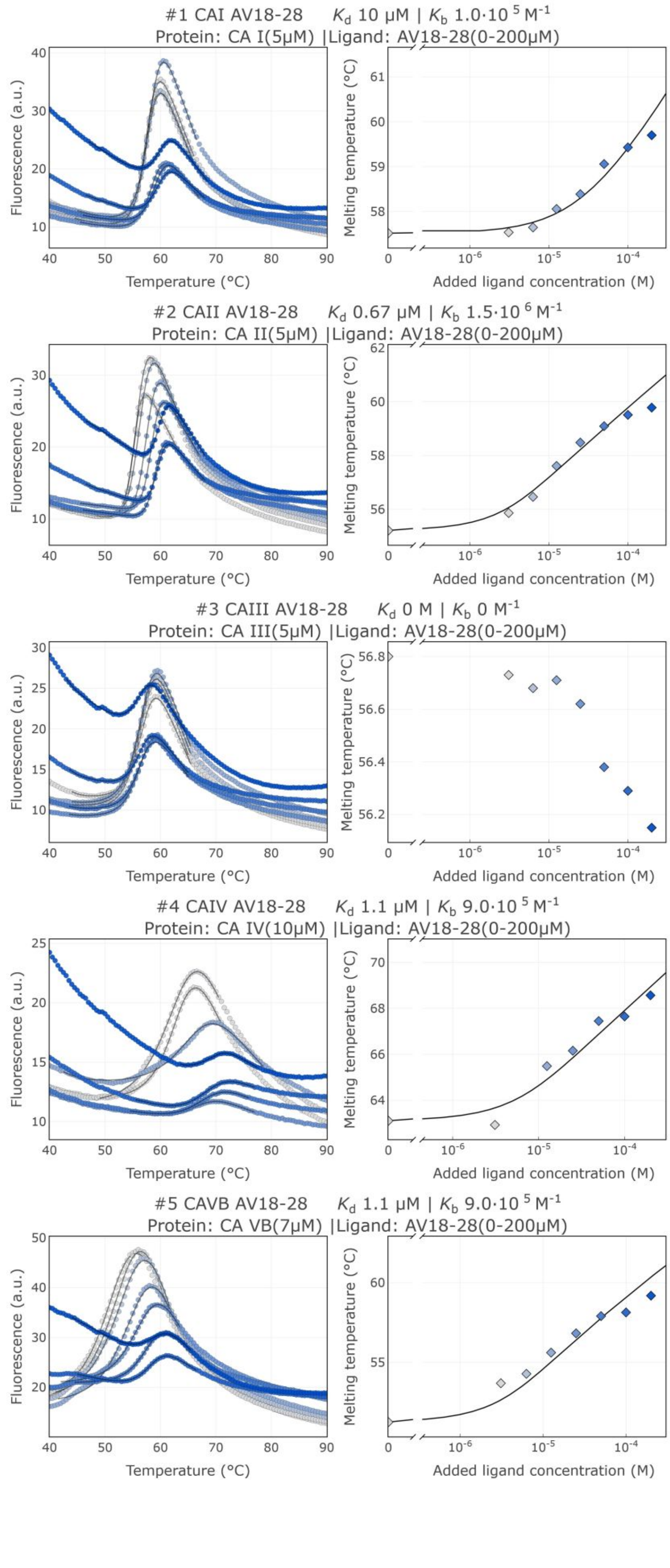

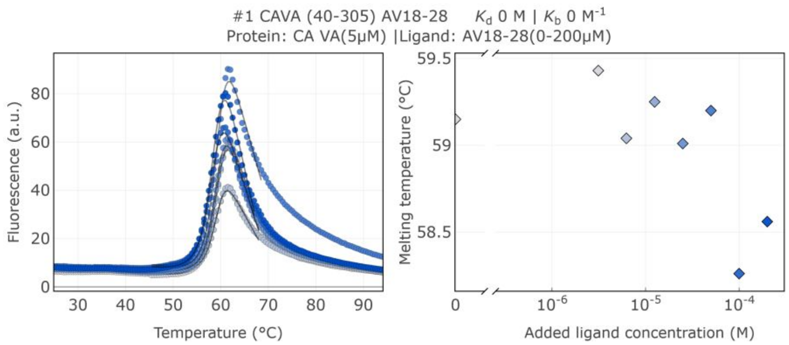
#1 CAVA (40-305) AV18-28 K_d 0 M | K_b 0 M^{-1}
Protein: CA VA(5μM) |Ligand: AV18-28(0-200μM)
Fluorescence (a.u.)
Temperature (°C)
Melting temperature (°C)
Added ligand concentration (M)

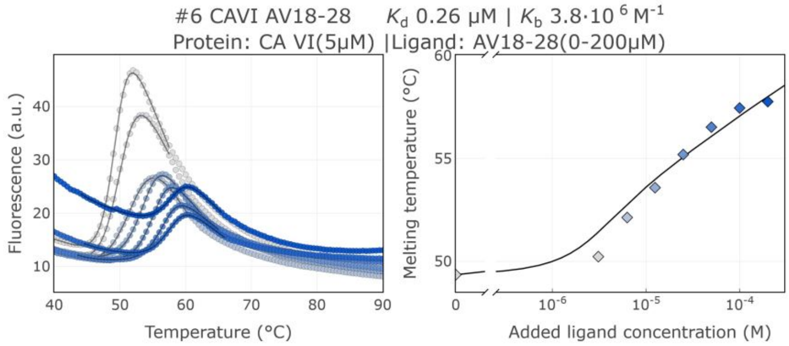
#6 CAVI AV18-28 K_d 0.26 μM | K_b 3.8·10 ^{6} M^{-1}
Protein: CA VI(5μM) |Ligand: AV18-28(0-200μM)
Fluorescence (a.u.)
Temperature (°C)
Melting temperature (°C)
Added ligand concentration (M)

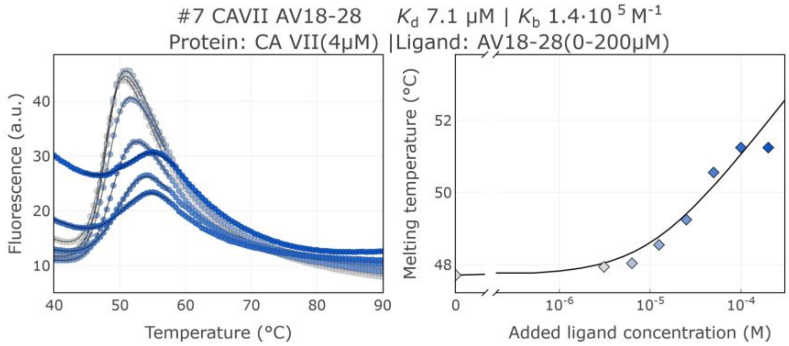
#7 CAVII AV18-28 K_d 7.1 μM | K_b 1.4·10 ^{5} M^{-1}
Protein: CA VII(4μM) |Ligand: AV18-28(0-200μM)
Fluorescence (a.u.)
Temperature (°C)
Melting temperature (°C)
Added ligand concentration (M)

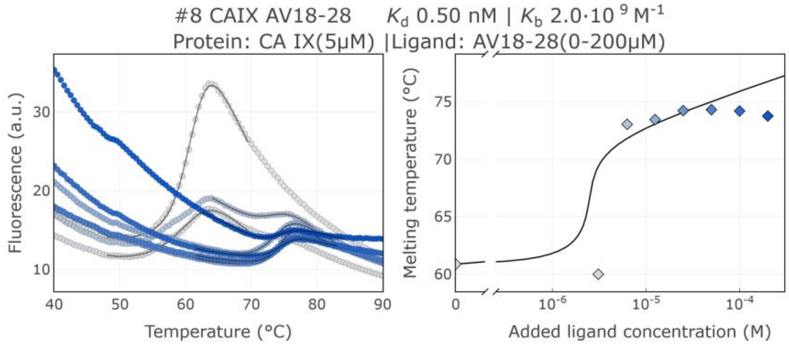
#8 CAIX AV18-28 K_d 0.50 nM | K_b 2.0·10 ^{9} M^{-1}
Protein: CA IX(5μM) |Ligand: AV18-28(0-200μM)
Fluorescence (a.u.)
Temperature (°C)
Melting temperature (°C)
Added ligand concentration (M)

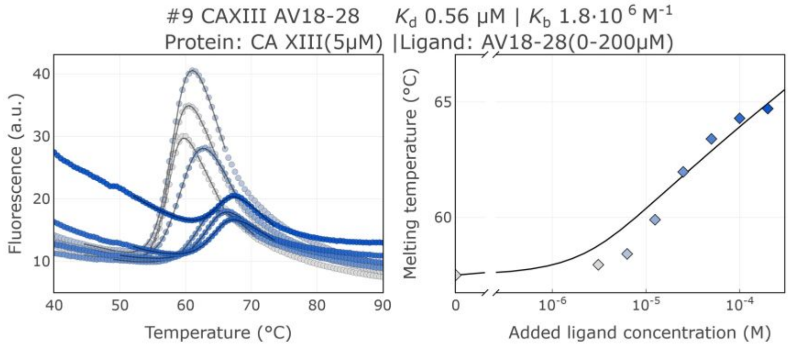
#9 CAXIII AV18-28 K_d 0.56 μM | K_b 1.8·10 ^{6} M^{-1}
Protein: CA XIII(5μM) |Ligand: AV18-28(0-200μM)
Fluorescence (a.u.)
Temperature (°C)
Melting temperature (°C)
Added ligand concentration (M)

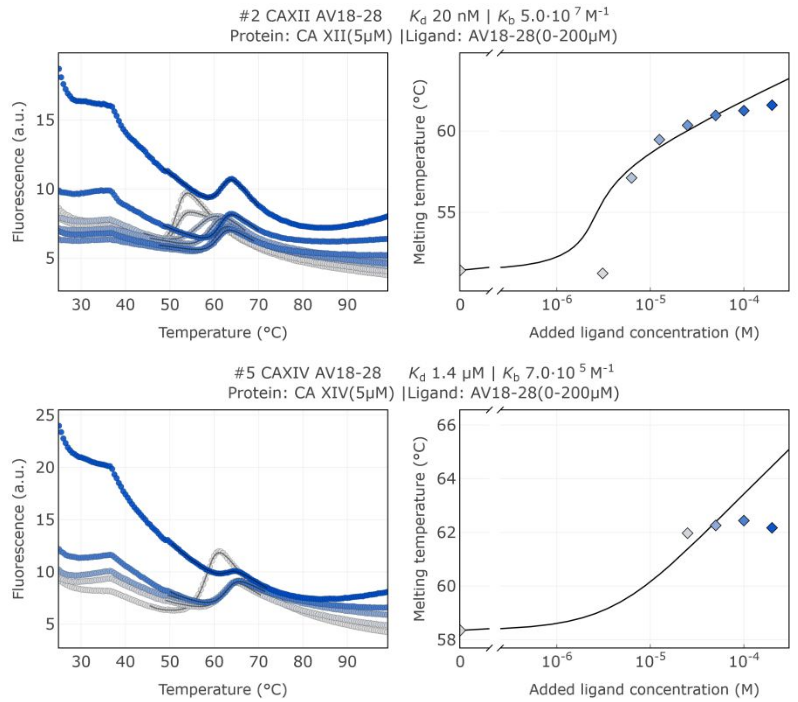

Figure S20. Compound 26 (MZ24-71) binding to CA isozymes

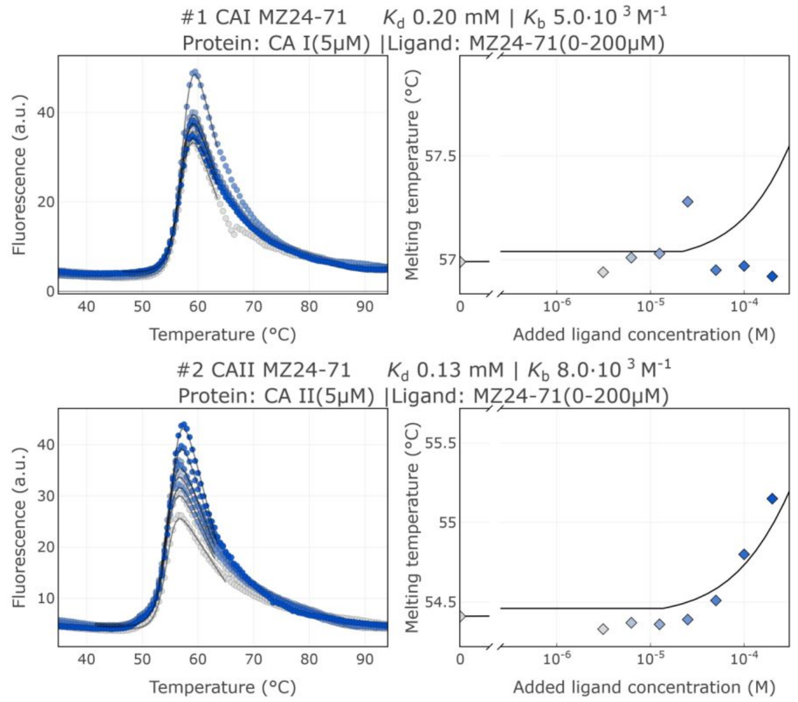

#6 CAIV MZ24-71 K_d 0.20 mM | K_b $5.0 \cdot 10^{3}$ M^{-1}
Protein: CA IV(10μM) |Ligand: MZ24-71(0-200μM)

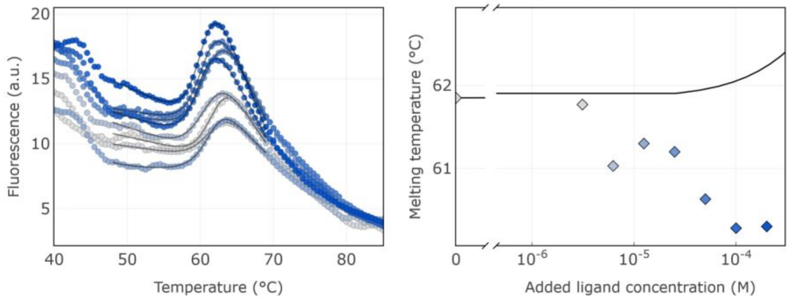

#1 CAVA MZ24-71 K_d 0.20 mM | K_b $5.0 \cdot 10^{3}$ M^{-1}
Protein: CA VA(5μM) |Ligand: MZ24-71(0-200μM)

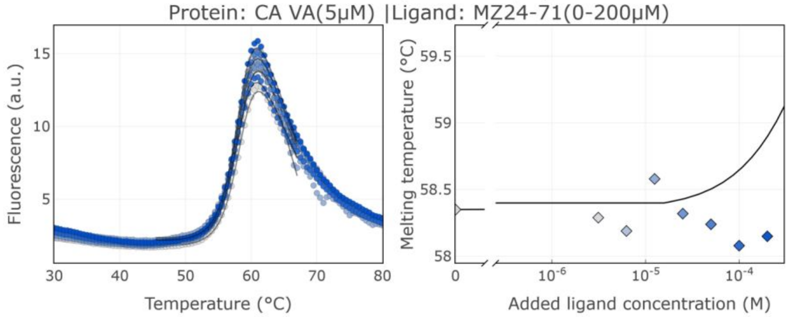

#2 CAVB MZ24-71 K_d 69 μM | K_b $1.5 \cdot 10^{4}$ M^{-1}
Protein: CA VB(5μM) |Ligand: MZ24-71(0-200μM)

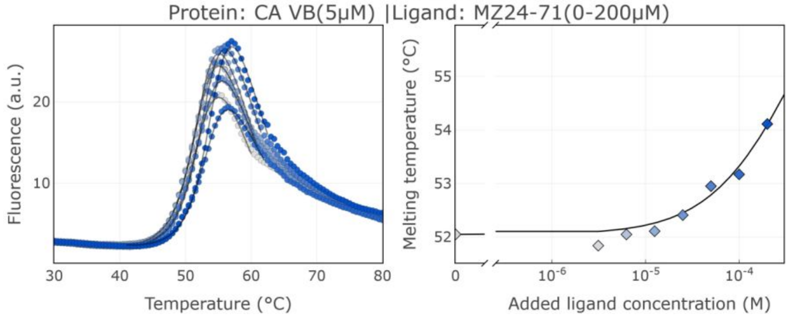

#1 CAVI MZ24-71 K_d 3.3 μM | K_b $3.0 \cdot 10^{5}$ M^{-1}
Protein: CA VI(5μM) |Ligand: MZ24-71(0-200μM)

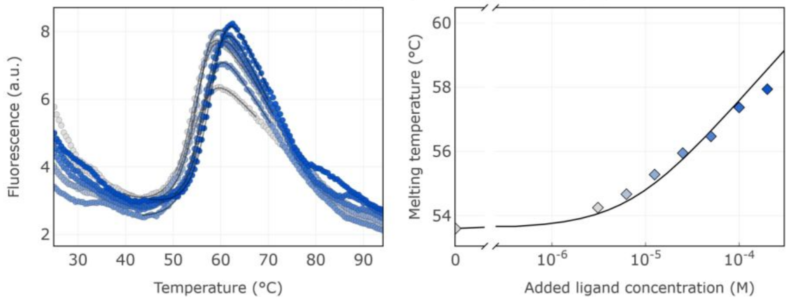

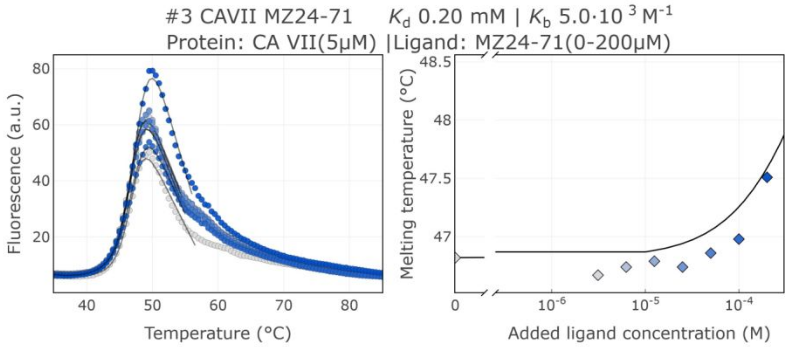
#3 CAVII MZ24-71 K_d 0.20 mM | K_b 5.0·10 ^{3} M^{-1}
Protein: CA VII(5μM) |Ligand: MZ24-71(0-200μM)
Fluorescence (a.u.)
Temperature (°C)
Melting temperature (°C)
Added ligand concentration (M)

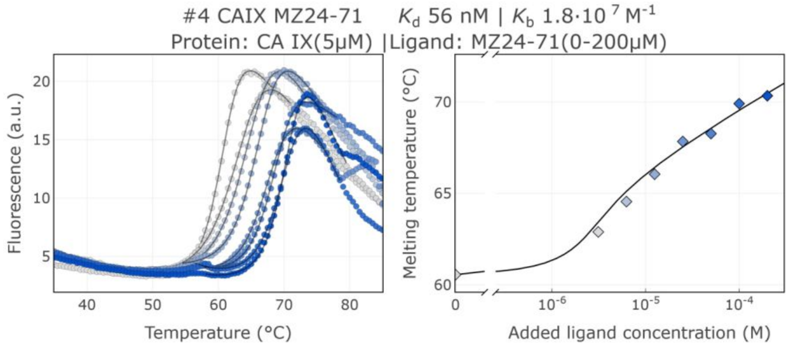
#4 CAIX MZ24-71 K_d 56 nM | K_b 1.8·10 ^{7} M^{-1}
Protein: CA IX(5μM) |Ligand: MZ24-71(0-200μM)
Fluorescence (a.u.)
Temperature (°C)
Melting temperature (°C)
Added ligand concentration (M)

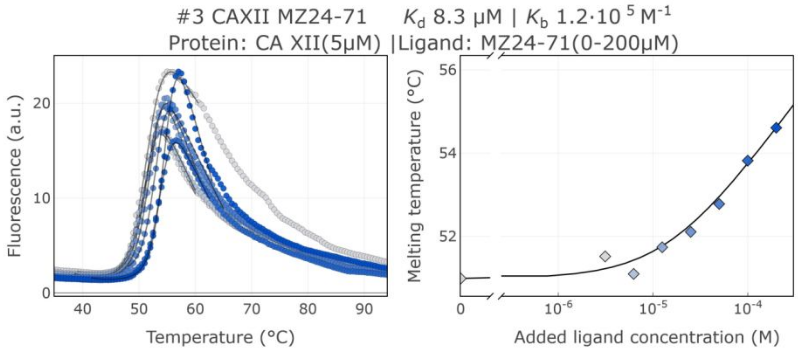
#3 CAXII MZ24-71 K_d 8.3 μM | K_b 1.2·10 ^{5} M^{-1}
Protein: CA XII(5μM) |Ligand: MZ24-71(0-200μM)
Fluorescence (a.u.)
Temperature (°C)
Melting temperature (°C)
Added ligand concentration (M)

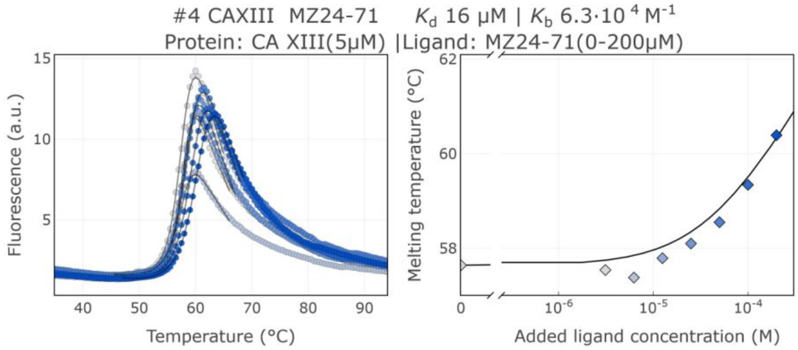
#4 CAXIII MZ24-71 K_d 16 μM | K_b 6.3·10 ^{4} M^{-1}
Protein: CA XIII(5μM) |Ligand: MZ24-71(0-200μM)
Fluorescence (a.u.)
Temperature (°C)
Melting temperature (°C)
Added ligand concentration (M)

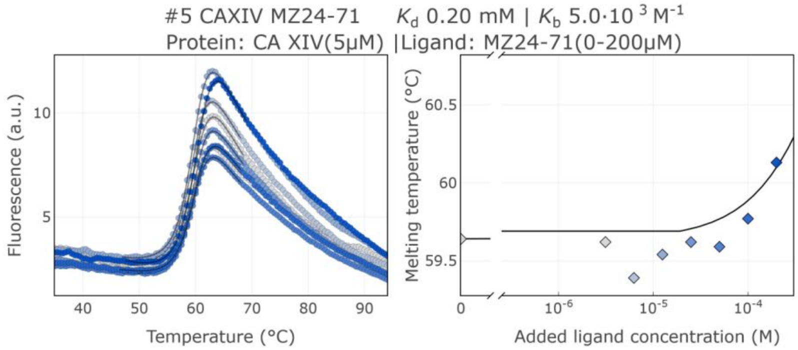
#5 CAXIV MZ24-71 K_d 0.20 mM | K_b 5.0·10 ^{3} M^{-1}
Protein: CA XIV(5μM) |Ligand: MZ24-71(0-200μM)
Fluorescence (a.u.)
Temperature (°C)
Melting temperature (°C)
Added ligand concentration (M)

Figure S21. Compound 27 (MZ24-75) binding to CA isozymes

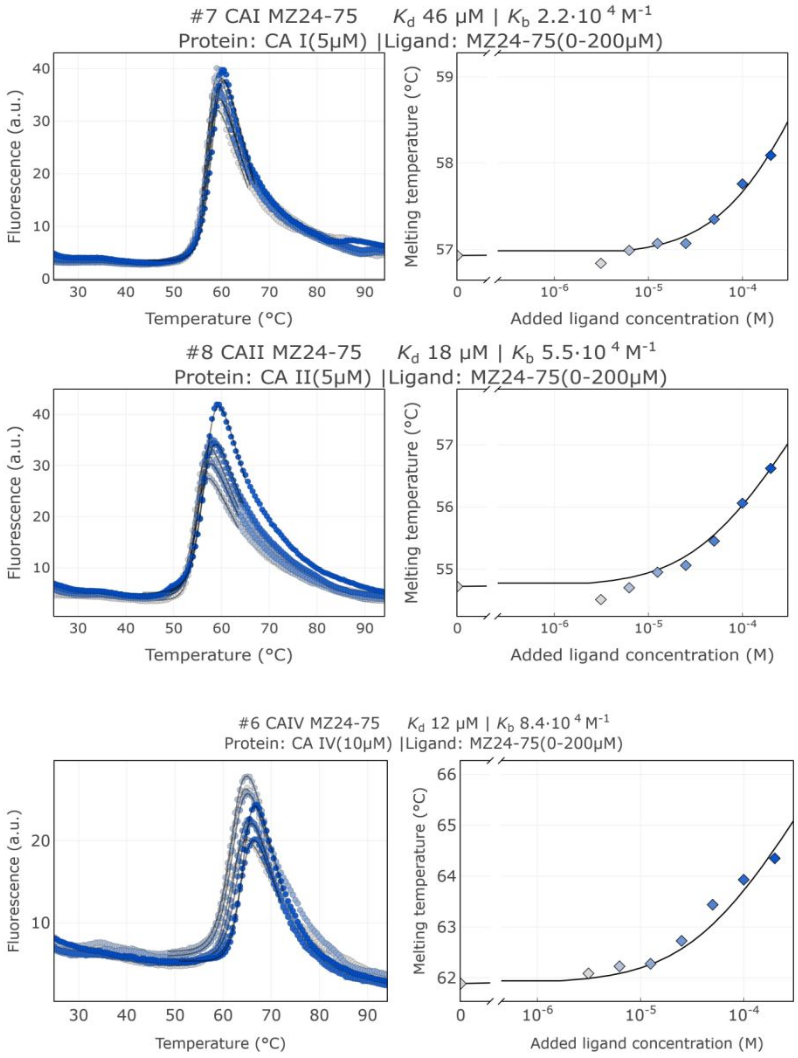

#1 CAVA MZ24-75 K_d 0.20 mM | K_b 5.0·10^{3} M^{-1}
Protein: CA VA(5μM) |Ligand: MZ24-75(0-200μM)

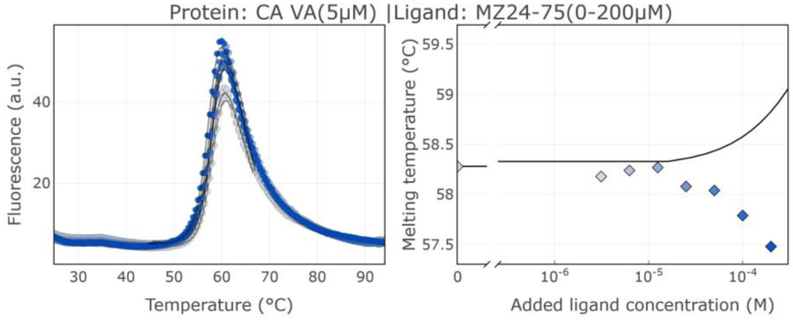

#2 CAVB MZ24-75 K_d 8.3 μM | K_b 1.2·10^{5} M^{-1}
Protein: CA VB(5μM) |Ligand: MZ24-75(0-200μM)

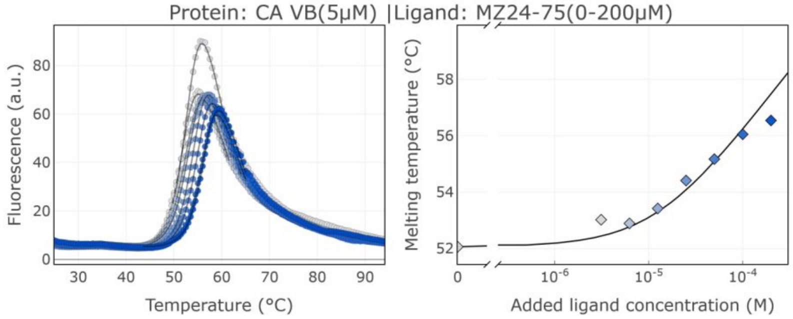

#2 CAVI MZ24-75 K_d 1.4 μM | K_b 7.0·10^{5} M^{-1}
Protein: CA VI(5μM) |Ligand: MZ24-75(0-200μM)

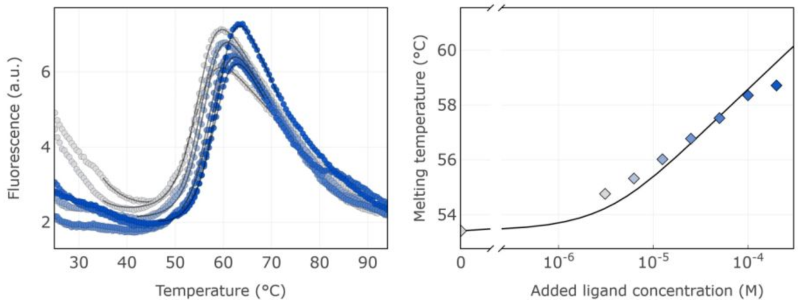

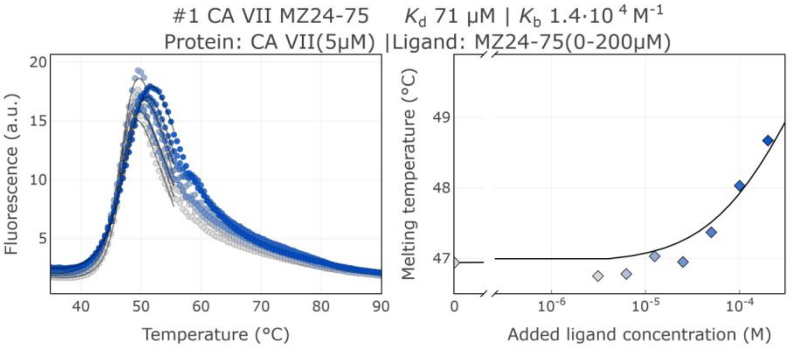
#1 CA VII MZ24-75 K_d 71 µM | K_b $1.4\cdot10^{4}$ M^{-1}
Protein: CA VII(5µM) |Ligand: MZ24-75(0-200µM)
Fluorescence (a.u.)
Temperature (°C)
Melting temperature (°C)
Added ligand concentration (M)

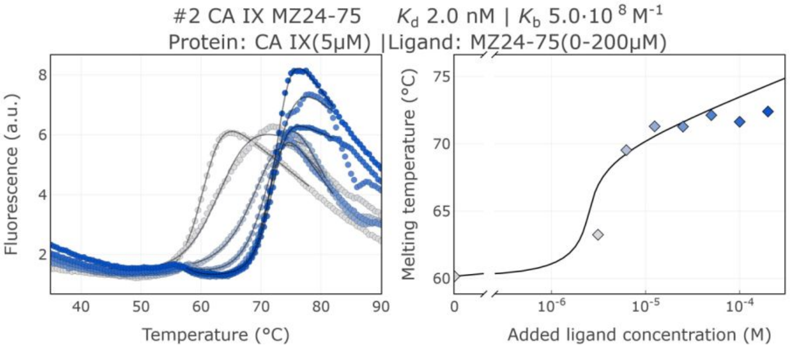
#2 CA IX MZ24-75 K_d 2.0 nM | K_b $5.0\cdot10^{8}$ M^{-1}
Protein: CA IX(5µM) |Ligand: MZ24-75(0-200µM)
Fluorescence (a.u.)
Temperature (°C)
Melting temperature (°C)
Added ligand concentration (M)

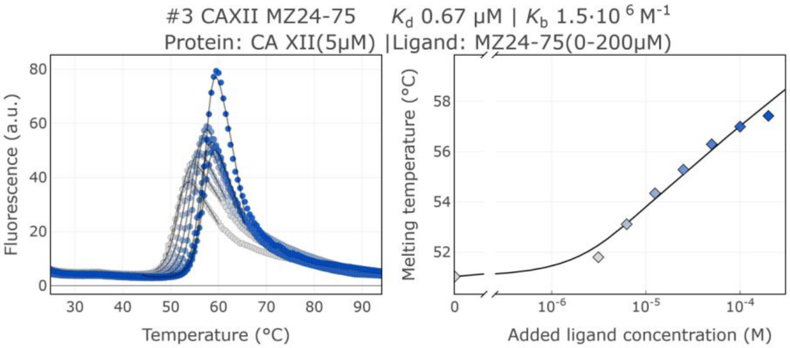
#3 CAXII MZ24-75 K_d 0.67 µM | K_b $1.5\cdot10^{6}$ M^{-1}
Protein: CA XII(5µM) |Ligand: MZ24-75(0-200µM)
Fluorescence (a.u.)
Temperature (°C)
Melting temperature (°C)
Added ligand concentration (M)

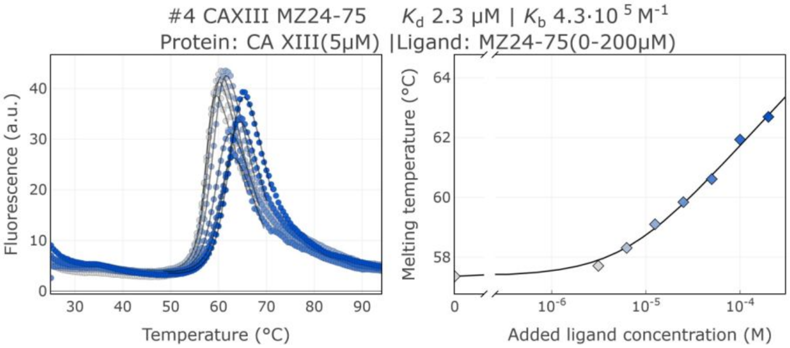
#4 CAXIII MZ24-75 K_d 2.3 µM | K_b $4.3\cdot10^{5}$ M^{-1}
Protein: CA XIII(5µM) |Ligand: MZ24-75(0-200µM)
Fluorescence (a.u.)
Temperature (°C)
Melting temperature (°C)
Added ligand concentration (M)

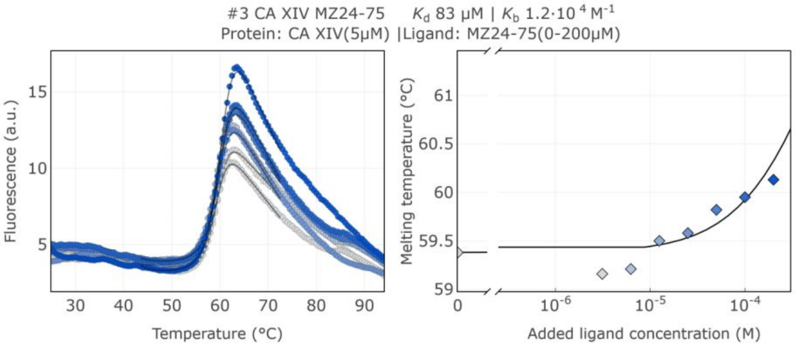
#3 CA XIV MZ24-75 K_d 83 µM | K_b $1.2\cdot10^{4}$ M^{-1}
Protein: CA XIV(5µM) |Ligand: MZ24-75(0-200µM)
Fluorescence (a.u.)
Temperature (°C)
Melting temperature (°C)
Added ligand concentration (M)

Figure S22. Compound 28 (MZ24-76) binding to CA isozymes

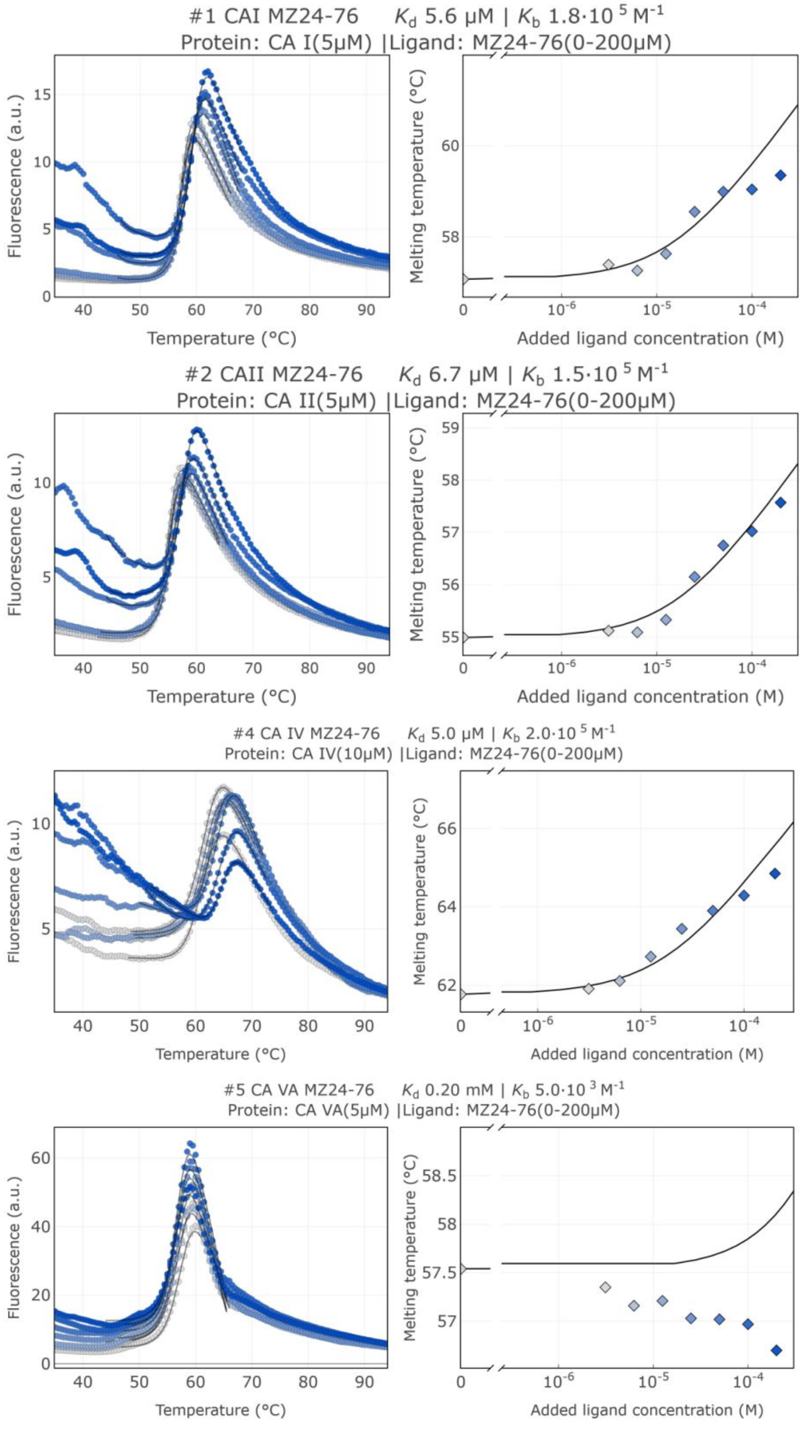

#9 CAVB MZ24-76 K_d 10 µM | K_b 1.0·10^5 M^{-1}
Protein: CA VB(5µM) |Ligand: MZ24-76(0-200µM)

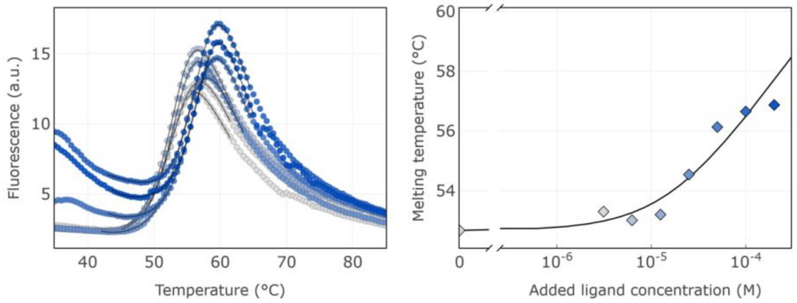

#3 CAVI MZ24-76 K_d 1.0 µM | K_b 1.0·10^6 M^{-1}
Protein: CA VI(5µM) |Ligand: MZ24-76(0-200µM)

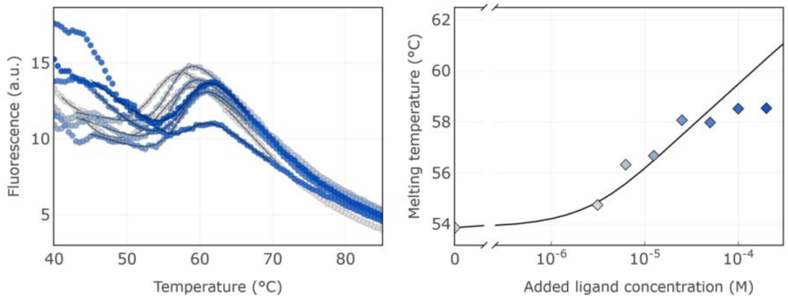

#3 CAVII MZ24-76 K_d 67 µM | K_b 1.5·10^4 M^{-1}
Protein: CA VII(5µM) |Ligand: MZ24-76(0-200µM)

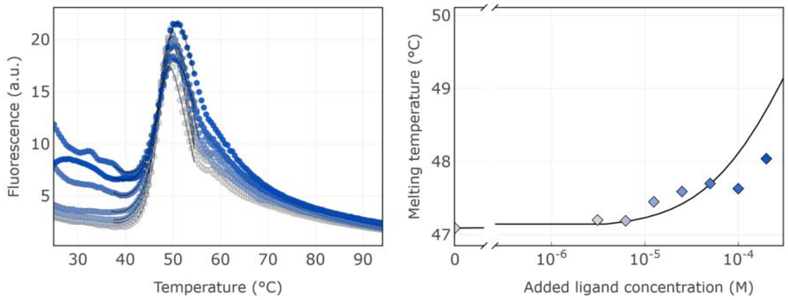

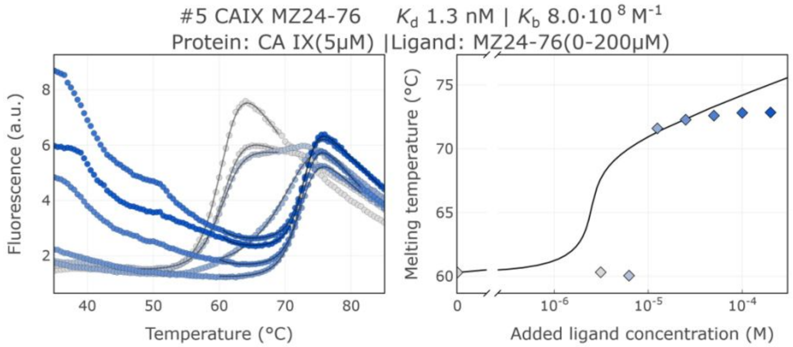
#5 CAIX MZ24-76 K_d 1.3 nM | K_b 8.0·10^8 M^{-1}
Protein: CA IX(5μM) |Ligand: MZ24-76(0-200μM)
Fluorescence (a.u.)
Temperature (°C)
Melting temperature (°C)
Added ligand concentration (M)

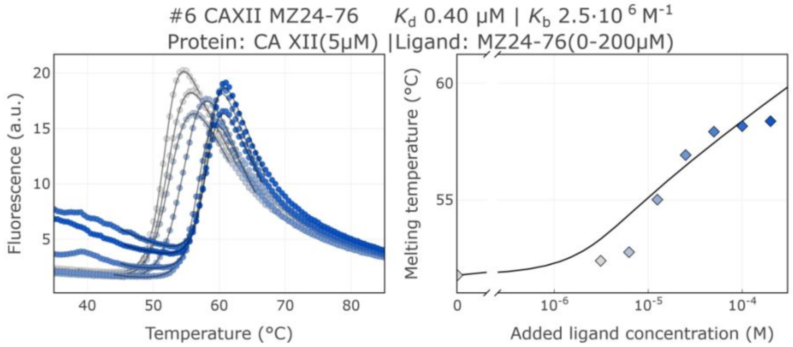
#6 CAXII MZ24-76 K_d 0.40 μM | K_b 2.5·10^6 M^{-1}
Protein: CA XII(5μM) |Ligand: MZ24-76(0-200μM)
Fluorescence (a.u.)
Temperature (°C)
Melting temperature (°C)
Added ligand concentration (M)

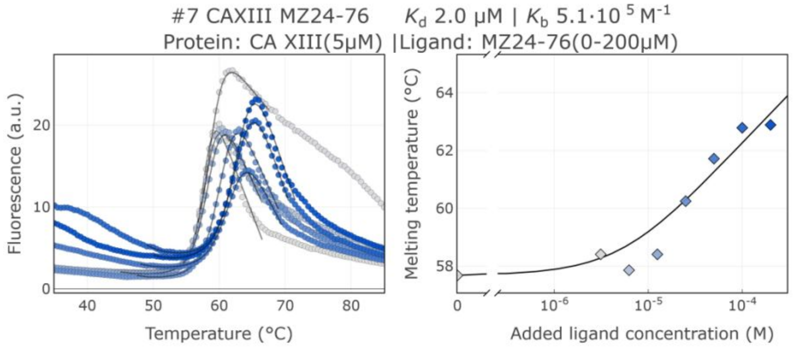
#7 CAXIII MZ24-76 K_d 2.0 μM | K_b 5.1·10^5 M^{-1}
Protein: CA XIII(5μM) |Ligand: MZ24-76(0-200μM)
Fluorescence (a.u.)
Temperature (°C)
Melting temperature (°C)
Added ligand concentration (M)

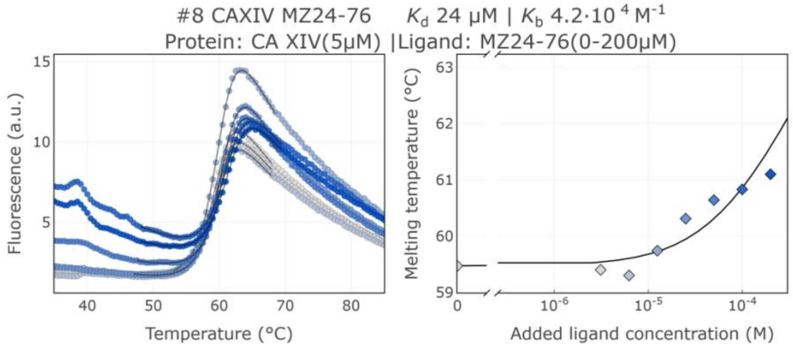
#8 CAXIV MZ24-76 K_d 24 μM | K_b 4.2·10^4 M^{-1}
Protein: CA XIV(5μM) |Ligand: MZ24-76(0-200μM)
Fluorescence (a.u.)
Temperature (°C)
Melting temperature (°C)
Added ligand concentration (M)

Figure S23. Compound 29 (MZ24-93) binding to CA isozymes

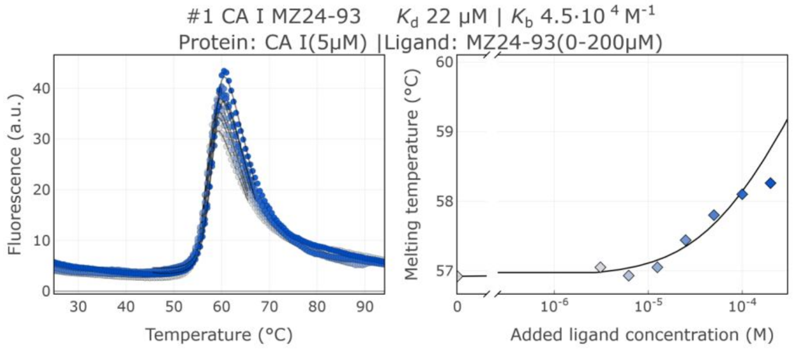

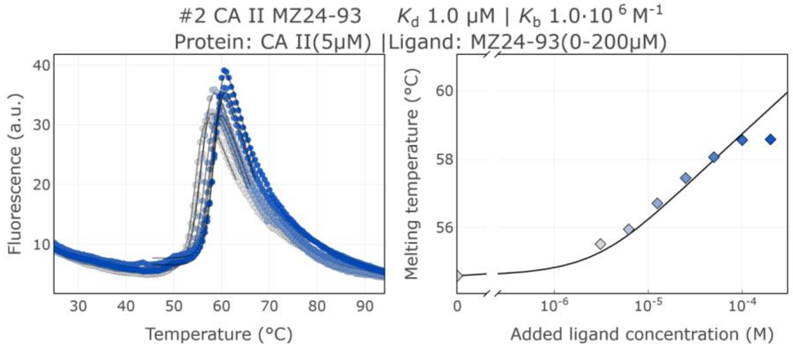

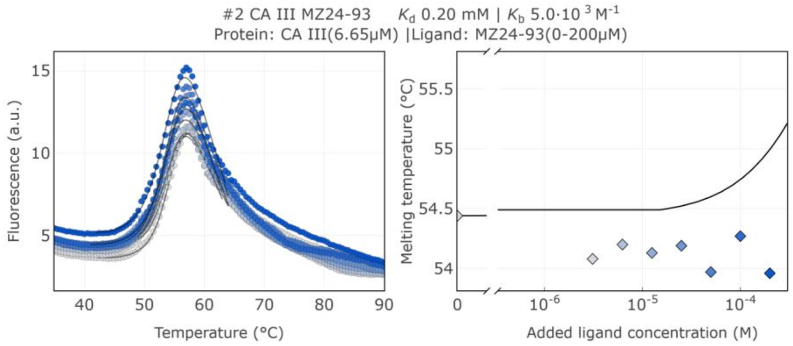

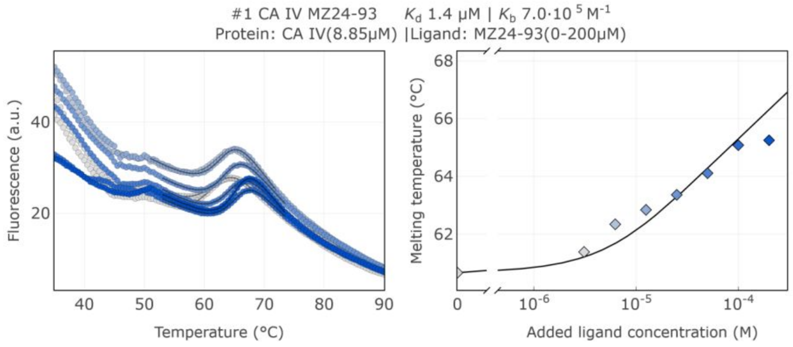

#1 CA VA MZ24-93 K_d 0.20 mM | K_b $5.0 \cdot 10^{3}$ M^{-1}
Protein: CA VA(5μM) |Ligand: MZ24-93(0-200μM)

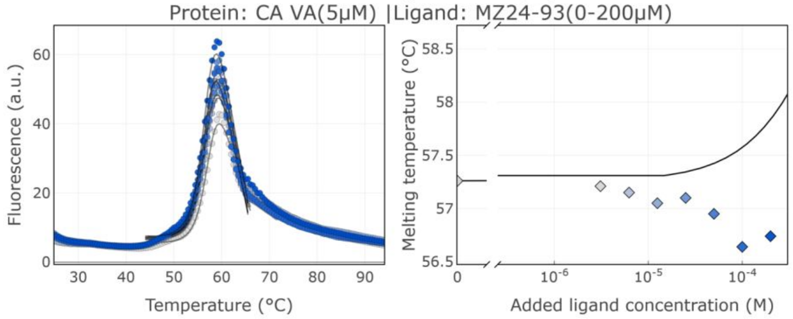

#2 CA VB MZ24-93 K_d 0.61 μM | K_b $1.6 \cdot 10^{6}$ M^{-1}
Protein: CA VB(5μM) |Ligand: MZ24-93(0-200μM)

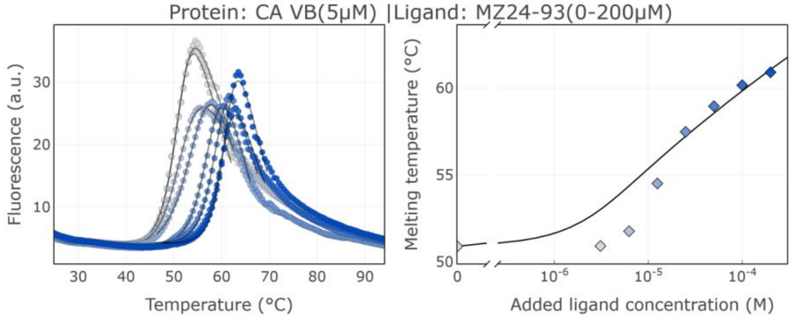

#3 CA VI MZ24-93 K_d 5.0 μM | K_b $2.0 \cdot 10^{5}$ M^{-1}
Protein: CA VI(5μM) |Ligand: MZ24-93(0-200μM)

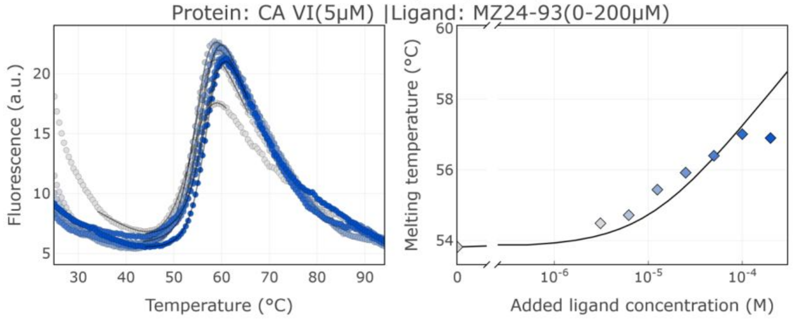

#4 CA VII MZ24-93 K_d 14 μM | K_b $7.1 \cdot 10^{4}$ M^{-1}
Protein: CA VII(5μM) |Ligand: MZ24-93(0-200μM)

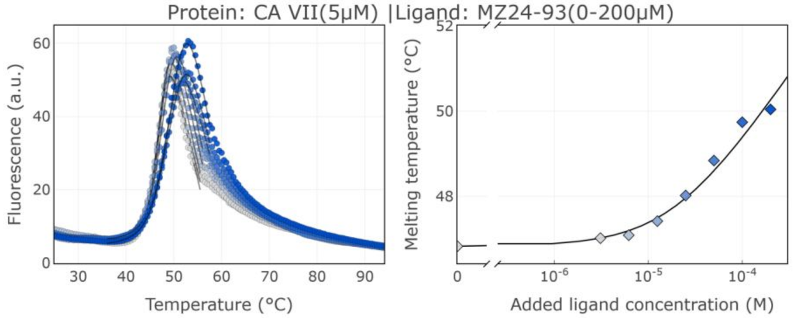

#5 CA IX MZ24-93 K_d 0.20 nM | K_b $5.0 \cdot 10^{9}$ M^{-1}
Protein: CA IX(5μM) |Ligand: MZ24-93(0-200μM)

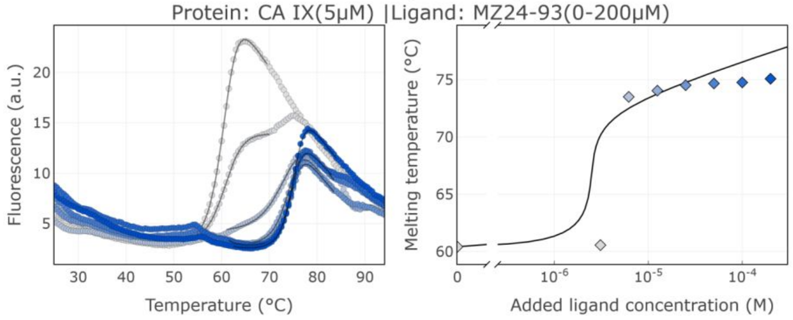

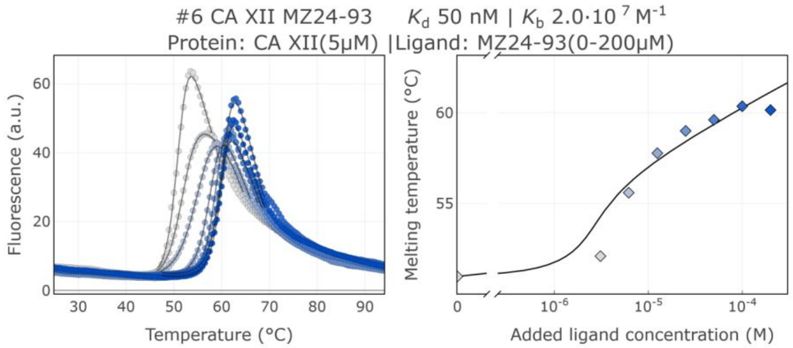
#6 CA XII MZ24-93 K_d 50 nM | K_b $2.0 \cdot 10^7$ M^{-1}
Protein: CA XII(5μM) |Ligand: MZ24-93(0-200μM)
Fluorescence (a.u.)
Temperature (°C)
Melting temperature (°C)
Added ligand concentration (M)

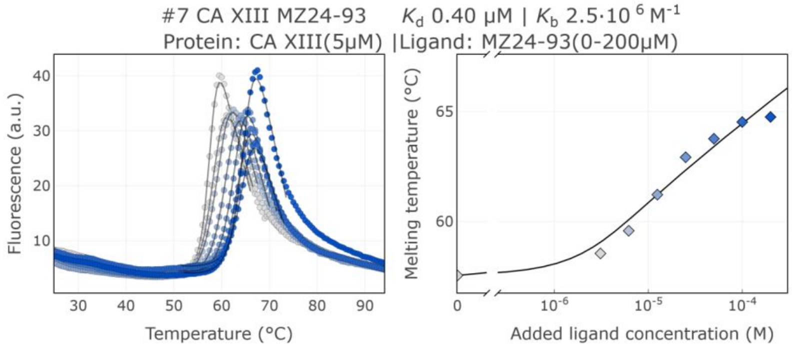
#7 CA XIII MZ24-93 K_d 0.40 μM | K_b $2.5 \cdot 10^6$ M^{-1}
Protein: CA XIII(5μM) |Ligand: MZ24-93(0-200μM)
Fluorescence (a.u.)
Temperature (°C)
Melting temperature (°C)
Added ligand concentration (M)

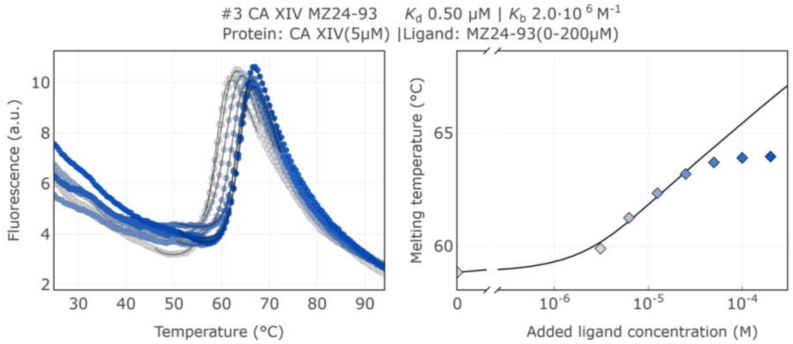
#3 CA XIV MZ24-93 K_d 0.50 μM | K_b $2.0 \cdot 10^6$ M^{-1}
Protein: CA XIV(5μM) |Ligand: MZ24-93(0-200μM)
Fluorescence (a.u.)
Temperature (°C)
Melting temperature (°C)
Added ligand concentration (M)

Table S1. Compound selectivity for CAIX is evaluated as the ratio of compound affinity towards off-target CA relative to CAIX isozyme

Cmpd	CAI/ CAIX	CAII/ CAIX	CAIII/ CAIX	CAIV/ CAIX	CAVA/ CAIX	CAVB/ CAIX	CAVI/ CAIX	CAVII/ CAIX	CAXII /CAIX	CAXIII/ CAIX	CAXIV /CAIX
	$K_{d,obs}$ ratio										
**4a**[1]	0.013	0.66	1000	66	5.8	1.3	17	0.44	10	0.5	0.54
**4b**[1]	0.004	0.34	660	3.2	5.8	0.44	1.3	0.14	5	0.58	0.10
**4c**	0.006	0.73	1100	31	22	0.37	19	0.37	9	0.93	0.73
**5a**	40000	1100	2000000	1400	67000	170	4000	250	100	220	420
**5b**[2]	14000	1200	800000	500	50000	110	1900	200	66	72	32
**5c**	59000	1600	≥9100000	5900	77000	290	7700	110	59	91	230
**6**	600000	4300	1400000	6800	≥17000000	7900	4300	6000	59	430	280
**7**	390000	4900	≥17000000	2800	≥17000000	5600	6900	8300	20	3100	24000
**8**	86000	1700	≥1400000	3200	≥1400000	8600	2600	5100	100	650	360
**9**	110000	28000	≥950000	62000	≥950000	3100	6700	19000	430	3000	12000
**10**	52000	5500	≥690000	29000	≥690000	14000	45	15000	120	4100	86000
**11**	13000	2000	≥100000	3800	≥100000	48000	250	42000	200	650	49000
**12**	210000	4100	400000	14000	≥20000000	6200	6700	2300	140	200	250
**13**	300000	1400	17000000	9100	≥43000000	630	18000	830	11	54	590
**14**	310000	1300	4700000	5600	≥44000000	8200	16000	1800	24	380	780
**15**	20000	2500	≥20000	≥20000	ND	≥20000	250	≥20000	250	500	≥20000
**16**	690000	2300	≥690000	690	ND	2700	1700	110000	69	4500	3100
**17**	91000	1100	270000	1400	≥2200000	3600	3200	2200	12	910	280
**18**	78000	2100	650000	ND	≥5000000	16000	ND	2500	25	550	480
**19**	39000	1300	2400000	ND	≥3000000	14000	ND	4500	16	450	900
**20**	11000	2300	≥910000	1800	≥910000	6400	1300	30000	59	1500	5000
**21**	2700	2700	≥1400000	4200	≥1400000	6200	4100	4000	71	26	61
**24**	33000	2700	≥800000	2000	ND	5600	1300	52000	80	2000	5600
**26**	3600	2300	ND	≥3600	≥3600	1200	59	≥3600	150	290	≥3600
**27**	23000	9000	ND	6000	≥100000	4200	700	36000	340	1200	42000
**28**	4300	5200	ND	3800	≥150000	7700	770	52000	310	1500	18000
**29**	110000	5000	≥1000000	7000	≥1000000	3100	25000	70000	250	2000	2500

pK_a values of sulfonamide amino group

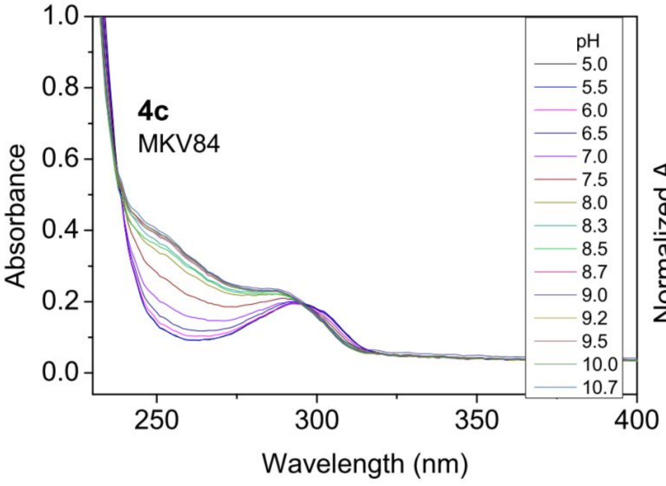

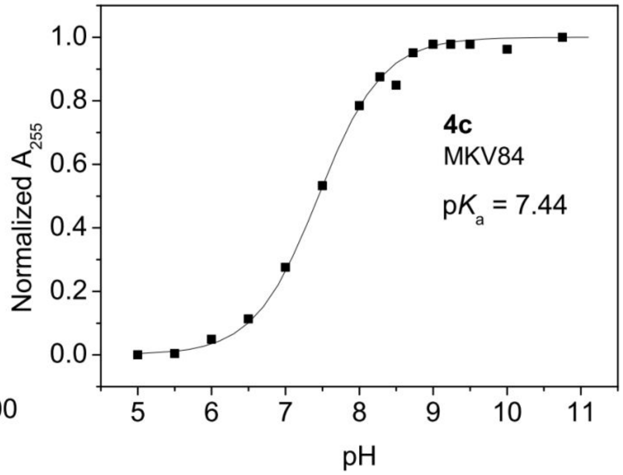

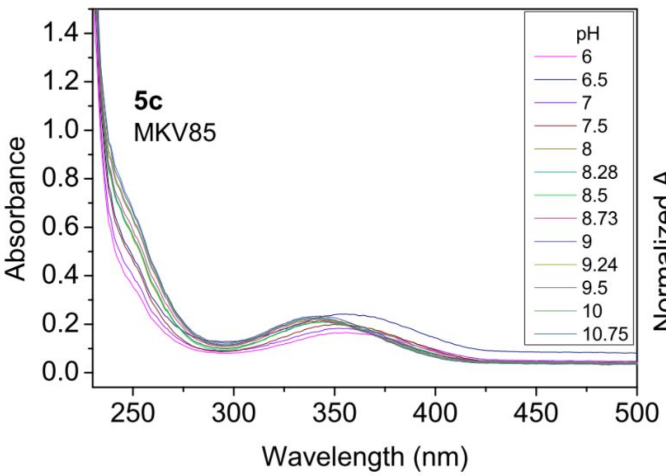

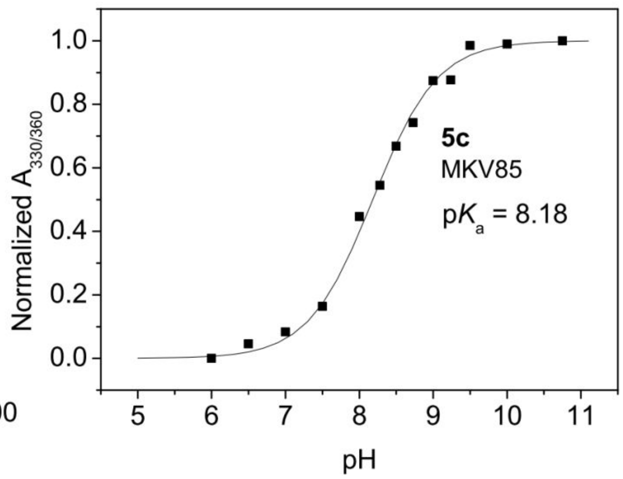

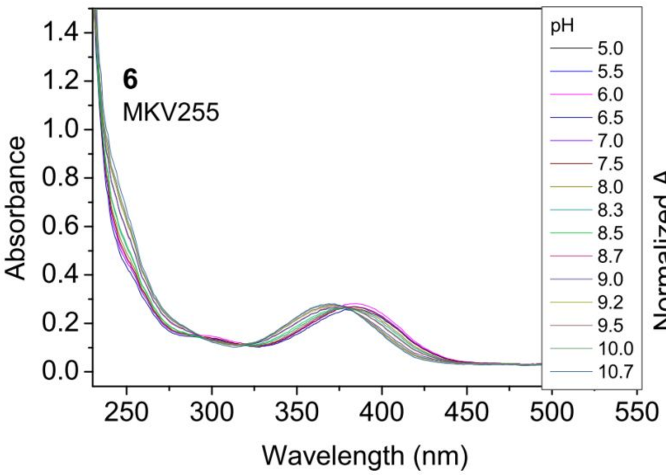

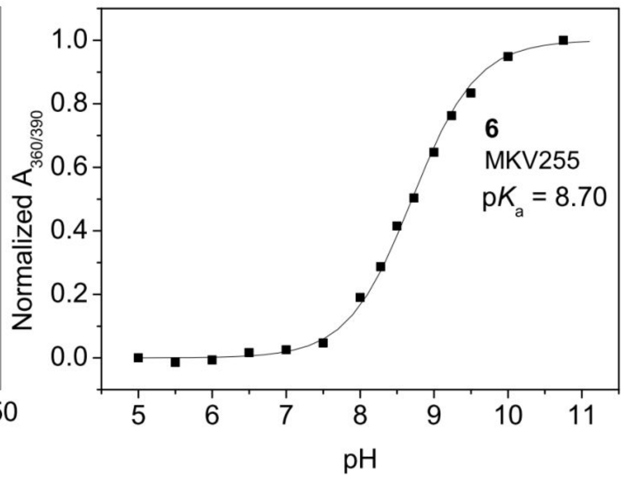

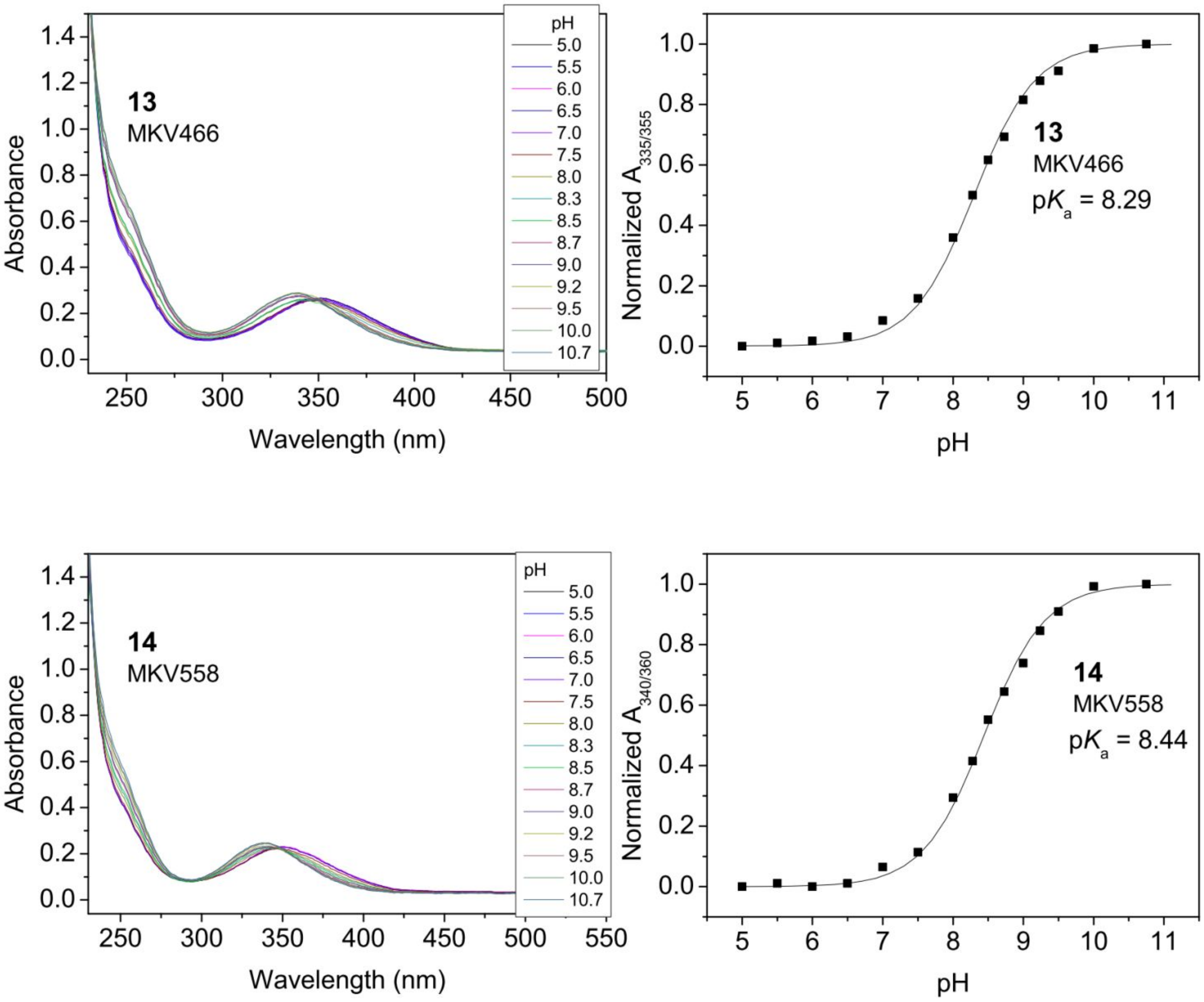

Figure S24. pK_a of sulfonamide amino group measurement. Absorption spectra of compounds at various pHs (left figures). The normalized ratio of absorbances (right figures, black squares) was plotted as a function of pH and fitted to the Henderson–Hasselbalch curve (black line) using the least square method.

Table S2. Observed dissociation constant $K_{d,obs}$ measured at pH 7.0 and pH 5.0 for CAIX isozyme and intrinsic constant $K_{d,int}$ calculated from the $K_{d,obs}$ values determined at pH 7.0 and pH 5.0.

Cmpd	Cmpd	$K_{d,obs}$ (CAIX), nM		$K_{d,int}$ (CAIX), nM	
		at pH 7.0	at pH 5.0	calculated from pH 7.0	calculated from pH 5.0
MKV84	**4c**	15	330	1.2	1.2
MKV85	**5c**	0.022	1.1	0.00039	0.00072
MKV255	**6**	0.012	0.67	0.000066	0.00013
MKV466	**13**	0.0046	0.17	0.000064	0.000084
MKV558	**14**	0.0045	0.25	0.000045	0.000089

Figure S25. ITC data of compound **13** binding to CAIX performed in 50 mM Tris, 100 mM NaCl buffer at different pHs: A – pH5.3, B – pH 7.0, C – pH 9.6 and D – pH 10.0.

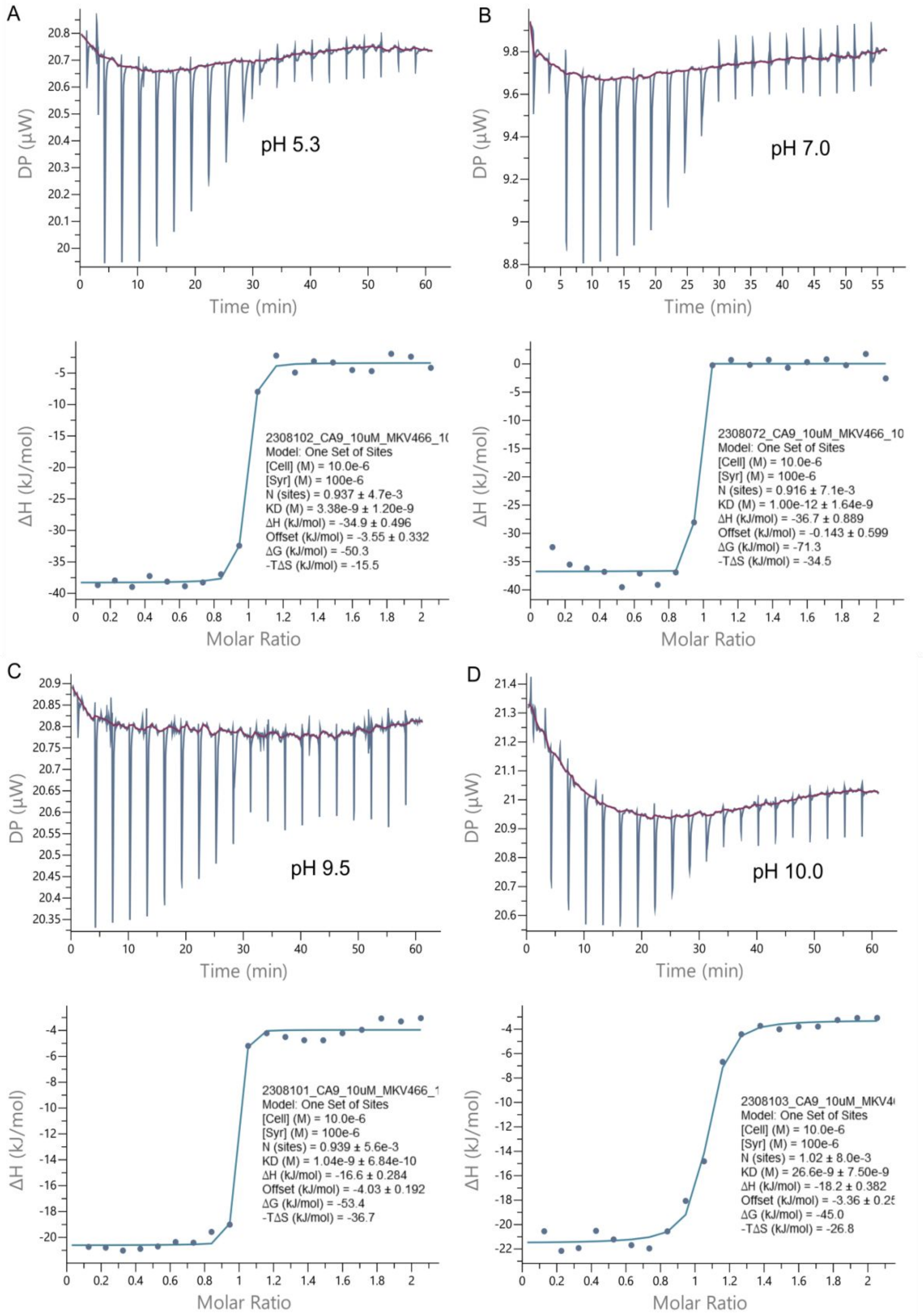

Table S3. Crystal structures of CA XII complexes with ligands obtained by soaking

Structure	CAXII – **10**	CAXII – **13**	CAXII – **9**	CAXII – **14**
Data collection statistics				
Space group	P 1 21 1	P 1 21 1	P 1 21 1	P 1 21 1
Cell constants: a, b, c, Å; α, β, γ, (º)	a=77.46 Å, b=74.10 Å, c=91.60 Å, α=γ=90º, β=108.94º	a=77.52 Å, b=74.08 Å, c=91.84 Å, α=γ=90º, β=108.84º	a=42.19 Å, b=41.32 Å, c=71.91 Å, α=γ=90º, β=104.20º	a=42.46 Å, b=41.40 Å, c=71.72 Å, α=γ=90º, β=103.99º
Unique reflections: overall (outer shell)	147538 (3726)	357013 (9280)	290166 (6944)	362792 (9234)
Resolution range, Å	72.53-1.21	86.44-1.12	86.73-1.21	73.96-1.12
Completeness: overall (outer shell), %	97.1 (50.1)	96.4 (50.8)	97.0 (46.9)	97.2 (50.3)
Multiplicity: overall (outer shell)	6.6 (1.9)	6.7 (1.9)	6.6 (1.9)	6.4 (2.0)
I/σ: overall (outer shell)	8.4 (0.7)	10.9 (1.8)	18.6 (1.4)	10.8 (0.9)
Rmerge: overall (outer shell)	0.085 (0.760)	0.087 (0.356)	0.035 (0.461)	0.081 (0.887)
Wilson B-factor, Å2	16.4	9.9	13.9	11.4
Refinement statistics				
Reflections: work / test	137654 / 9649	337942 / 19023	270936 / 19172	330692 / 30424
Rcryst / Rfree	0.140 / 0.185	0.136 / 0.178	0.137 / 0.182	0.149 / 0.179
RMSD: bond lengths, Å / bond angles, (°)	0.016 / 2.028	0.015 / 2.037	0.018 / 2.207	0.014 / 2.104
Ramachandran: favoured/ allowed/ outliers, %	98 / 2 / 0	98 / 2 / 0	97 / 3/ 0	95 / 5 / 0
average B-factors: all atoms/ main chain/ side chain/ Zn / ligand / solvent, $Å^2$	25.1 / 21.1 / 25.7 / 14.0 / 22.5 / 39.9	18.9 / 13.9 / 20.2 / 7.5 / 13.3 / 33.0	24.8 / 19.6 / 27.3 / 13.2 / 24.9 / 38.4	21.7 / 17.2 / 24.0 / 11.1 / 20.1 / 48.9
PDB ID	9F2N	9F2O	9F3G	9F30

Table S4. Crystal structures of CAIX and CAXII complexes with ligands obtained by co-crystallization.

Structure	CAIX – **26**	CAXII – **10**	CAXII –**13**	CAXII – **14**
space group	H3	P1	P1	P1
cell dimensions				
a (Å)	152.59	46.57	46.62	46.59
b (Å)	152.59	76.70	67.47	67.12
c (Å)	172.57	78.87	80.86	80.46
α (°)	90.00	62.61	81.17	81.54
β (°)	90.00	79.07	84.19	84.30
γ (°)	120.00	72.89	86.37	86.47
resolution (Å)	47.98-2.00	44.41-1.45	41.75-1.25	47.64-1.19
highest resolution shell (Å)	2.00-2.03	1.45-1.47	1.25-1.27	1.19-1.21
no. of reflections	101262	158363	255967	292651
no. of reflections in test set	395	584	924	1085
completeness (%)	100.0 (100.0[a])	96.6 (94.8[a])	95.6 (92.9[a])	95.2 (92.4[a])
R_{merge}	0.13 (1.48[a])	0.06 (0.31[a])	0.08 (0.31[a])	0.03 (0.22[a])
$\langle I/\sigma I \rangle$	15.5 (2.1[a])	10.2 (3.9[a])	7.8 (3.6[a])	18.3 (4.6[a])
average multiplicity	10.5 (10.8[a])	3.6 (3.5[a])	3.6 (3.6[a])	3.6 (3.5[a])
R-factor	0.17 (0.27[a])	0.18 (0.20[a])	0.18 (0.22[a])	0.14 (0.18[a])
R_{free}	0.21 (0.29[a])	0.21 (0.24[a])	0.22 (0.23[a])	0.17 (0.21[a])
average B factor (Å^2)	36.4	12.3	11.5	11.9
average B factor for inhibitor (Å^2)	64.4	23.1	10.1	11.3
$\langle B \rangle$ from Wilson plot (Å^2)	32.9	8.3	7.1	7.6
no. of protein atoms	7360	8359	8336	8348
no. of inhibitor atoms	128	136	120	124
no. of solvent molecules	444	773	1081	1114
rms deviations from ideal values				
bond lengths (Å)	0.01	0.01	0.01	0.01
bond angles (°)	1.81	1.99	1.97	2.05
outliers in Ramachandran plot (%)	0.32	0.1	0.48	0.39
PDB code	9R30	9R0L	9R31	9R0U

[a] – outer shell.

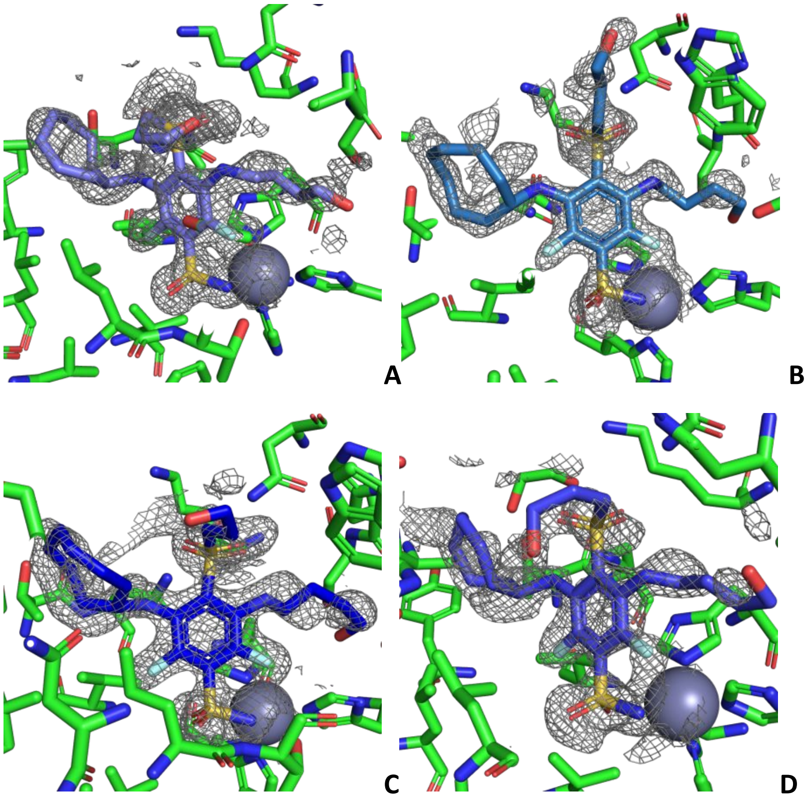

Figure S26. A-D. Electron density difference (Fo-Fc) maps for CAXII in complex with compound **9** (PDB ID: 9F3G) are contoured at 2σ. The overall orientation of the ligand in all four subunits is similar, but variations in position and conformation between chains are more pronounced compared to other compounds. The electron density in all chains is weak, indicating that alternate conformations are highly likely. A single conformation has been modeled, but its precise positioning could not be determined with confidence. The cyclooctyl rings are only partially visible in the electron density maps, yet they appear to be positioned similarly across the subunits. Attempts to place the rings in the hydrophilic region of the binding pocket were unsuccessful, suggesting that this conformation is unfavorable. The *meta*-hydroxybutyl substituent is relatively large and does not easily fit into the hydrophilic region of the active site. The electron density suggests that it adopts multiple conformations, preventing the exact position of this substituent from being modeled with high confidence.

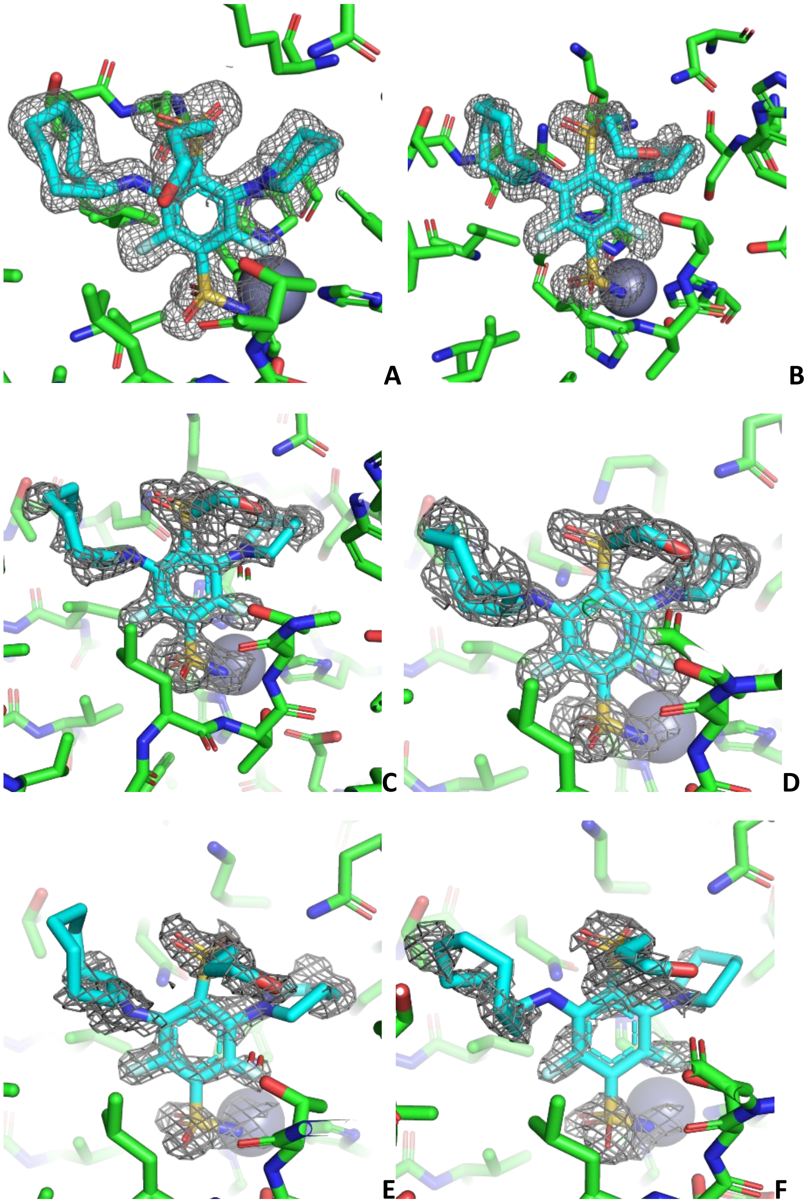

Figure S27. Compound **10** bound in the active site of CAXII. Electron density difference (Fo-Fc) maps are contoured at 2σ. (A) and (B) show two subunits in the asymmetric unit of PDB ID: 9F2N, while (C-F) display the four subunits of PDB ID: 9R0L. The electron density for the ligand in PDB ID: 9F2N is well-defined, allowing for clear tracing of all atoms within the binding site. In contrast, the electron density in PDB ID: 9R0L is much weaker, making it difficult to confidently trace certain ligand atoms. Despite the differences in density quality, the overall conformations of the ligand in all subunits are highly similar.

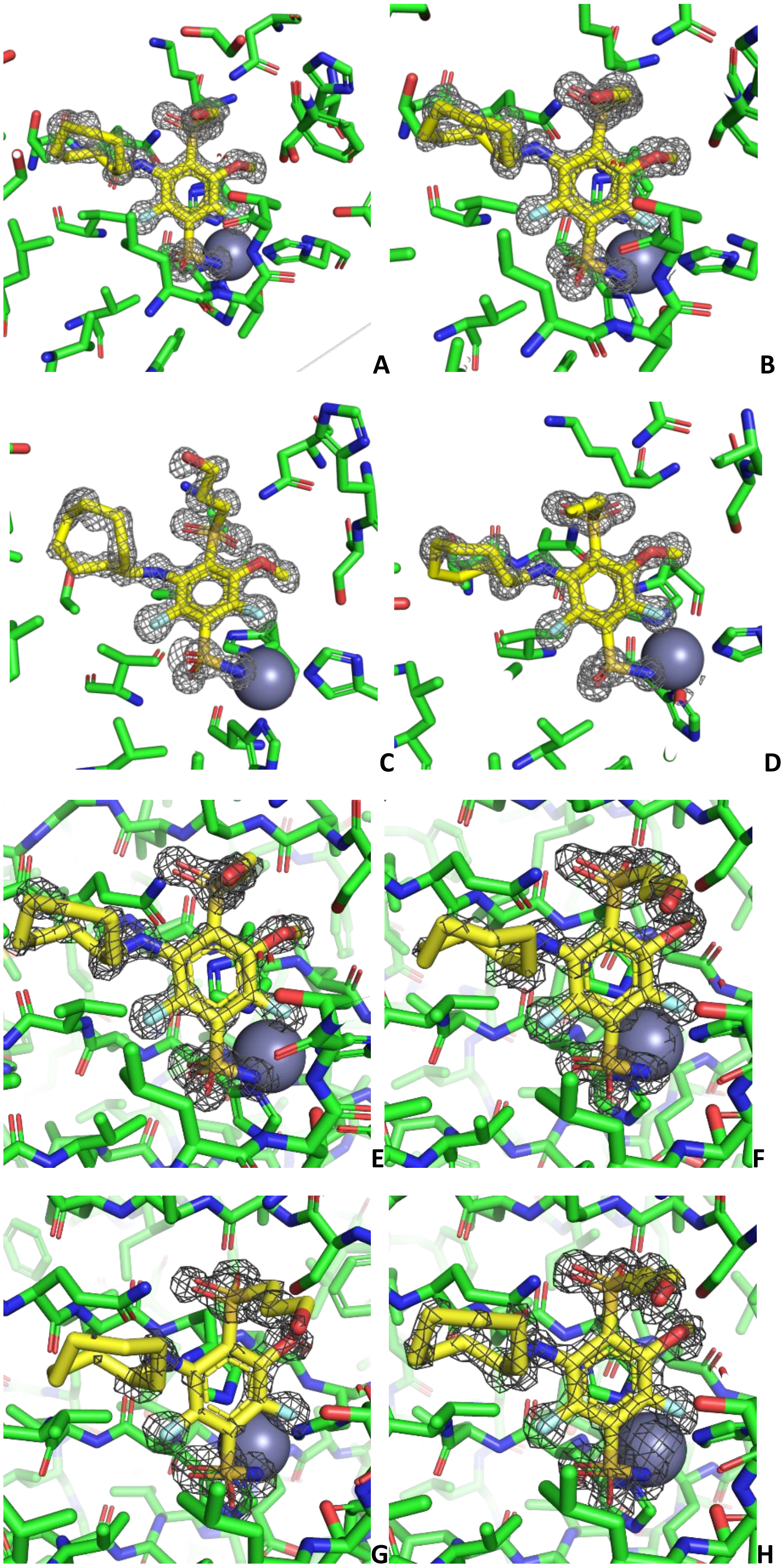

Figure S28. Electron density difference (Fo-Fc) maps are contoured for CAXII – **13** at 3σ. (A-D) – show different subunits of PDB ID: 9F2O, while (E-H) correspond to PDB ID: 9R31. Both structures contain four protein chains in the asymmetric unit, allowing for the comparison of eight independent ligand-binding cases. The ligands bound in all protein chains adopt a highly similar

conformation, with well-defined electron density across most subunits. Uncertainties are observed in the conformations of the cyclo-octyl ring and the *para*-tail, suggesting minimal variability in ligand positioning within the binding site.

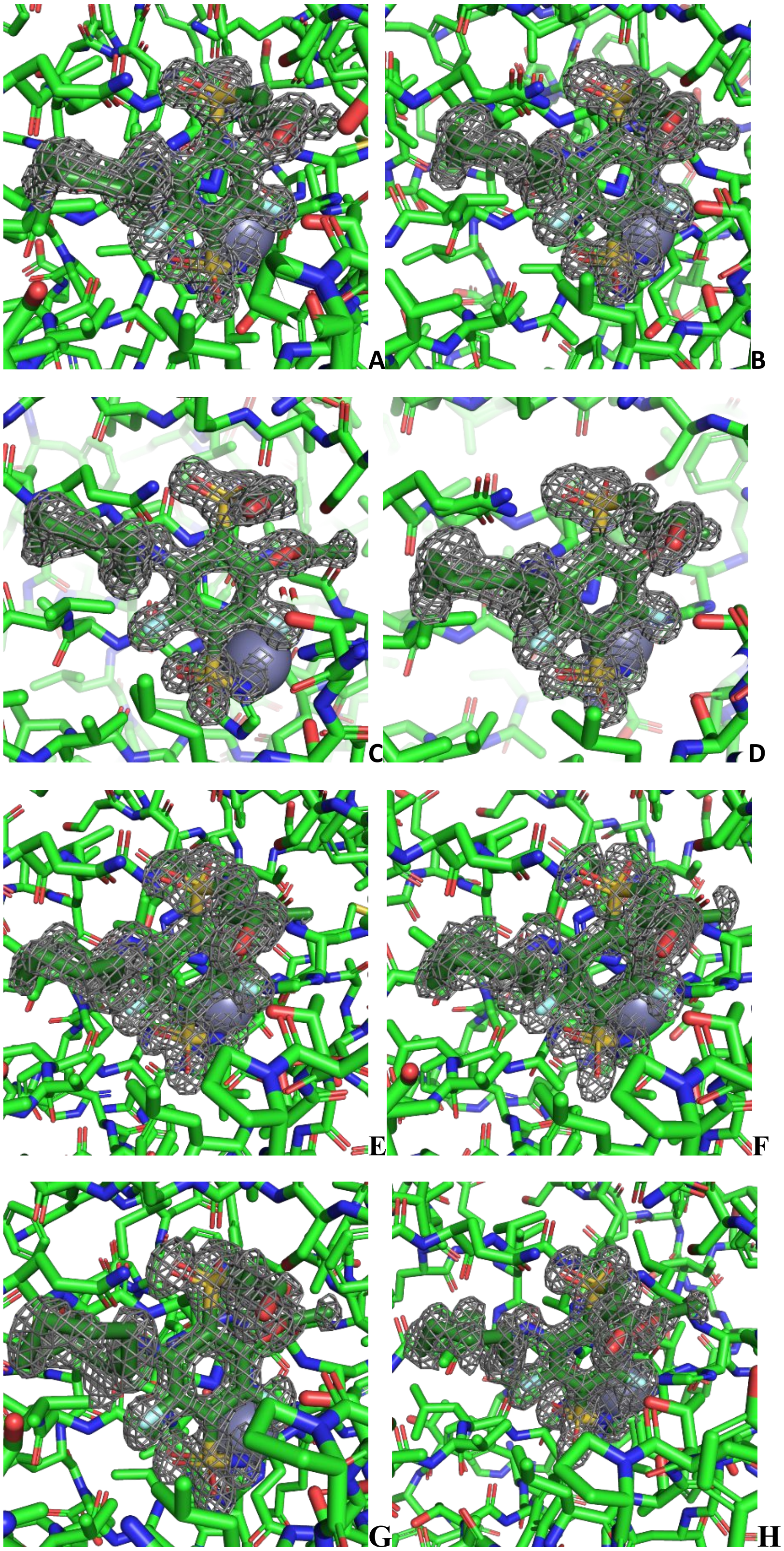

Figure S29. Electron density difference (Fo-Fc) maps for CAXII – **14** are contoured at 2σ. (A-D) – show different subunits of PDB ID: 9F30, while (E-H) corresponds to PDB ID: 9R0U. Both structures contain four protein chains in the asymmetric unit, enabling the comparison of eight independent ligand-binding cases. The electron density of the compound in the active site of

CAXII is well-defined in PDB ID: 9F30, whereas it is weaker in PDB ID: 9R0U. Despite this, the overall conformation of all ligand molecules remains highly similar, with only small differences observed in the cyclo-octyl ring and *para*-tail conformations.

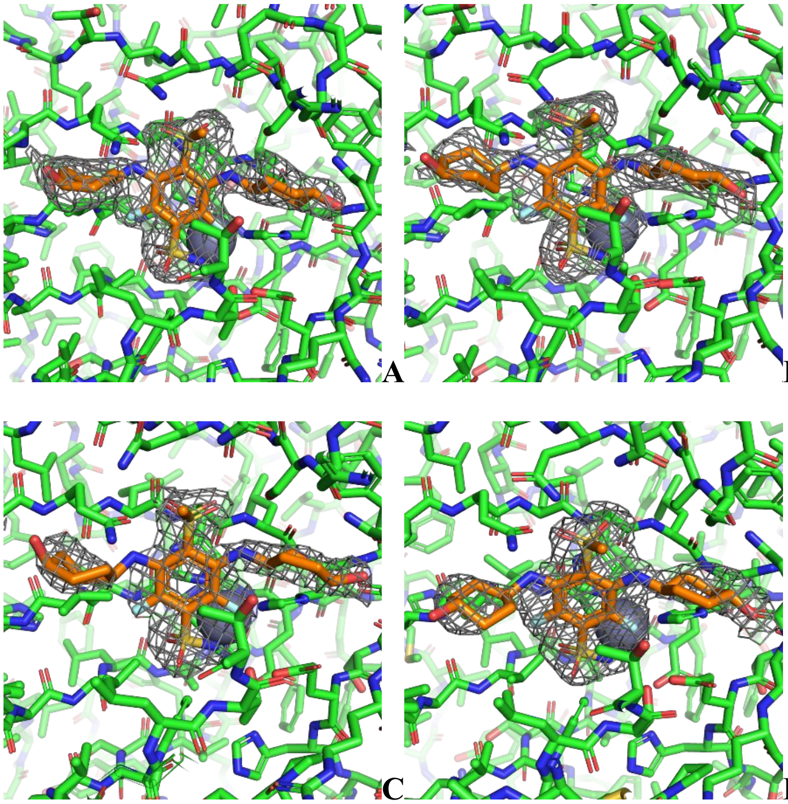

A B C D

Figure S30. A-D. Electron density difference (Fo-Fc) maps for CAIX – **26** (PDB ID: 9R30) are contoured at 2σ. Due to lower resolution and weaker electron density, a single alternative conformation has been modeled, though the presence of additional alternative conformations remains likely.

Compound ^{1}H NMR, ^{13}C NMR, ^{19}F NMR and HRMS spectra

Figure S31. Compound **4c** ^{1}H NMR:

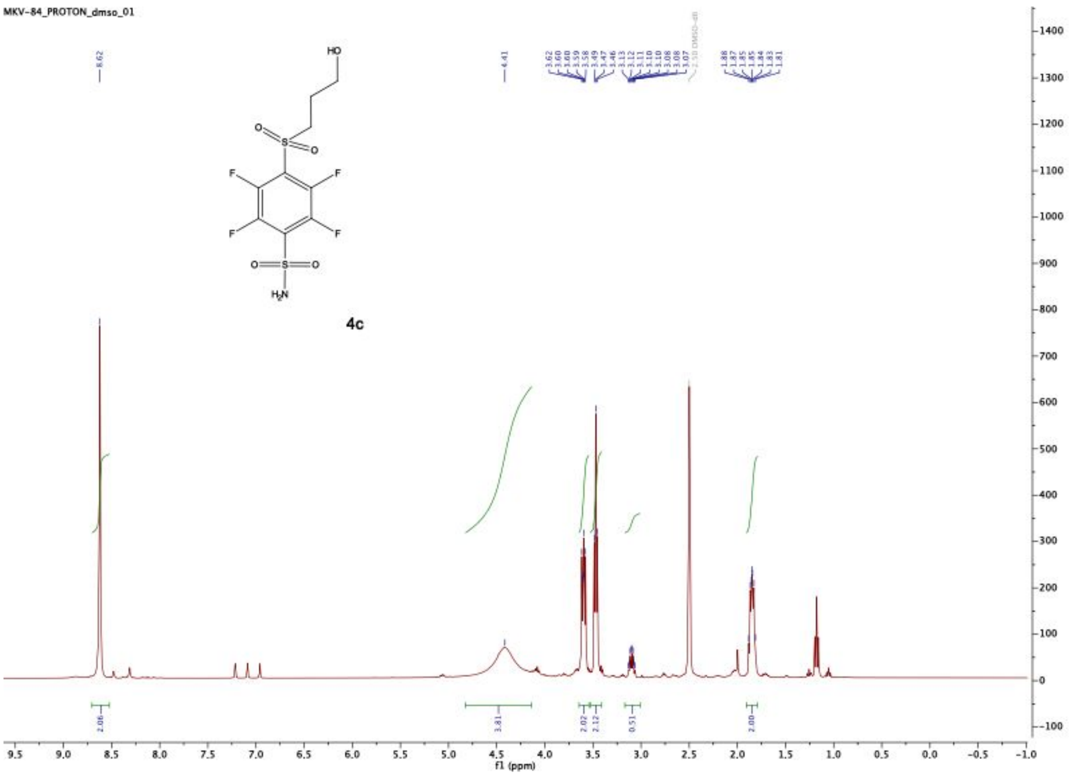

Figure S32. Compound **4c** ^{13}C NMR:

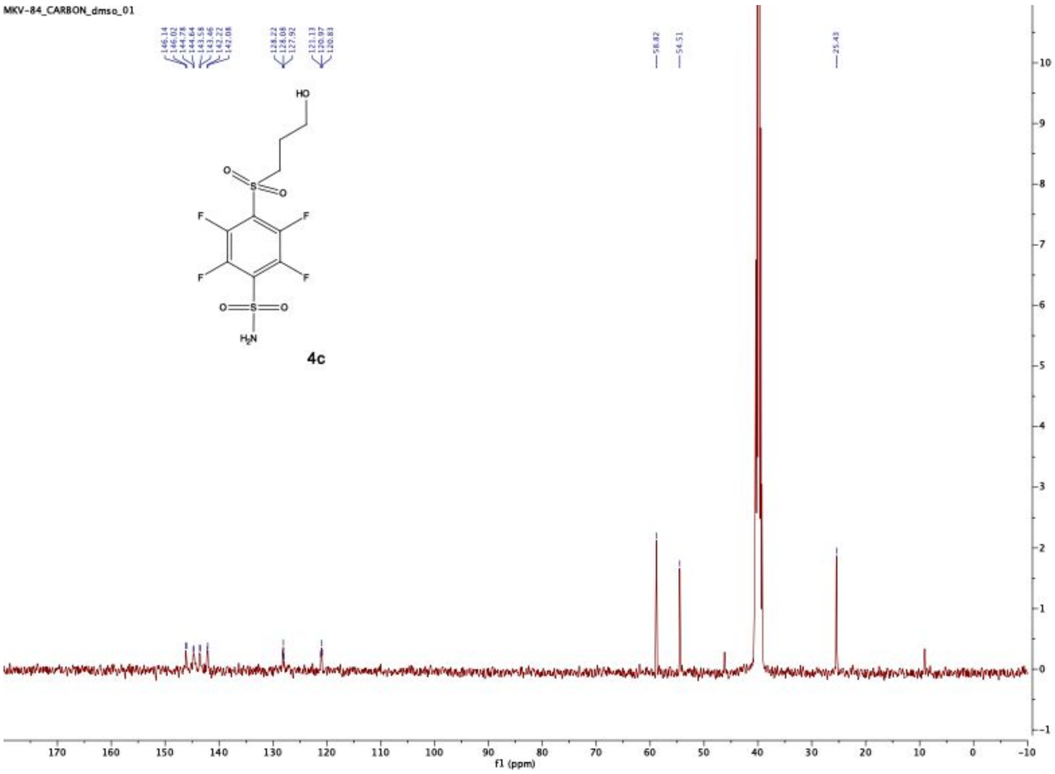

Figure S33. Compound **4c** ^{19}F NMR:

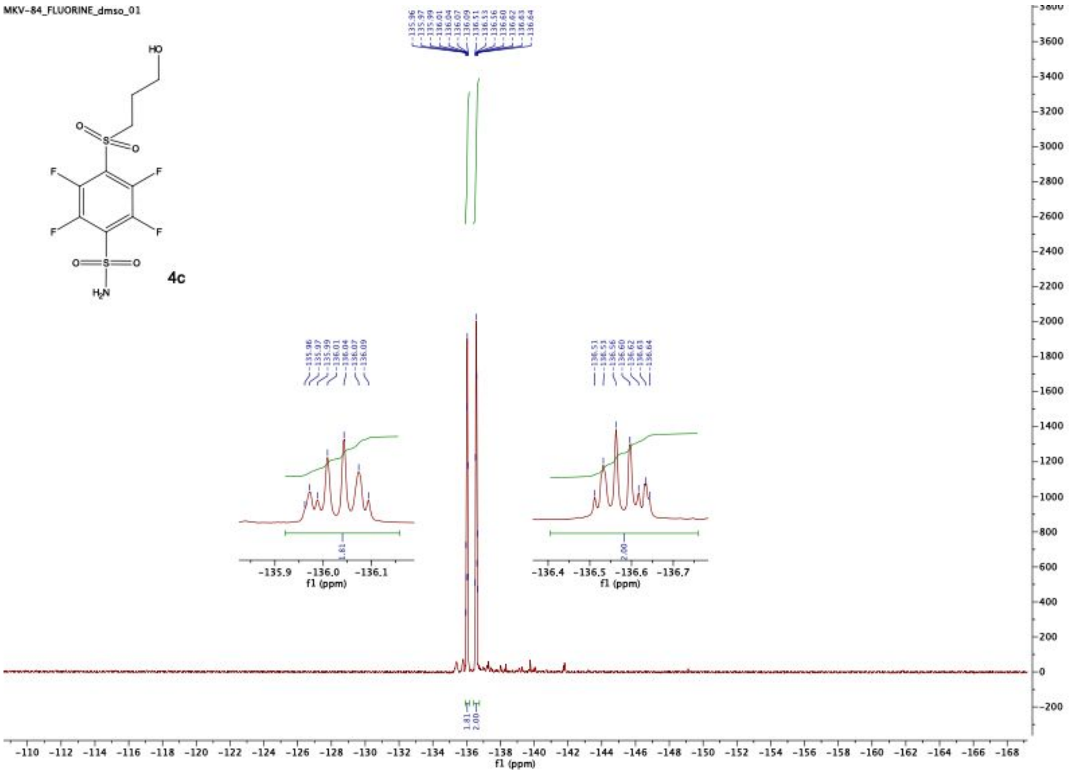

Figure S34. Compound **4c** HRMS:

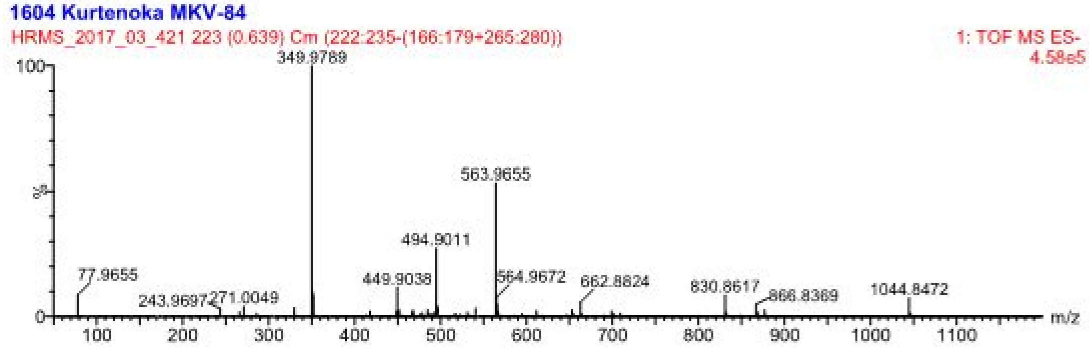

Figure S35. Compound **5c** ^{1}H NMR:

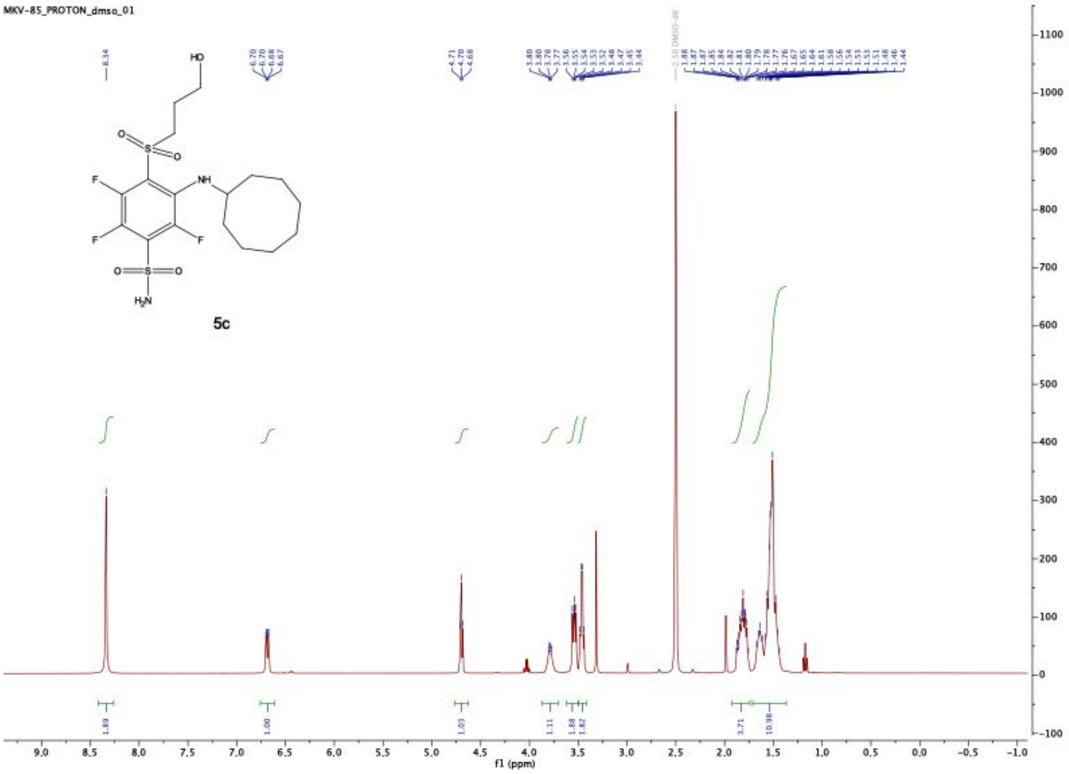

Figure S36. Compound **5c** ^{13}C NMR:

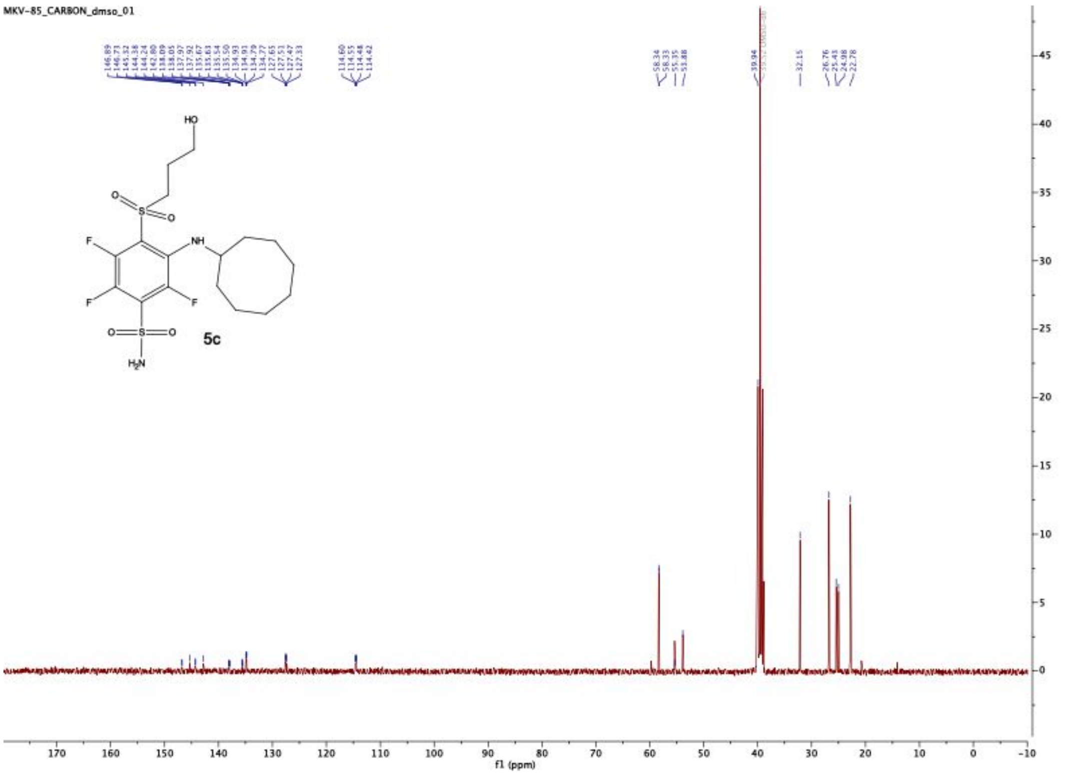

Figure S37. Compound **5c** ^{19}F NMR:

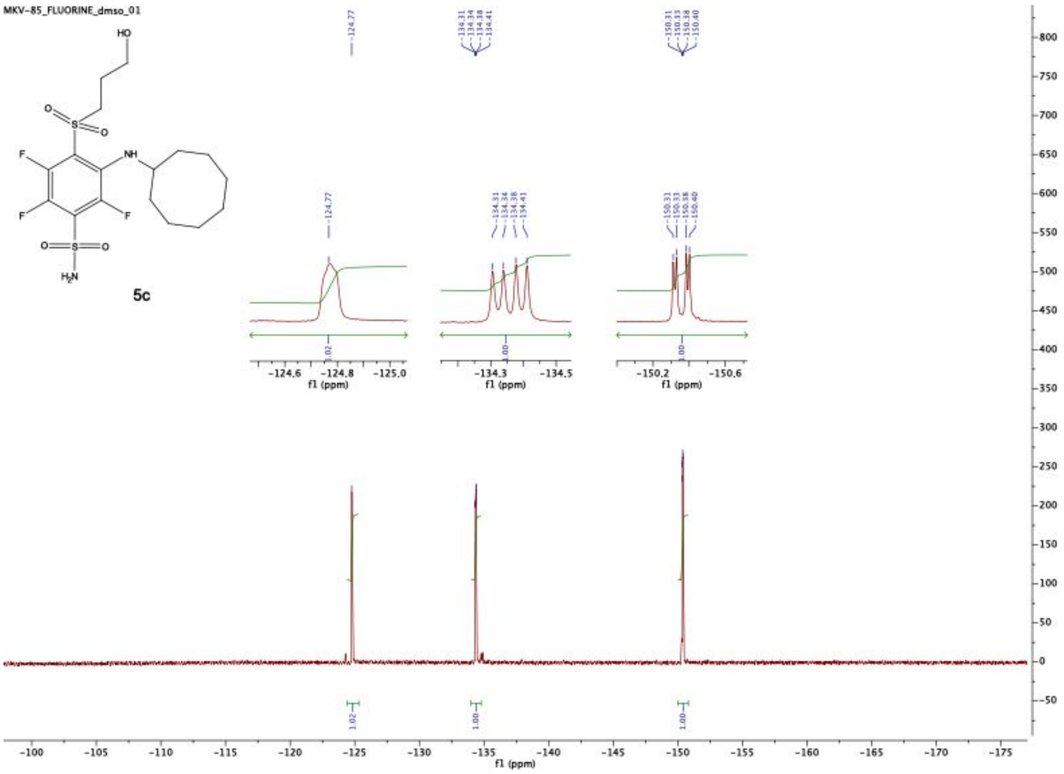

Figure S38. Compound **5c** HRMS:

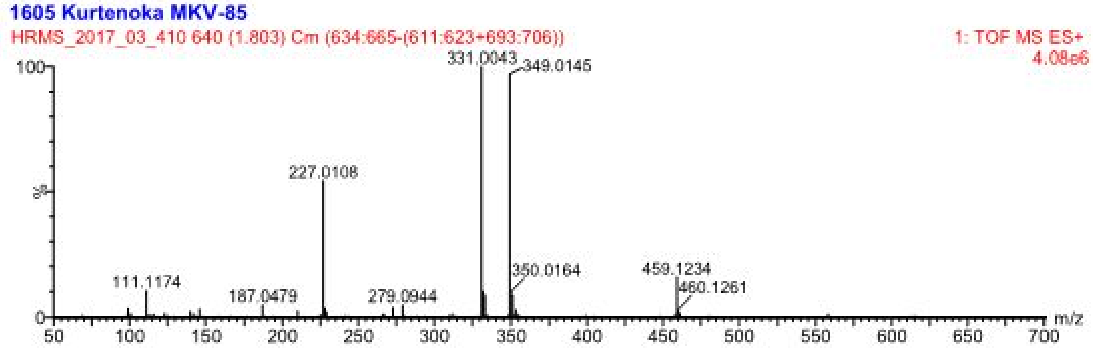

Figure S39. Compound **6** ^{1}H NMR:

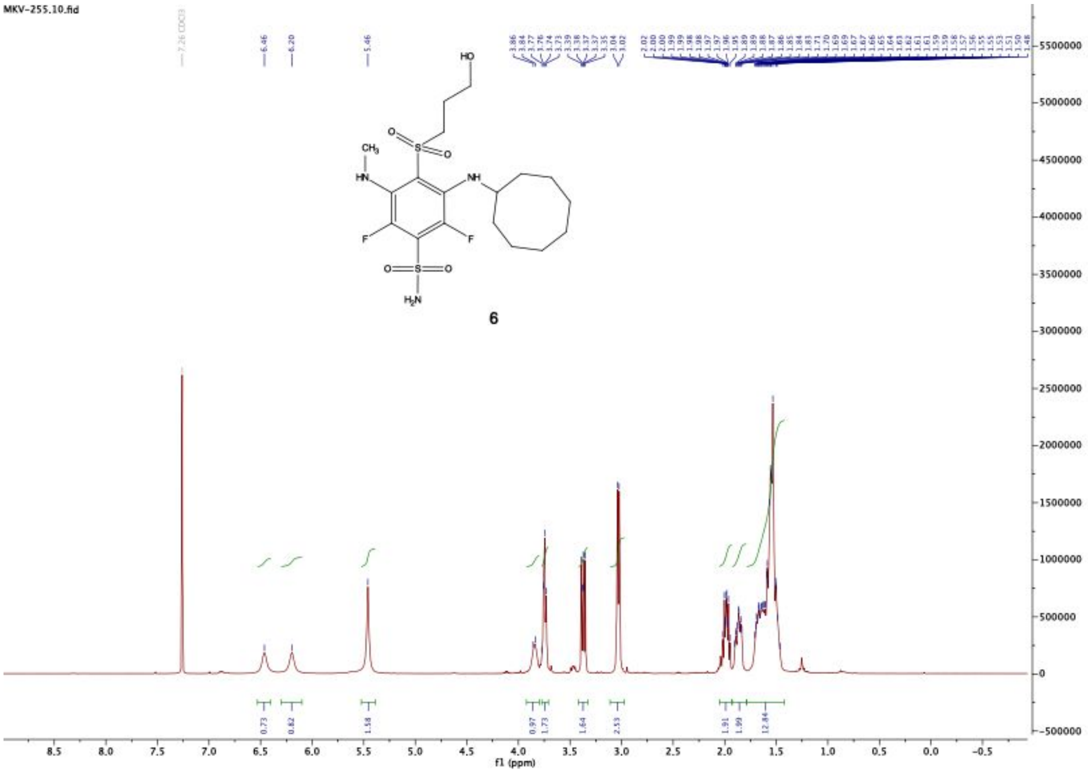

Figure S40. Compound **6** ^{13}C NMR:

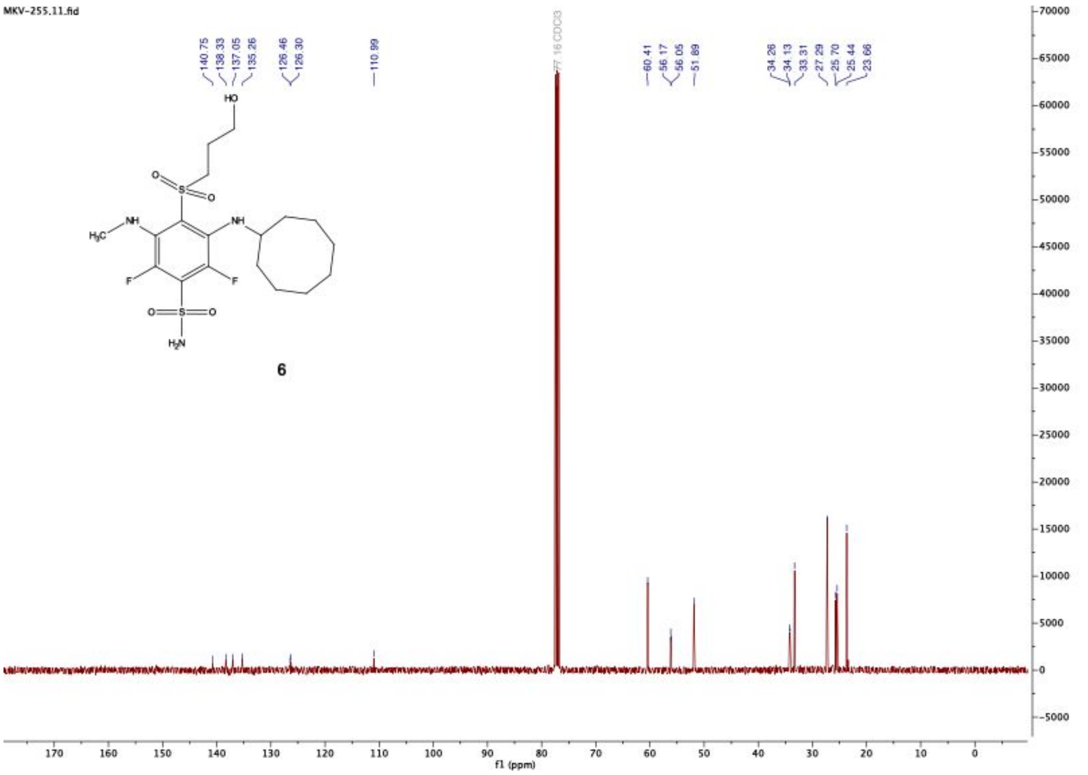

Figure S41. Compound **6** ^{19}F NMR:

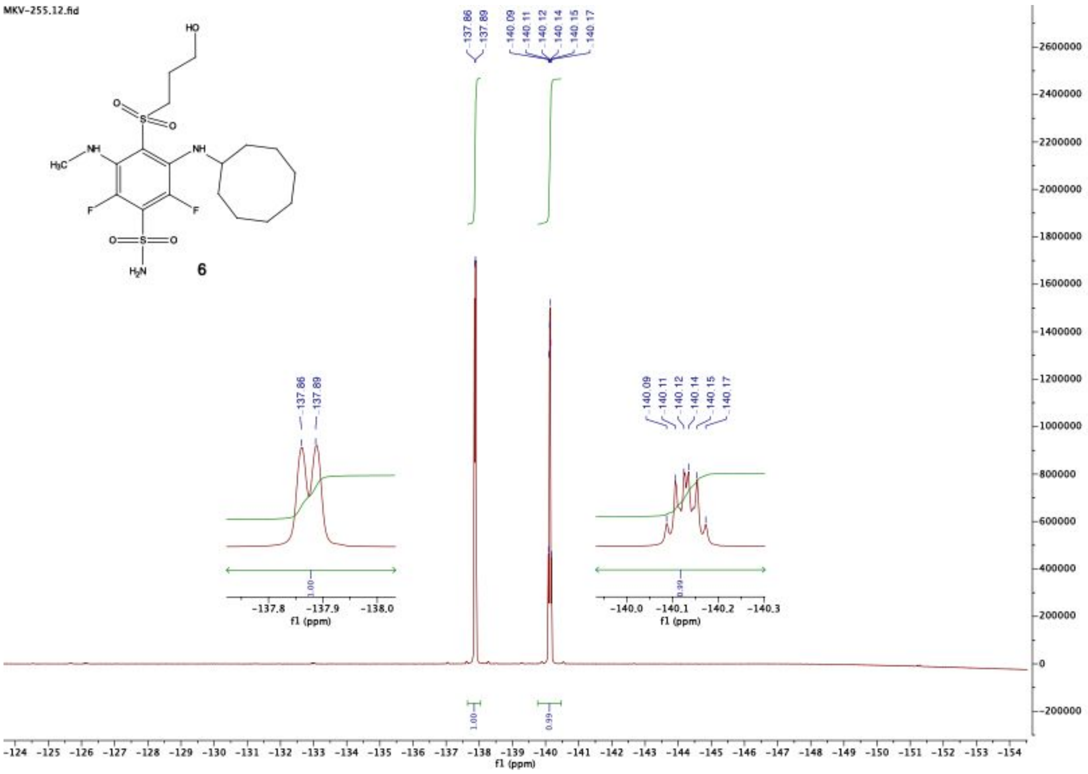

Figure S42. Compound **6** HRMS:

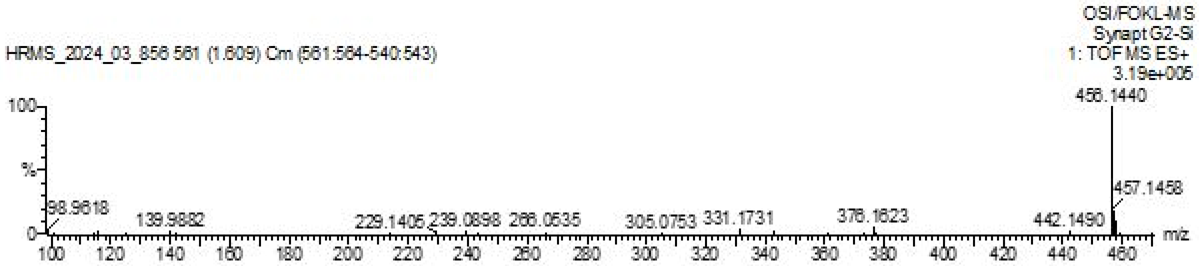

Figure S43. Compound **7** ^{1}H NMR:

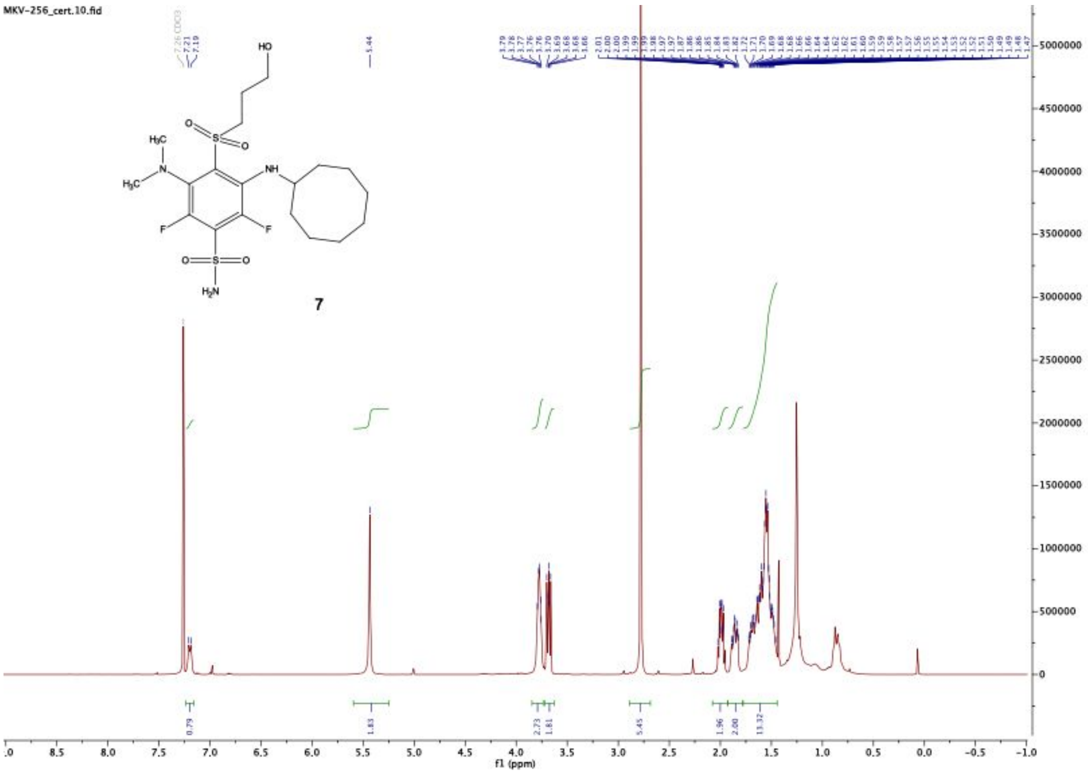

Figure S44. Compound **7** ^{13}C NMR:

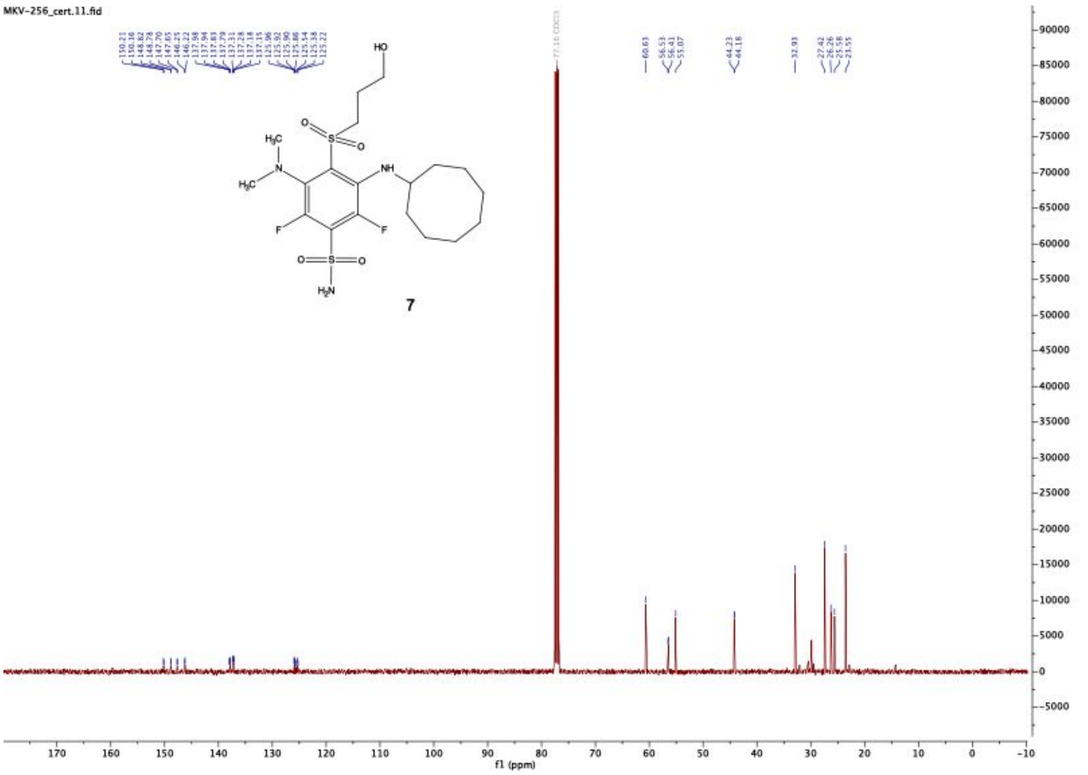

Figure S45. Compound **7** ^{19}F NMR:

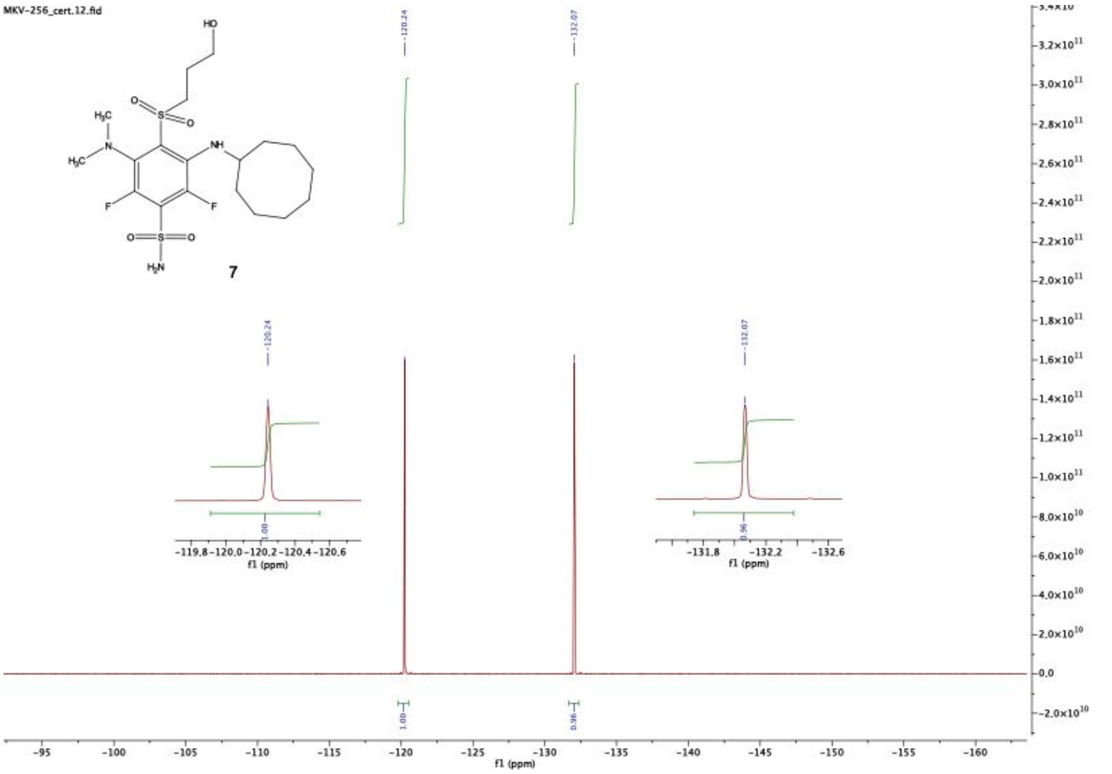

Figure S46. Compound **7** HRMS:

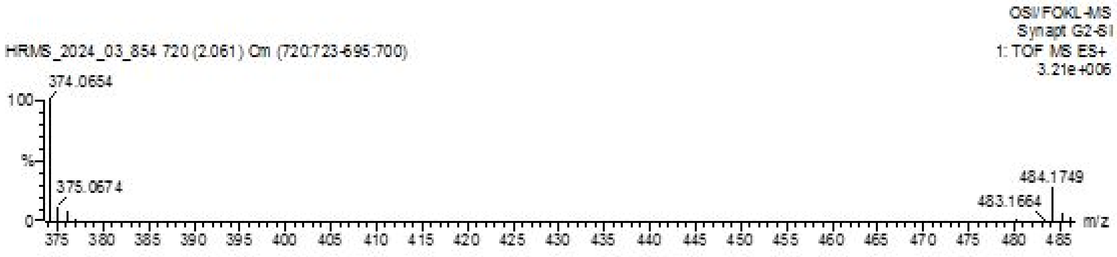

Figure S47. Compound **8** ^{1}H NMR:

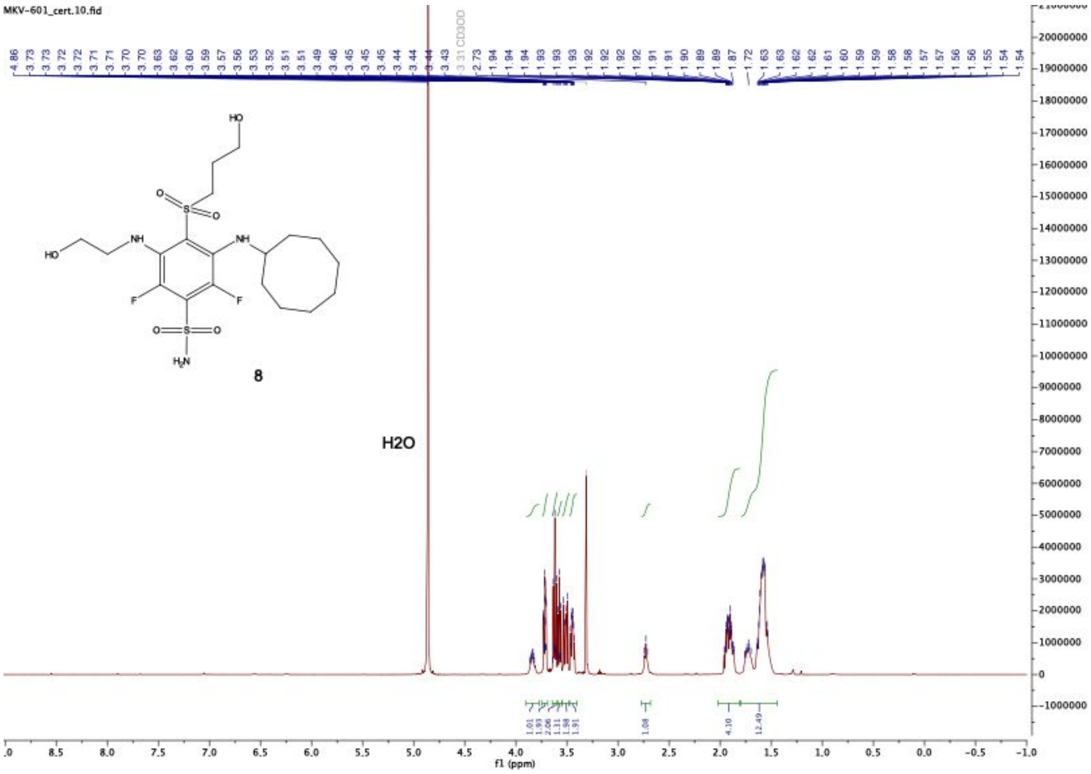

Figure S48. Compound **8** ^{13}C NMR:

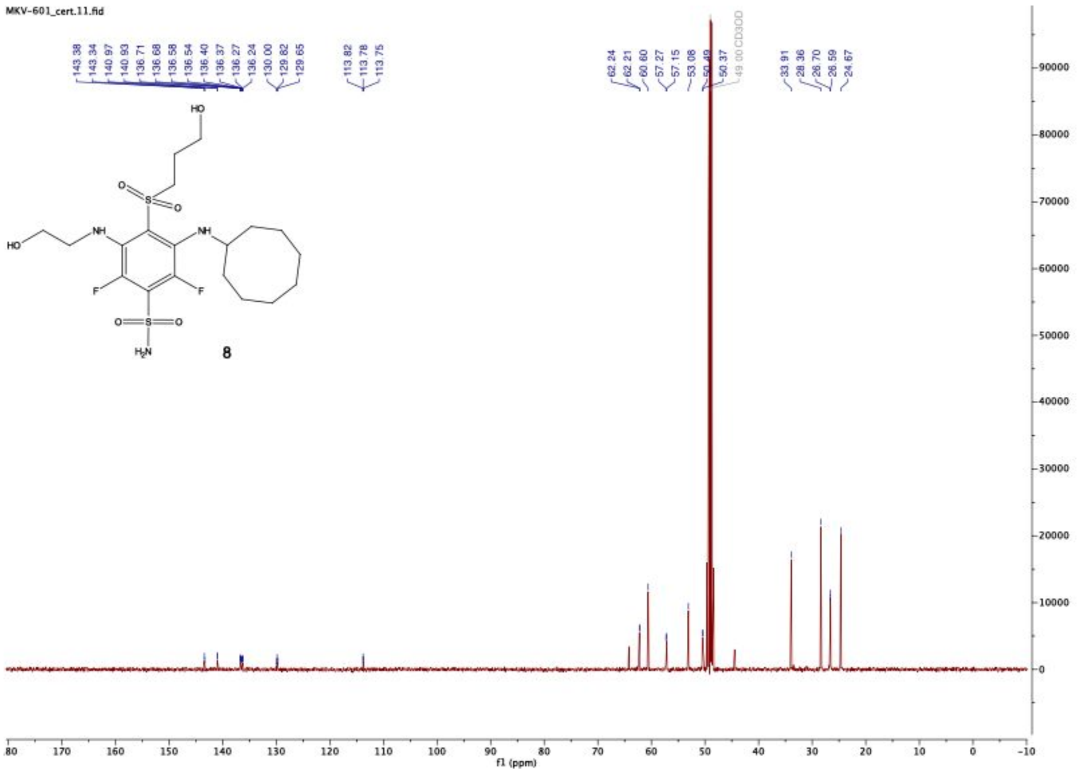

Figure S49. Compound **8** ^{19}F NMR:

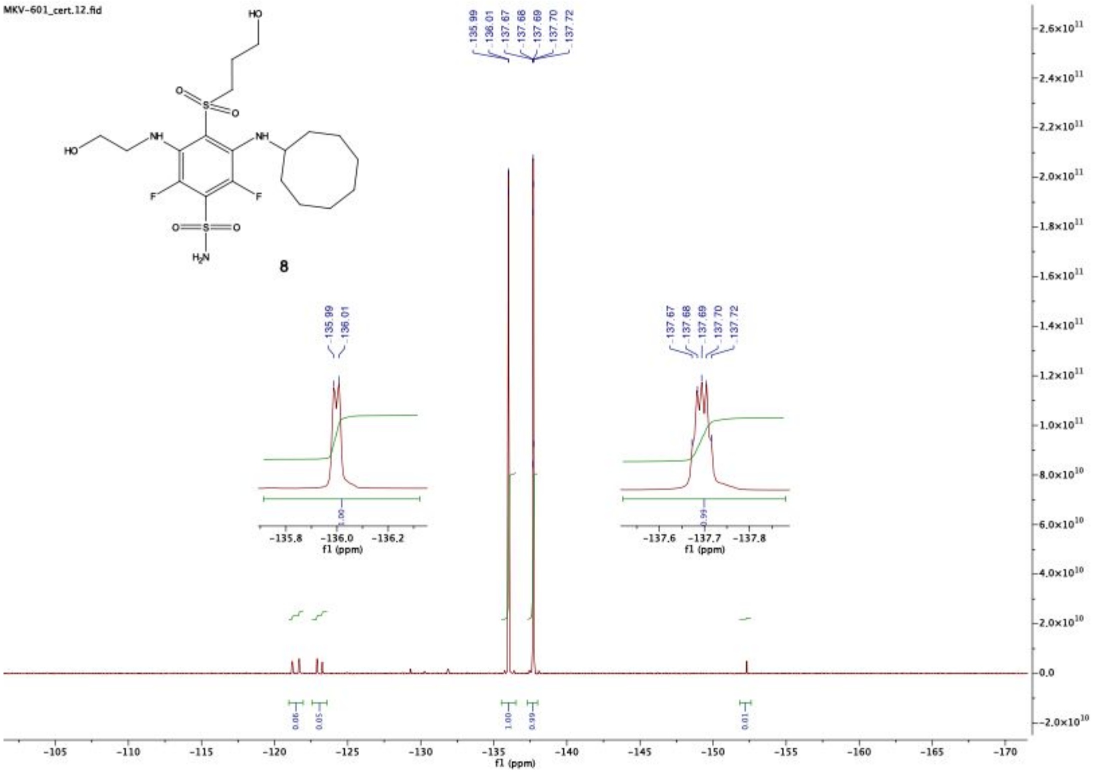

Figure S50. Compound **8** HRMS:

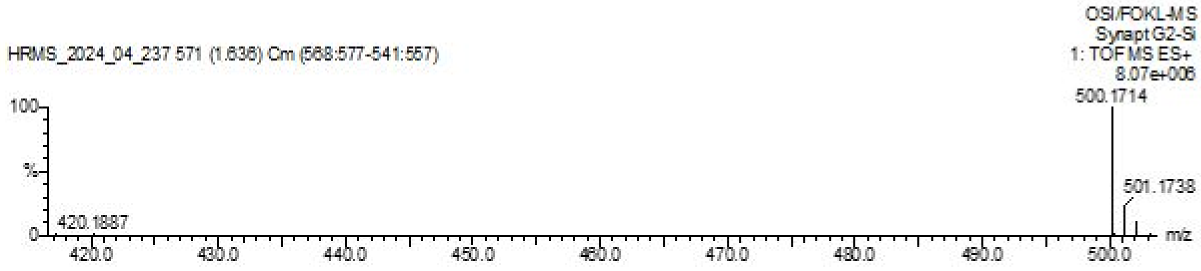

Figure S51. Compound **9** ^{1}H NMR:

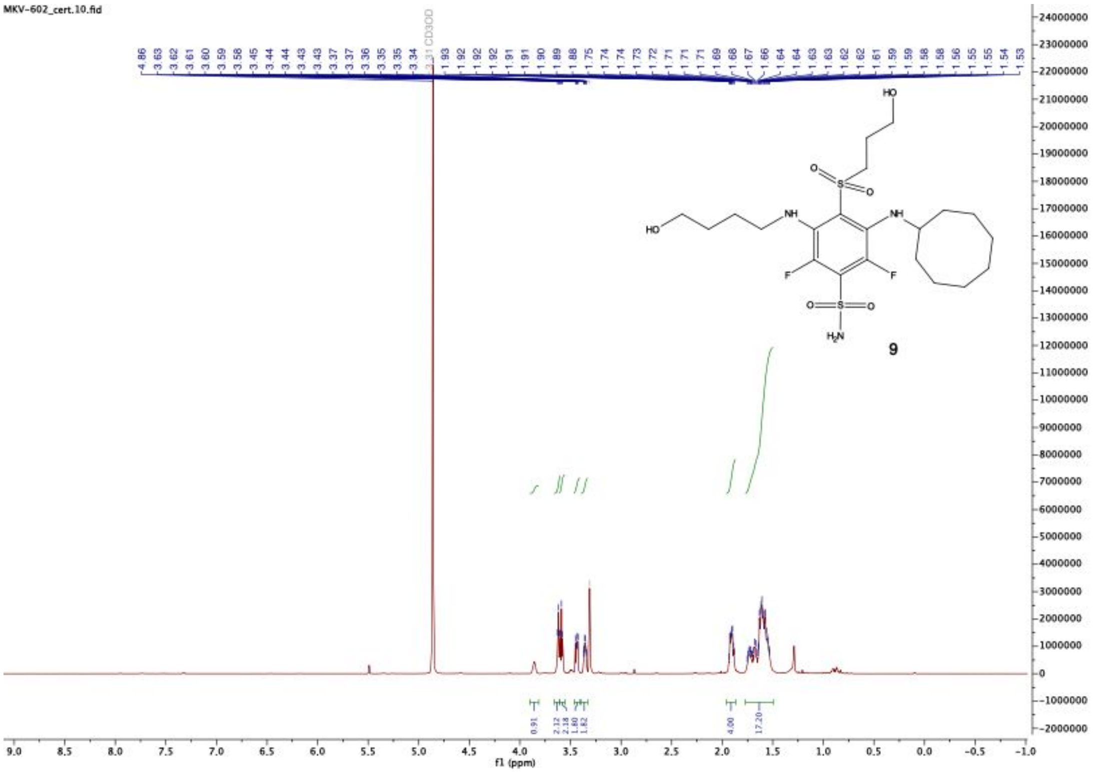

Figure S52. Compound **9** ^{13}C NMR:

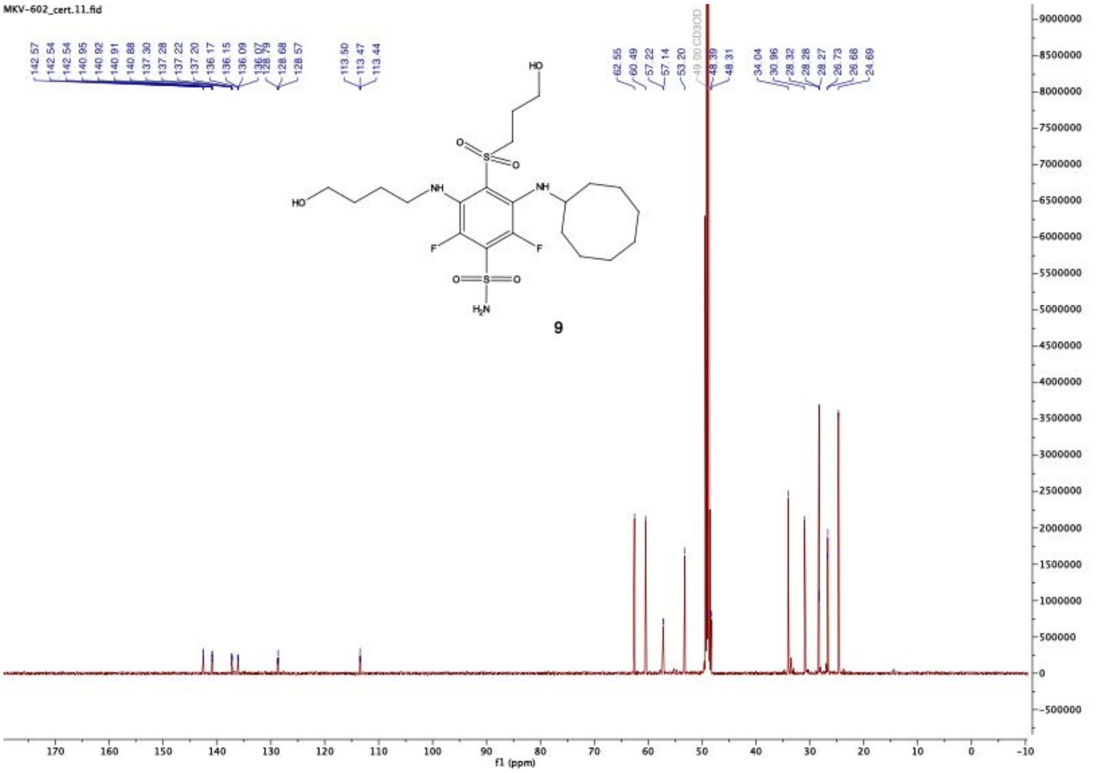

Figure S53. Compound **9** ^{19}F NMR:

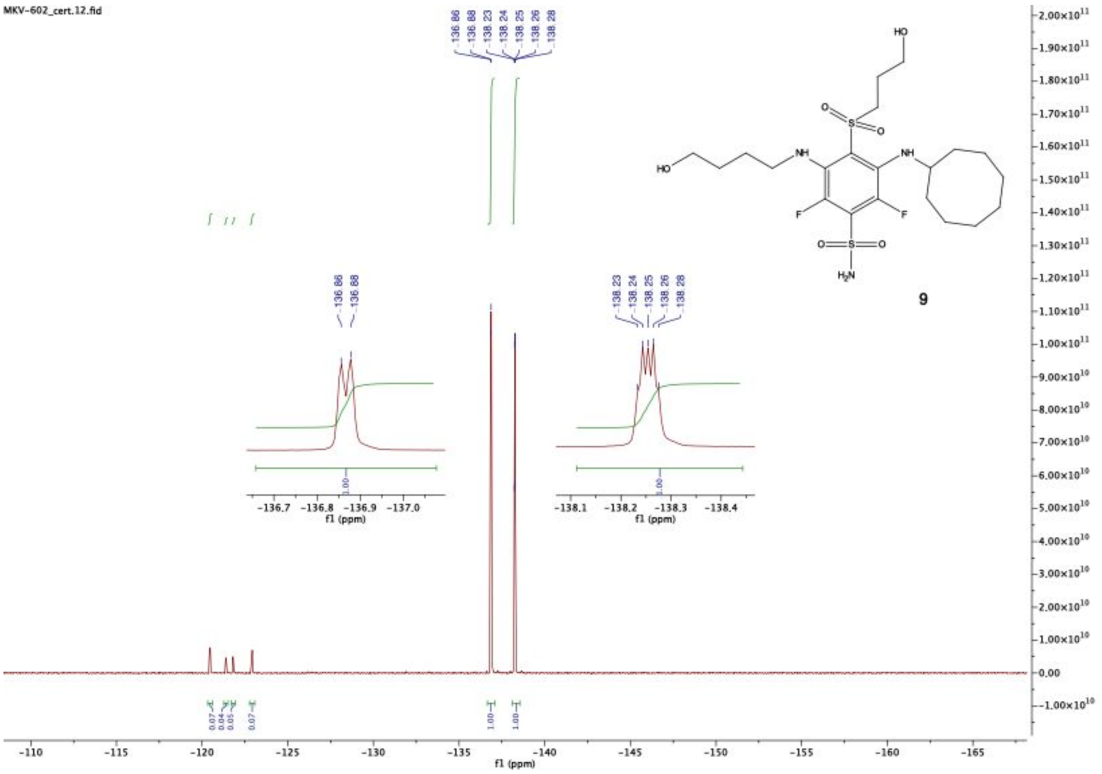

Figure S54. Compound **9** HRMS:

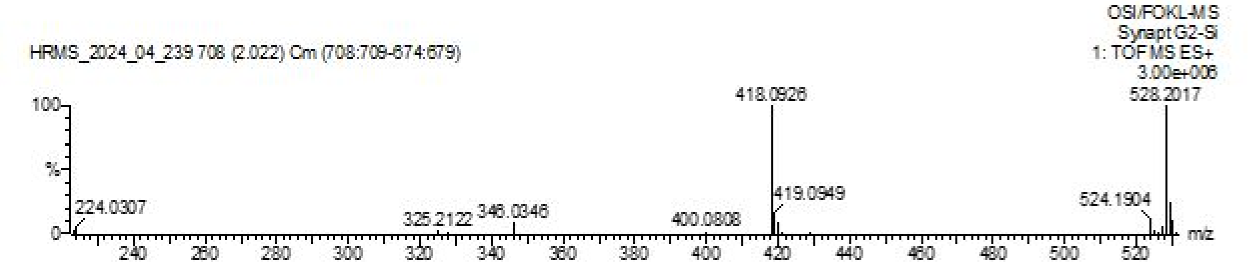

Figure S55. Compound **10** ^{1}H NMR:

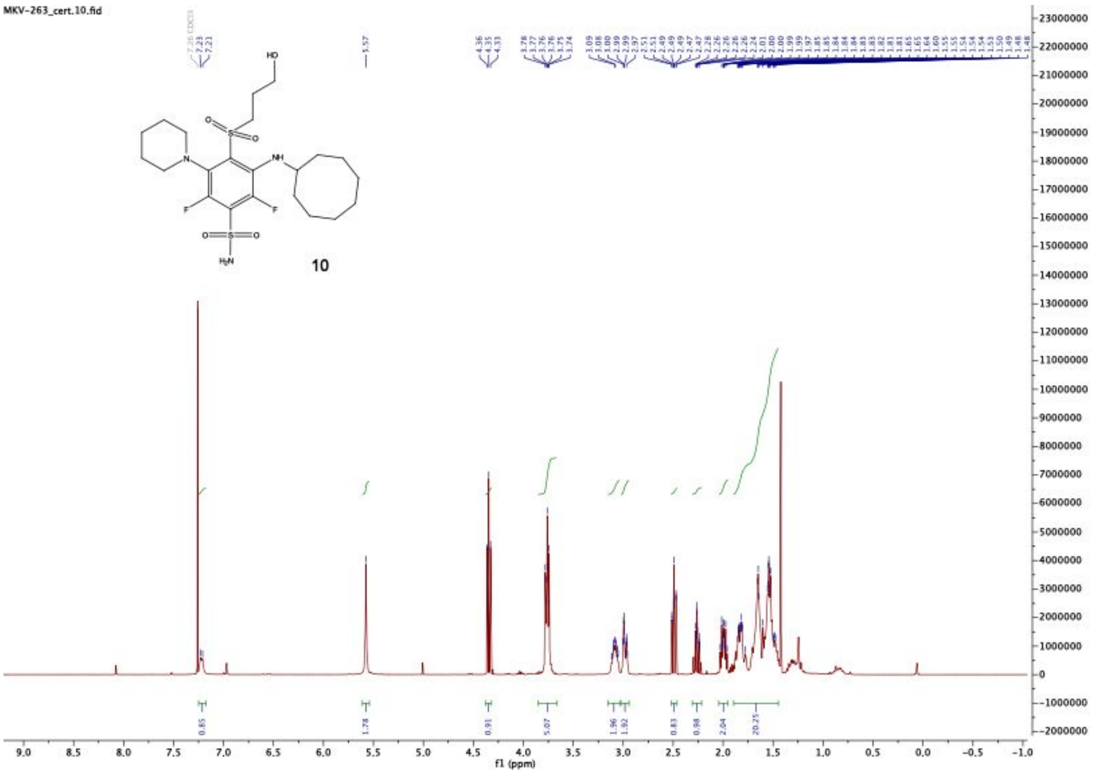

Figure S56. Compound **10** ^{13}C NMR:

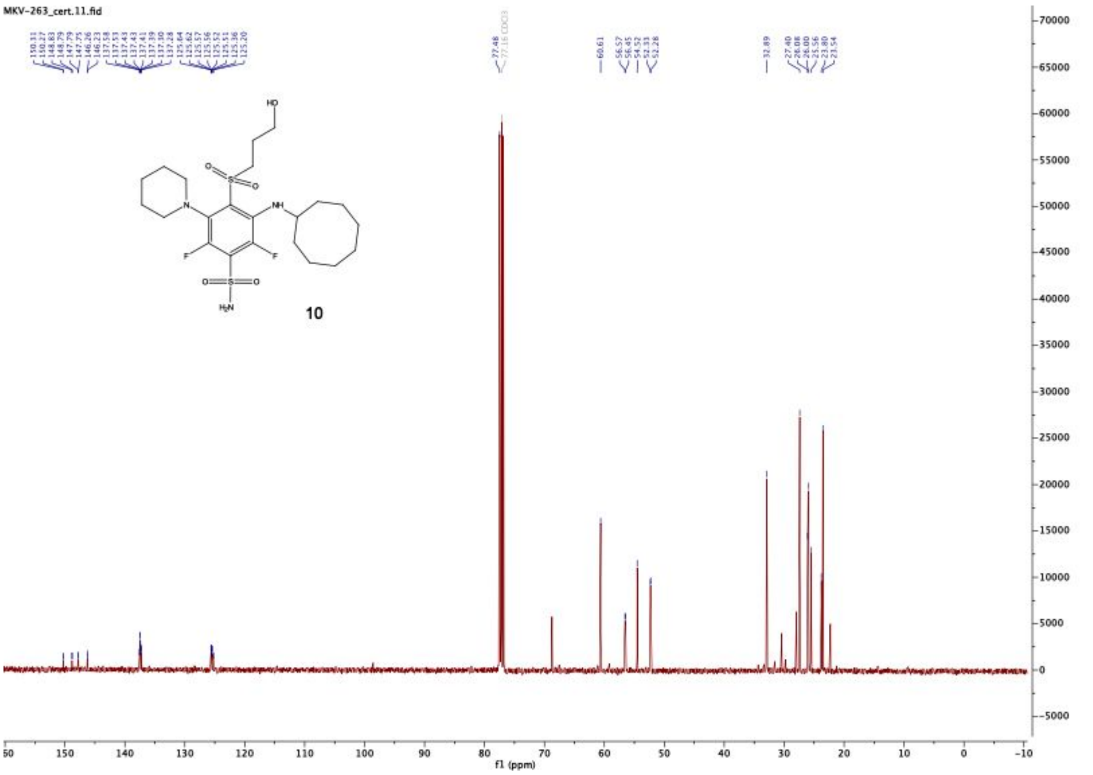

Figure S57. Compound **10** ^{19}F NMR:

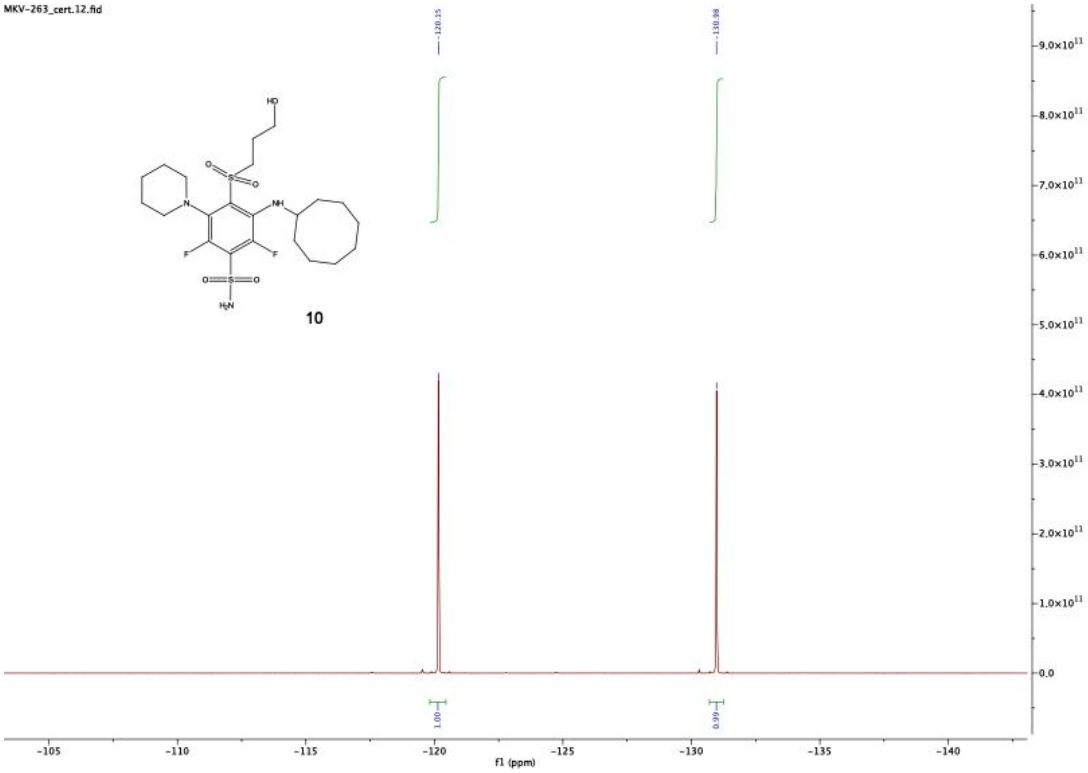

Figure S58. Compound **10** HRMS:

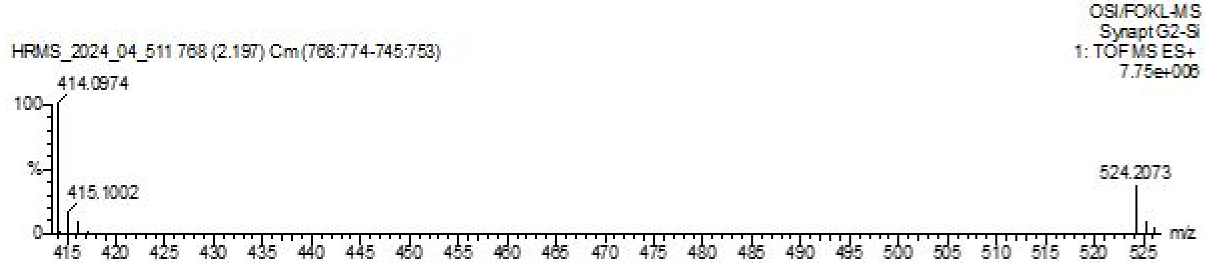

Figure S59. Compound **11** ^{1}H NMR:

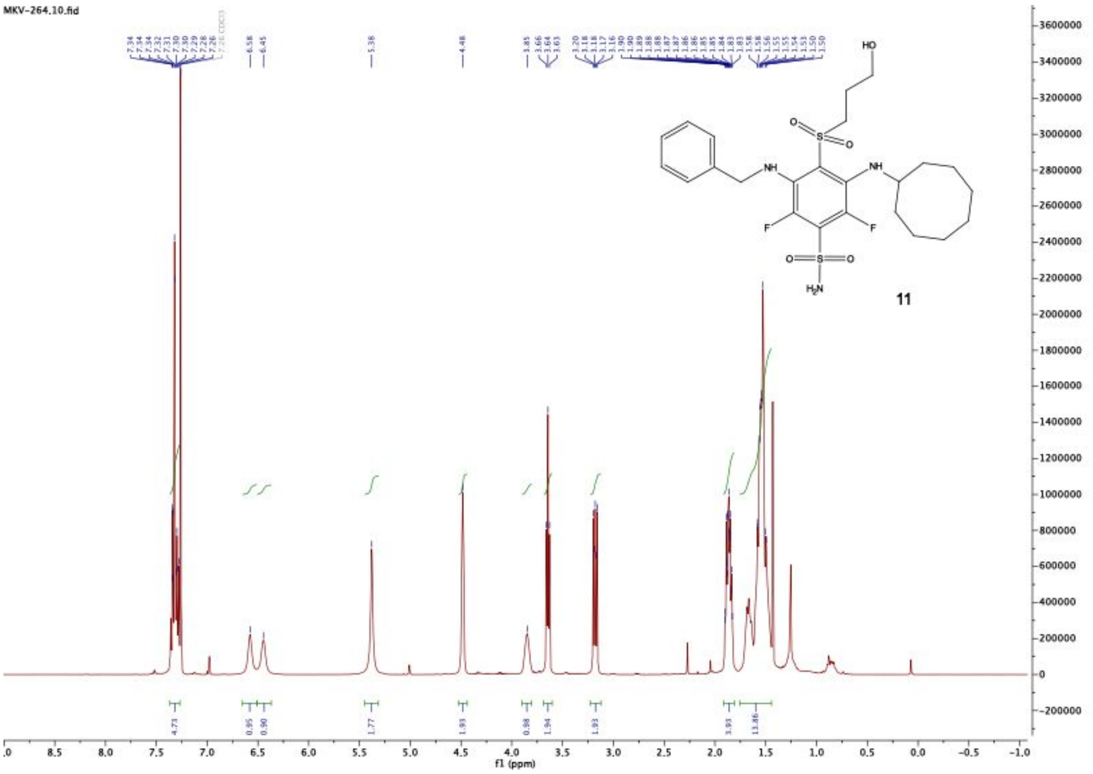

Figure S60. Compound **11** ^{13}C NMR:

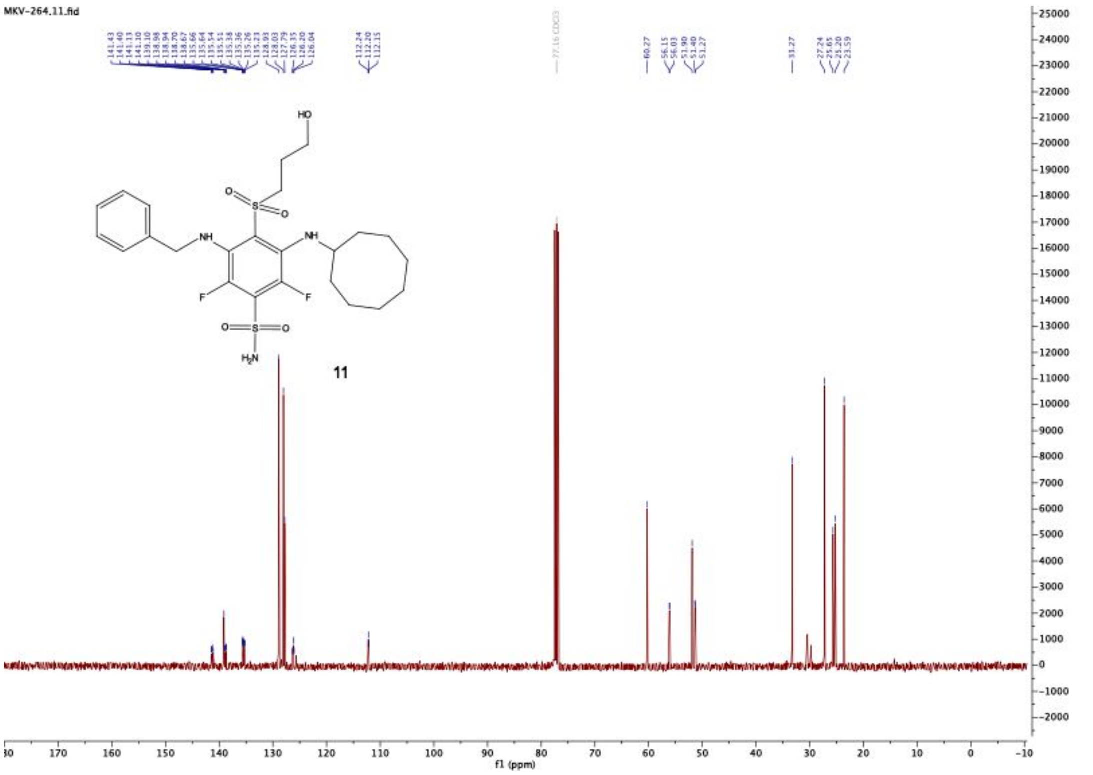

Figure S61. Compound **11** ^{19}F NMR:

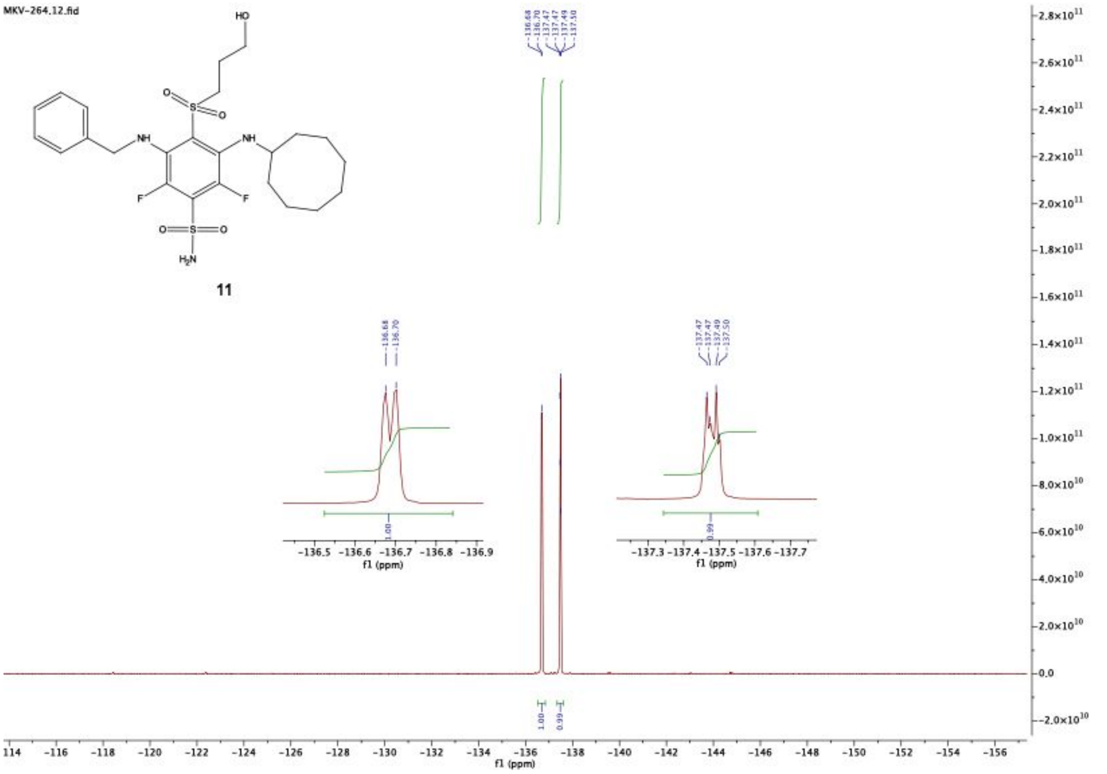

Figure S62. Compound **11** HRMS:

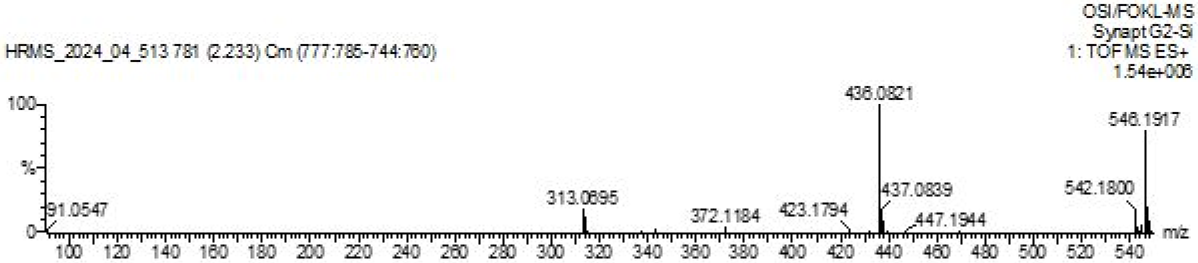

Figure S63. Compound **12** ^{1}H NMR:

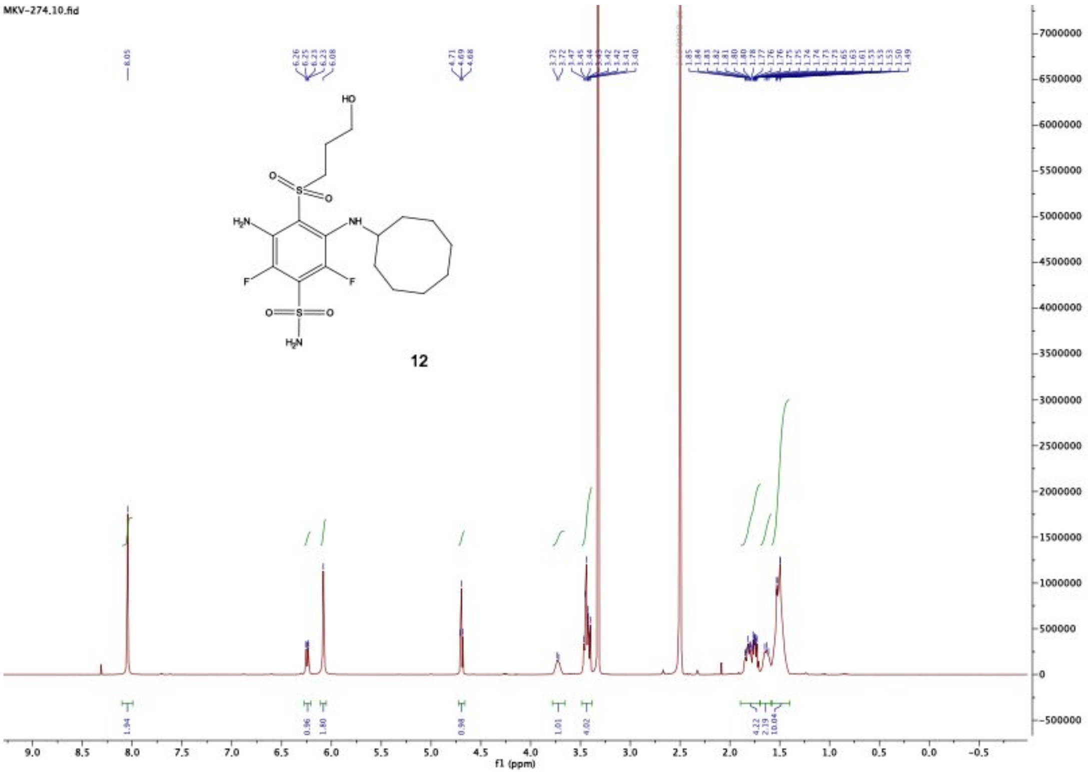

Figure S64. Compound **12** ^{13}C NMR:

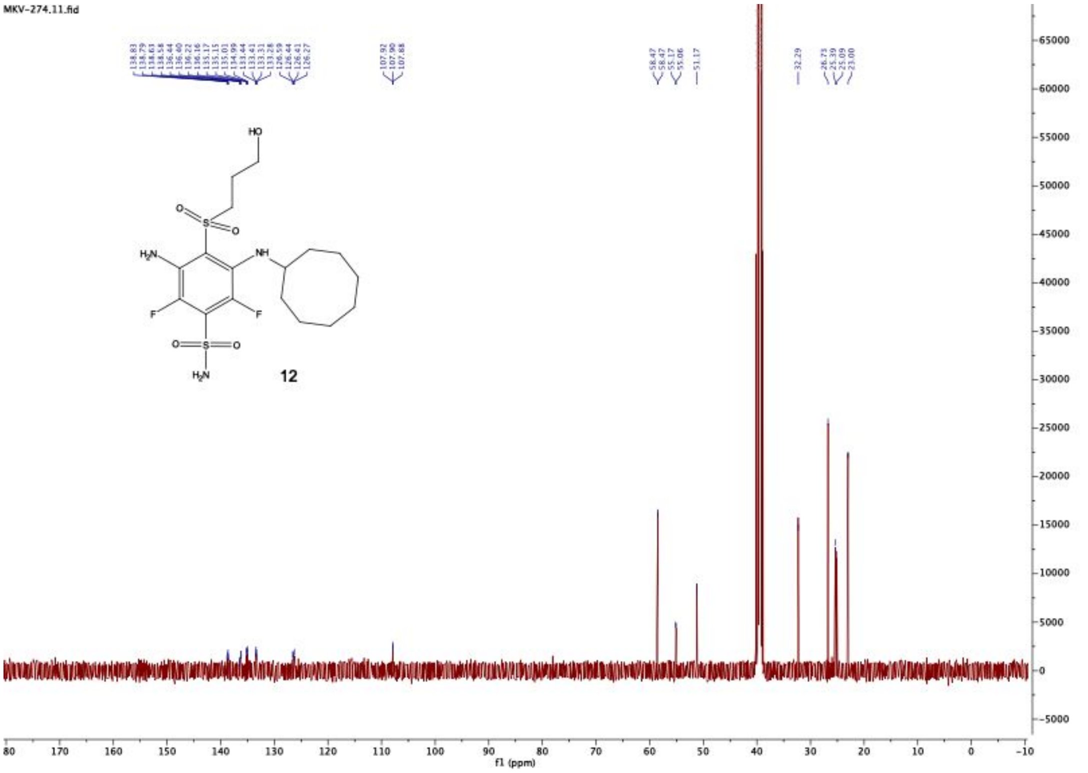

Figure S65. Compound **12** ^{19}F NMR:

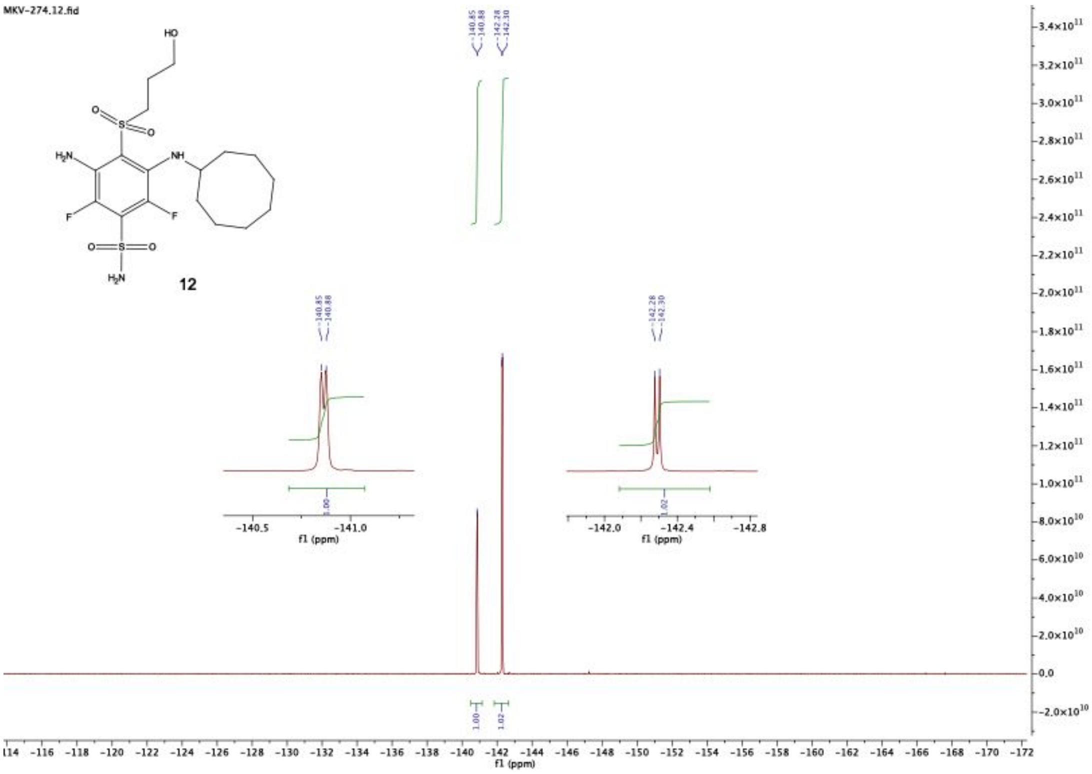

Figure S66. Compound **12** HRMS:

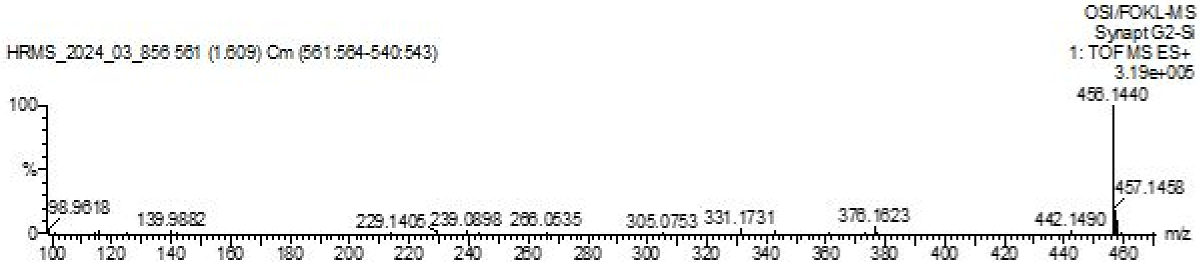

Figure S67. Compound **13** ^{1}H NMR:

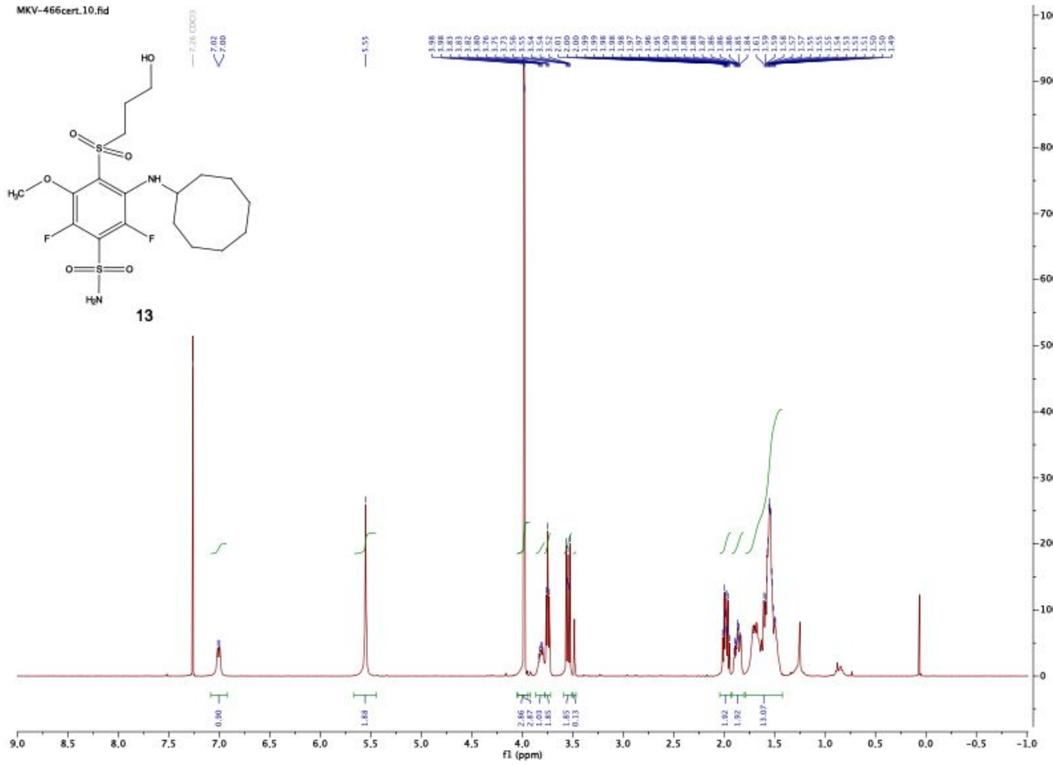

Figure S68. Compound **13** ^{13}C NMR:

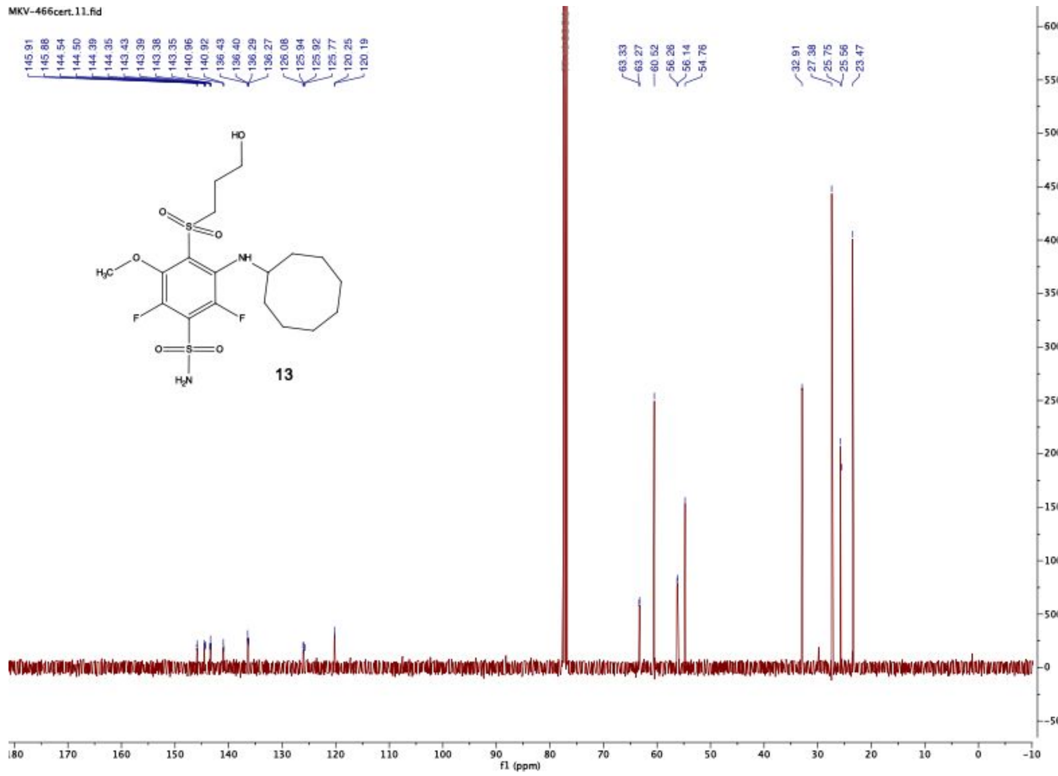

Figure S69. Compound **13** ^{19}F NMR:

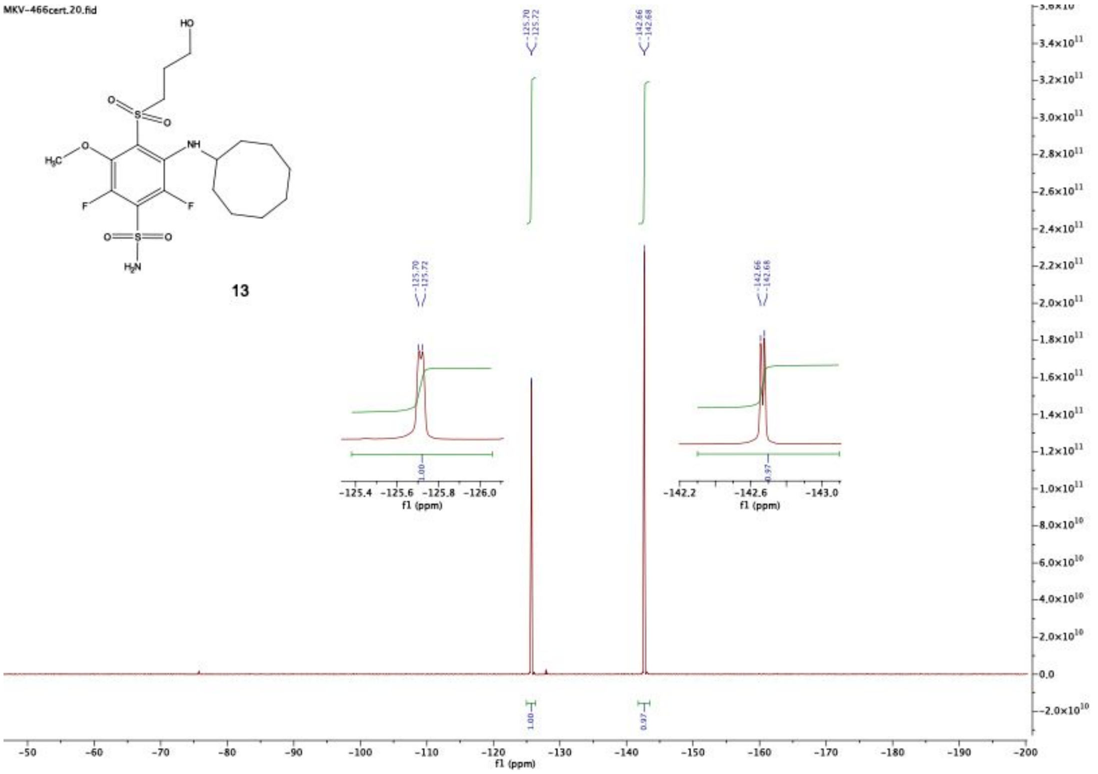

Figure S70. Compound **13** HRMS:

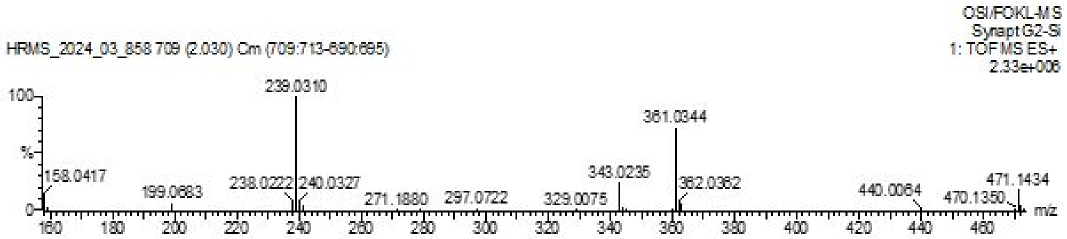

Figure S71. Compound **14** ^{1}H NMR:

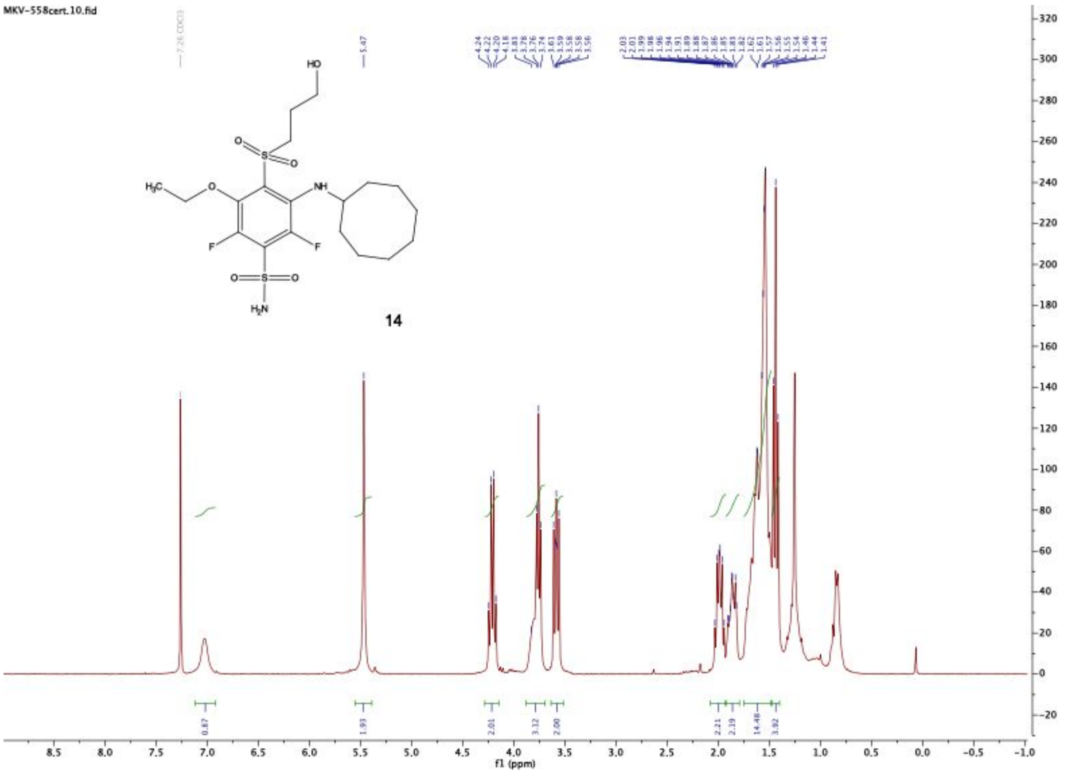

Figure S72. Compound **14** ^{13}C NMR:

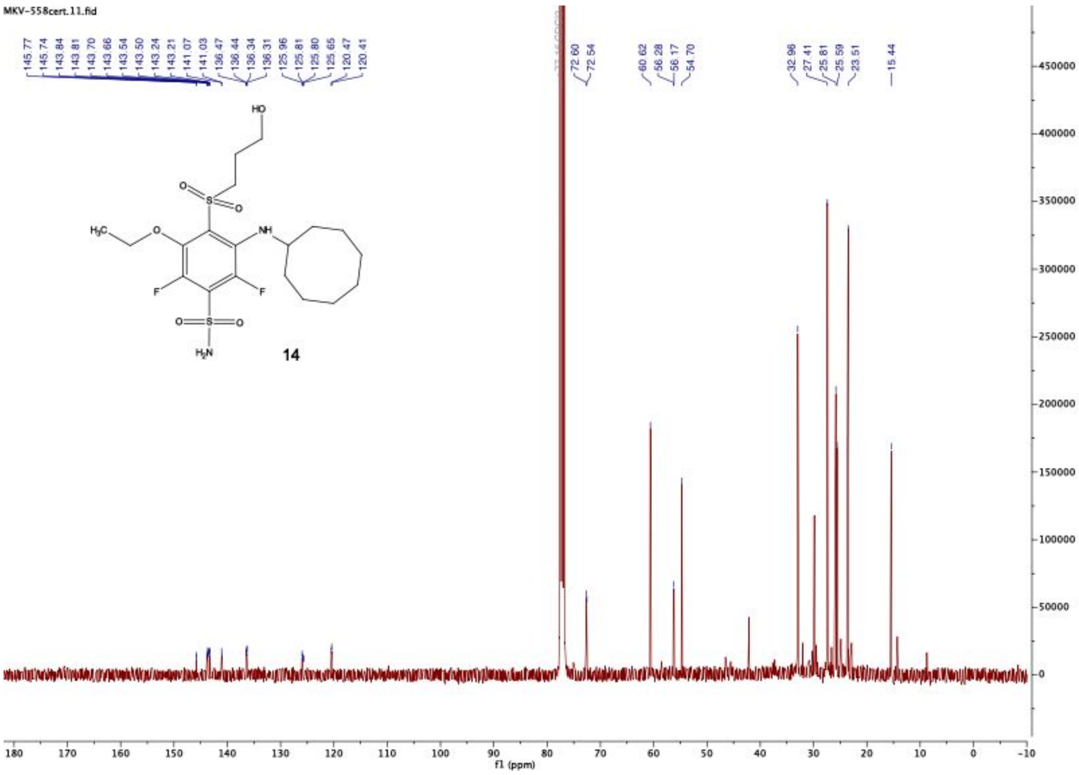

Figure S73. Compound **14** ^{19}F NMR:

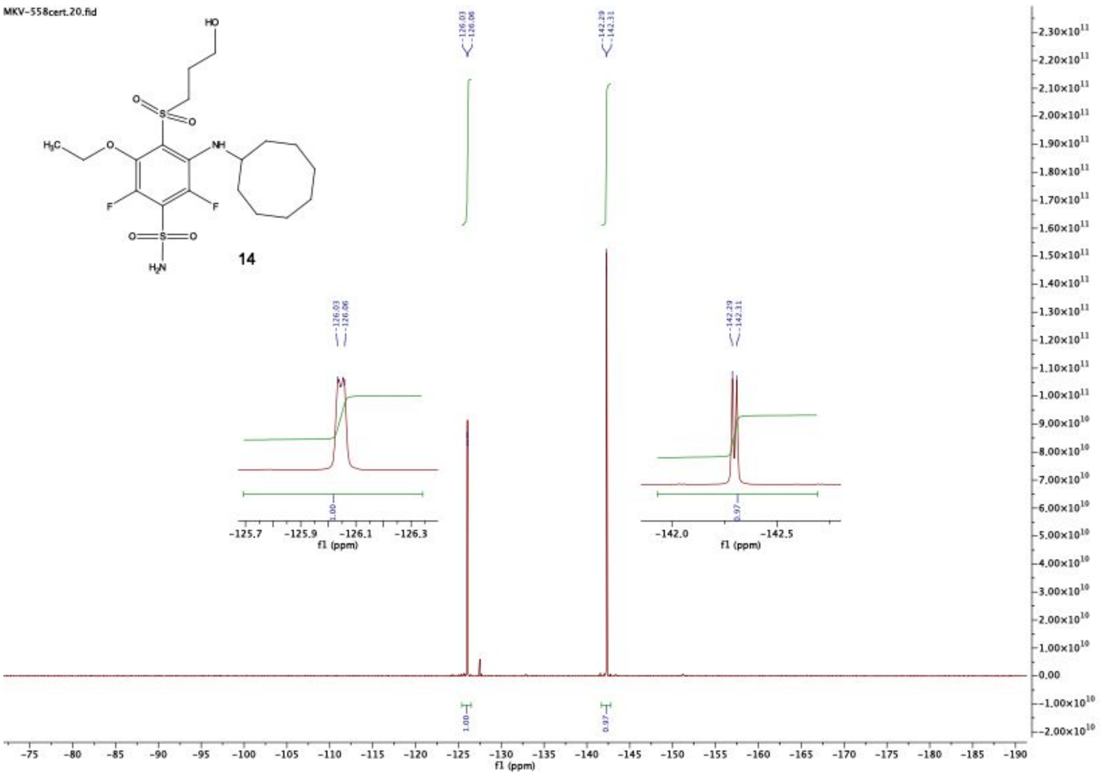

Figure S74. Compound **14** HRMS:

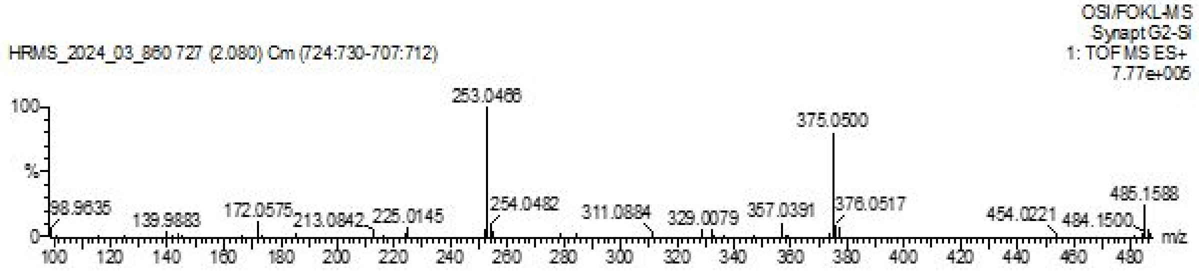

Figure S75. Compound **15** ^{1}H NMR:

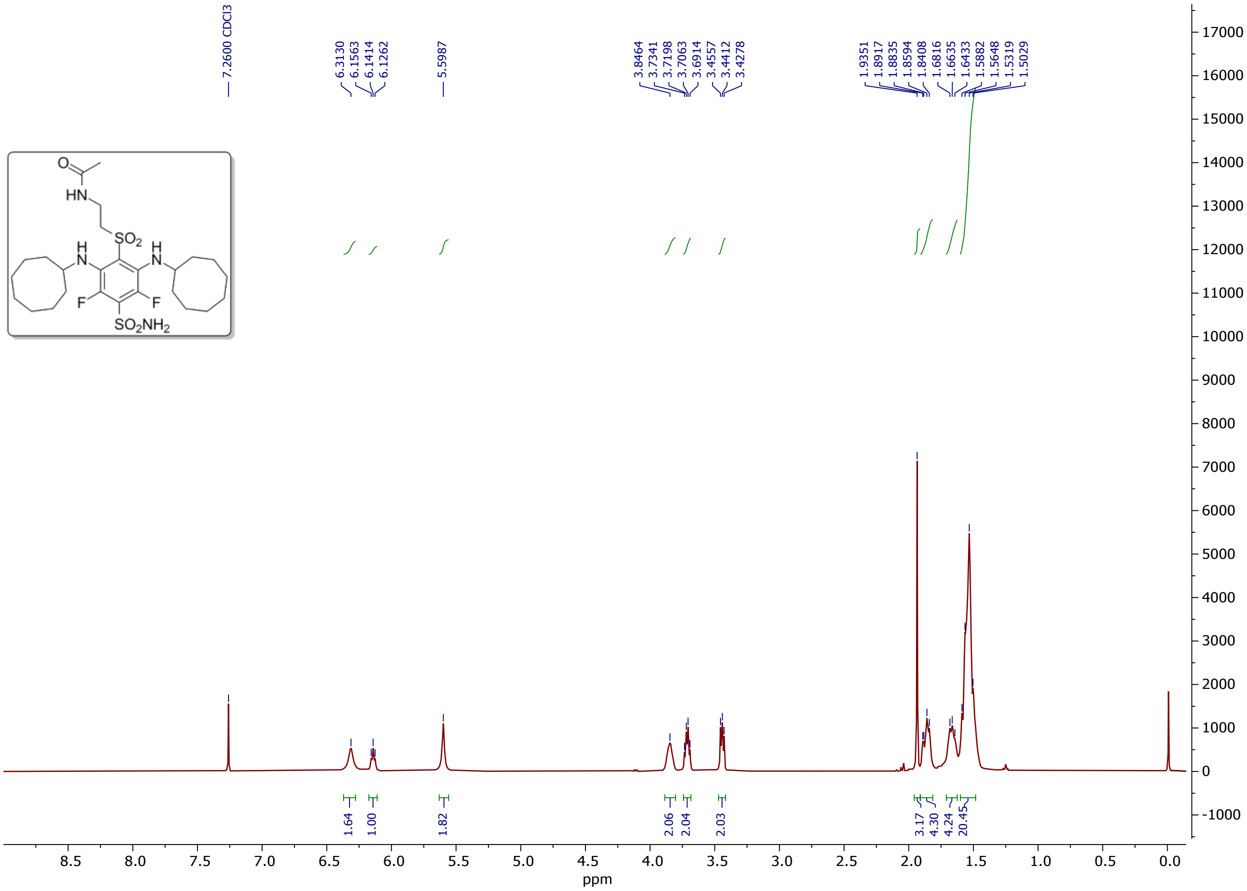

Figure S76. Compound **15** ^{13}C NMR:

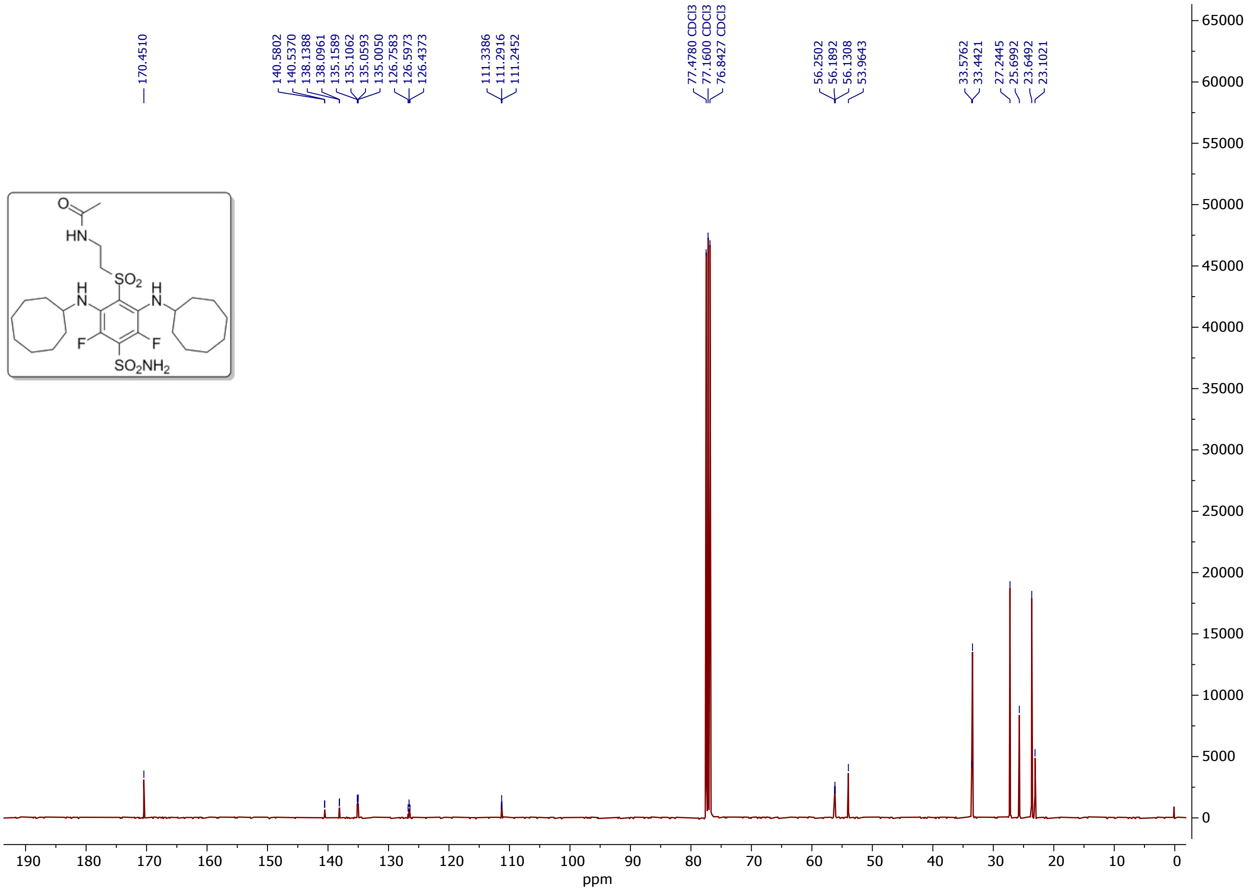

Figure S77. Compound **15** ^{19}F NMR:

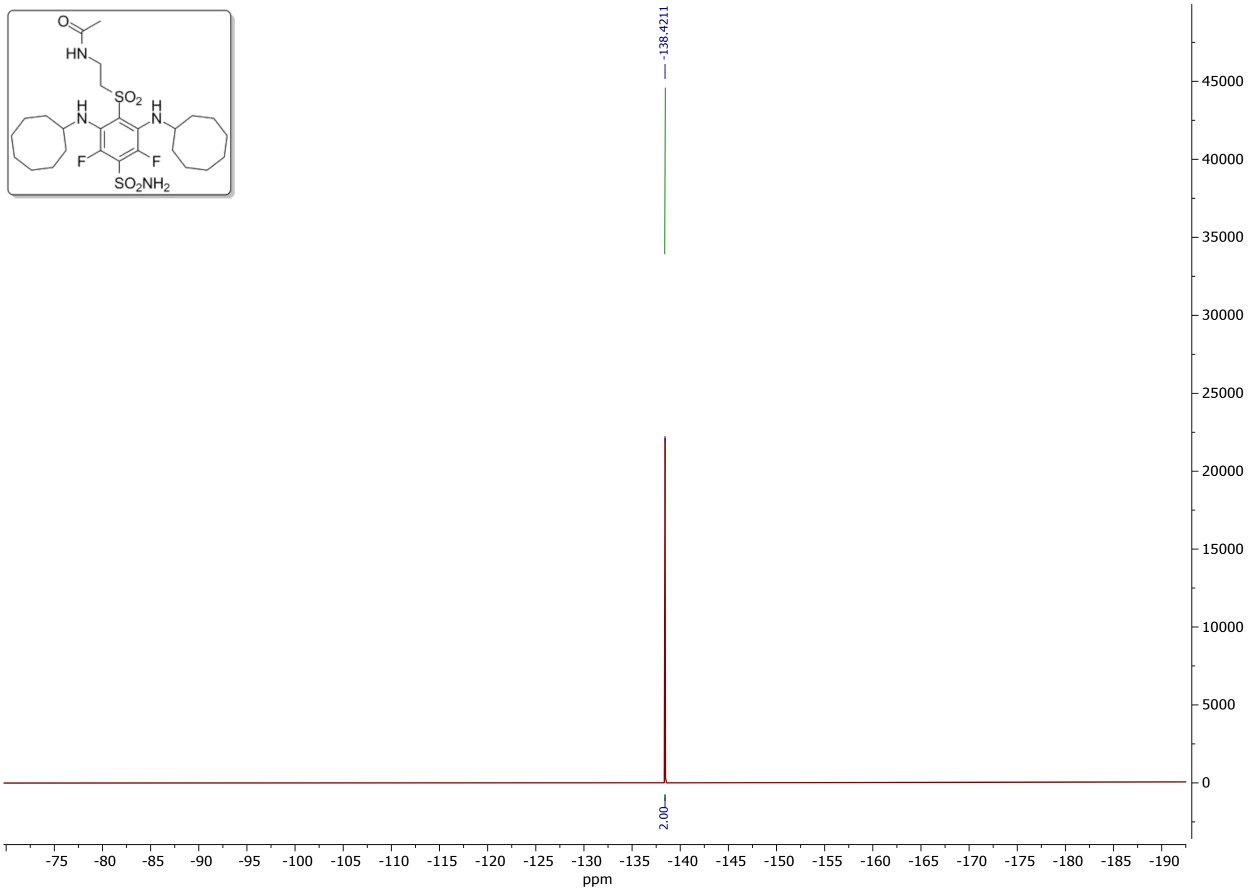

Figure S78. Compound **15** HRMS:

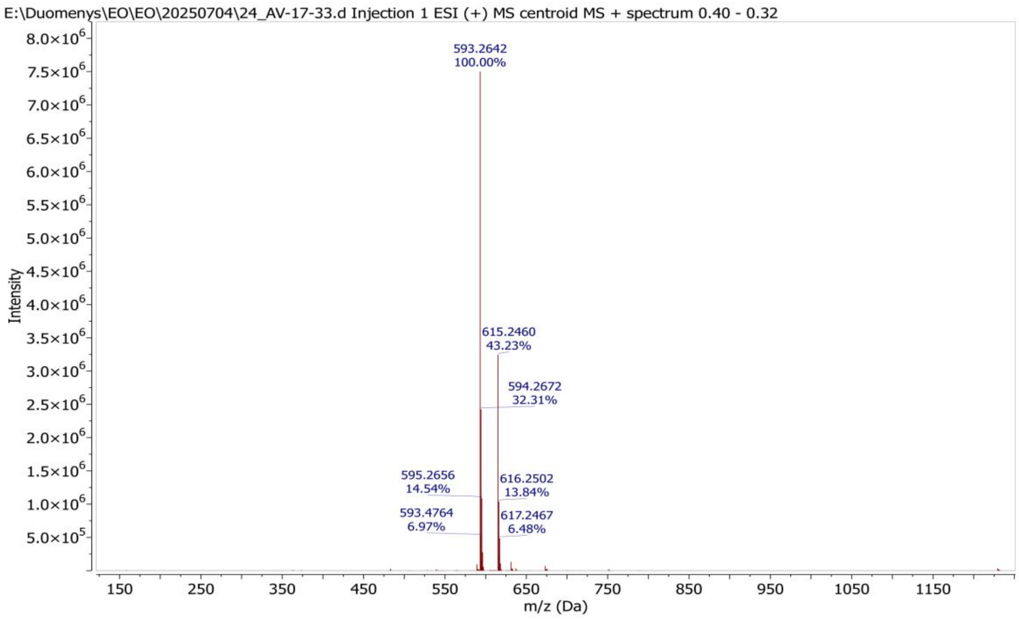

Figure S79. Compound **16** ^{1}H NMR:

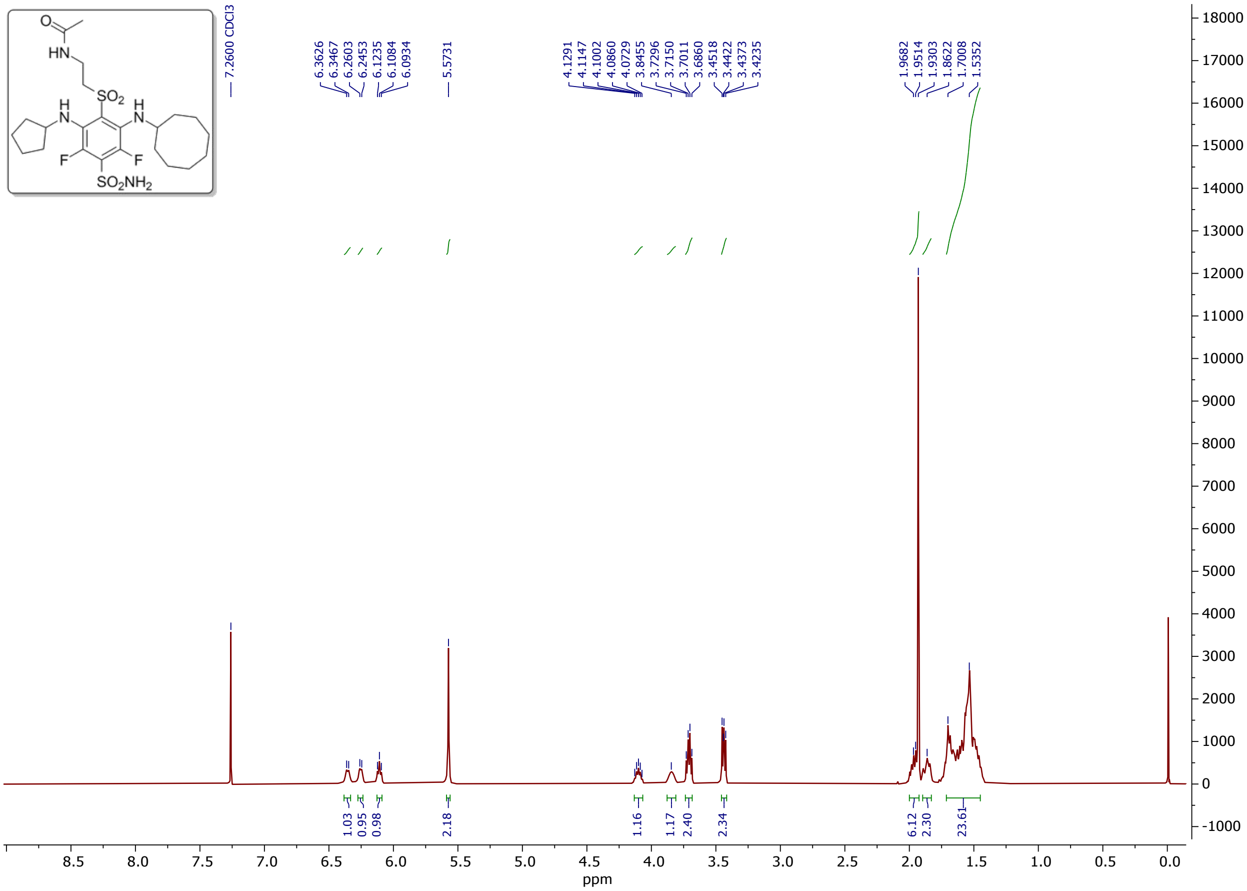

Figure S80. Compound **16** ^{13}C NMR:

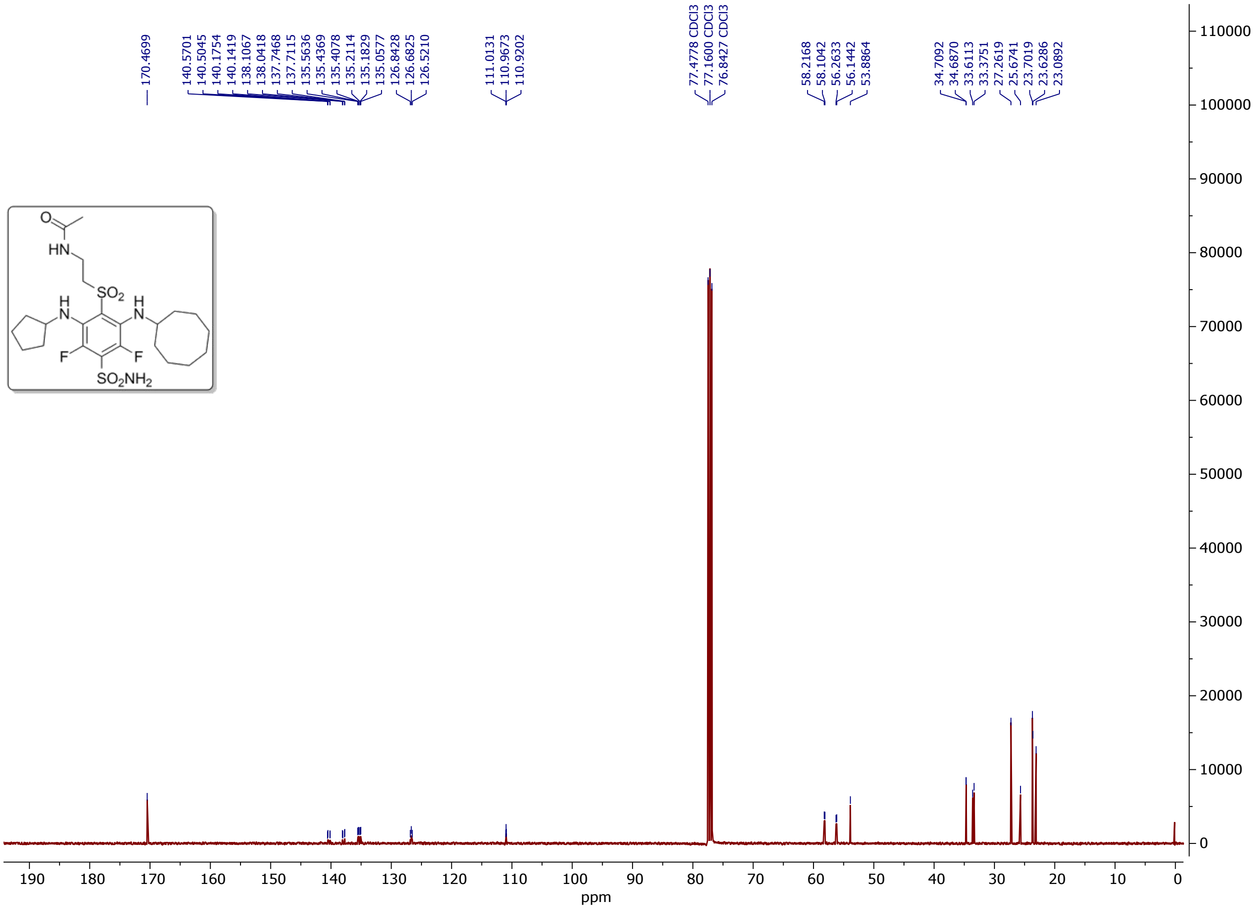

Figure S81. Compound **16** ^{19}F NMR:

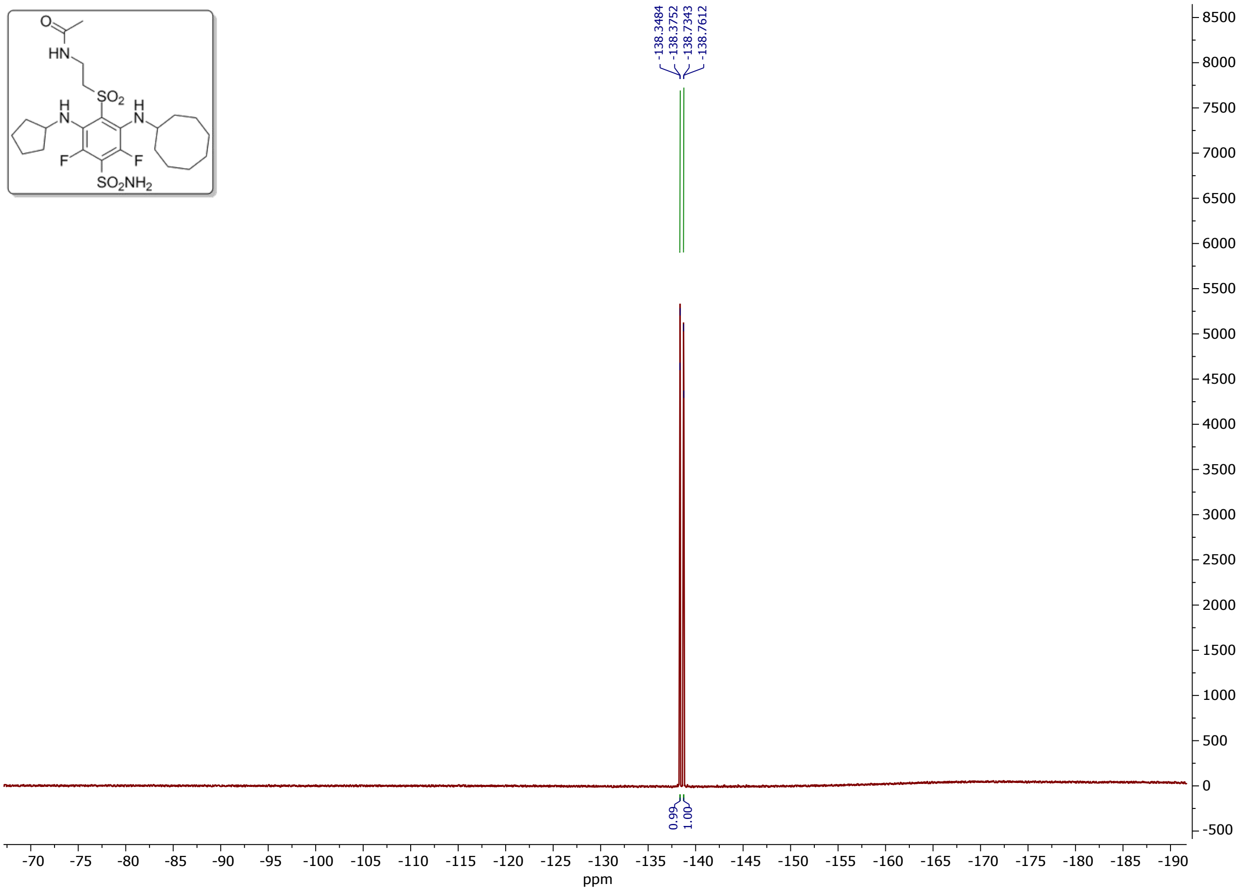

Figure S82. Compound **16** HRMS:

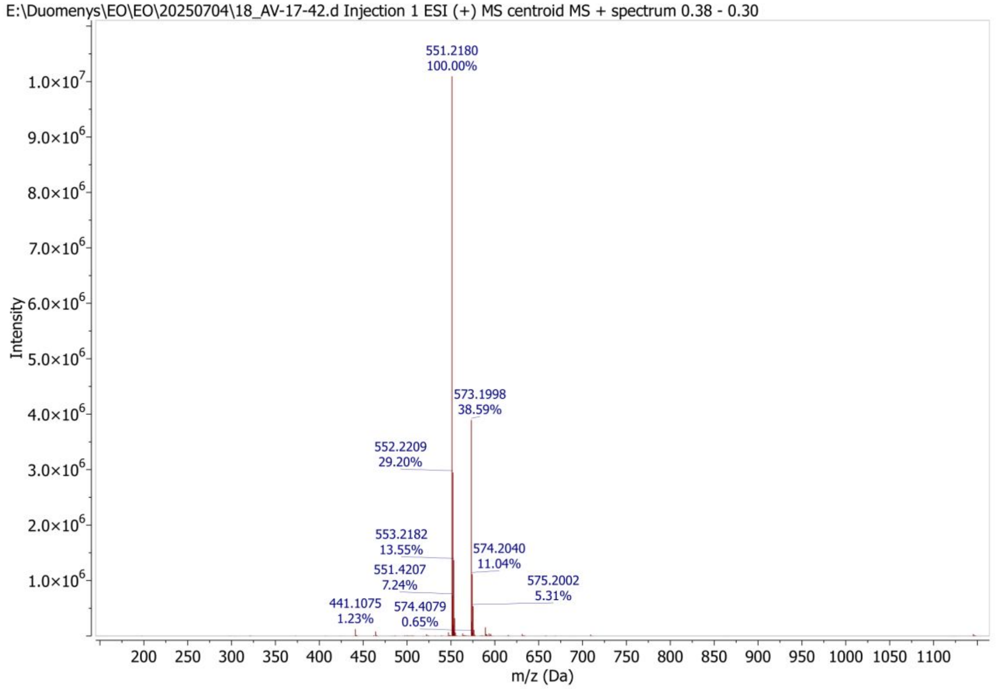

Figure S83. Compound **17** ^{1}H NMR:

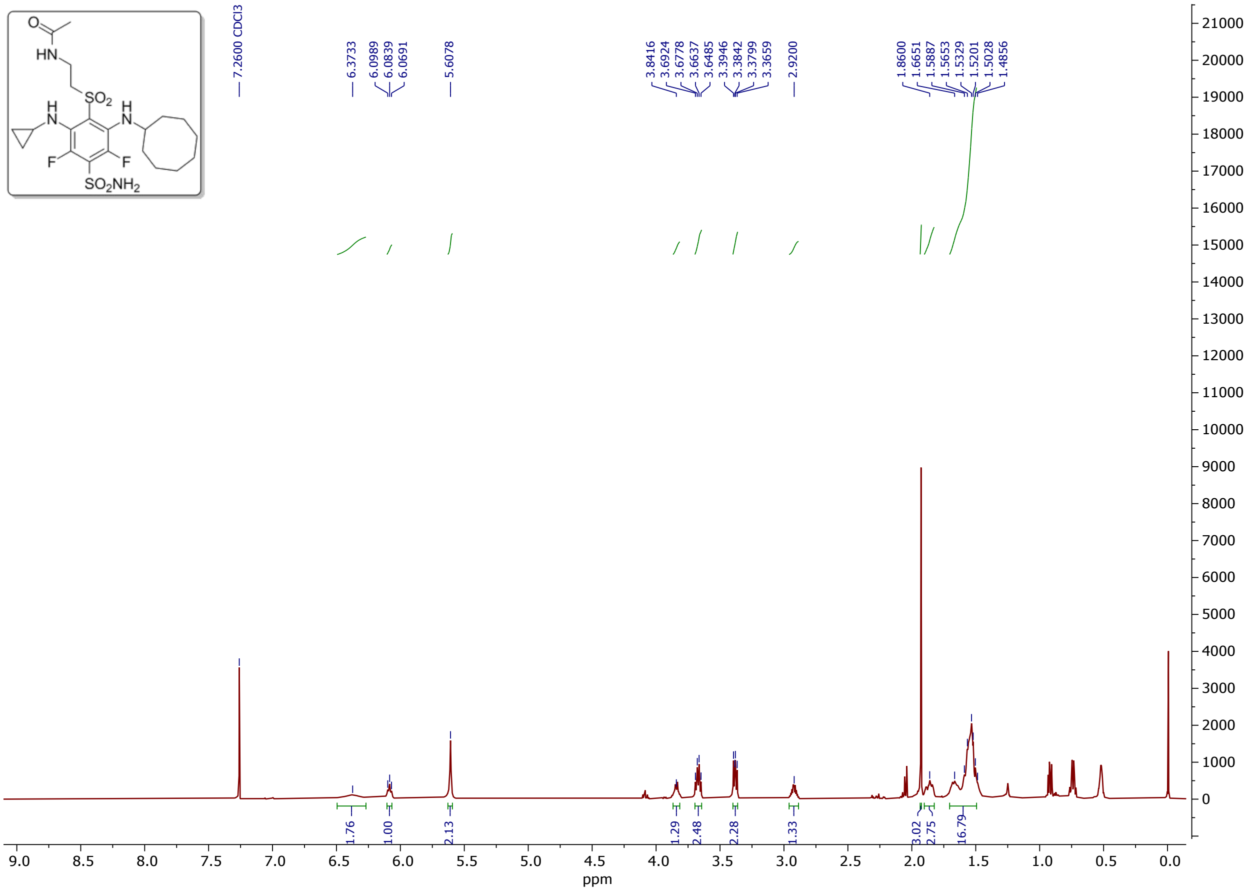
7.2600 CDCl3
6.3733
6.0989
6.0839
6.0691
5.6078
3.8416
3.6924
3.6778
3.6637
3.6485
3.3946
3.3842
3.3799
3.3659
2.9200
1.8600
1.6651
1.5887
1.5653
1.5329
1.5201
1.5028
1.4856
1.76
1.00
2.13
1.29
2.48
2.28
1.33
3.02
2.75
16.79
ppm

Figure S84. Compound **17** ^{13}C NMR:

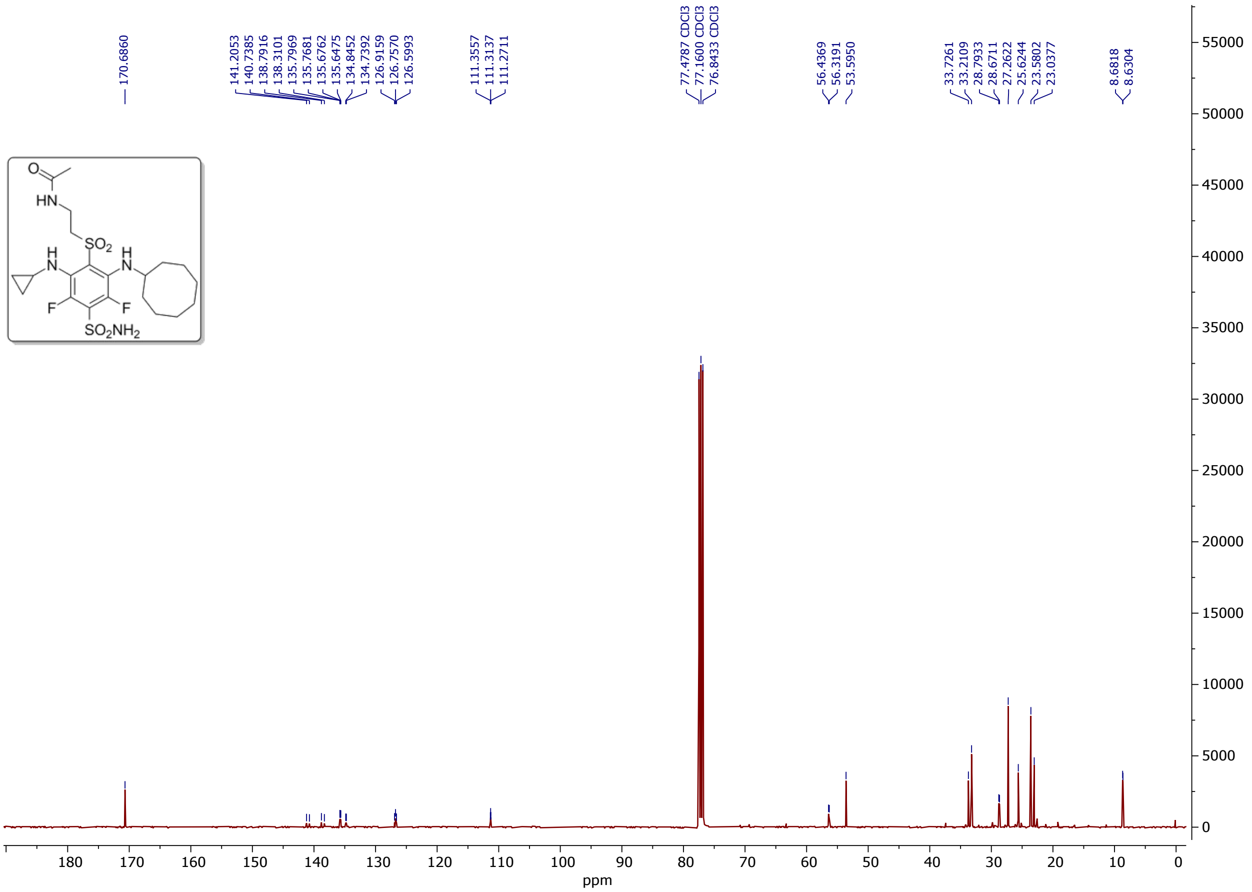

Figure S85. Compound **17** ^{19}F NMR:

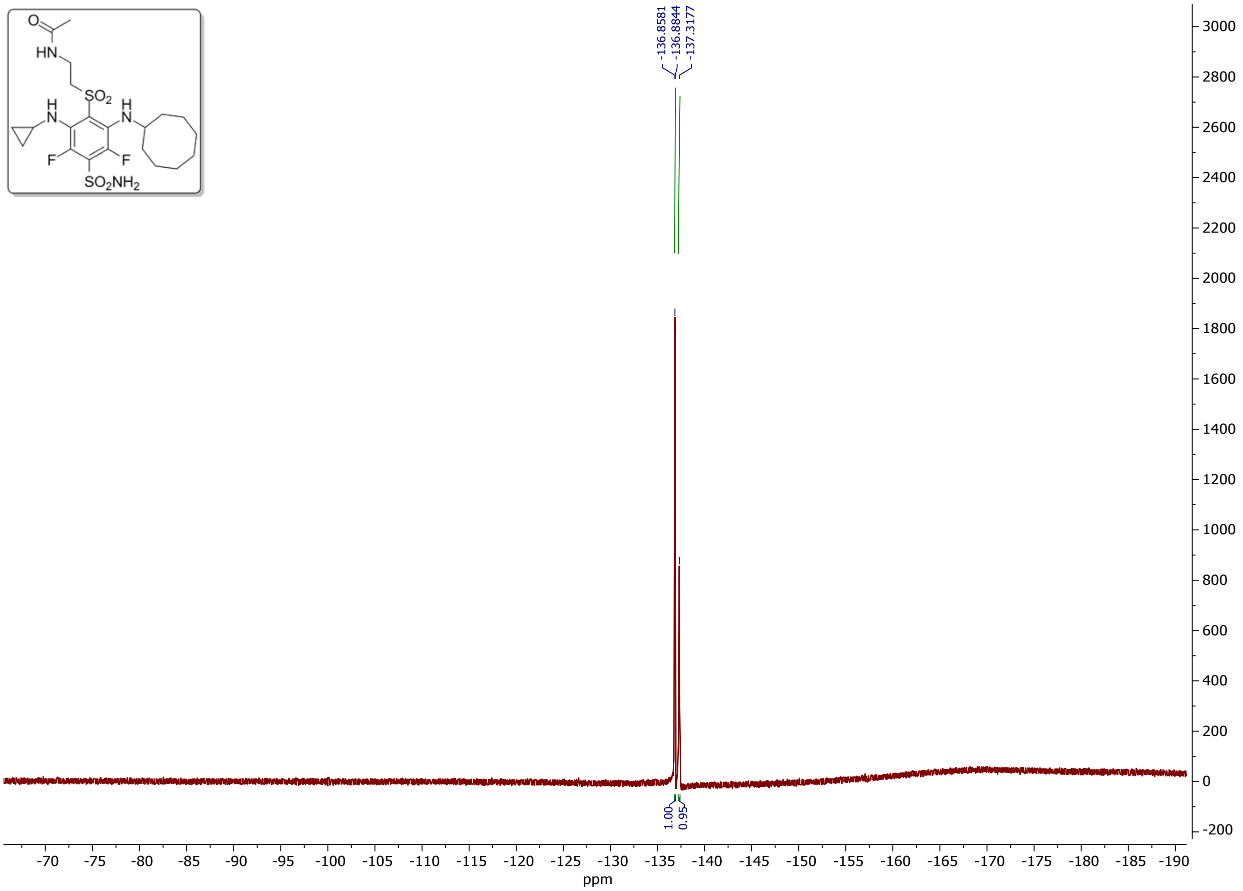

Figure S86. Compound **17** HRMS:

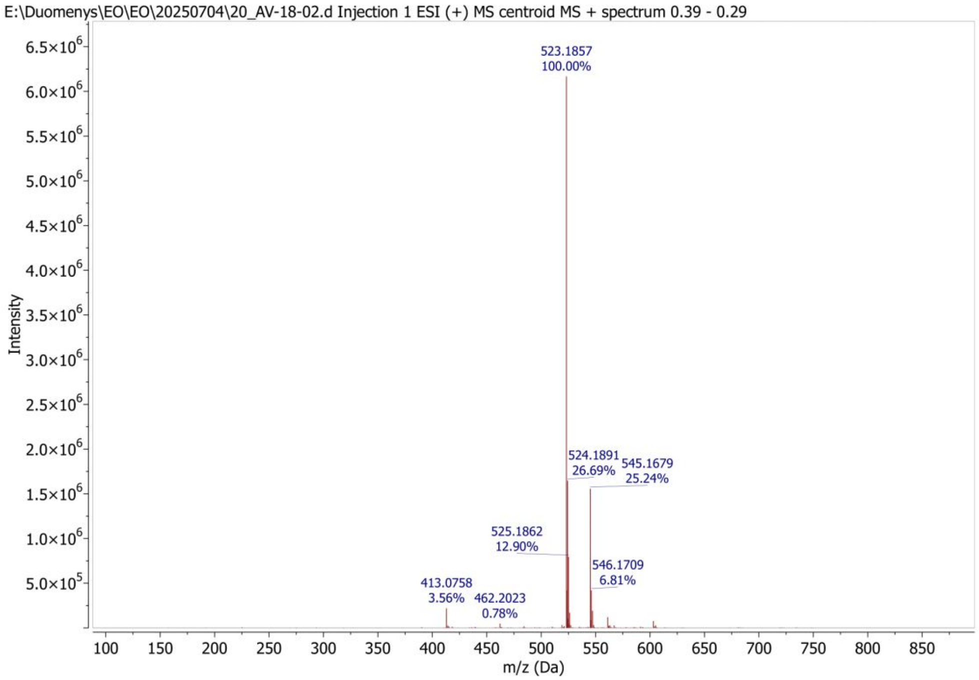

Figure S87. Compound **18** ^{1}H NMR:

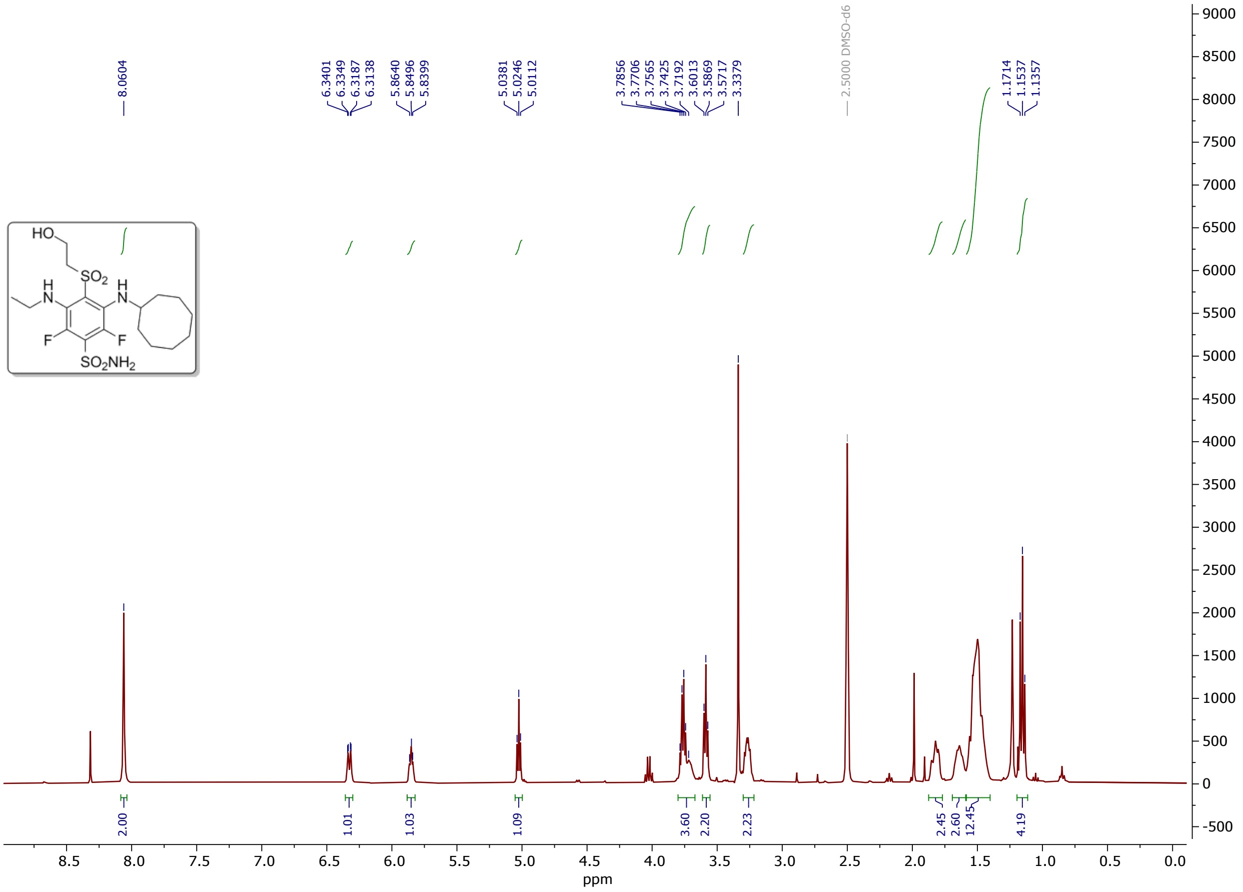

Figure S88. Compound **18** ^{13}C NMR:

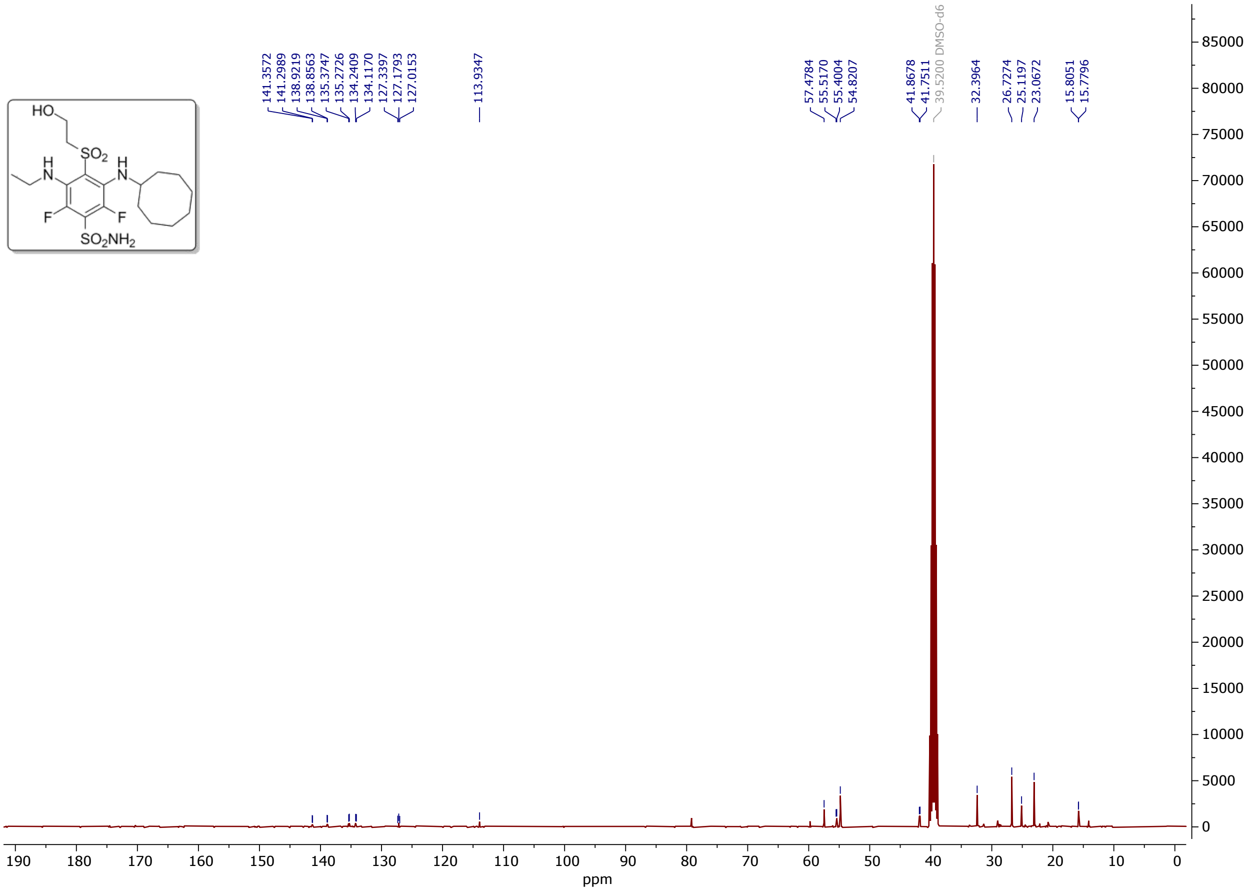

Figure S89. Compound **18** ^{19}F NMR:

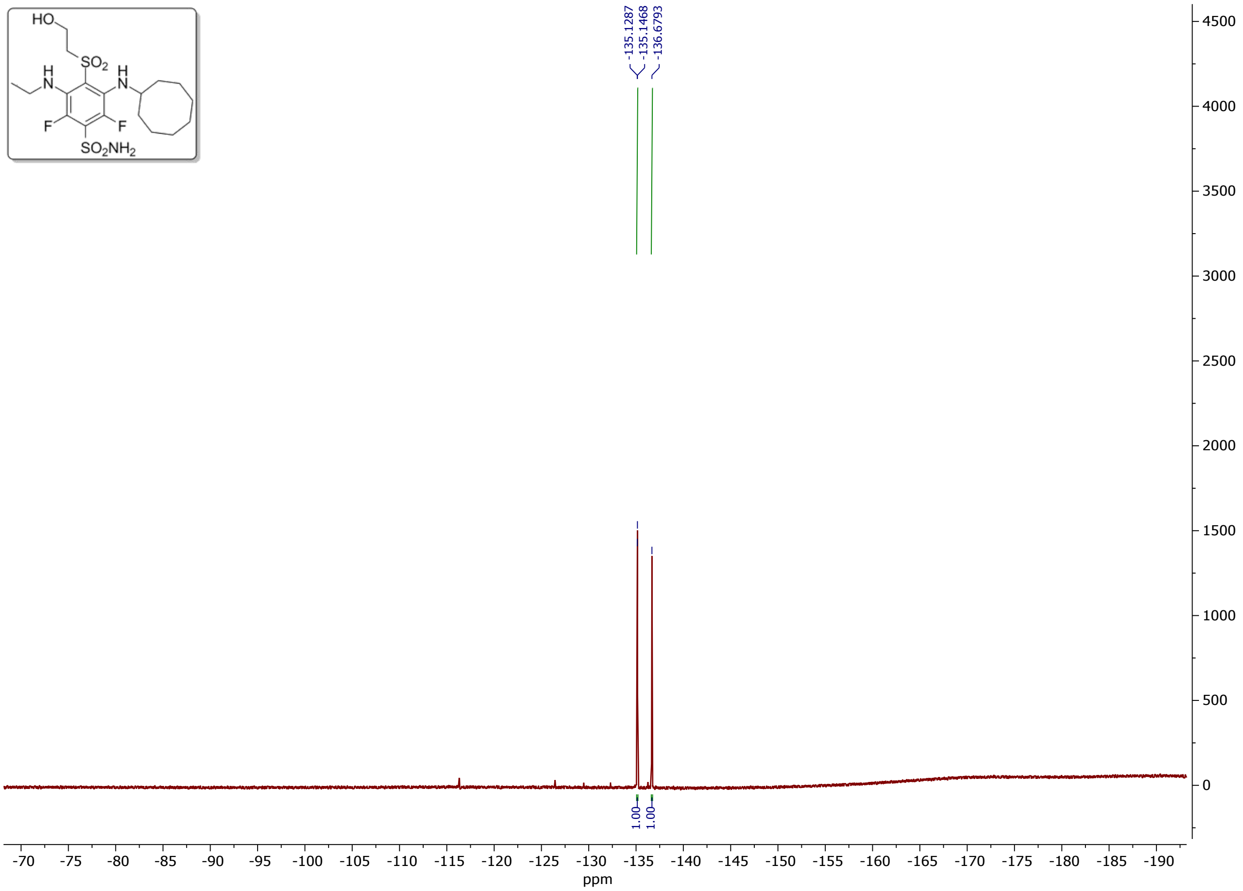

Figure S90. Compound **18** HRMS:

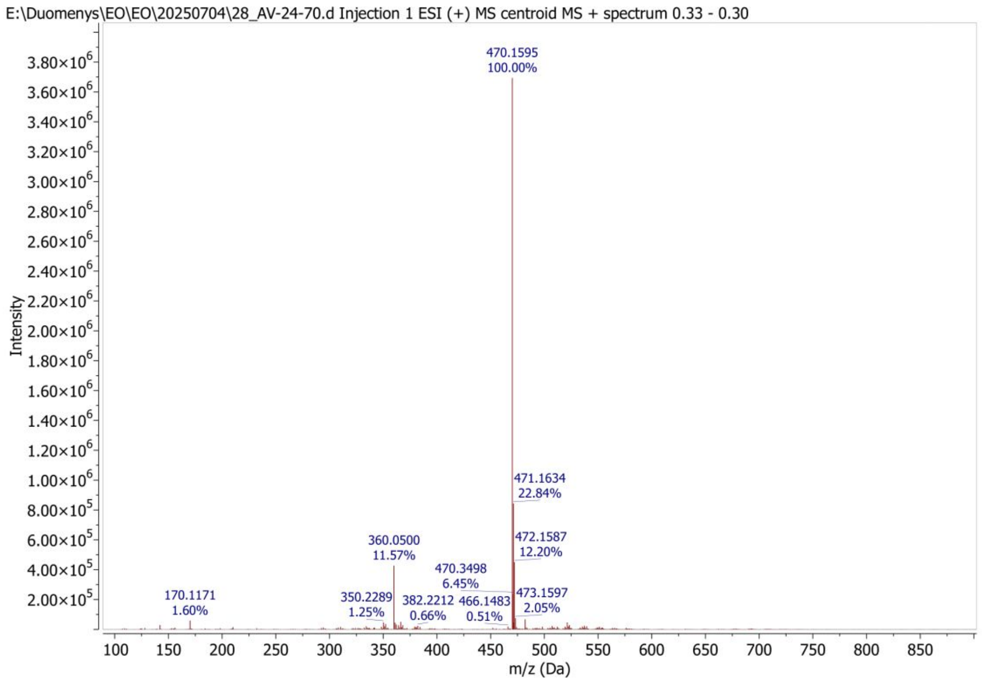

Figure S91. Compound **19** ^{1}H NMR:

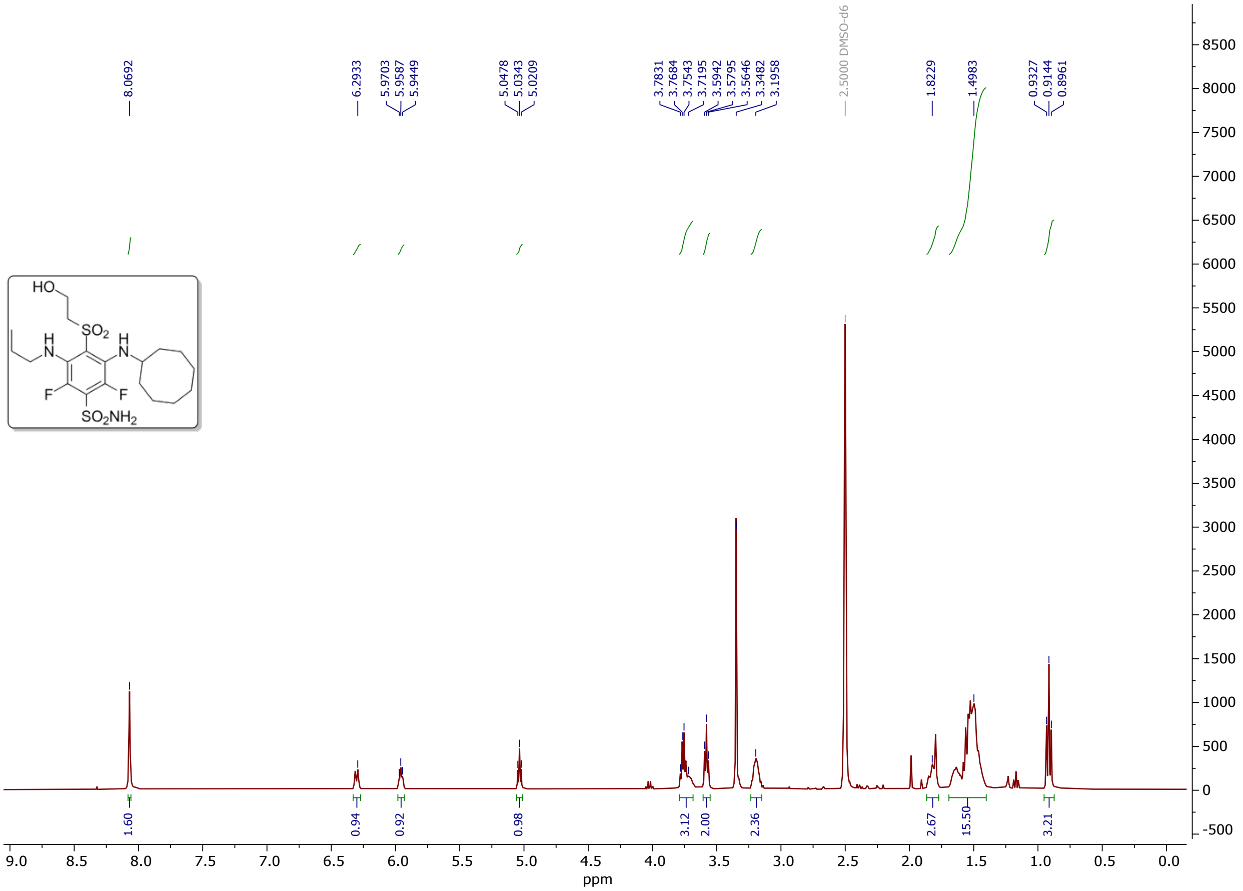

Figure S92. Compound **19** ^{13}C NMR:

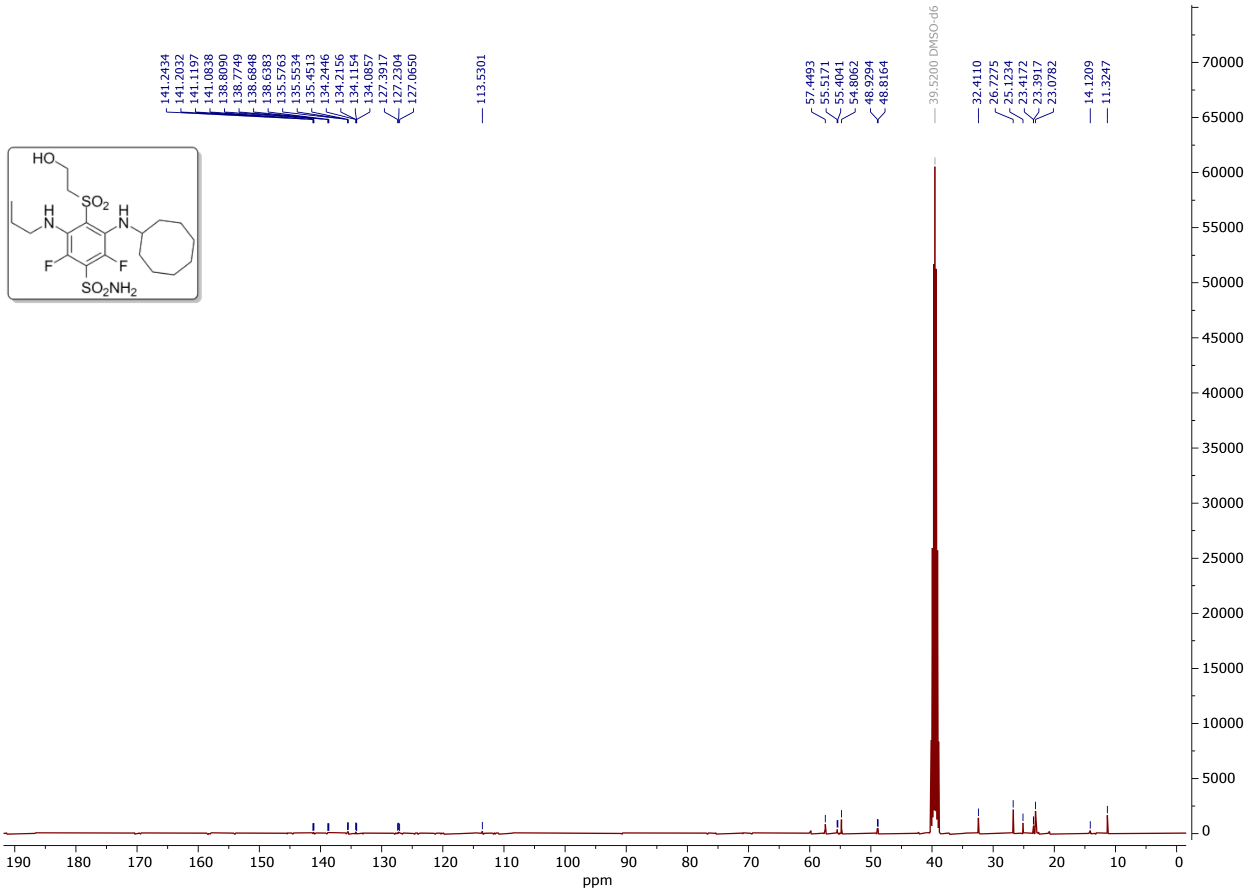

Figure S93. Compound **19** ^{19}F NMR:

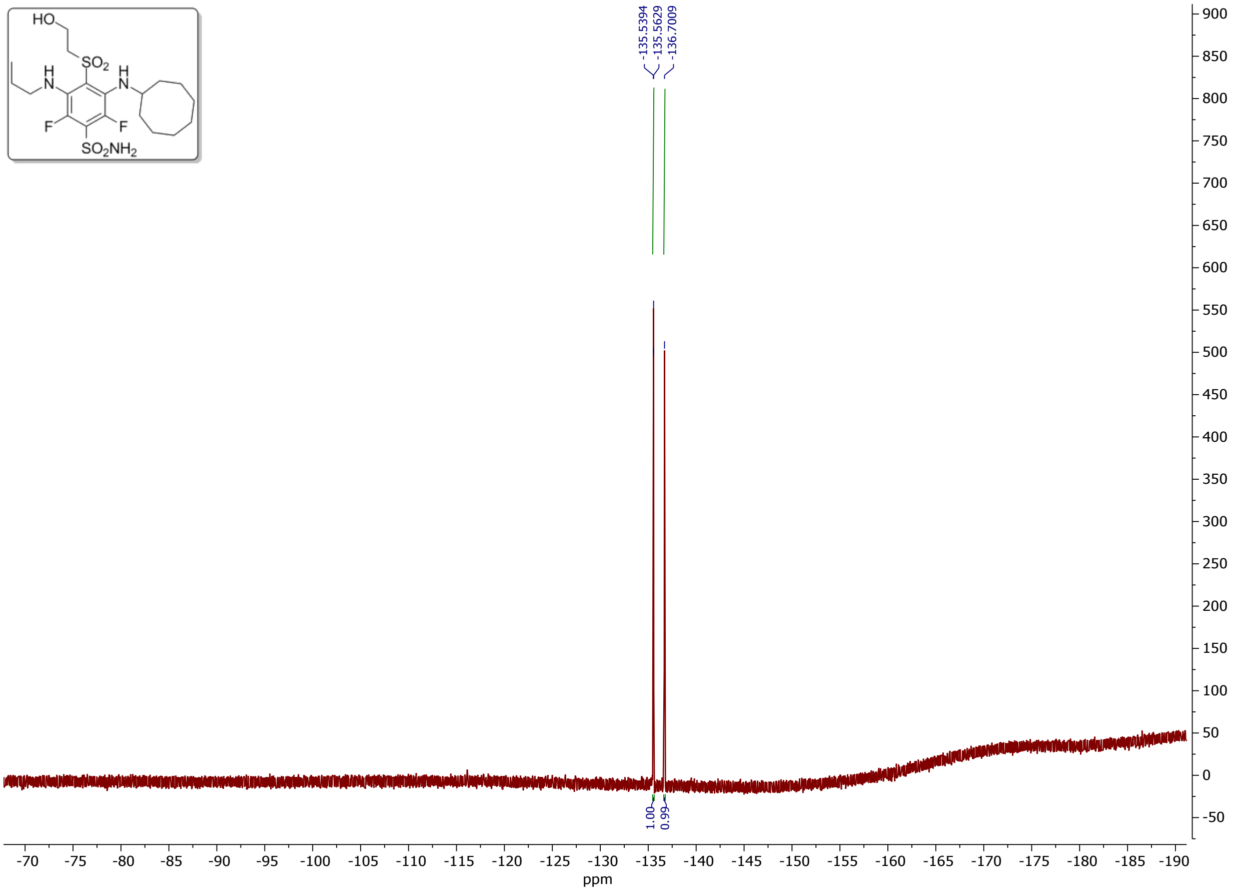

Figure S94. Compound **19** HRMS:

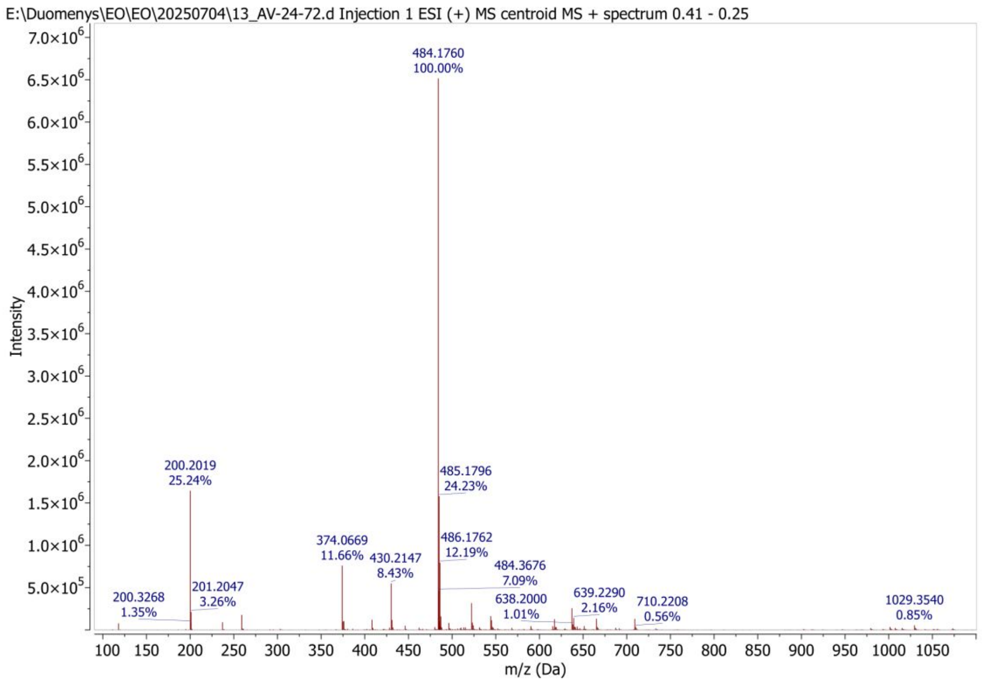

Figure S95. Compound **21** ^{1}H NMR:

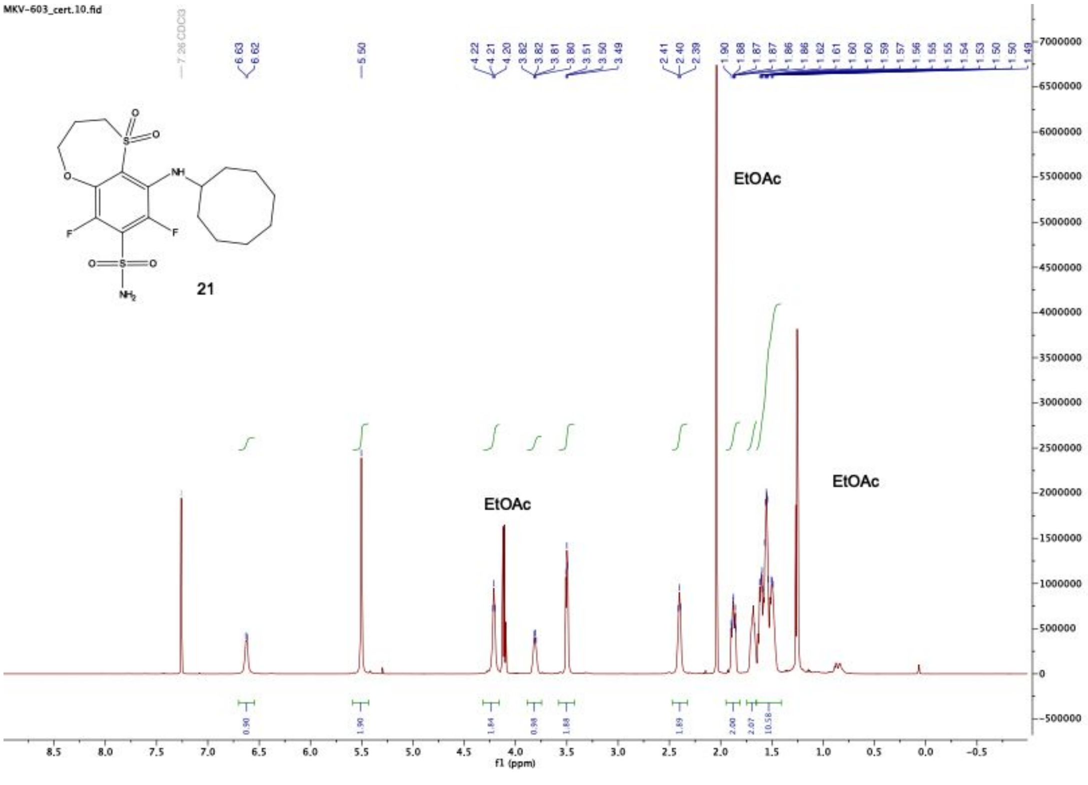

Figure S96. Compound **21** ^{13}C NMR:

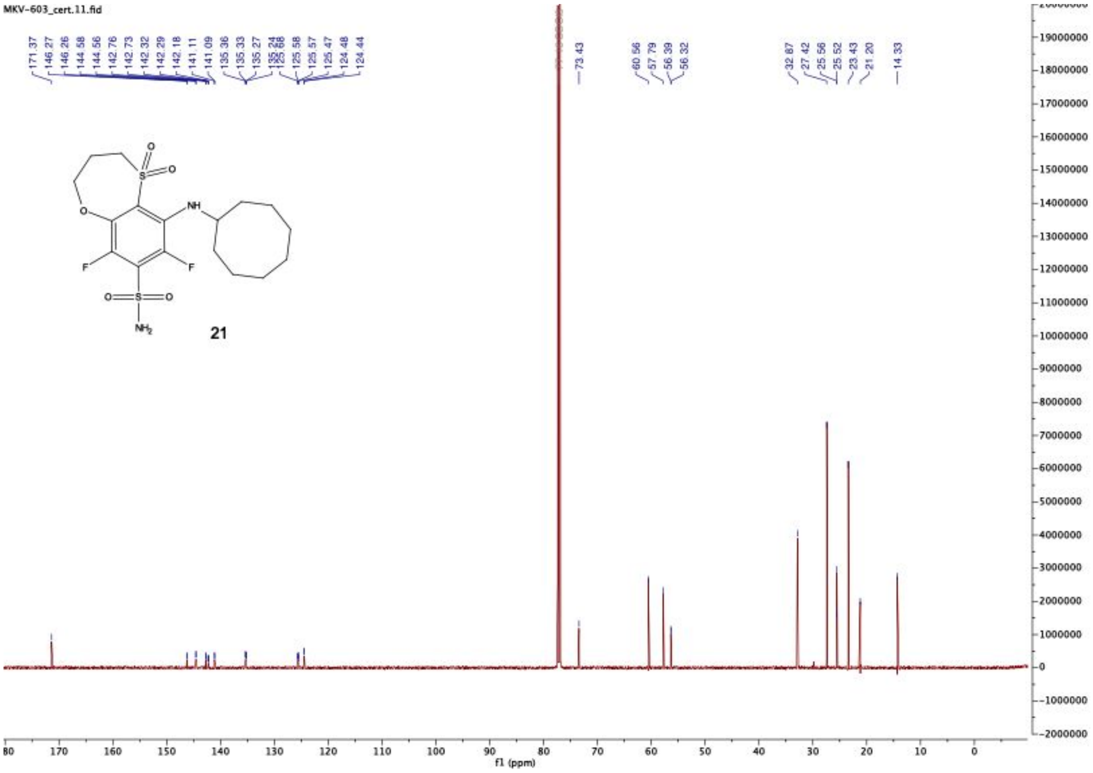

Figure S97. Compound **21** ^{19}F NMR:

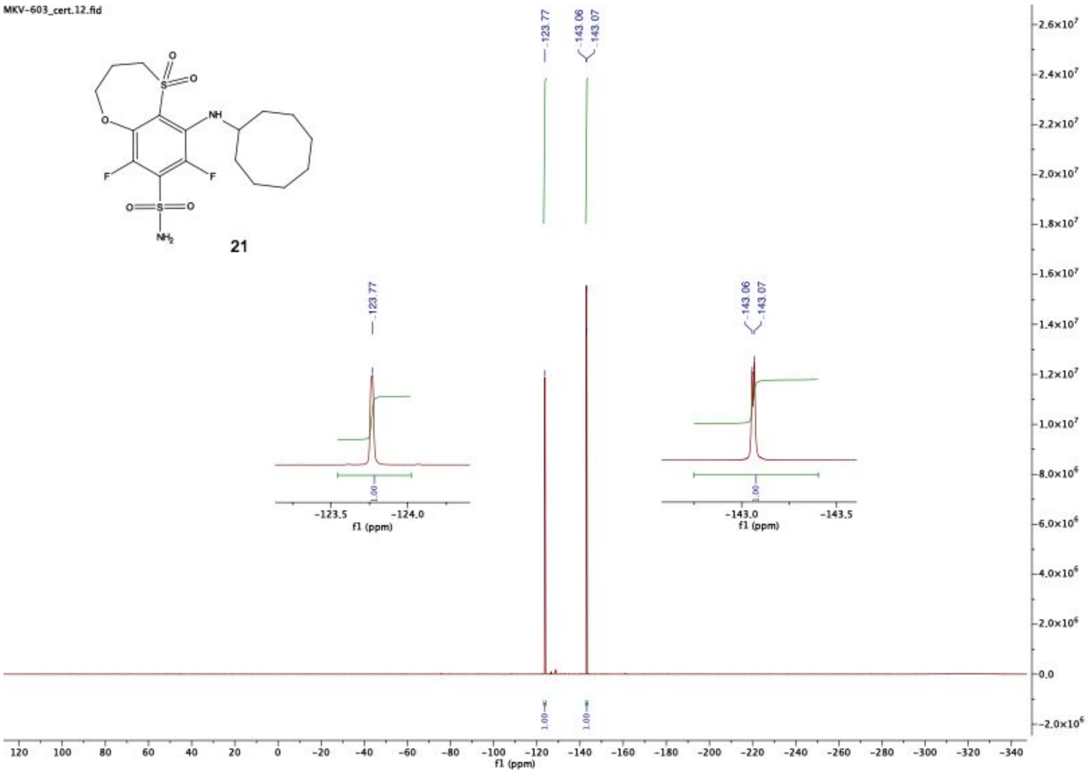

Figure S98. Compound **21** HRMS:

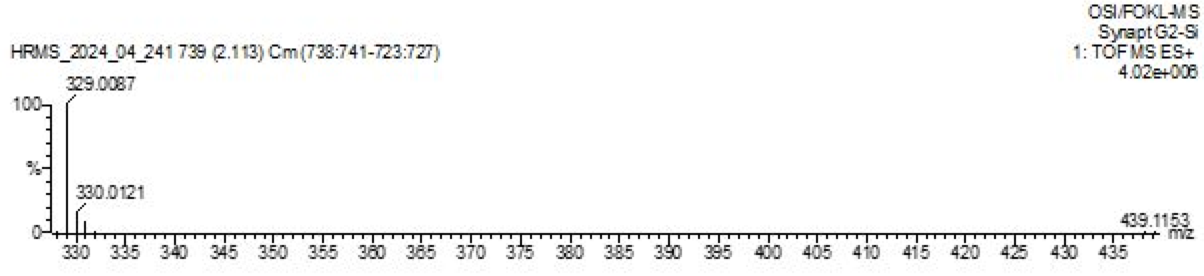

Figure S99. Compound **22** ^{1}H NMR:

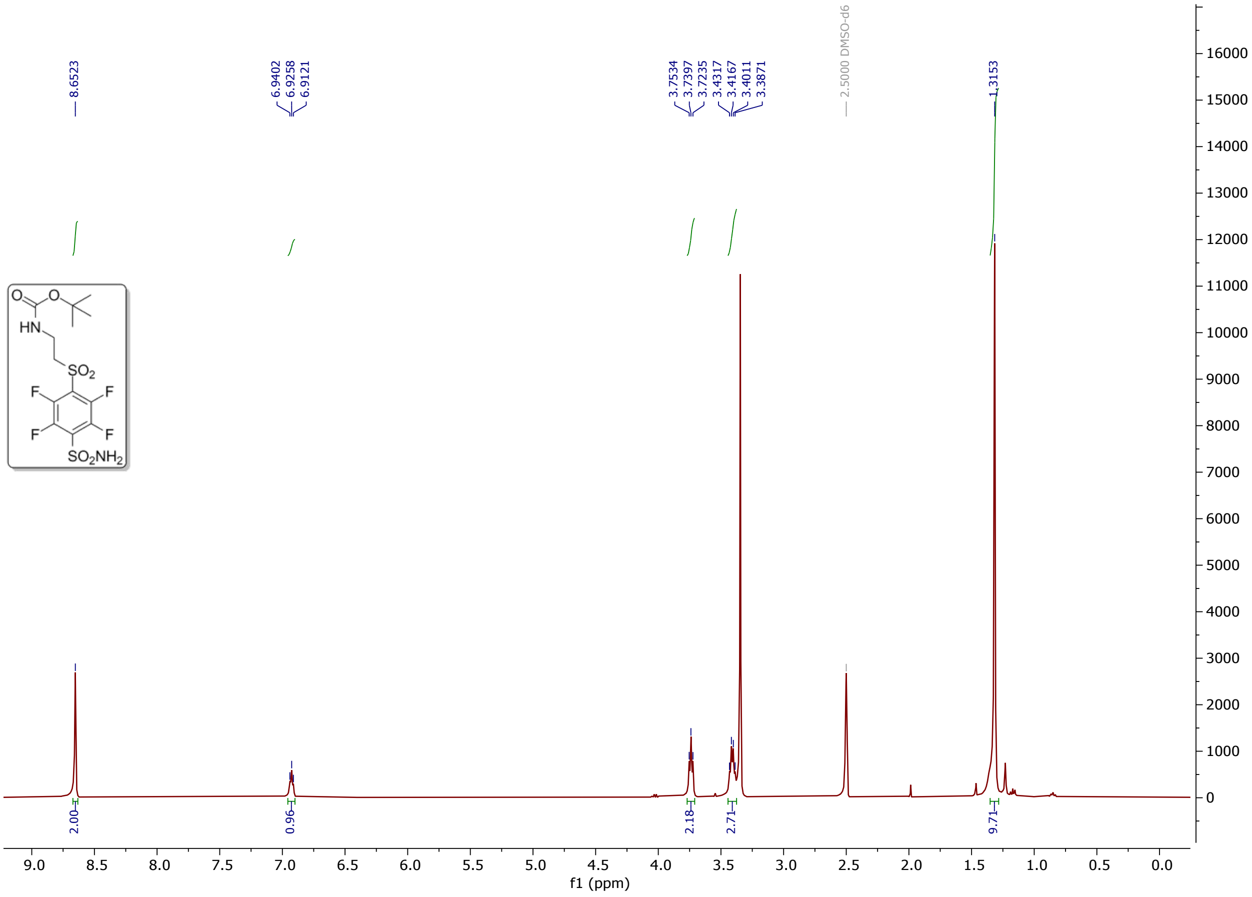

Figure S100. Compound **22** ^{13}C NMR:

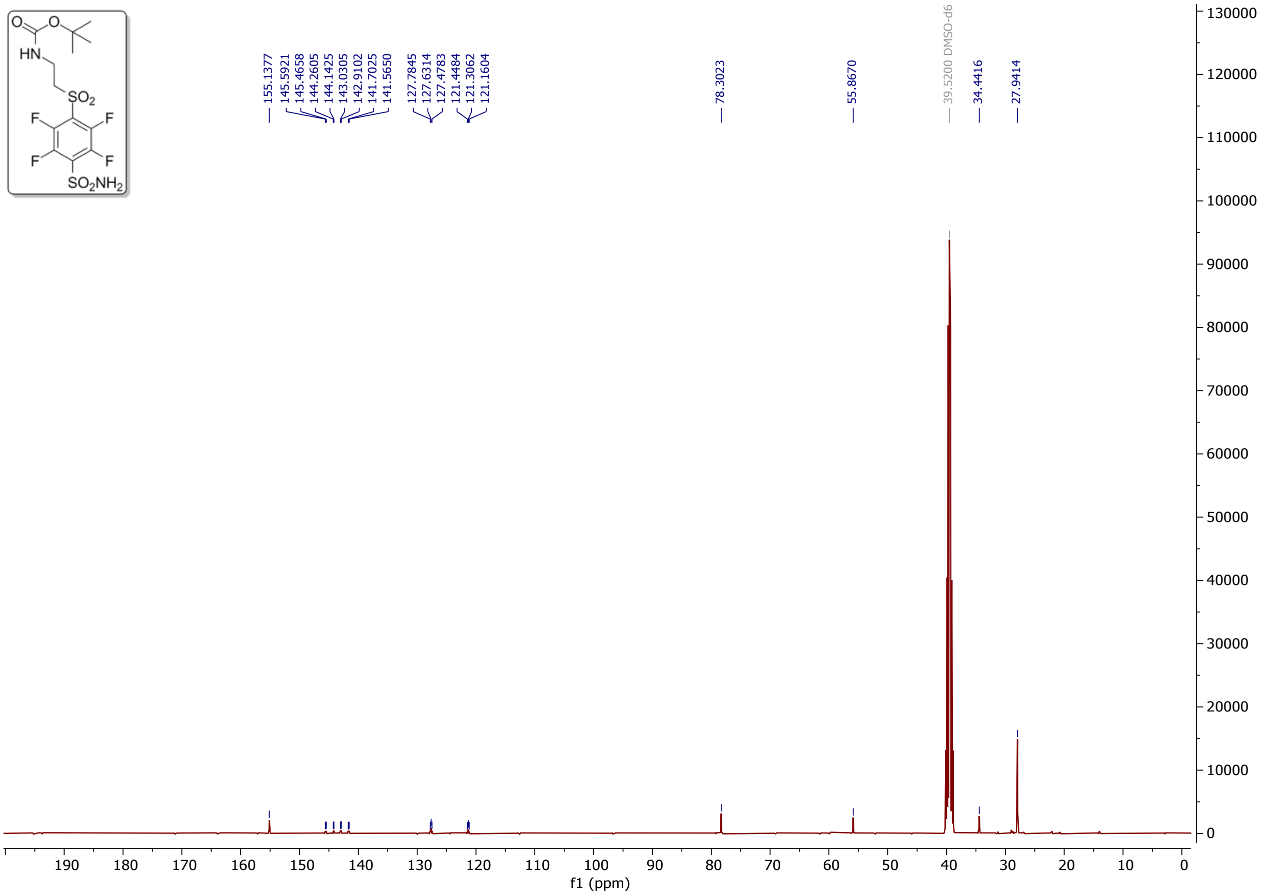

Figure S101. Compound **22** ^{19}F NMR:

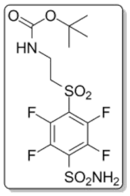

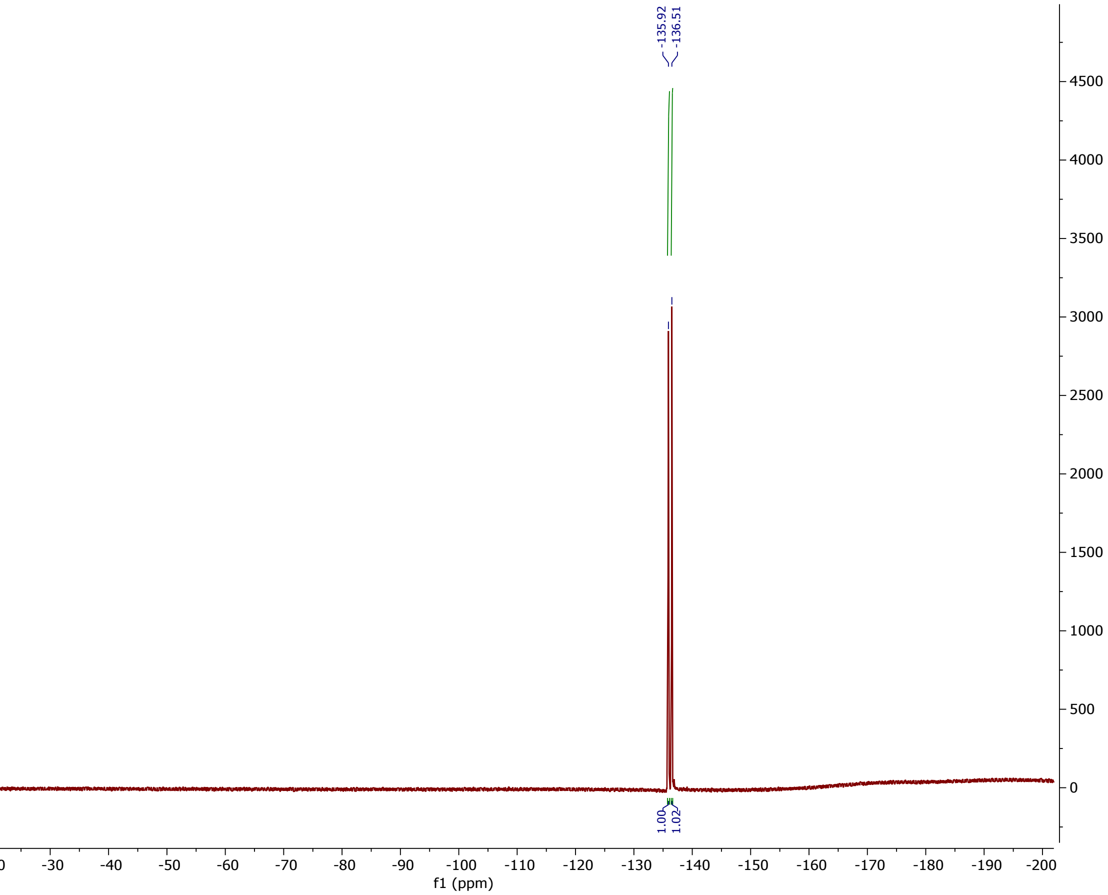

Figure S102. Compound **23** ^{19}H NMR:

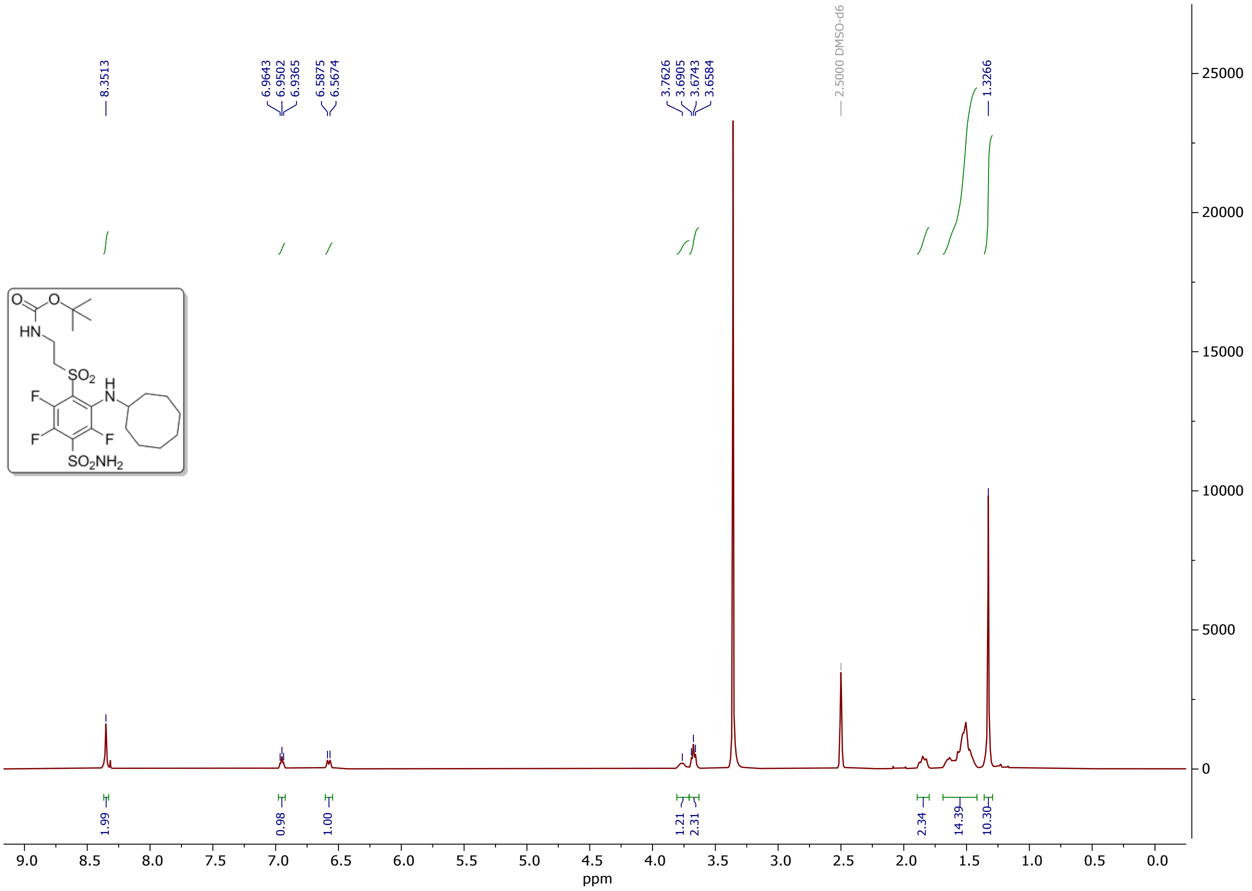

Figure S103. Compound **23** ^{13}C NMR:

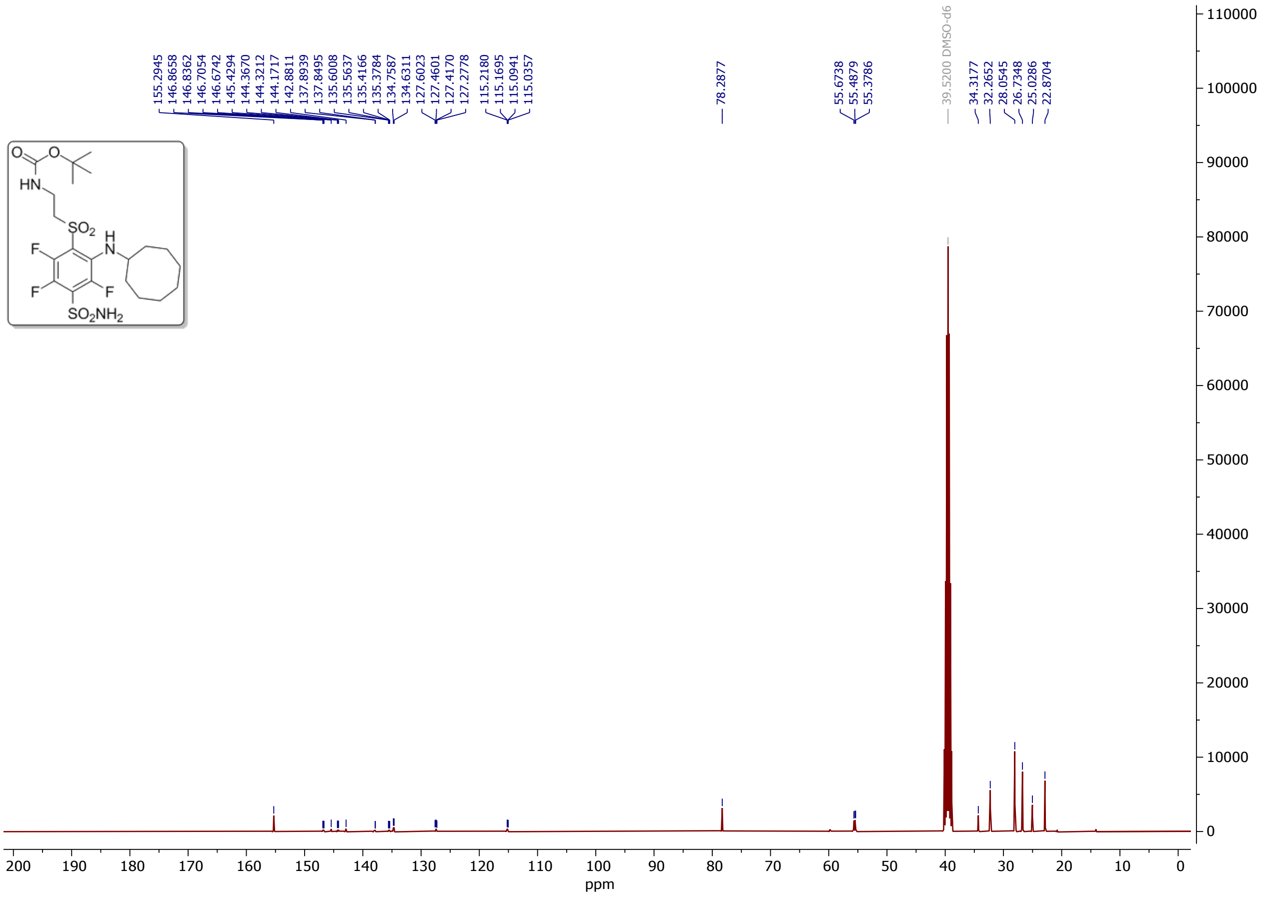

Figure S104. Compound **23** ^{19}F NMR:

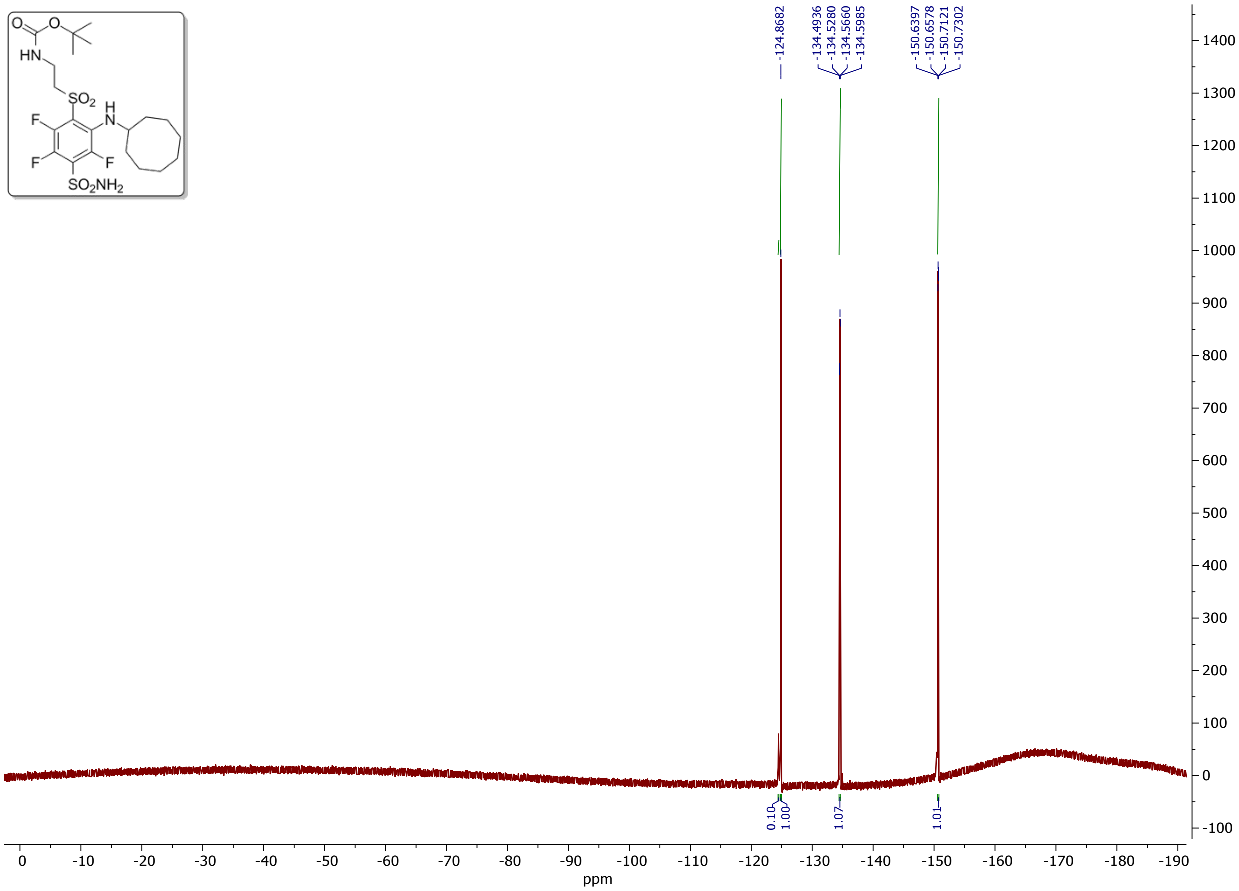

Figure S105. Compound **23** HRMS:

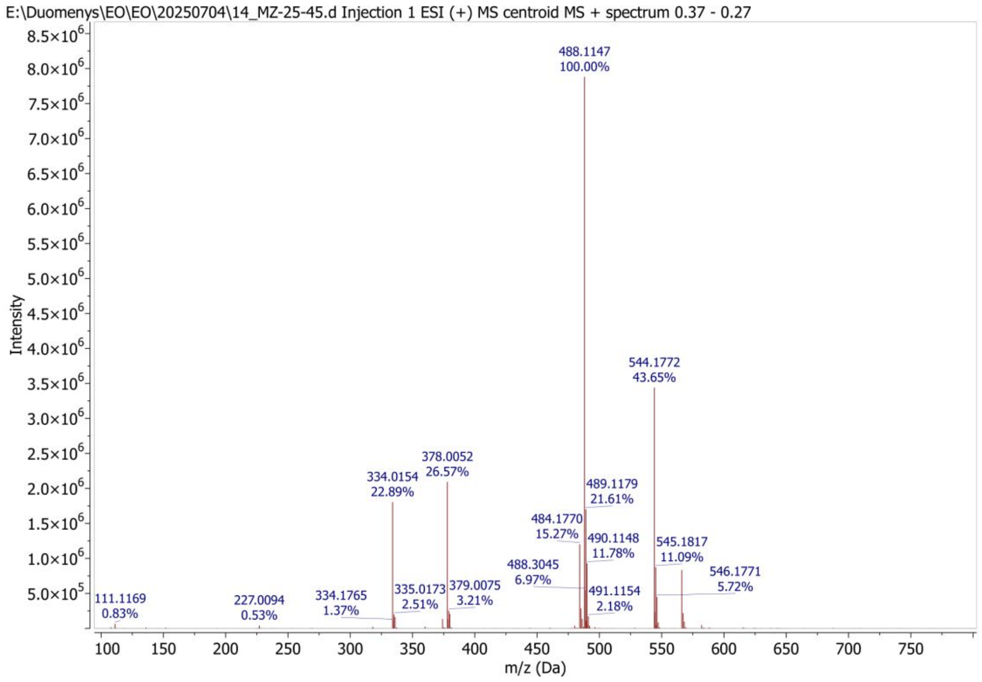

Figure S106. Compound **24** ^{1}H NMR:

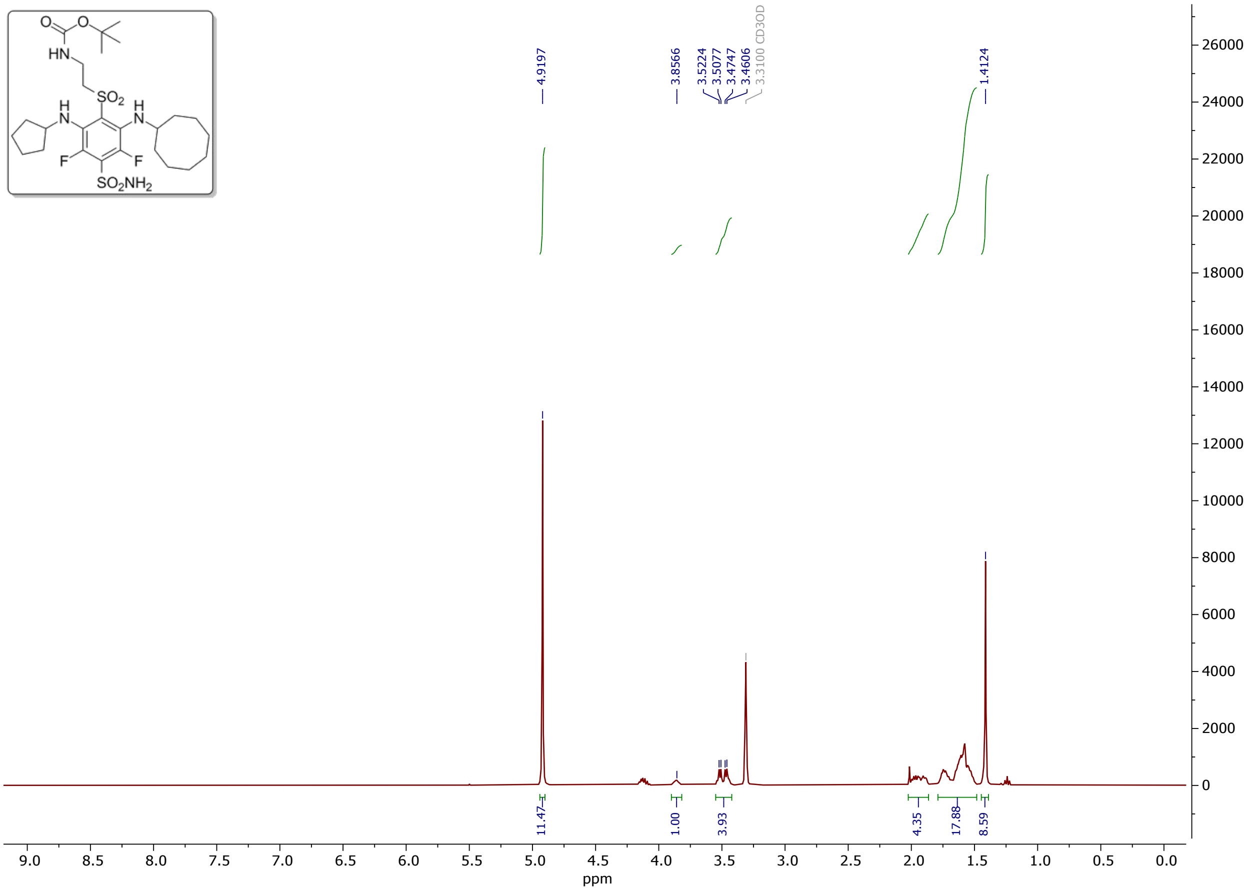

Figure S107. Compound **24** ^{13}C NMR:

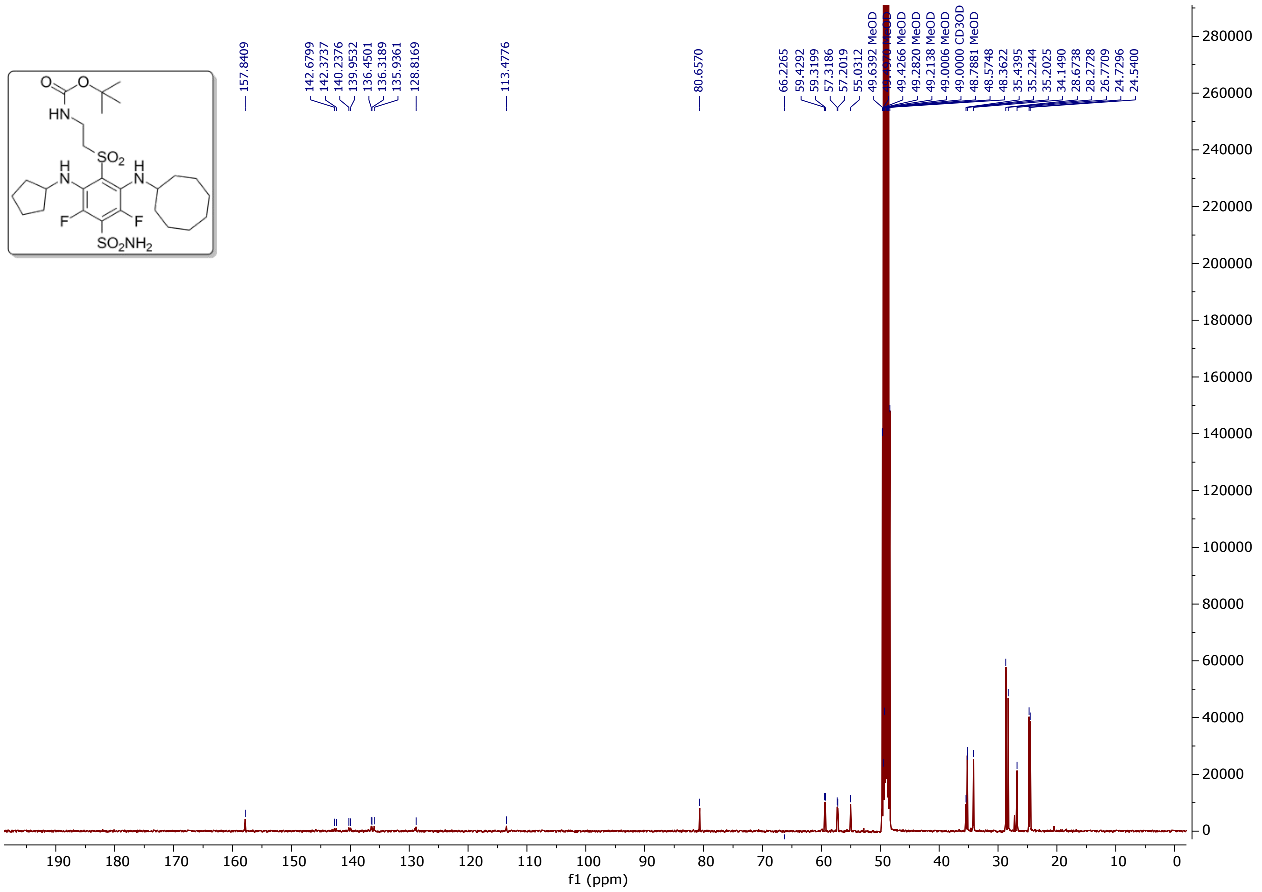

Figure S108. Compound **24** ^{19}F NMR:

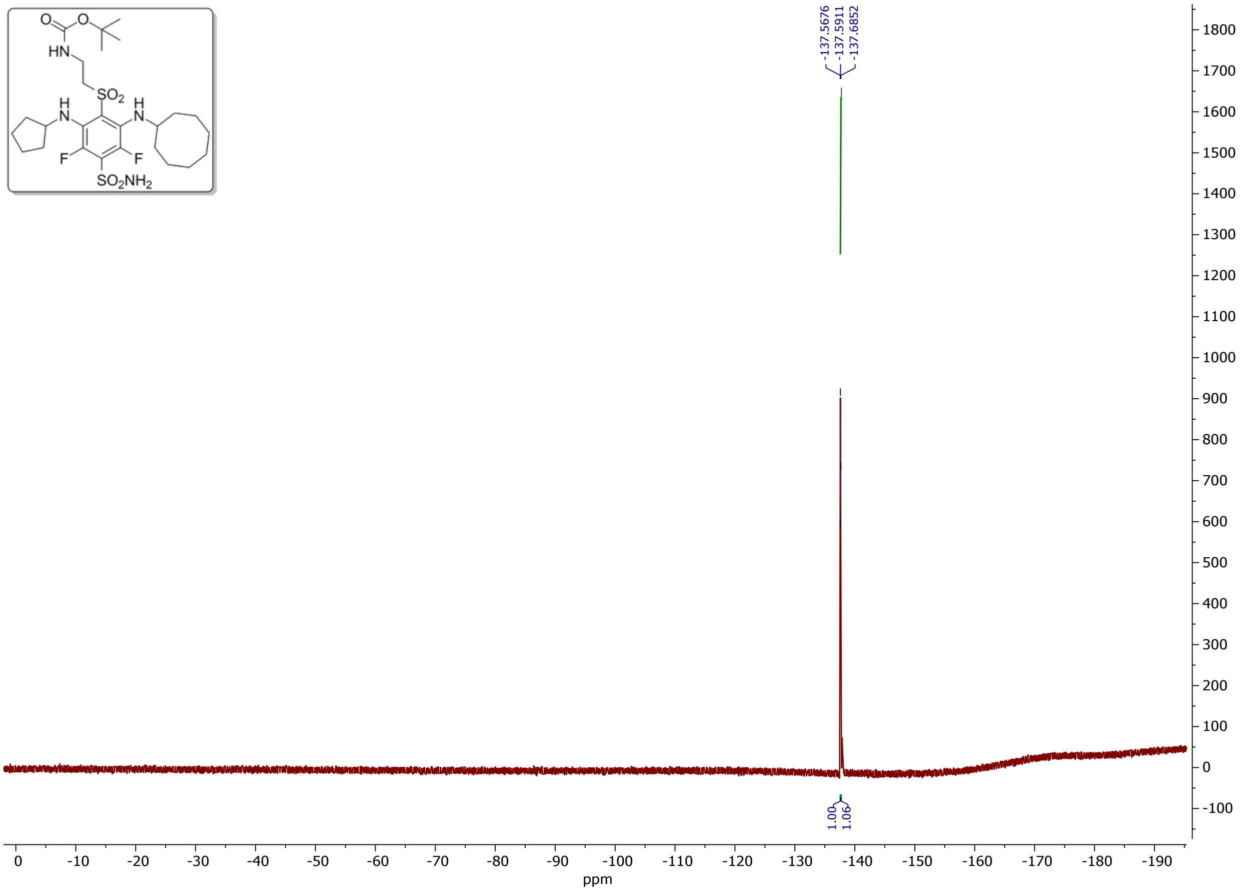

Figure S109. Compound **24** HRMS:

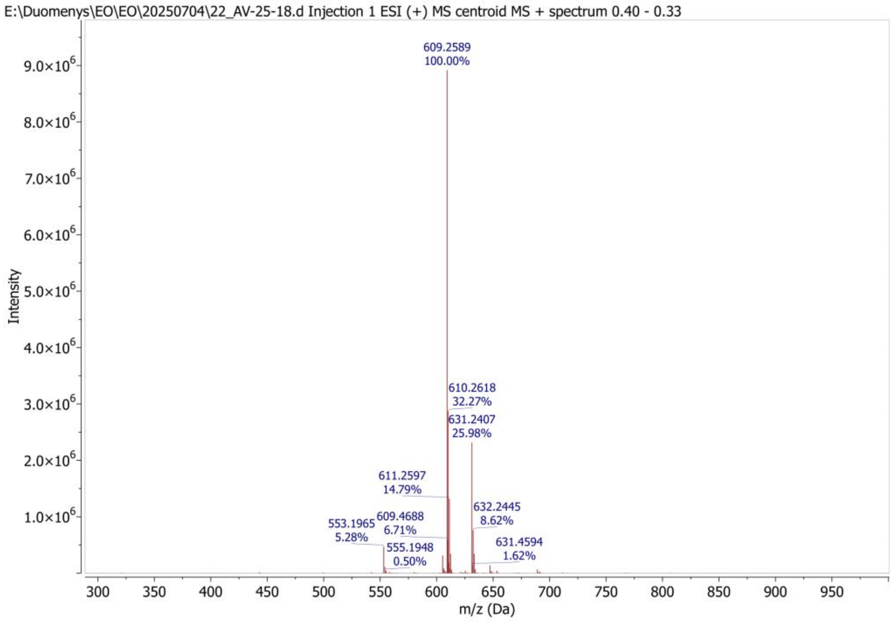

Figure S110. Compound **25** ^{1}H NMR

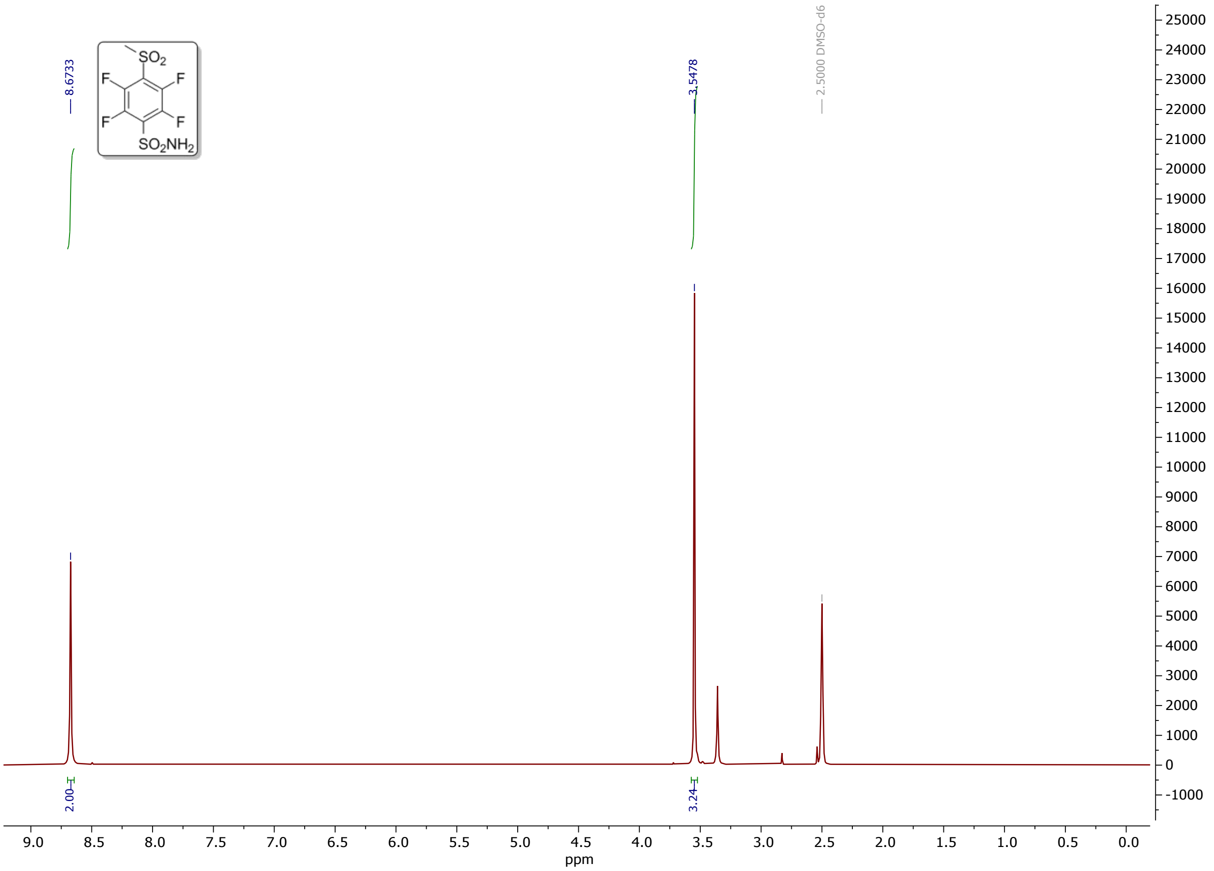

Figure S111. Compound **25** ^{19}F NMR:

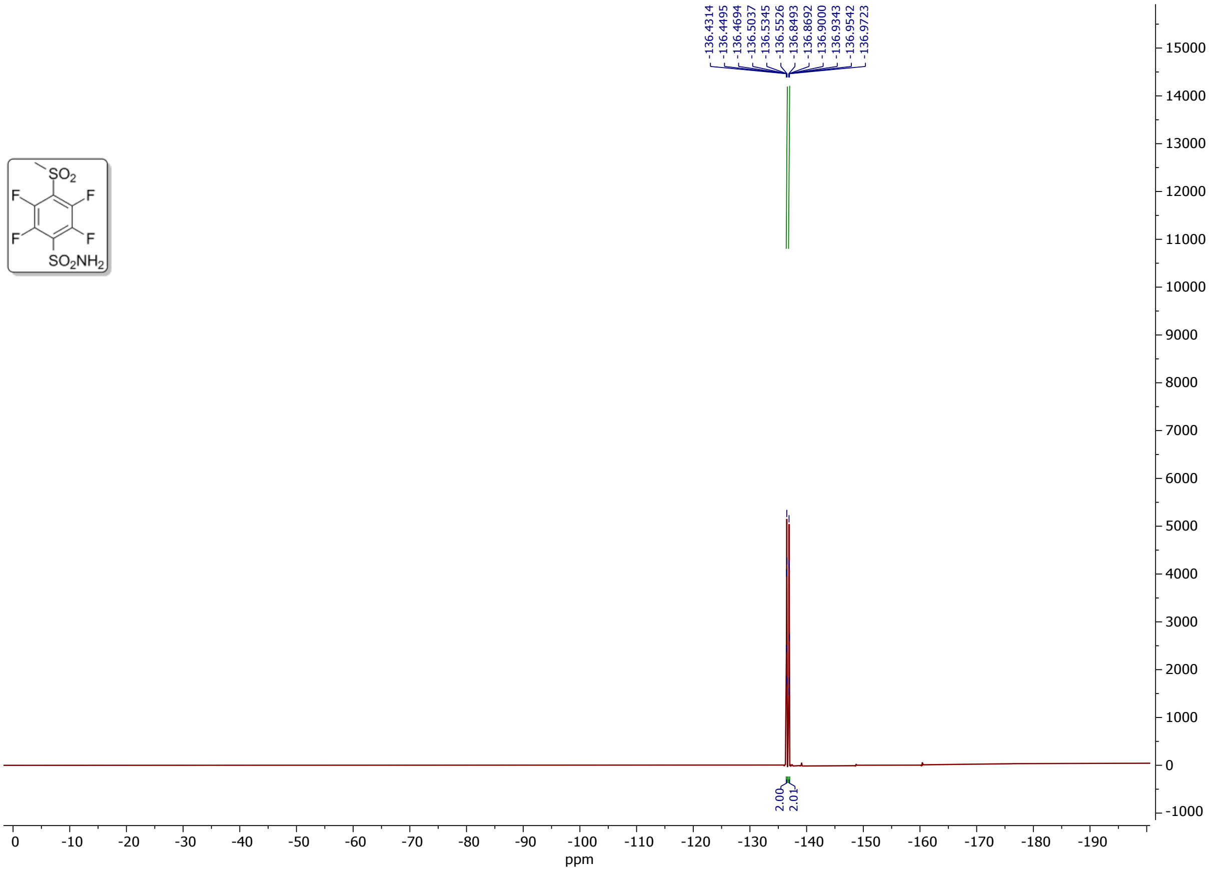

Figure S112. Compound **25** ^{13}C NMR:

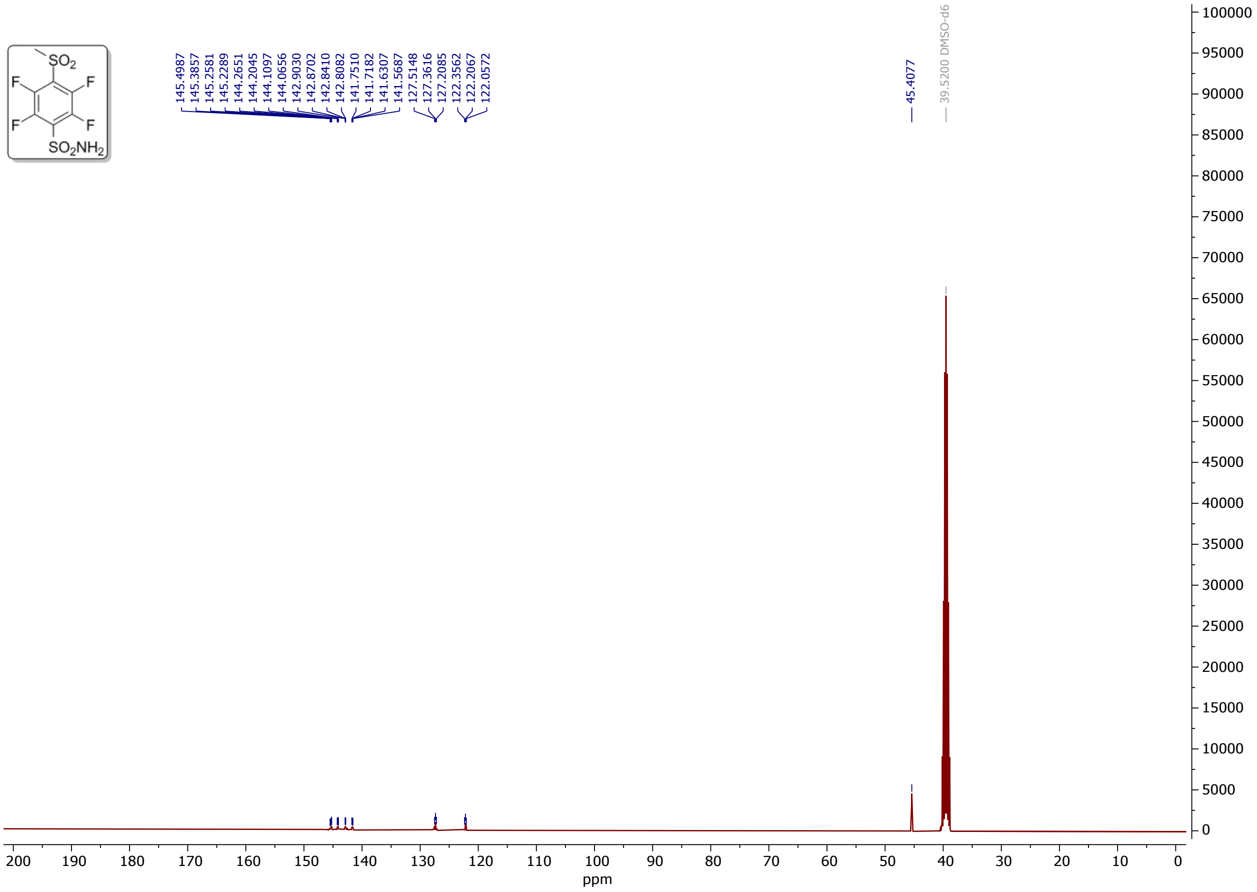

Figure S113. Compound **26** ^{1}H NMR:

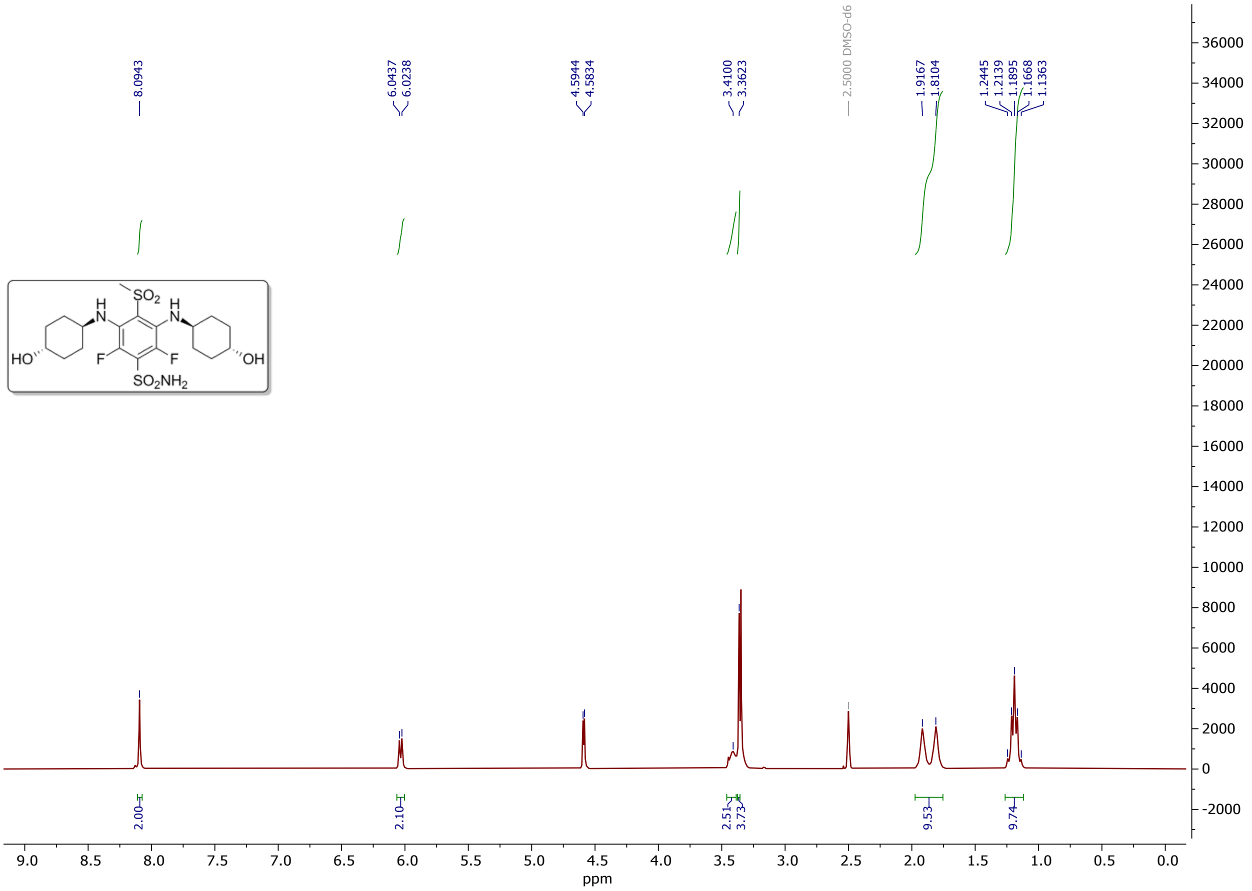

Figure S114. Compound **26** ^{19}F NMR:

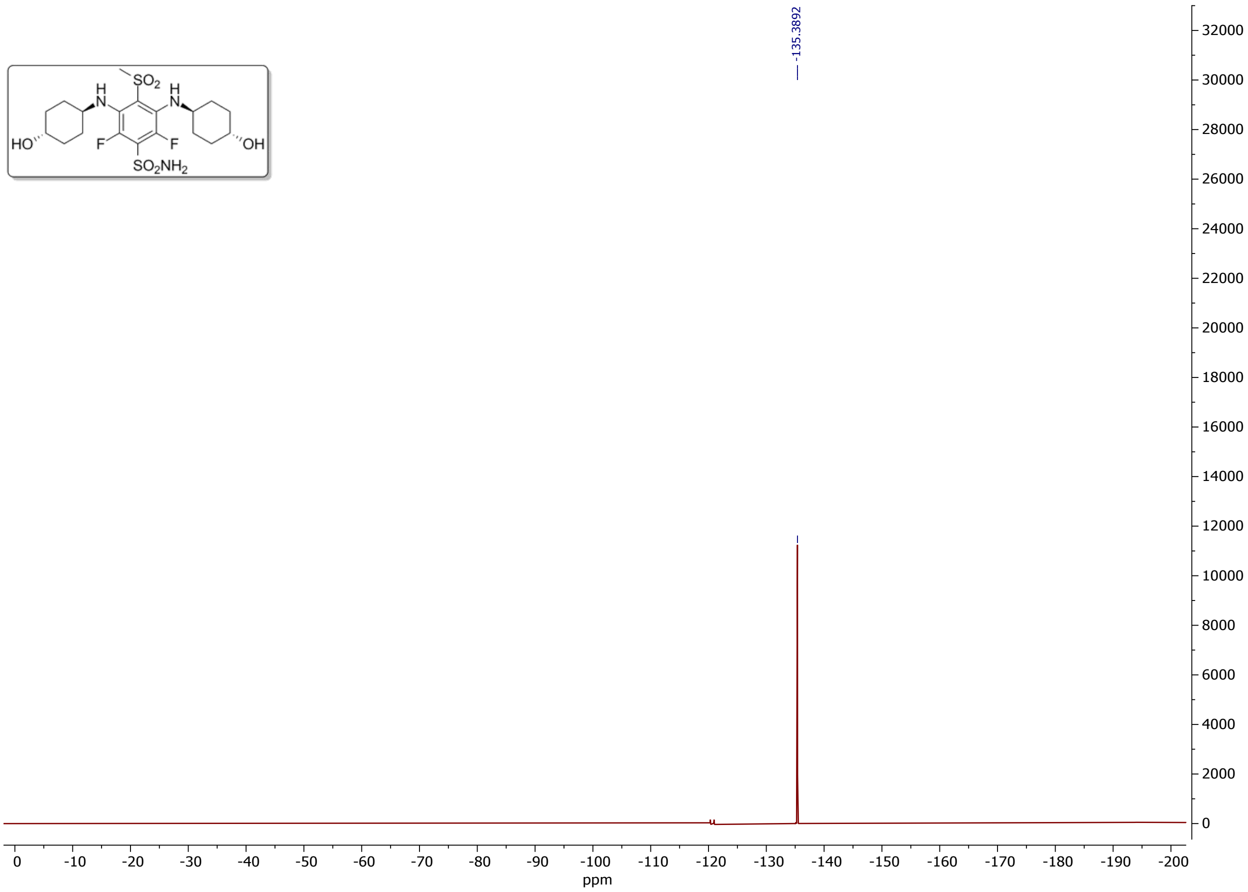

Figure S115. Compound **26** ^{13}C NMR:

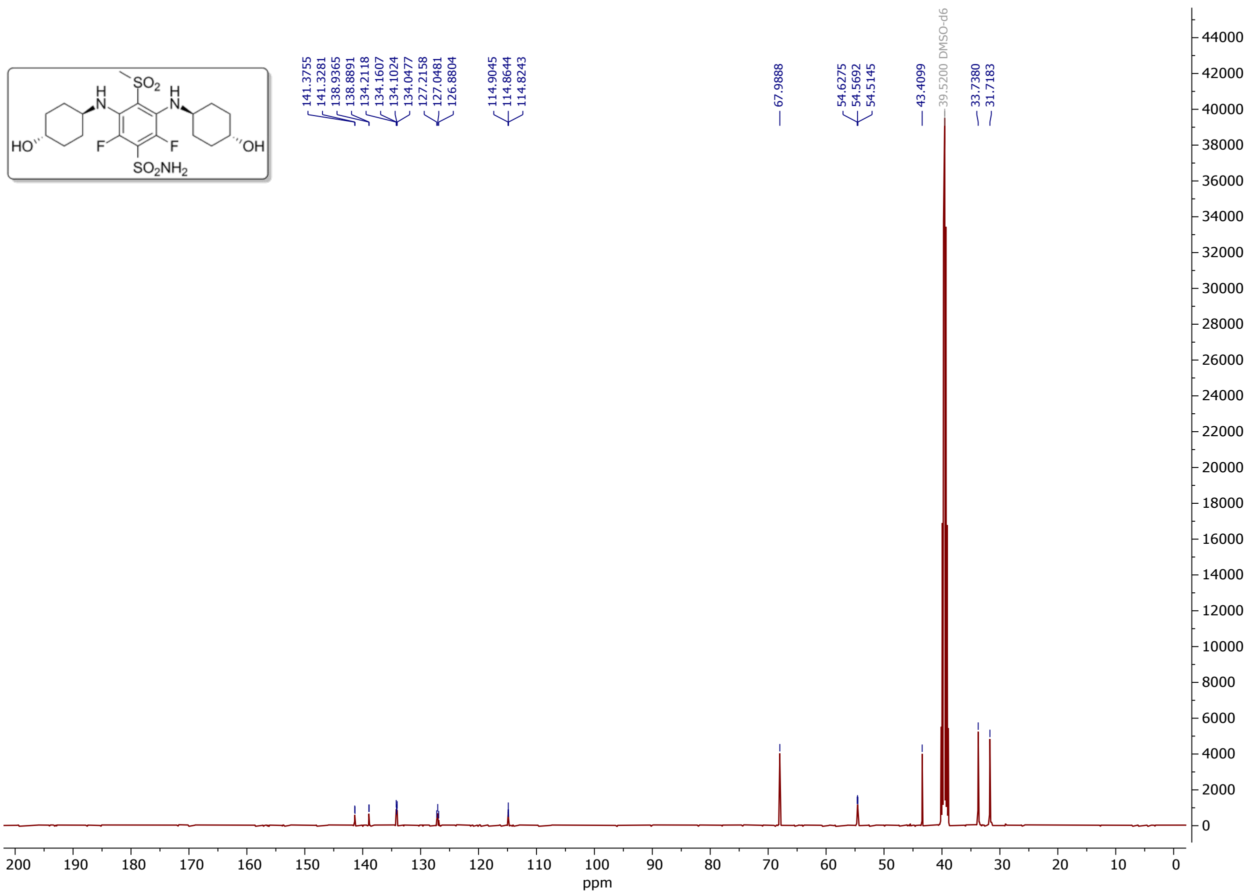

Figure S116. Compound **26** HRMS:

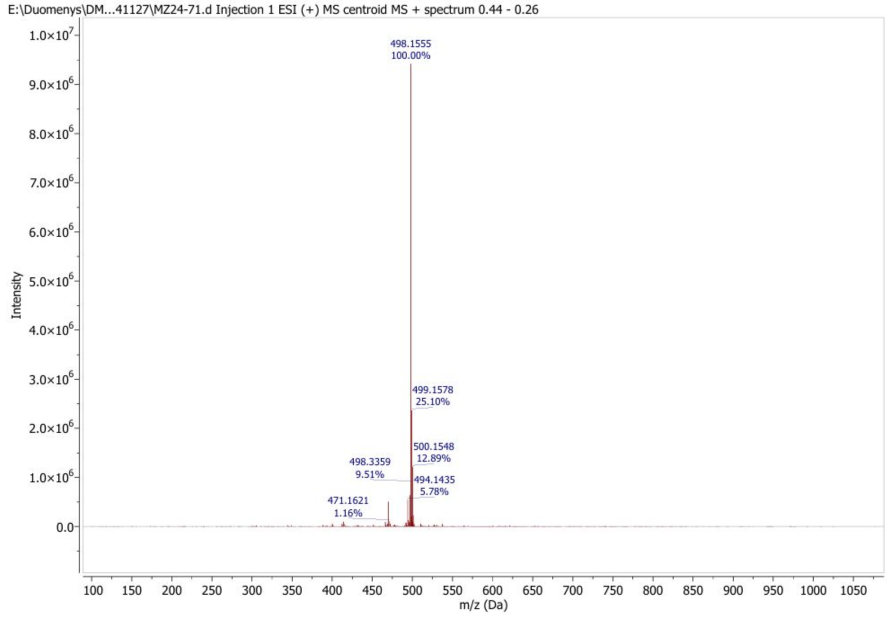

Figure S117. Compound **27** ^{1}H NMR:

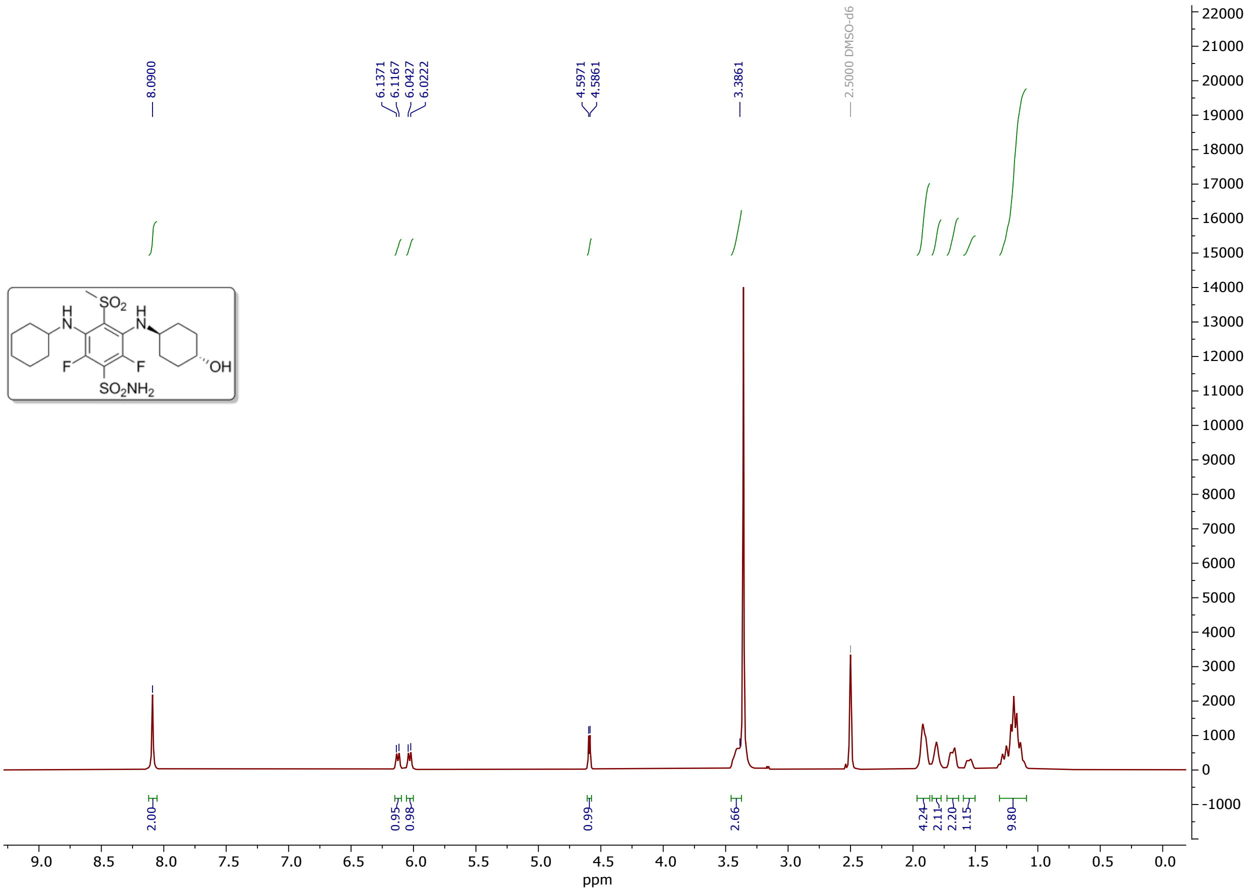

Figure S118. Compound **27** ^{19}F NMR:

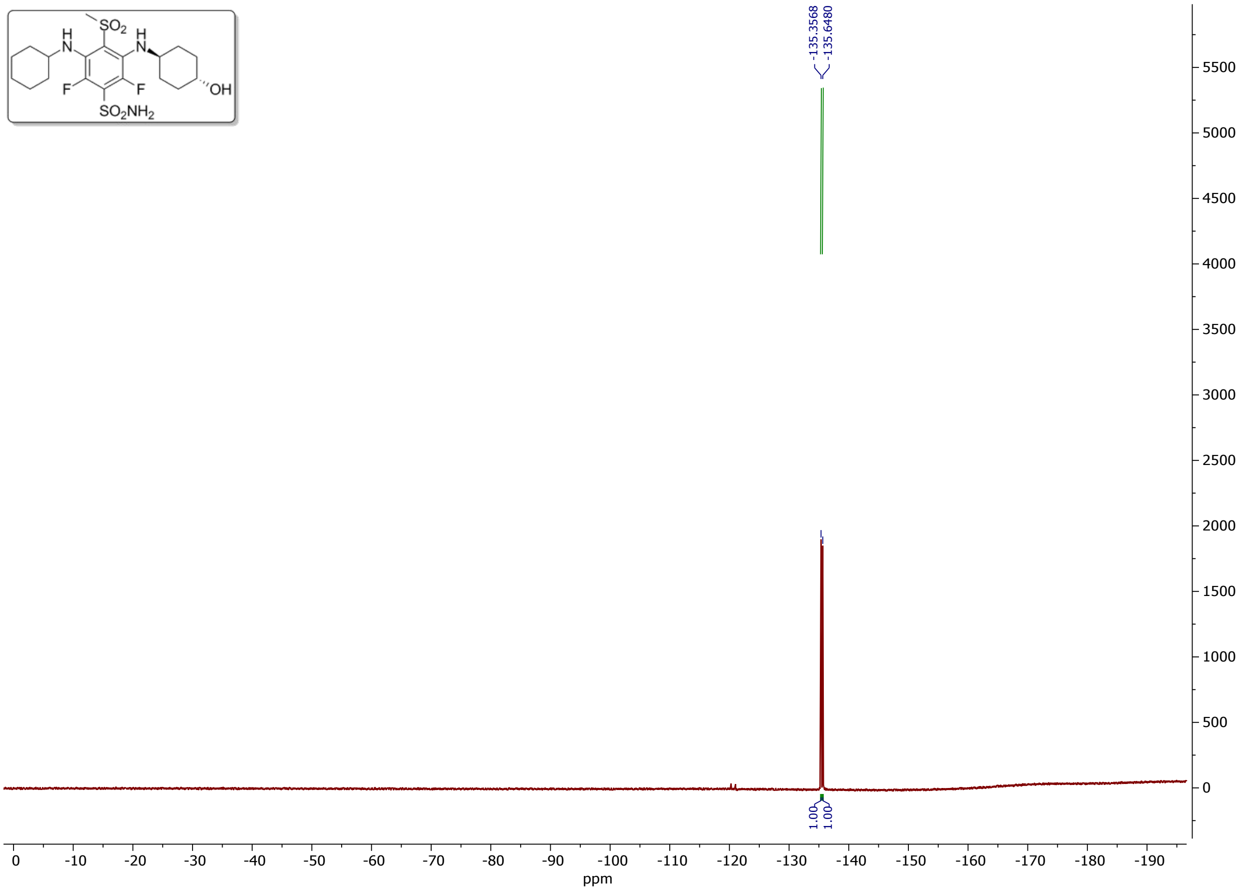

Figure S119. Compound **27** ^{13}C NMR:

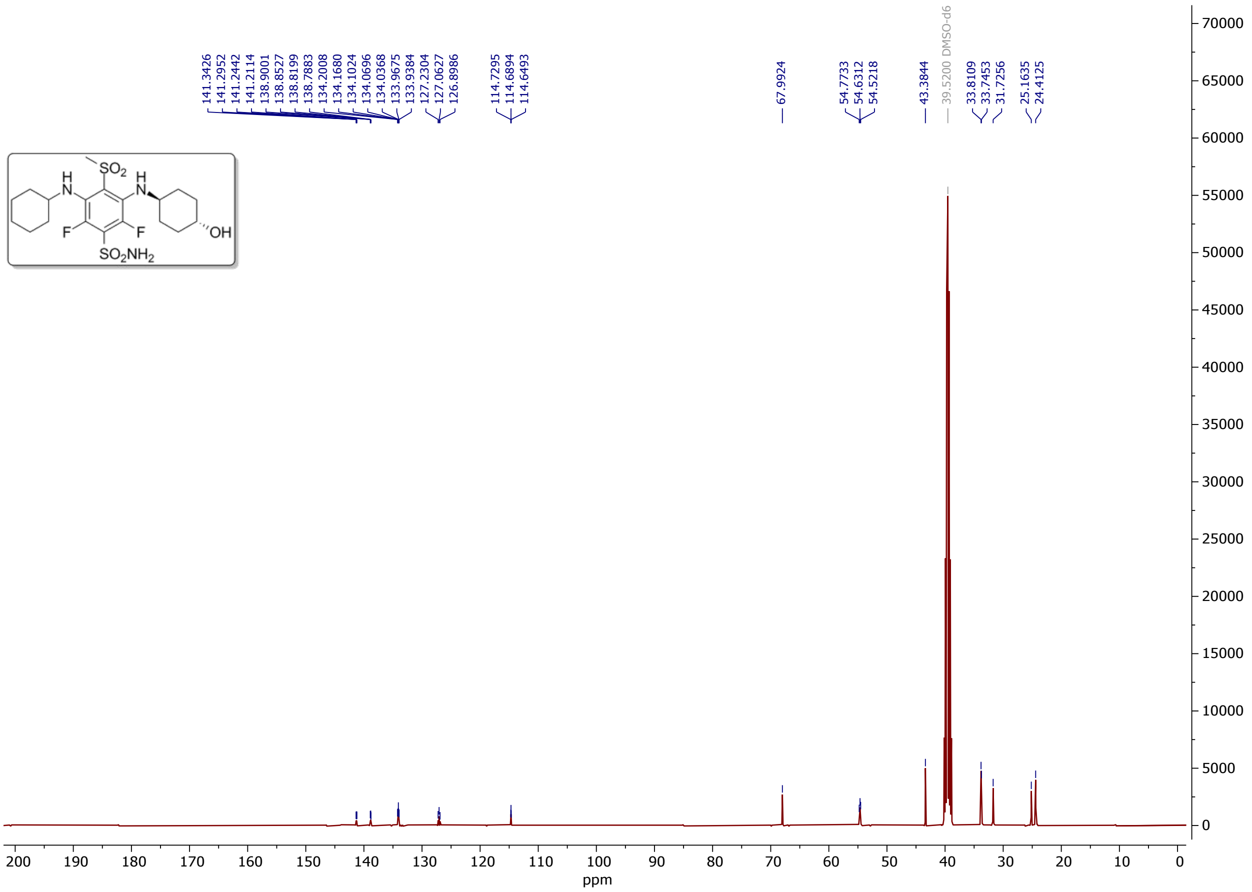

Figure S120. Compound **27** HRMS:

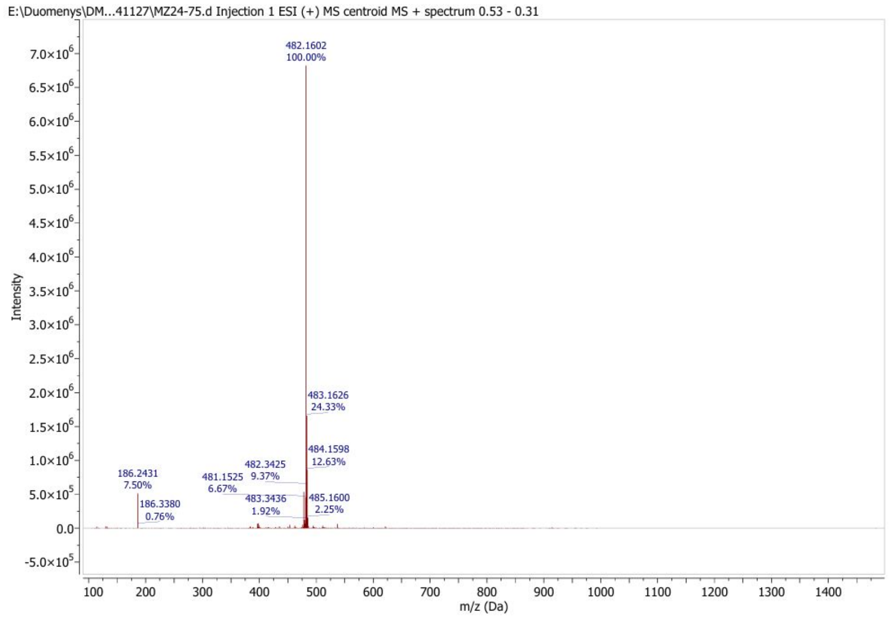

Figure S121. Compound **28** ^{1}H NMR:

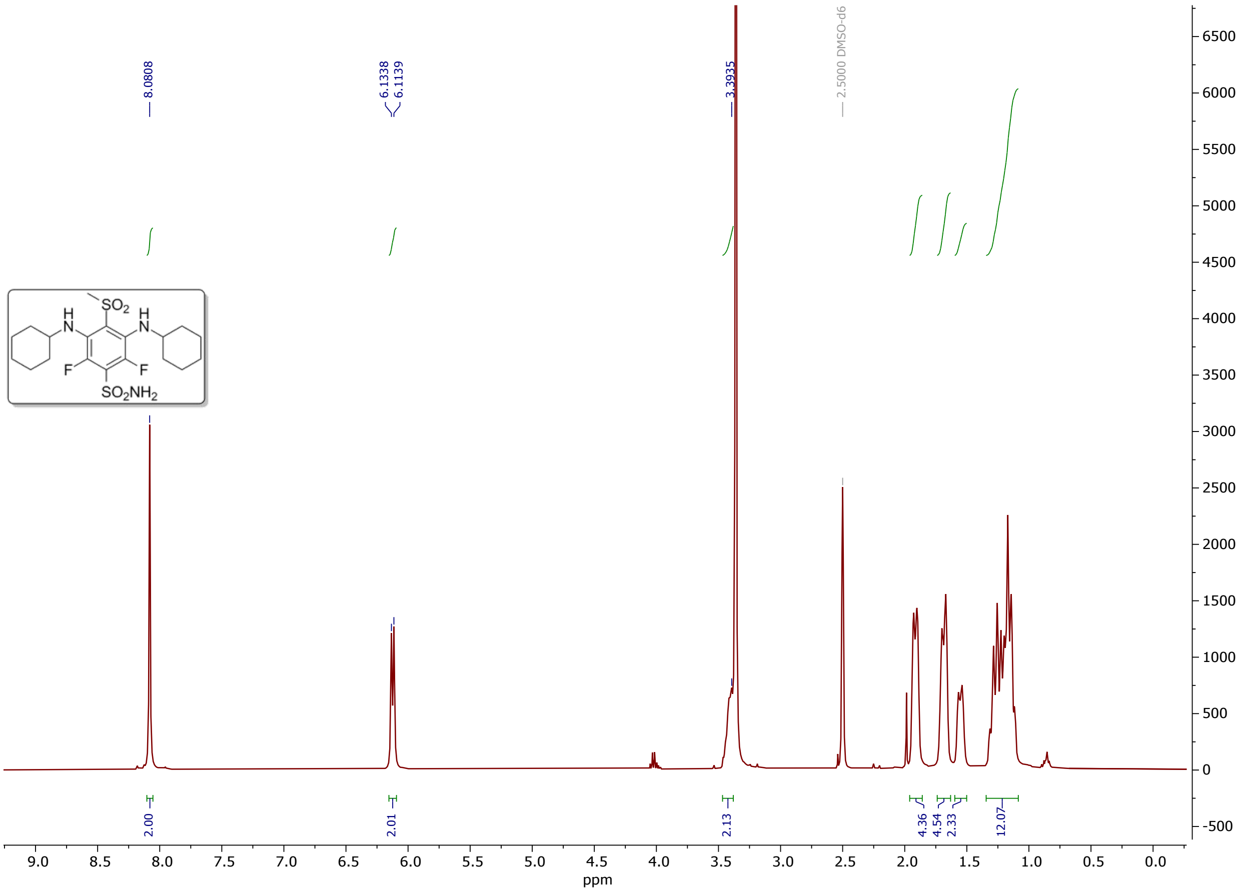

Figure S122. Compound **28** ^{19}F NMR:

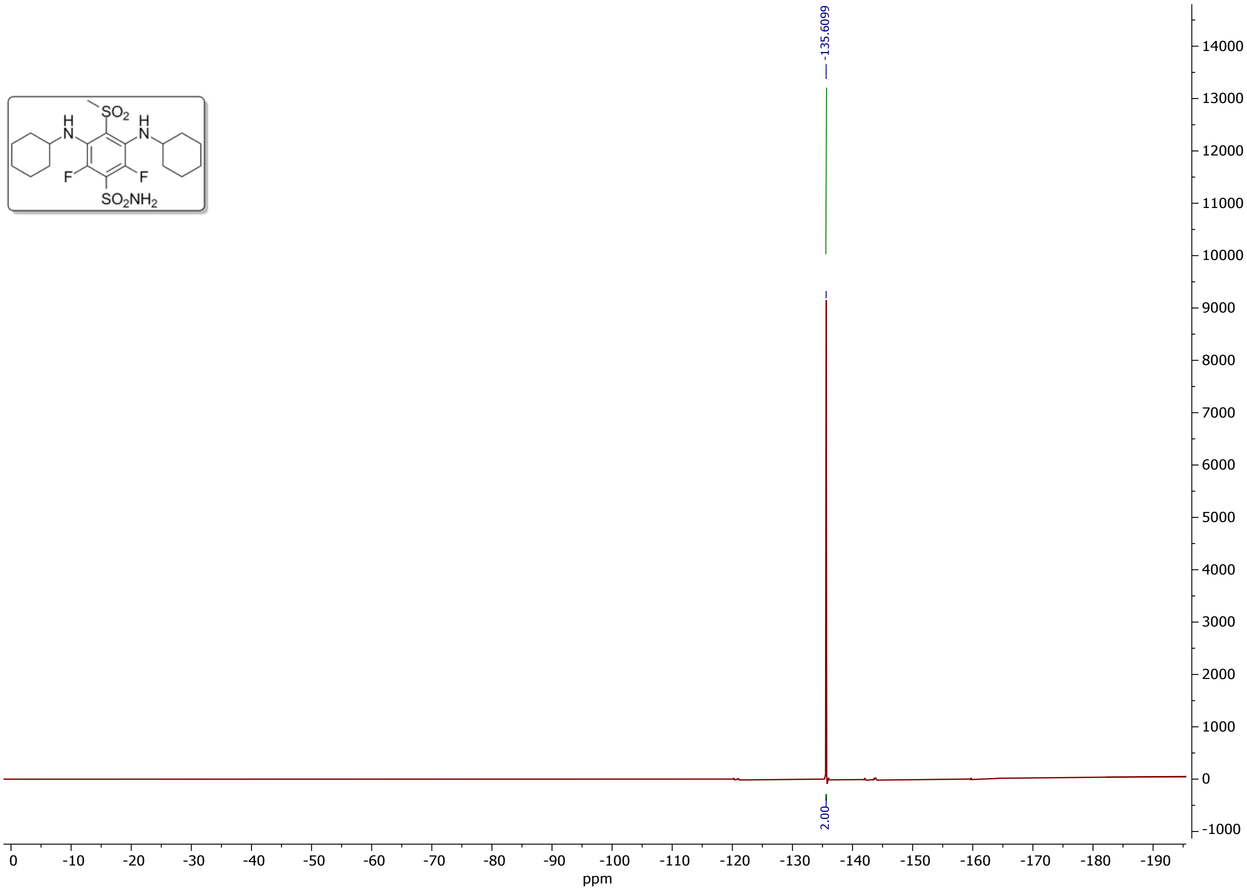

Figure S123. Compound **28** ^{13}C NMR:

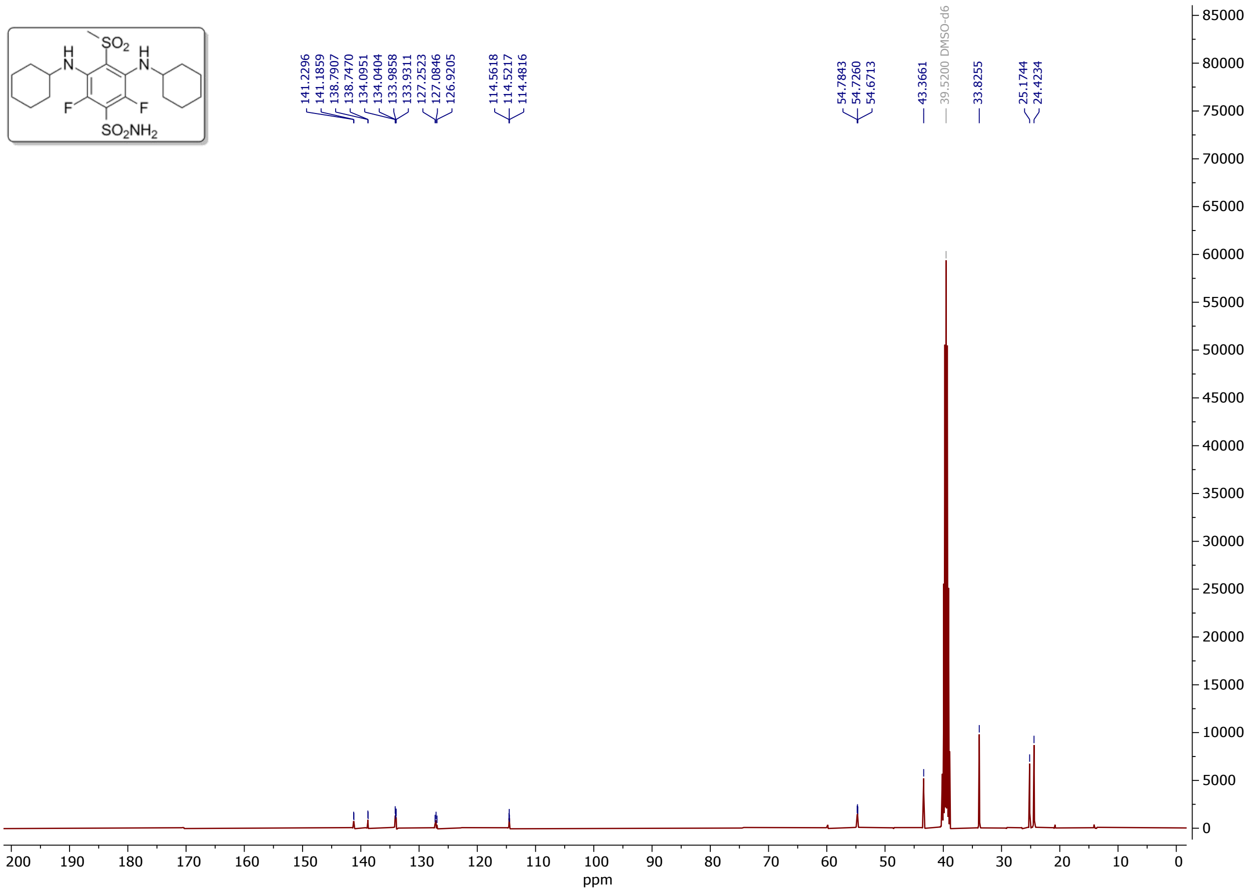

Figure S124. Compound **28** HRMS:

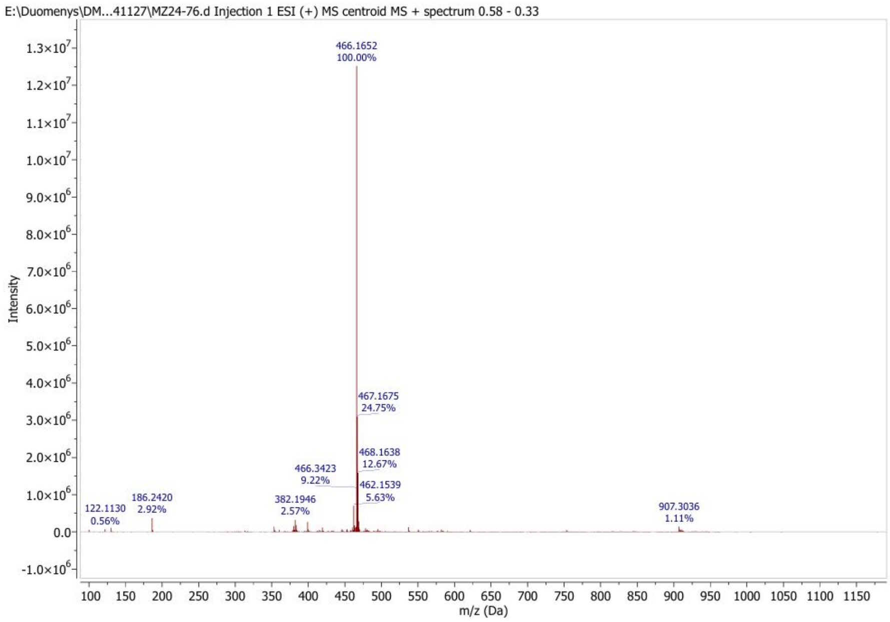

Figure S125. Compound **29** ^{1}H NMR:

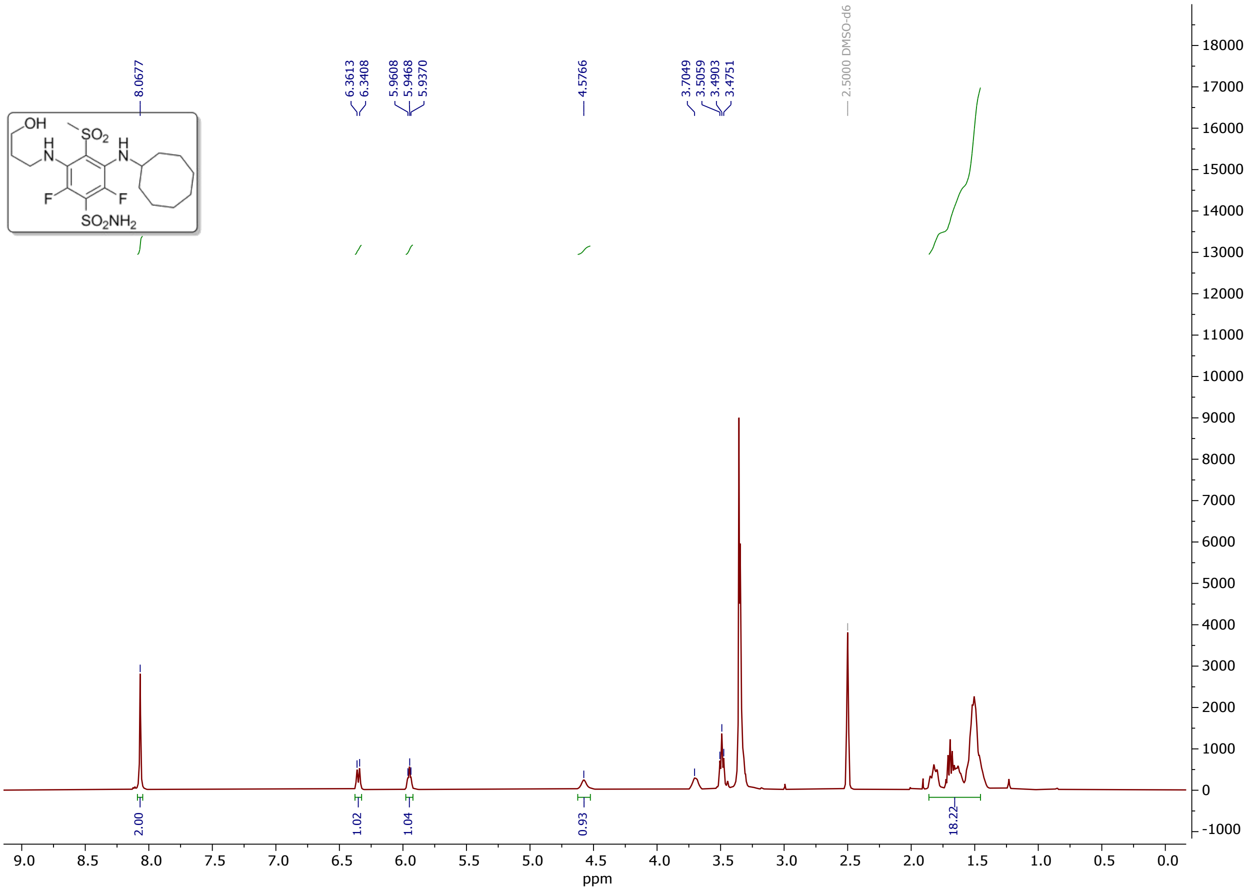

Figure S126. Compound **29** ^{19}F NMR:

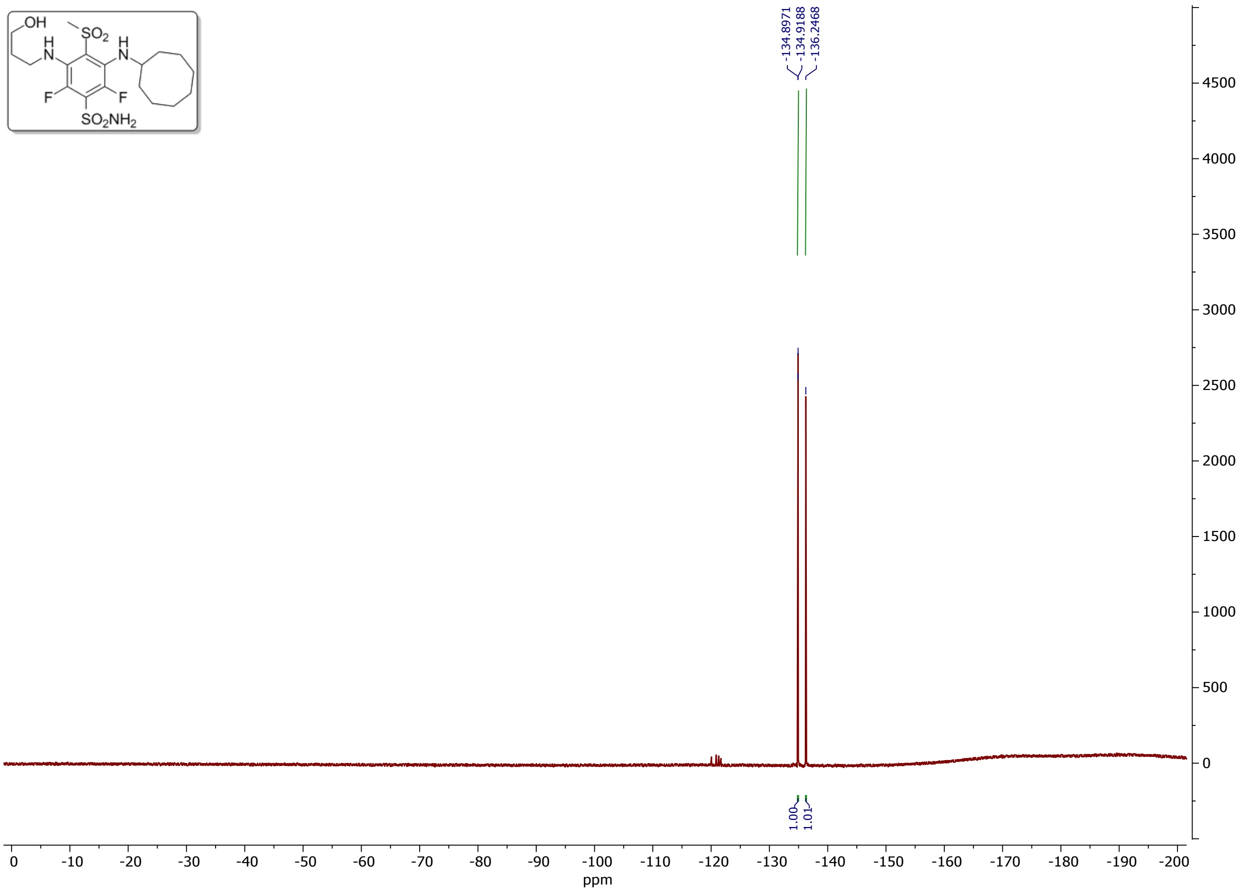

Figure S127. Compound **29** ^{13}C NMR:

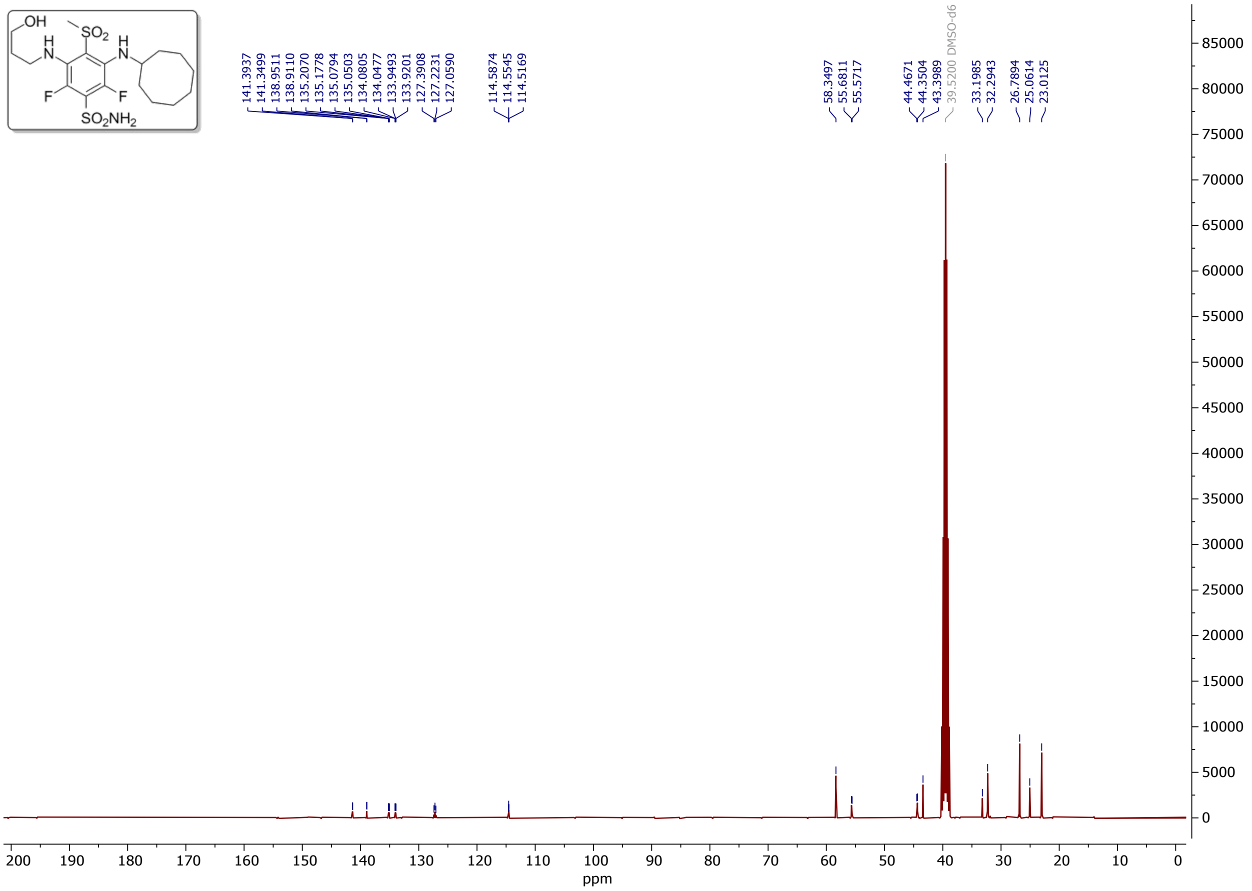

Figure S128. Compound **29** HRMS:

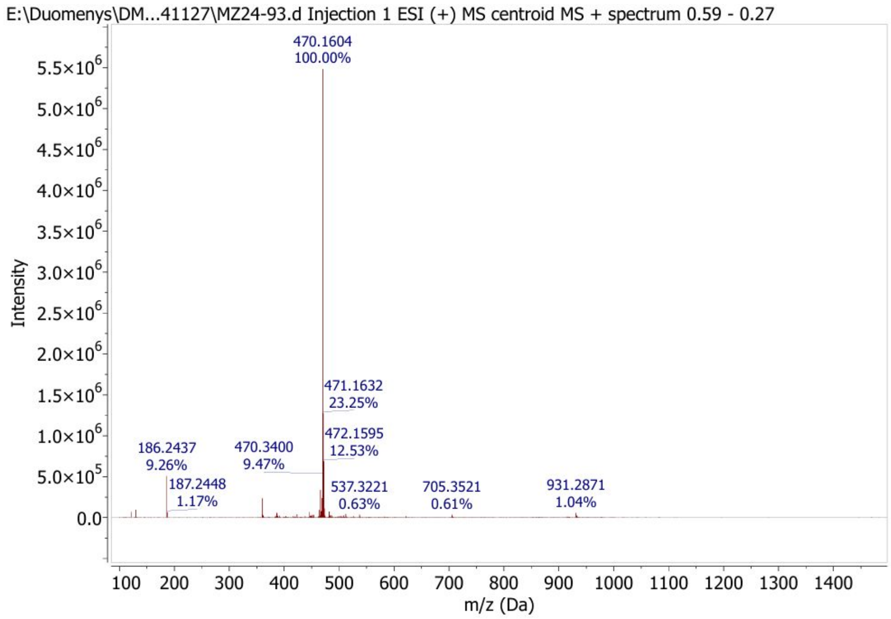

Figure S129. UPLC spectrum of **13**

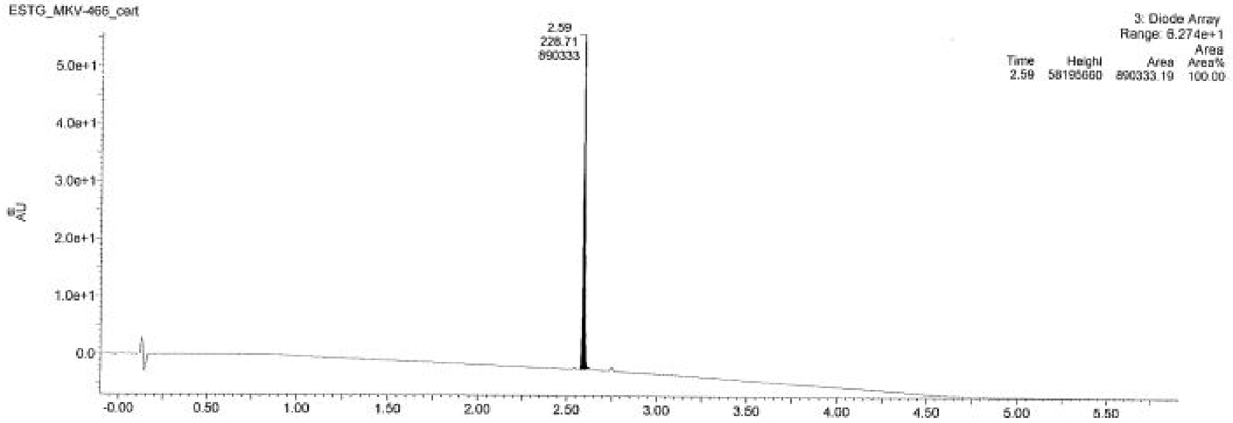

Figure S130. UPLC spectrum of **14**

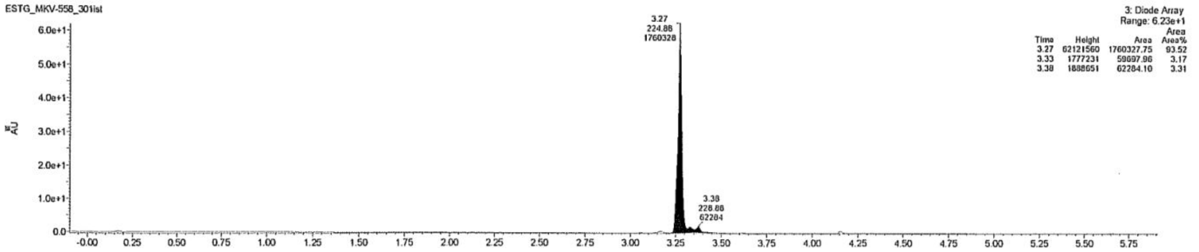

Supplement: Supplementary file 1 [file jm5c01142_si_001.pdf]
